# Block Forests: random forests for blocks of clinical and omics covariate data

**DOI:** 10.1186/s12859-019-2942-y

**Published:** 2019-06-27

**Authors:** Roman Hornung, Marvin N. Wright

**Affiliations:** 1 Institute for Medical Information Processing, Biometry and Epidemiology, University of Munich, Marchioninistr. 15, Munich, 81377 Germany; 2 Leibniz Institute for Prevention Research and Epidemiology – BIPS, Achterstr. 30, Bremen, 28359 Germany; 3 Section of Biostatistics, Department of Public Health, University of Copenhagen, Øster Farimagsgade 5, Copenhagen, 1014 Denmark

**Keywords:** Multi-omics data, Prediction, Random forest, Machine learning, Statistics, Survival analysis, Cancer

## Abstract

**Background:**

In the last years more and more multi-omics data are becoming available, that is, data featuring measurements of several types of omics data for each patient. Using multi-omics data as covariate data in outcome prediction is both promising and challenging due to the complex structure of such data. Random forest is a prediction method known for its ability to render complex dependency patterns between the outcome and the covariates. Against this background we developed five candidate random forest variants tailored to multi-omics covariate data. These variants modify the split point selection of random forest to incorporate the block structure of multi-omics data and can be applied to any outcome type for which a random forest variant exists, such as categorical, continuous and survival outcomes. Using 20 publicly available multi-omics data sets with survival outcome we compared the prediction performances of the block forest variants with alternatives. We also considered the common special case of having clinical covariates and measurements of a single omics data type available.

**Results:**

We identify one variant termed “block forest” that outperformed all other approaches in the comparison study. In particular, it performed significantly better than standard random survival forest (adjusted *p*-value: 0.027). The two best performing variants have in common that the block choice is randomized in the split point selection procedure. In the case of having clinical covariates and a single omics data type available, the improvements of the variants over random survival forest were larger than in the case of the multi-omics data. The degrees of improvements over random survival forest varied strongly across data sets. Moreover, considering all clinical covariates mandatorily improved the performance. This result should however be interpreted with caution, because the level of predictive information contained in clinical covariates depends on the specific application.

**Conclusions:**

The new prediction method block forest for multi-omics data can significantly improve the prediction performance of random forest and outperformed alternatives in the comparison. Block forest is particularly effective for the special case of using clinical covariates in combination with measurements of a single omics data type.

**Electronic supplementary material:**

The online version of this article (10.1186/s12859-019-2942-y) contains supplementary material, which is available to authorized users.

## Background

In the last decade the measurement of various types of omics data, such as gene expression, methylation or copy number variation data has become increasingly fast and cost-effective. Therefore, there exist more and more patient data for which several types of omics data are available for the same patients. In the following, such data are denoted as multi-omics data and the different subsets of this data containing the individual data types are referred to as “blocks”. Using multi-omics data in prediction modeling is promising because each type of omics data may contribute information valuable for the prediction of phenotypic outcomes. However, combining different types of omics data effectively is challenging for several reasons. First, the predictive information contained in the individual blocks is overlapping. Second, the levels of predictive information differ between the blocks and depend on the particular outcome considered [1]. Third, there exist interactions between variables across the different blocks, which should be taken into account [2].

While pioneering work in the area of prediction modelling using multi-omics covariate data was already published as early as 2004 [3], further methodological developments in this area rarely seem to have been pursued until the last several years. A PubMed search for the term “multi-omics” resulted in two papers from the year 2006 (first mentioning of the term), five papers from the year 2010, but 368 papers from the year 2018. This long-lasting lack of prediction methods tailored to multi-omics covariate data was probably due to the fact that multi-omics data had not been available on a larger scale until recently. Simon et al. [4] presented the sparse group lasso in 2013, a prediction method for grouped covariate data that automatically removes non-informative covariate groups and performs lasso-type variable selection for the remaining covariate groups. A disadvantage of the sparse lasso in applications to multi-omics data is that it does not explicitly take the different levels of predictive information of the blocks into account. This is different for the IPF-LASSO [5], a lasso-type regression method for multi-omics data in which each block is associated with an individual penalty parameter. Vazquez et al. [6] model the relationship between phenotypic outcomes and multi-omics covariate data fully Bayesian using a Bayesian generalized additive model. Mankoo et al. [7] consider a two-step approach: In the first step they aim to remove redundancies between the different blocks by filtering out highly correlated pairs of variables from different blocks and in the second step they apply standard *L*_1_ regularized Cox regression [8] using the remaining variables. Seoane et al. [9] use multiple kernel learning methods, considering composite kernels as linear combinations of base kernels derived from each block, where they incorporate pathway information in the selection of relevant variables. Similarly, Fuchs et al. [10] consider combining classifiers, each learned using one of the blocks. In the context of a comparison study, Boulesteix et al. [5] again consider an approach based on combining prediction rules, each learned using a single block: first, lasso is fitted to each block and, second, the resulting linear predictors are used as covariates in a low-dimensional regression model. In addition to the approach mentioned above, Fuchs et al. [10] also consider performing variable selection separately for each block and then learning a single classifier using all blocks. Klau et al. [11] present the priority-Lasso, a lasso-type prediction method for multi-omics data that differs from the approaches described above in that its main focus is not prediction accuracy but applicability from a practical point of view: with this method the user has to provide a priority order of the blocks that is for example motivated by the costs of generating each type of data. Blocks of low priority are likely to be automatically excluded by this method, which should frequently lead to prediction rules that are easy to apply in practice and, at the same time, feature a high prediction accuracy. A related method is the TANDEM approach [12], which attributes a lower priority to gene expressions than to the other omics data types in order to avoid the prediction rule to be strongly dominated by the gene expressions. For a recent overview of approaches for analyzing multi-omics data focused on data mining see Huang et al. [2]. Recently, a multi-omics deep learning approach, SALMON [13], was suggested that employs eigengenes in neural networks to learn hidden layers, which are subsequently used in Cox regression. A supervised dimension reduction method for multi-omics data called Integrative SIR (ISIR) was also proposed recently [14]. Beyond conventional prediction modelling, the integrative analysis of different omics data types can deliver new insights into biomedical processes; see, for example, Yaneske & Angione [15] for a multi-omics based assessment procedure of biological age of humans. An extensive review of various statistical procedures commonly used in practice in the analysis of multi-omics data can be found in Wu et al. [16].

Apart from multi-omics data, in most cases where a certain type of omics data is available, the corresponding phenotypic data set features several clinical covariates. The latter are often of great prognostic relevance and should be prioritized over or at least be used in addition to the omics data. Many of the methods described above can be used for such data as well, if the clinical data is treated as an omics data type from a methodological point of view. However, since this problem was known before the rise of multi-omics data, there also exist various strategies for effectively using the clinical information in combination with a single omics data type. See Boulesteix & Sauerbrei [17] for a detailed discussion of such approaches and De Bin et al. [18] for a comparison study illustrating their application.

The random forest algorithm [19] is a powerful prediction method that is known to be able to capture complex dependency patterns between the outcome and the covariates. The latter feature makes random forest a promising candidate for developing a prediction method tailored to the challenges of multi-omics data. In this paper we set out to develop such a variant, where we initially consider five different candidate methods. Each of these five considered random forest variants differs from conventional random forests merely with respect to the selection of the split points in the decision trees constituting the forests. Therefore, most other components of a random forest are unchanged and each of these five variants can be applied to any outcome type, for which there exists a random forest variant, e.g., categorial, continuous and survival outcomes. Using 20 real multi-omics data sets with survival outcome we compared the prediction performances of the five variants with each other and with alternatives, including random survival forest (RSF) [20] as a reference method. RSF is known to be a strong prediction method for survival outcomes, see for example Bou-Hamad et al. [21] and Yosefian et al. [22] and the references therein. In this comparison study we identified one particularly well performing variant that performed best, both when considering all blocks and for the special case of having only clinical information and a single omics data type. This variant is denoted as the block forest algorithm in the following. We implemented all five variants for categorical, continuous and survival outcomes in our R package blockForest (version 0.2.0) available from CRAN (https://cran.r-project.org/package=blockForest, GitHub version: https://github.com/bips-hb/blockForest), where block forest is used by default. The other variants should be considered with caution only as these may deliver worse prediction results. In an additional analysis, we considered variations of the five variants that include all clinical covariates mandatorily in the split point selection (see “Outlook: Prioritizing the clinical covariates by including them mandatorily in the split point selection” subsection of “Results” section).

We present all five considered variants in the paper and the results obtained for these. As noted above, we recommend the variant that performed best in the comparison for use in practice. Merely reporting the results obtained with the variant that performed best would have been associated with overoptimism, similar to that associated with reporting only significant results after performing several statistical tests.

The paper is structured as follows. In the next section, the results of the comparison study are presented and described. In “Discussion” section, we summarize our main results and draw specific conclusions from the results of the comparison study. The main conclusions are summarized in “Conclusions” section. Finally, in “Methods” section, after briefly describing the multi-omics data format and the splitting procedure performed by standard random forest, we detail each of the five considered random forest variants for multi-omics data. Here, we also discuss the rationale behind each procedure. Lastly, we describe the design of the comparison study.

## Results

In the following, we will present and evaluate the results of our comparison study. As noted above, detailed descriptions of the design of the comparison study and the five considered variants are given in “Methods” section. The five variants are denoted as VarProb, SplitWeights, BlockVarSel, RandomBlock, and BlockForest. Consulting “Methods” section before reading the current section further should make it easier to follow the results presented in the following.

### Multi-omics data

**Fig. 1:**
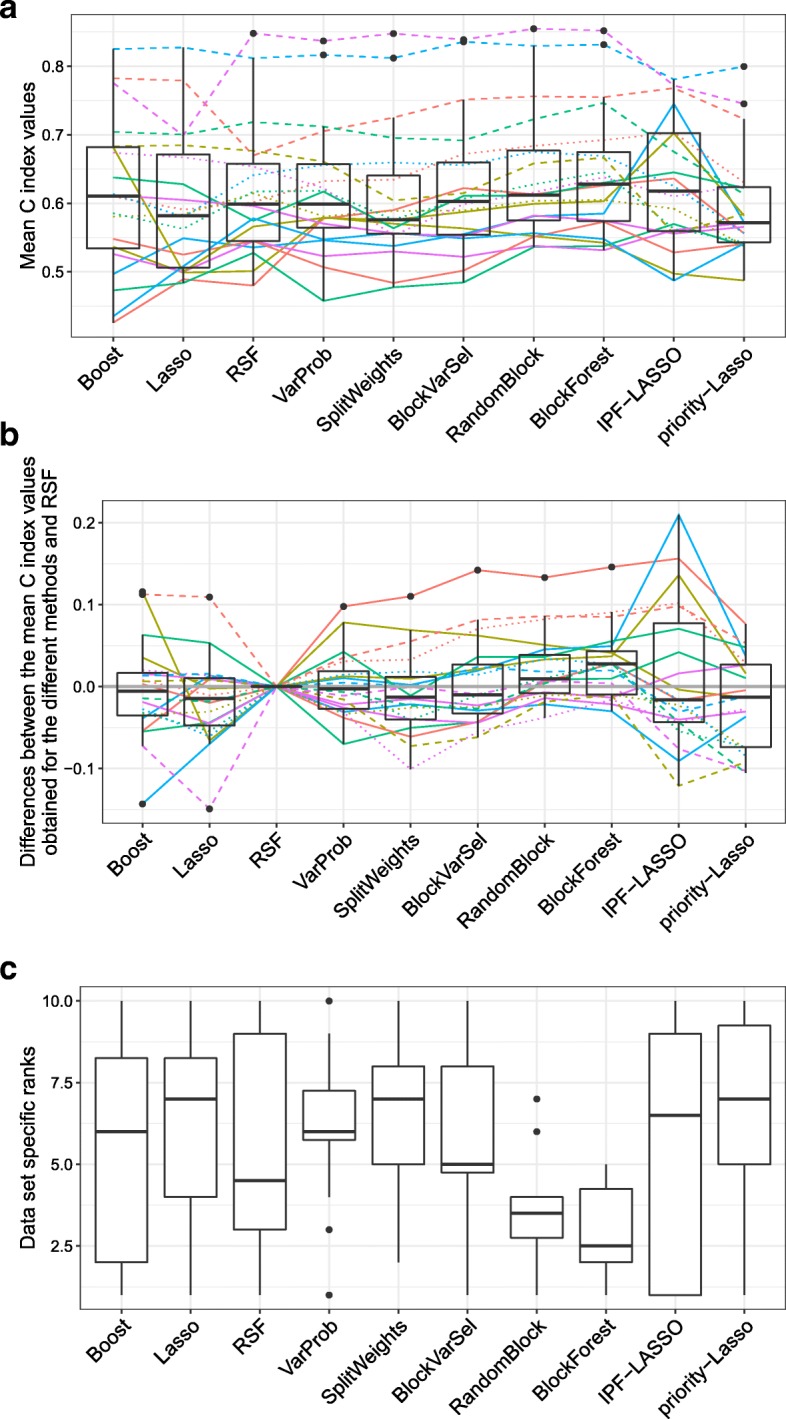
Multi-omics data: Performances of the ten considered methods. **a** Mean C index values for each of the 20 data sets and each of the ten methods considered. **b** Differences between the mean C that obtained using RSF. **c** Data set specific ranks of each method among the other methods in terms of the mean cross-validated C index values. The colored lines in **a** and **b** connect the results obtained for the same data sets

Figure 1 shows the results of the multi-omics comparison study. Figure 1a shows boxplots of the mean cross-validated C index values obtained for all methods and data sets, Fig. 1b shows the differences between the mean cross-validated C index values obtained for the different methods and those obtained using RSF, and Fig. 1c shows boxplots of ranks of the methods calculated using the mean cross-validated C index values of the data sets. On average, BlockForest and RandomBlock performed best among all methods. Specifically, BlockForest achieved the highest median C index across the data sets and a median rank of 2.5. The mean ranks of the methods are as follows (from best to worst): 2.95 (BlockForest), 3.55 (RandomBlock), 5.45 (RSF), 5.45 (IPF-LASSO), 5.6 (Boost), 5.85 (BlockVarSel), 6.1 (VarProb), 6.45 (Lasso), 6.55 (SplitWeights), 7.05 (priority-Lasso). The ranks of IPF-LASSO are very variable across data sets. For some data sets it performed best, but mediocre to bad for the majority of data sets. By contrast, priority-Lasso performed worst in this comparison, slightly worse than the original Lasso. RSF performed best among the methods that do not take the block structure into account, followed by boosting and Lasso. In Additional file 1: Figures S1 and S2 the C index values obtained for the individual repetitions of the cross-validation are shown separately for each data set. Note that, for three of the 20 data sets, only four of the five considered blocks were available. Because the results might be systematically different for these three data sets, as a sensitivity analysis we temporarily excluded these three data sets and re-produced Fig. 1. The results are shown in Additional file 1: Figure S3. While the results are not drastically different under the exclusion of these three data sets, we observe that the improvements of BlockForest and RandomBlock over RSF became slightly stronger.

**Fig. 2:**
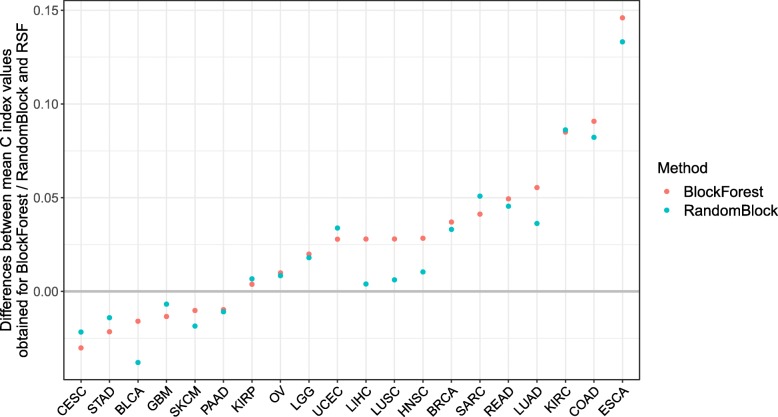
Multi-omics data: Performances of BlockForest and RandomBlock relative to that of RSF. Differences between the mean C index values obtained using BlockForest / RandomBlock and that obtained using RSF, ordered by difference between the values obtained for BlockForest and RSF

In Fig. 2, for each data set, the differences between the data set specific mean C index values obtained using BlockForest and RSF and the differences between the data set specific mean C index values obtained using RandomBlock and RSF are shown. Both BlockForest and RSF were better than RSF for 14 of the 20 data sets (70%), where the degrees of these improvements differ quite strongly across the data sets. Both variants outperformed RSF by more than 0.05 in terms of absolute difference for four of the data sets.

In Section C of Additional file 1 we analyze the influence of data set characteristics on the performance of BlockForest relative to that of RSF. The improvements of BlockForest over RSF tended to become greater for larger sample sizes and slightly lower in cases in which single blocks dominated the other blocks with respect to their importances for prediction. Moreover, for weaker biological signals the degrees of improvement attained through using BlockForest instead of RSF varied more strongly, that is, for weaker signals there were more often stronger improvements, but also more often merely weak improvements and also (slight) impairments.

We performed statistical testing to investigate whether the improvements over RSF are statistically significant. We performed a one-sided paired Student’s *t*-test for each variant with the null hypothesis of non-inferiority of RSF over the considered variant. In the tests, we treated the data set specific mean C index values as independent observations [23], which was possible because the different data sets feature different, non-overlapping sets of samples. Because the tests therefore used one pair of mean C index values per data set, each of them is based on a sample size of 20. The five *p*-values for the variants were adjusted for multiple testing with the Bonferroni-Holm method. The following adjusted *p*-values were obtained: 1.000 (VarProb), 1.000 (SplitWeights), 1.000 (BlockVarSel), 0.056 (RandomBlock), 0.027 (BlockForest). Thus, BlockForest performed significantly better than RSF, while RandomBlock showed merely a weakly significant improvement over RSF (adjusted *p*-value larger than 0.05, but smaller than 0.10). The same conclusions were obtained for the tests when excluding the three data sets for which the measurements of only four of the five blocks were available (results not shown).

In Section D of Additional file 1 we provide in-depth analyses of the optimized values of the tuning parameters associated with the different variants for each data set. In the following we will merely present some important conclusions obtained in these analyses, for details see Additional file 1. The optimized block selection probabilities *b*_1_,…,*b*_*M*_ associated with RandomBlock can give indications of the relative importances of the different blocks for prediction, see “Methods” section. We obtained the following mean block selection probabilities across the data sets (sorted from highest to lowest): 0.43 (mutation), 0.29 (RNA), 0.12 (clinical), 0.11 (CNV), 0.07 (miRNA). Thus, the mutation block and the RNA block seem to be by far the most important blocks. Moreover, we found the correlations between the *b*_*m*_ values (averaged per data set) obtained for the mutation block and that obtained for the RNA block to be strongly negative. This suggests that there is a strong overlap in the information contained in these two blocks. The correlation between the *b*_*m*_ values of the clinical block and that of the mutation block was negative, but that between the *b*_*m*_ values of the clinical block and that of the RNA block was very weak and positive. This suggests that the additional predictive value of the mutation block to the clinical block might in general be smaller than that of the RNA block to the clinical block. A strong additional predictive value of the RNA block over the clinical block would make it particularly effective to exploit the predictive information contained in clinical covariates in situations in which such variables are available in addition to RNA measurements.

We excluded the data set PRAD a posteriori from the results. The survival times in this data set were censored for more than 98% of the patients, leaving merely seven observed events as opposed to 418 censored events. This resulted in extremely unstable performance estimates, since the C index cannot compare pairs of observations for which the shorter time is censored. Note, however, that it is a very delicate issue to exclude a data set from a study after having observed the results for this data set. Doing so offers potential for mechanisms related to fishing for significance [23, 24]. We obtained the following mean cross-validated C index values for the data set PRAD (sorted from largest to smallest): 0.584 (RSF) 0.545 (BlockVarSel) 0.522 (BlockForest) 0.504 (SplitWeights) 0.502 (RandomBlock) 0.500 (Boost) 0.467 (VarProb) 0.39 (priority-Lasso) 0.377 (IPF-LASSO). Lasso is not listed in the mean C index values for this data set because all 25 iterations of the five times repeated 5-fold cross-validation terminated with an error message. Also, in the cases of boosting, IPF-LASSO, and priority-Lasso, there were iterations that resulted in errors for this data set. Note that the variant BlockForest that performed best overall, performed worst in comparison to RSF for this data set among all data sets. We suppose this bad result obtained with BlockForest to be related to overfitting with respect to the optimized tuning parameter values. While Random Forest is quite robust with respect to the choice of *mtry* [25], this is likely not the case for the variants with respect to their block-specific tuning parameter values. As described above, the C index values obtained for this data set are very variable due to the small number of observed survival times, which is why the optimized tuning parameter values can be expected to be very unreliable for this data set. Therefore, the optimized tuning parameter values may be far from the values that are actually optimal for these parameters, which could explain the bad prediction performance.

### Clinical covariates plus RNA measurements

**Fig. 3:**
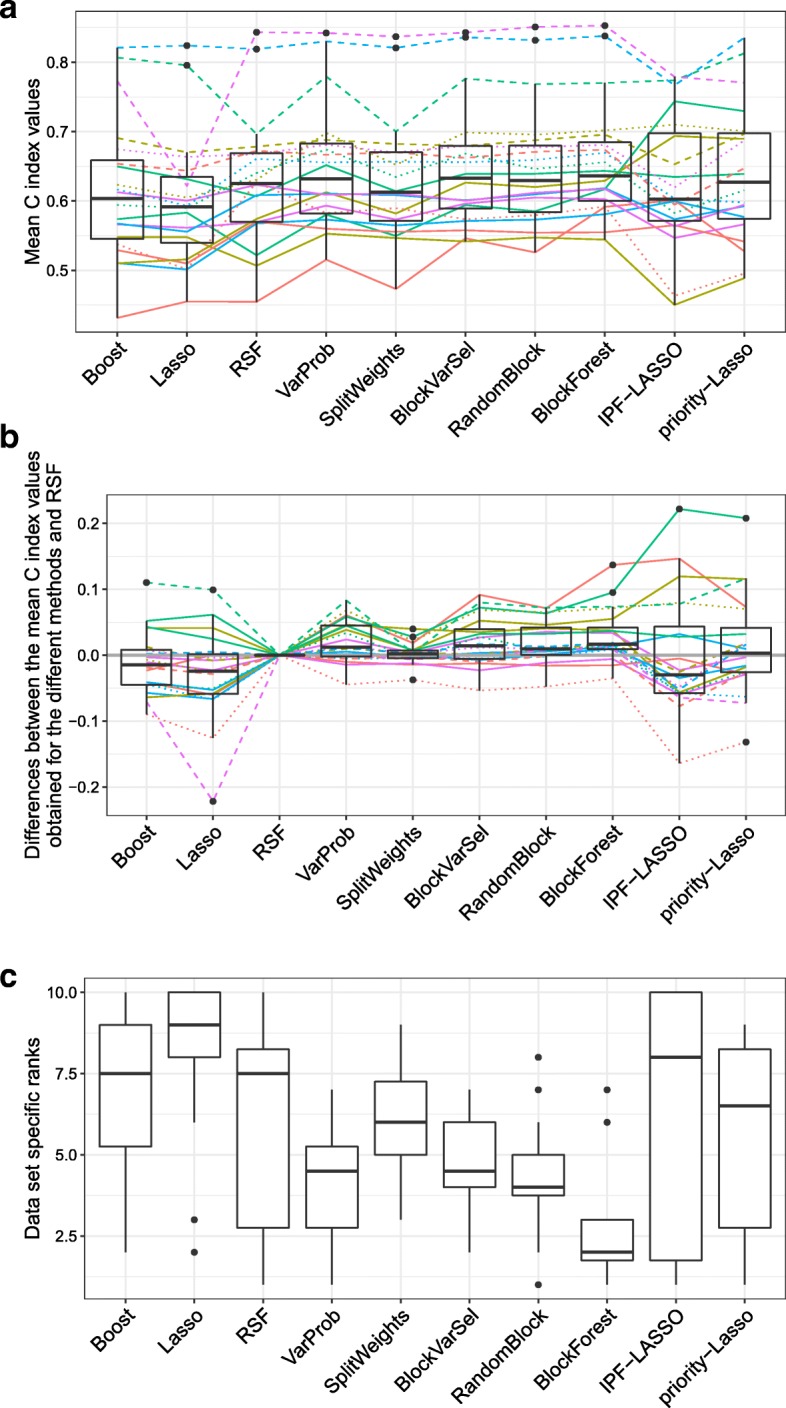
Clinical covariates plus RNA measurements: Performances of the ten considered methods. **a** Mean C index values for each of the 20 data sets and each of the ten methods considered. **b** Differences between the mean C index values obtained using the different methods and that obtained using RSF. **c** Data set specific ranks of each method among the other methods in terms of the mean cross-validated C index values. The colored lines in **a** and **b** connect the results obtained for the same data sets

Figure 3 shows the results obtained for the analysis in which we used only the clinical block and the RNA block. The results are presented in the same form as in Fig. 1. Again, BlockForest performed best, achieving the highest average C index value and a median rank of two.

**Fig. 4:**
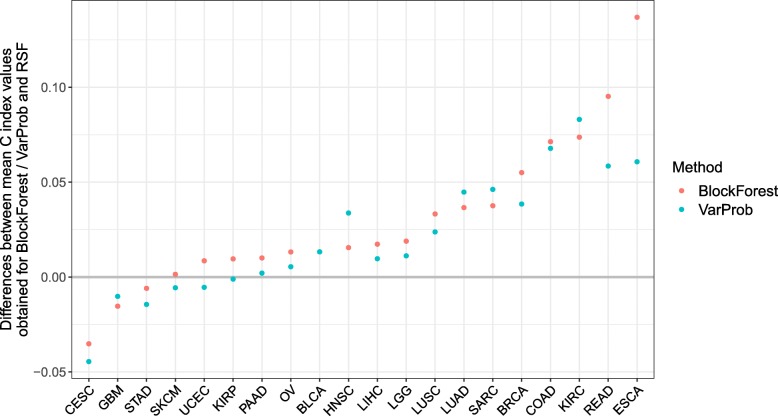
Clinical covariates plus RNA measurements: Performances of BlockForest and VarProb relative to that of RSF. Differences between the mean C index values obtained using BlockForest / VarProb and that obtained using RSF, ordered by difference between the values obtained for BlockForest and RSF

Figure 4 shows the differences between the data set specific mean C index values obtained using BlockForest and RSF and the differences between the data set specific mean C index values obtained using VarProb and RSF. Note that here we show the results obtained for VarProb, as opposed to those obtained with RandomBlock as in Fig. 2, because here VarProb was the second best performing method in terms of the mean ranks of the methods (see further down). BlockForest performed better than RSF for 17 of the 20 data sets (85%), where for five of these data sets the improvement of BlockForest over RSF was greater than 0.05 in terms of absolute difference, while there was only a mild improvement for other data sets. VarProb showed an improvement over RSF for 14 of the 20 data sets (70%). The distributions of the ranks of the methods (Fig. 3c) reveal that BlockForest sets itself apart more strongly from RandomBlock than in the analysis of the multi-omics data. Moreover, excluding SplitWeights, now also the other variants outperform RSF. The performance of SplitWeights is very similar to that of RSF (Fig. 3b) for the great majority of data sets. The reason for this is probably that with SplitWeights as with RSF the clinical covariates are selected very infrequently, which is why the obtained predictions do not differ strongly between these two methods if there is only a clinical block plus a single omics block. As in the multi-omics case, the ranks of IPF-LASSO are very variable, where this method now performs worse than the variants and RSF. However, in contrast to the multi-omics case, priority-Lasso outperforms IPF-LASSO here, but performs worse than most of the variants. Regarding the methods that do not take the block structure into account, again RSF takes the first place, boosting the second place and Lasso the third place, where the latter performs the worst out of all methods here. We obtain the following mean ranks for the methods (from best to worst): 2.65 (BlockForest), 4.10 (VarProb), 4.35 (RandomBlock), 4.70 (BlockVarSel), 5.40 (priority-Lasso), 6.05 (RSF), 6.20 (SplitWeights), 6.65 (IPF-LASSO), 6.75 (Boost), 8.15 (Lasso). As already indicated above, VarProb worked much better here than for the multi-omics data. However, since BlockForest still performed considerably better than VarProb, the latter cannot be recommended for the case of having a clinical block plus an omics block available (nor for the multi-omics case). An explanation for why VarProb performed better here than in the multi-omics case, could be that the optimized variable selection probabilities for the clinical block were considerably larger than in the multi-omics case (see Additional file 1: Figures S5 and S6 and Additional file 1: Figures S18 and S19, respectively). As will be seen in “Outlook: Prioritizing the clinical covariates by including them mandatorily in the split point selection” section, artificially prioritizing the clinical block can improve the prediction performance of the variants. The fact that the optimized variable selection probabilities for the clinical block are larger here can probably be explained by the fact that the larger the number of blocks, the smaller the optimized variable selection probabilities of the individual blocks will be, since the variable selection probabilities add up to one. The C index values obtained for the individual repetitions of the cross-validation separately for each data set are shown in Additional file 1: Figures S15 and S16.

In the same manner as for the multi-omics data, we performed *t*-tests to test for superiority of each of the variants over RSF, again adjusting for multiple testing using Bonferroni-Holm. We obtained the following adjusted *p*-values: 0.019 (VarProb), 0.238 (SplitWeights), 0.026 (BlockVarSel), 0.019 (RandomBlock), 0.01 (BlockForest). Thus, there is a significant improvement over RSF for all of the variants except for SplitWeights.

As for the analysis of the multi-omics data, we investigated the influences of different data set characteristics on the performance of BlockForest relative to that of RSF. There was no clear relation between the sample sizes and the improvements of BlockForest over RSF, which could be related to the fact that when considering only two blocks instead of five (or four), less tuning parameter values have to be optimized. Moreover, the larger the *b*_*m*_ value of the clinical block optimized using RandomBlock was, the greater the improvement of BlockForest over RSF tended to be. This suggests that BlockForest performs particularly strong in situations in which there are highly predictive clinical covariates. Lastly, the improvements of BlockForest over RSF tended to be greater for weaker biological signals. See Section F of Additional file 1 for details.

Analogous to the multi-omics data case, in Section G of Additional file 1 we provide detailed analyses of the optimized tuning parameter values associated with the variants for each data set. The mean optimized block selection probabilities associated with RandomBlock across data sets were as follows: 0.24 (clinical), 0.76 (RNA). In the median, the block selection probability of the RNA block was 3.7 higher than that of the clinical block. This can be interpreted as that the RNA block was in the median 3.7 times as important for prediction as the clinical block for this collection of data sets when considering only the clinical block and the RNA block. For further details, see Additional file 1.

Above we made the presumption that the additional predictive value of the mutation block to the clinical block might be smaller than that of the RNA block to the clinical block. In order to investigate whether the prediction performance is actually lower when using the mutation block instead of the RNA block, we re-performed the analysis presented in this subsection using the clinical block in combination with the mutation block, instead of in combination with the RNA block. For each of the ten included methods, the mean data set specific C index values were smaller than in the case of using the RNA block, where this was statistically significant for four of the ten methods after adjustment for multiple testing. Only the BlockForest variant significantly outperformed RSF (adjusted *p*-value: 0.003). BlockForest and IPF-LASSO performed best out of all methods here, where BlockForest featured the lowest mean rank. See Section H of Additional file 1 for more details.

### Outlook: Prioritizing the clinical covariates by including them mandatorily in the split point selection

As noted above and also seen in the results of the analyses presented in this paper, the clinical covariates often carry much predictive information. In such cases, it might be worthwhile artificially prioritizing these covariates in an effort to improve the predictions.

For this reason, for each of the five random forest variants for multi-omics data presented in this paper, we considered a variation that, for each split, includes all clinical covariates in the sets of variables tried out for finding the optimal split point. Apart from the latter the variations hardly differ from the original variants; see Section I of Additional file 1 for detailed descriptions of these variations. We extended each of the three settings of the comparison study considered in this paper (i.e., the multi-omics case and the two cases using clinical covariates plus RNA / mutation measurements) by the inclusion of these variations.

In Section J of Additional file 1 we show the results of this analysis and discuss them in detail. In almost all cases the variants benefited from including the clinical covariates mandatorily. In the multi-omics case the best performing method out of the now 15 methods was the variation of RandomBlock that includes the clinical covariates mandatorily, where the corresponding variation of BlockForest performed second best. In the two cases that include the clinical block plus the RNA block or the mutation block, respectively, the variation of BlockForest performed best overall, where in the case of using the mutation block, the improvement over the original BlockForest variant was very limited.

Even though in our comparison study considering the clinical covariates mandatorily in the split point selection improved the predictions in most cases, we do not recommend this strategy as a default choice. This is because, whether or not we can expect improvements by including the clinical covariates mandatorily, depends crucially on the level of predictive information contained in the clinical covariates. In situations in which the clinical covariates contain less predictive information than those in the data sets considered in the comparison study, artificially prioritizing the clinical covariates might lead to worse prediction results.

## Discussion

The information overlap between the blocks that manifests itself in negative correlations between the *b*_*m*_ values (associated with RandomBlock) obtained for different blocks, might help to explain why the two methods, block forest aka BlockForest and RandomBlock performed best in our comparison study. With both of these methods the block choice is randomized for each split. Breiman shows in the original random forest paper [19] that the prediction performance of random forests benefits both from strongly differing predictions of the individual trees and from a strong prediction performance of the individual tree predictors. For both BlockForest and RandomBlock, the trees can be expected to differ to a stronger degree than those obtained with the other variants. This is because the succession of the blocks used for splitting in the trees is random in the case of these two methods, completely random for RandomBlock and partly random for BlockForest. The fact that the trees differ more strongly when randomizing the block choice leads to more strongly differing predictions of the individual trees. Moreover, the fact that, by randomizing the block choice, not all blocks are considered in each individual split can be expected to have only a limited negative effect on the prediction performance of the individual trees. The latter is due to the fact that the predictive information contained in the different blocks overlaps. To conclude, randomizing the block choice should make the tree predictions more different and strong at the same time, leading to a strong prediction performance of the forest, as shown in [19].

The fact that four of the five variants delivered better results than RSF for the case of using clinical covariates plus RNA measurements indicates that it is crucial to treat the clinical covariates differently than the omics variables. As already mentioned in “Background” section, clinical covariates often have high prognostic relevance. Due to their small number in comparison to the omics variables, these variables are too infrequently used for splitting in a standard random (survival) forest, which is why their prognostic value is not exploited in it. Of course, the latter is also true for any other prediction method for high-dimensional variable data applied to clinical covariates plus a certain type of omics variables that does not differentiate between clinical and omics variables. By extending the comparison study by variations of the variants that include the clinical covariates mandatorily, we saw that it can even be effective to artificially prioritize the clinical covariates. Even though we cannot recommend this strategy as a default choice (see the previous subsection for details), it should in each case be tried out in order to assess, whether it can improve the prediction results in the specific application. When including the clinical covariates mandatorily, for multi-omics data (as opposed to clinical covariates in combination with a single omics data type), RandomBlock may be preferable over BlockForest. A problem with the base version of RandomBlock that includes the clinical block in the collection of blocks to sample from might be that the clinical covariates are not considered frequently enough, because only a single block is randomly drawn for each split. Note that when trying out several approaches and choosing the one with the optimal cross-validated prediction performance estimate, this estimate is optimistically biased [26, 27]. A simple solution to this issue would be to report, as a conservative performance estimate, the worst prediction performance estimate out of those obtained. Alternatively, nested cross-validation can be used, that is, repeating the model selection within an outer cross-validation loop. For more sophisticated approaches, see [26].

While it can be very effective to allow for prioritization of the clinical block over high-dimensional omics blocks, less benefit can be expected from imposing different prioritizations between omics blocks of similar size. This is because, if the variables from two omics blocks of similar size are assigned the same prioritizations, the variables from the more informative block will still be used more often for splitting than the variables from the less informative block. These considerations also affect the interpretation of the results obtained for the analysis of the multi-omics data. As described in “Methods” section, in the analysis of the multi-omics data, for blocks with more than 2500 variables, we pre-selected the 2500 variables with smallest *p*-values from univariate Cox regressions. By doing so, three of the four omics blocks had 2500 variables after pre-selection. The only block with less than 2500 variables was the miRNA block, which was, however, non-informative for almost all data sets. Thus, all influential omics blocks had the same numbers of variables (after pre-selection) in the analysis of the multi-omics data. For this reason, the different prioritizations of the omics blocks might not have been that crucial compared to situations in which the informative omics blocks feature highly differing numbers of variables. In such situations, the expected performance gain by using BlockForest instead of standard RSF might be larger. For example, consider a scenario involving two blocks A and B, where block A features 50 variables and block B 10000 variables. Suppose, moreover, that block A is informative and block B is uninformative. In this situation, it is quite possible that RSF uses block B erroneously more often than block A simply because block B contains more variables. This is because, since observed data is subject to random fluctuations, when continually searching for suitable splits in uninformative variables, eventually a split will be found that divides the observations well with respect to the target variable. However, since the selected variable is uninformative, the corresponding split will not be useful when used in prediction, that is, applied to observations external to the training data.

Comparing the mean C index values obtained when using all available blocks (Fig. 1) and when using clinical covariates plus RNA measurements (Fig. 3) an interesting observation can be made: The mean C index values are in most cases higher when using clinical covariates plus RNA measurements only than when using all available blocks. This is true for both standard RSF as well as for the variants. When using all available blocks there is more predictive information available than when using only a single block, which is why it appears contradictory at first that the prediction performance is worse when considering all available blocks for prediction. However, we can make three observations that help to explain, why the prediction rules obtained using the combination of the clinical block and the RNA block performed that strongly. First, the results obtained in Section D of Additional file 1 suggested that the predictive information contained in the two most important blocks, the mutation block and the RNA block, is highly overlapping. The evidence that supported this assumption was that the method RandomBlock in the great majority of cases attributed a very large value of the selection probability to one of these two blocks and a very small value to the respective other block. The strong overlap in information between the mutation block and the RNA block has the effect that there tends to be limited gain in prediction accuracy by including the mutation block in addition to the RNA block even though the mutation block does contain much predictive information. Second, the RandomBlock algorithm attributed in most cases small selection probabilities to the remaining blocks, which suggests that these most often provided limited additional predictive value over the mutation block and/or the RNA block. Third, the results obtained with all blocks also suggested that the additional predictive value of the RNA block over the clinical block is higher than that of the mutation block over the clinical block. The prediction performances of the methods were also better when using the clinical block in combination with the RNA block then they were when using the clinical block in combination with the mutation block (significantly better for four of the ten methods). For these three reasons, in practice it should in many cases be sufficient to consider only the clinical information plus the RNA measurements. Such prediction rules are quite convenient to handle. This is because it is merely required that the necessary clinical information and the RNA measurements are available instead of measurements of a multitude of different omics data types, both, for the purpose of constructing the prediction rule and, even more importantly, for the purpose of applying it.

Because the variants feature a tuning parameter for each block, the numbers of involved tuning parameters are relatively large. As discussed already in “Results” section in the context of describing the results obtained for the data set PRAD, this might be associated with an overfitting mechanism: the optimized tuning parameter values might be overly well adjusted to the given data set. The fact that we observed a trend of greater improvements of BlockForest over RSF for larger data sets is indicative of that. Nevertheless, as the results of the comparison study demonstrate, this overoptimism, in case it exists, is weak enough to warrant that BlockForest still outperforms RSF (to varying degrees) for the majority of data sets.

For each of the variants, we fixed the number of variables to be randomly sampled at each split. The reason for this choice was to avoid the need of having to optimize another tuning parameter. Nevertheless, it might be worthwhile to consider optimizing this number or rather the proportion of variables from all variables to be sampled (where in the cases of BlockVarSel, RandomBlock, and BlockForest sampling from each block is performed). By contrast, in the case of RSF we did optimize the number *mtry* of variables sampled for each split, which likely explains, why some of the variants performed even worse than RSF in the comparison study.

As noted in “Background” section, the considered variants are applicable for any types of outcomes (e.g., categorical or continuous outcomes), for which there exists a random forest variant. Our comparison studies were limited to survival outcomes. However, the discussed advantage of block forest (and RandomBlock), that it tackles the issue of overlapping predictive information between the blocks holds also for other types of outcomes. Nevertheless, to assess the prediction performance of block forest for other types of outcomes, analogous comparison studies like the one performed in this paper would be necessary.

## Conclusions

Using a collection of 20 real multi-omics data sets we compared the prediction performance of five different candidate random (survival) forest variants for multi-omics covariate data to that of competitors, in particular to that of random survival forest, which we considered as a reference method. We also considered the common situation of having only the clinical block plus a single type of omics data (in our case RNA or mutation measurements, respectively) available. Both, for the latter case and for the multi-omics data the variant “BlockForest” or “block forest” performed best and significantly better than the reference method random survival forest. Therefore, we recommend using block forest in applications to exploit effectively the predictive information contained in combinations of clinical data and one or several types of omics data. The other random forest variants can be consulted for academic purposes, for example, in the context of further methodological developments. Depending on the level of information contained in the clinical covariates, considering these covariates mandatorily in the split point selection of block forest (or RandomBlock) may improve the prediction results.

## Methods

### Multi-omics data format

A multi-omics data set with *n* observations consists of *M* covariate matrices ***X***_1_,…,***X***_*M*_ and an outcome vector ***y***_1_,…,***y***_*n*_. The *m*th matrix ***X***_*m*_ of dimension *n*×*p*_*m*_ contains, for each observation, the measurements of the *p*_*m*_ variables in the *m*th block. The outcome values ***y***_*i*_,*i*=1,…,*n*, are most often scalars, for example ***y***_*i*_∈{0,1} for binary outcomes or $\boldsymbol {y}_{i} \in \mathbb R$ for metric outcomes. They may, however, also take the form of vectors, for example ***y***_*i*_={*y*_*i*,1_,*y*_*i*,2_} with $y_{i,1} \in \mathbb R_{> 0}$ and *y*_*i*,2_∈{0,1} for survival outcomes, where *y*_*i*,1_ denotes the survival/censoring time of the *i*th observation and *y*_*i*,2_ its value of the censoring indicator. The covariate matrices can be concatenated in order to form a single covariate matrix ***X***:=[***X***_1_,…,***X***_*M*_] with $p = \sum \nolimits _{m = 1}^{M} p_{m}$ columns.

### Split selection procedures for random forests tailored to multi-omics data

As described in “Background” section, the five considered random forest variants for multi-omics data differ merely with respect to split point selection. In the following, we first recall the standard random forest algorithm and the split point selection performed by this algorithm. Subsequently, we describe the split point selection procedures of the considered variants and briefly discuss the motivations behind each of these approaches. Each of these procedures involves block-specific parameters, which are chosen automatically using an optimization procedure described in “Optimization of tuning parameters” section.

#### Standard random forest

A random forest prediction rule is a collection of decision trees, where each of the latter is constructed using a subsample or bootstrap sample of a training data set. Each of the tree decision rules performs a series of binary decisions, where each decision is obtained using a threshold, called “split point”, in the values of one of the covariate variables available in the training data set. The decision trees are constructed by recursively dividing the available samples in two subgroups using the split points which are obtained during the construction of the trees. The nested subgroups are denoted as nodes.

In standard random forest a split point for a node in a tree is obtained as follows (assuming only continuous variables for ease of presentation). First, a number *mtry* of variables is randomly sampled from all variables. Second, an optimal split point in the ordered values of the sampled variables is obtained in the following way: 1) Divide the node once according to each possible split point in each variable and for every division obtained, calculate the value of a certain quantitative split criterion; 2) Use that split point among all split points considered in 1) that was best according to the split point criterion. The block structure of multi-omics data is obviously not taken into account in this split point selection procedure. Note that the split point selection is performed slightly differently for nominal variables, for details see Hastie et al. [28] (chapter 9.2.4).

#### VarProb: Block-specific variable selection probabilities

The procedure of drawing from all variables without taking the block structure into account, as performed in the split point selection of standard random forest, has two main issues. First, with this procedure, blocks that involve fewer variables are underrepresented in the drawn variables. In many cases, it would be preferable if a variable from a block with fewer variables would be drawn more often than a variable from a block with many variables. This is because the predictive information in blocks with fewer variables is often more dense. For example, it is well known that, while the block containing clinical information does usually contain a very small number of variables in comparison to the omics blocks, a large fraction of this small number of variables can be expected to be highly predictive. Beyond this specific example, there needs to be a negative correlation between block size and information density. If, by contrast, this was not the case, this would either mean that the amount of predictive information of a block has a positive dependency on its size, or that informative variables are weaker in larger blocks.

The second, related issue is that even for equal block sizes, the procedure of drawing variables from different blocks with the same frequencies does not take into account that the blocks differ with respect to their levels of predictive information contained. It could be an advantage if variables from blocks with much predictive information would be drawn more often than variables from blocks with little predictive information.

Both of the above issues are addressed by using block-specific variable selection probabilities in the variant VarProb presented in this subsection. This has the effect that the sampling probabilities of variables from some blocks are higher than those of variables from other blocks. With the split selection procedure of VarProb, a variable in block *m* has sampling probability *v*_*m*_, where we ensure that the sampling probabilities of all variables add up to one, that is, we impose the following restriction: $\sum \nolimits _{m=1}^{M} p_{m} v_{m} = 1$. Note that the *v*_1_,…,*v*_*M*_ are the block-specific parameters of VarProb, which are, as noted above, optimized automatically (see “Optimization of tuning parameters” section). In order to fix the number of variables to be drawn for each split to *mtry* we proceed as follows: Each sampled variable is drawn one after another. If, in this process, a variable is drawn that is already in the set of drawn variables, the drawing is repeated until a variable is drawn that is not yet in the set of drawn variables. This process is repeated until *mtry* variables have been drawn. Finally, the best split point is determined in the *mtry* drawn variables as in standard random forest. The value of *mtry* is set to $\sum \nolimits _{m=1}^{M} \sqrt {p_{m}}$.

#### SplitWeights: Block-specific weights of split criterion values

Using block-specific variable selection probabilities in VarProb has the effect of prioritizing some blocks over others. Another way to accomplish the latter is to use block-specific weights *w*_*m*_ (*m*=1,…,*M*) for the split criterion values, where *w*_1_,…,*w*_*m*_>0 and max{*w*_1_,…,*w*_*M*_}=1 for reasons of identifiability. With this procedure, variables from blocks with high *w*_*m*_ values are prioritized over variables from blocks with low *w*_*m*_ values. First, a number of $mtry = \sum \nolimits _{m=1}^{M} \sqrt {p_{m}}$ variables are drawn from all variables, as in conventional random forest. Second, the split criterion values associated with all split points in the sampled variables are calculated and these values are weighted using the block-specific weights *w*_*m*_. Third, the split point is chosen that features the highest weighted split criterion value.

As in the case of VarProb, with SplitWeights variables from different blocks are prioritized differently by the procedure. Because the predictive information contained in blocks with large numbers of variables tends to be less dense, more variables need to be sampled from these blocks in order to increase the likelihood of obtaining reasonably informative variables. However, when sampling different numbers of variables from different blocks in this way, the best variable from a large block tends to separate the observations better than the best variable from a small block just by chance, even though the best variable from the small block is often actually more suitable. This problem occurs for the same reason as the bias towards variables with many possible split points described by Strobl et al. [29]. The tendency of selecting suboptimal variables from large blocks is avoided when the split criterion values of these blocks are attributed smaller weights *w*_*m*_.

#### BlockVarSel: Separate sampling of variables from each block and block-specific weights of split criterion values

A disadvantage of SplitWeights is that the smaller the number of variables in a block is, the smaller is the probability that this block is present in the variables sampled for a split. For example, a block containing clinical information most often contains only few variables, which is why there will be no clinical covariates among the sampled variables for most of the splits. Clinical covariates, however, often contain much predictive information and interact with omics variables, which is why it is detrimental if they are considered only infrequently.

In order to avoid the above shortcoming of SplitWeights, with the split selection procedure BlockVarSel presented in this subsection, for each split, we sample fixed numbers of variables from each block separately. More precisely, for *m*=1,…,*M* we sample $\sqrt {p_{m}}$ variables from block *m*. Subsequently, split point selection is performed as with SplitWeights, that is, using weighted split criterion values with block-specific weights *w*_*m*_. Note that with this approach, as with SplitWeights, larger numbers of variables are sampled from larger blocks.

#### RandomBlock: Random block selection

A key reason for the strong prediction performance of standard random forest is that through considering only a random subset of *mtry* variables for each split, the resulting trees are very dissimilar from each other. Roughly formulated, each tree captures different aspects of the complex dependency structure between the outcome and the variables. In the context of multi-omics data, we are particularly interested in rendering aspects of the interplay of the different blocks with respect to their influence on the outcome. Therefore, it seems beneficial to make the trees not only very dissimilar with respect to the involved variables in general, but also in particular with respect to the involvements of the different blocks.

On the basis of this idea, with the split selection procedure RandomBlock, first, one of the blocks is selected randomly and, second, a subset of variables from the selected block is sampled. The sampled subset of variables from the selected block is subsequently considered for splitting and split point selection is performed among the sampled subset of variables as in standard random forest. In order to account for the different levels of predictive information associated with the different blocks, block-specific selection probabilities *b*_*m*_ are used, where $\sum \nolimits _{m=1}^{M} b_{m} = 1$. After block selection, $\sqrt {p_{m}}$ variables are sampled from the selected block.

The fact that the succession of the blocks used for splitting is random within the trees makes the resulting forest put high emphasis on rendering interactions between variables across blocks. A further distinctive property of this split selection procedure is that there are no comparisons of split points made across blocks, since only variables from a single block are considered for each split. This avoids issues with differences in dimensionality between the blocks that have to be dealt with in the other approaches. Moreover, it avoids problems caused by correlations between variables from different blocks, where these correlations are associated with the fact that the predictive information contained in different blocks is overlapping. A further advantage of RandomBlock is that the *b*_*m*_ values can give indications of the relative importances of the different blocks for prediction. The higher the *b*_*m*_ value of a block is, the higher its importance compared to the other blocks will tend to be. However, small *b*_*m*_ values must be interpreted with great care,because important blocks can be attributed small *b*_*m*_ values if these blocks share much predictive information with other important blocks. For details on this mechanism see Section D of Additional file 1 referenced in “Multi-omics data” subsection of “Results” section. The *b*_*m*_ values may also be used to screen out blocks that are not relevant for prediction given the remaining blocks. This can be done by excluding blocks that feature very small *b*_*m*_ values.

#### BlockForest: Block sampling with separate sampling of variables from each block and block-specific weights of split criterion values

A major advantage of the procedure RandomBlock that is described in the previous subsection is that it adds the additional randomization component “block selection” to the standard random forest algorithm. For the procedure described in the current subsection we extended the procedure BlockVarSel by a block selection randomization procedure. The new procedure is performed as follows: 1) Obtain a subset of all *M* blocks by selecting each block with probability 0.5, that is, all blocks are selected with probability 0.5^*M*^ and no block at all with the same probability; 2) If no block was selected, repeat 1) until at least one block is selected; 3) Perform the split selection procedure of BlockVarSel using only the blocks selected in 1) or 2), respectively. This new procedure leads to more strongly differing trees than BlockVarSel, because weaker blocks are better taken into account. We use the general term “BlockForest” or “block forest” for this procedure, because it performed best among the studied procedures in our comparison study.

### Optimization of tuning parameters

Each of the split point selection procedures described above contains *M* tuning parameters, where each tuning parameter is associated with one of the blocks. These tuning parameters are variable selection probabilities *v*_1_,…,*v*_*M*_ for VarProb, block weights *w*_1_,…,*w*_*M*_ for SplitWeights, BlockVarSel and BlockForest, as well as block selection probabilities *b*_1_,…,*b*_*M*_ for RandomBlock.

The optimization of the tuning parameter values is performed as follows for each of the five variants:

1. For *it*=1,…,*N*_sets_:
  - Generate a random set $\boldsymbol {{\mathcal {S}}}_{it}$ of *M* tuning parameter values.
  - Construct a forest with *num*.*trees*_pre_ trees using the tuning parameter value set $\boldsymbol {{\mathcal {S}}}_{it}$ and record the out-of-bag prediction error of that forest.
2. Use that tuning parameter set out of $\boldsymbol {{\mathcal {S}}}_{1}, \dots, \boldsymbol {{\mathcal {S}}}_{N_{\operatorname {sets}}}$, for which the corresponding forest delivered the smallest out-of-bag prediction error.

The generation of the random sets of tuning parameter values is described in Section K of Additional file 1. The choice of the prediction error measure depends on the considered outcome. For survival data (see the comparison study presented in “Comparison study” section), we use one minus the value of Harrell’s C index.

Note that while the optimization algorithm presented above is relatively inefficient, it has the important advantage that it is consistent with respect to the tuning parameter value set actually associated with the lowest out-of-bag prediction error. That is, for larger values of *N*_sets_, the optimized tuning parameter set will approximate the optimal tuning parameter value set, that is, the set with lowest possible out-of-bag prediction error, increasingly well. By contrast, the properties of more sophisticated procedures tend to be less clear, which is why such procedures can be prone to result in local optima. In our analyses, we use the values *N*_sets_=300 and *num*.*trees*_pre_=1500, which are also the default values in our R package blockForest. Such large numbers of sets and trees per constructed forest are possible because the package blockForest is a fork of ranger [30], which is a fast C++ random forest implementation.

### Comparison study

#### Data

We used 21 real multi-omics data sets with survival outcome, where each of these data sets contains measurements of patients with a certain cancer type. All data sets were downloaded from the database The Cancer Genome Atlas Project (TCGA) [31]. As described in “Results” section the data set ‘PRAD’ (cf. Table 1) was excluded a posteriori for reasons unrelated to the results obtained with this data set. The following blocks were present among the data sets: clinical information, miRNA data, mutation data, copy number variation measurements, and RNA data. Not every block was available for each data set: For two data sets (‘CESC’ and ‘GBM’) the miRNA block was not available and for one data set (‘READ’) there was no mutation data. While the numbers of variables available for each block do differ between the data sets, they are all in the same order of magnitude across data sets. On average, the following numbers of variables were available for each block: 4.5 (clinical block), 780.3 (miRNA block), 16579.8 (mutation block), 57888.4 (CNV block), 23442.6 (RNA block). Table 1 provides an overview of the data sets. In Additional file 1: Table S1 we provide a more detailed overview of the data sets in which we provide the numbers of variables available for each block in each data set.

**Table 1:** Overview of the data sets used in the comparison study

Name	Cancer type	Sample size	Uncensored observations
BLCA	Bladder Urothelial Carcinoma	310	32%
BRCA	Breast Invasive Carcinoma	863	9%
CESC	Cervical Squamous Cell Carcinoma and Endocervical Adenocarcinoma	206	15%
COAD	Colon Adenocarcinoma	350	22%
ESCA	Esophageal Carcinoma	121	21%
GBM	Glioblastoma Multiforme	154	73%
HNSC	Head and Neck Squamous Cell Carcinoma	411	35%
KIRC	Kidney Renal Clear Cell Carcinoma	322	22%
KIRP	Kidney Renal Papillary Cell Carcinoma	249	10%
LGG	Brain Lower Grade Glioma	454	21%
LIHC	Liver Hepatocellular Carcinoma	298	28%
LUAD	Lung Adenocarcinoma	424	30%
LUSC	Lung Squamous Cell Carcinoma	365	39%
OV	Ovarian Serous Cystadenocarcinoma	261	54%
PAAD	Pancreatic Adenocarcinoma	142	49%
PRAD	Prostate Adenocarcinoma	425	2%
READ	Rectum Adenocarcinoma	138	16%
SARC	Sarcoma	183	16%
SKCM	Skin Cutaneous Melanoma	264	25%
STAD	Stomach Adenocarcinoma	284	27%
UCEC	Uterine Corpus Endometrial Carcinoma	503	13%

The following information is given: Name of the data set, cancer type, sample size and the percentage of observations for which the survival time was uncensored. Note that the TCGA Project ID of each data set is “TCGA-[Name]”, with “[Name]” being the name of the data set (given in the first column)

We used *k* nearest neighbors imputation to impute missing values in the clinical block in data sets with more than two clinical covariates. We used the function knnImputation from the R package DMwR [32]. In cases with only two variables this was not possible because knnImputation is only applicable for a minimum of three variables. Here, we used univariate logistic regression for imputation. Note that by performing the imputation before the cross-validation used for performance estimation, we performed incomplete cross-validation, which, depending on the analysis step performed outside of the cross-validation, can lead to overoptimism in the resulting prediction performance estimates [33]. However, performing incomplete cross-validation with respect to imputation has been found to not affect the performance estimates to any relevant degree [33].

#### Study design

We compared the five variants VarProb, SplitWeights, BlockVarSel, RandomBlock, and BlockForest to RSF as a reference method and to four further methods: two prediction methods for high-dimensional covariate data, model-based gradient boosting [34] and CoxLasso [35], and two prediction methods for multi-omics covariate data, IPF-LASSO and priority-Lasso. Note again that since the data sets considered in the comparison study are survival data sets, the latter five forest variants for multi-omics data are variants of the RSF algorithm.

As noted in “Optimization of tuning parameters” section we set *N*_sets_=300 and *num*.*trees*_pre_=1500 for the forest variants. After optimizing the tuning parameter value sets, we constructed forests with 2000 trees. For RSF we optimized the values of *mtry* using grid search: First, for each $mtry \in \{ \lceil x \sqrt {p} \rceil | x \in \{0.1, 0.25, 0.5, 1, 2\}\}$ we constructed an RSF with 1500 trees and, second, used that of the *mtry* values tried that was associated with the smallest out-of-bag prediction error. Subsequently, we constructed a forest with 2000 trees as in the case of the variants. The optimization of *mtry* was performed, because the performance of random survival forest depends on the value chosen for this parameter. As split criterion we used the log-rank test statistic. Because of the computational expense of log-rank tests, evaluating each possible split point for each considered variable is too expensive in the context of forests applied to high-dimensional data. Therefore, for each variable tried, we merely considered one randomly sampled split point. This split point selection procedure is known as extremely randomized trees [36] for classification and regression trees. In an extensive comparison study, extremely randomized trees have been found to feature similar or even slightly better prediction performance than conventional trees for which each split point is tried out for each considered variable [36].

The number *m*_*stop*_ of boosting steps in model-based gradient boosting and the shrinkage parameter *λ* of CoxLasso were both optimized using cross-validation in each iteration of the (outer) cross-validation used to estimate the C index values (see further down). The IPF-LASSO features a shrinkage parameter for each block. Unless the number of blocks is low, using cross-validation to optimize these shrinkage parameters becomes computationally challenging due to the large number of shrinkage parameter value tuples needing to be considered in the optimization. As a solution to this issue, the two-step IPF-LASSO [37] was developed which we used in the comparison study. In the first step of this approach, for each block a so-called penalty factor is determined. In the second step, a CoxLasso model is fitted to the data, optimizing the shrinkage parameter using cross-validation, where in the penalty term of the optimization problem, the parameters are multiplied by the block-specific penalty factors. The block-specific shrinkage parameters are subsequently determined by multiplying the shrinkage parameter optimized in the second step by the penalty parameters. Roughly speaking, the penalty factors should be inverse proportional to the importances of the blocks, that is, the more important a block becomes, the smaller should be its penalty factor. In the case of the priority-Lasso, we did not have enough background knowledge to use substantial considerations for setting the priority orders of the blocks for the different data sets. For this reason, we chose the priority orders using the values of the penalty factors determined in the first step of the two-step IPF-LASSO: the block with the smallest penalty factor was attributed the highest priority, the block with the second smallest penalty factor the second highest priority and so on. We used the default priority-Lasso version without cross-validated offsets to reduce the computational burden, even though the version with cross-validated offsets delivers slightly better prediction results [11].

We used 5-fold cross-validation repeated five times to measure the performance of each method on each data set. As a performance metric we used Harrell’s C index for which we used the short form C index above. First, we calculated the C index on the left out fold in each iteration of the repeated cross-validation and, second, took the average of these values across the iterations and repetitions of the cross-validation. In the case of the data set PRAD that was excluded from the analysis of the results (see “Multi-omics data” section of “Results” section for details), the application of some methods terminated with errors for several cross-validation iterations. For other data sets, this was the case only for the methods boosting and priority-Lasso. While the application of priority-Lasso terminated prematurely for only few cross-validation iterations, for boosting errors occurred more frequently. In very rare cases the C index was not calculable on the left out fold for an iteration due to lack of comparable pairs of observations. All iterations with missing results were left out when calculating the corresponding averages.

In the case of blocks with more than 2500 variables, we conducted supervised variable selection on the training data sets within cross-validation, selecting the 2500 variables that featured the smallest *p*-values in univariate Cox regression. This was performed both, for computational efficiency and because for ultra high-dimensional omics data types most variables can be assumed to be without effect.

We performed the analysis once using all available blocks for each data set (including the clinical block) and once using only the clinical block and the RNA block. As described in “Background” section, the latter setting of having clinical covariates plus a certain type of omics data available is frequently occurring in practice. For this reason we were interested in comparing the methods also with respect to their performance when applied in this setting. We chose the RNA block as the involved omics block in this analysis, because this omics data type seems to be one of the most commonly found in practice, of those available for the considered data sets.

## Additional files

Additional file 1 Additional contents referenced in the paper. This file contains all Supporting Information referenced in the paper: **A** Multi-omics data: C index values obtained for the individual repetitions of the cross-validation. **B** Multi-omics data: Results obtained for the 17 data sets, for which all five blocks were available. **C** Multi-omics data: Analysis of the influence of data set characteristics on the performance of BlockForest relative to that of RSF. **D** Multi-omics data: Optimized block-specific tuning parameter values associated with the different variants. **E** Clinical covariates plus RNA measurements: C index values obtained for the individual repetitions of the cross-validation. **F** Clinical covariates plus RNA measurements: Analysis of the influence of data set characteristics on the performance of BlockForest relative to that of RSF. **G** Clinical covariates plus RNA measurements: Optimized block-specific tuning parameter values associated with the different variants. **H** Clinical covariates plus mutation measurements: Performances of the ten considered methods. **I** Variations of the variants that prioritize the clinical covariates by including them mandatorily in the split point selection: Description of the variations. **J** Variations of the variants that prioritize the clinical covariates by including them mandatorily in the split point selection: Results. **K** Algorithms used for generating random sets of tuning parameter values in the tuning parameter value optimization. **L** Overview of the data sets used in the comparison study. (PDF 590 kb)

Additional file 2 Electronic Appendix. This folder contains all R Code written to perform the analyses presented in this paper and in Additional file 1 as well as Rda files enabling fast evaluation of the results. (ZIP 26,855 kb)

## Data Availability

The data sets used in the analyses presented in this paper and in Additional file 1 were downloaded from the TCGA database (https://cancergenome.nih.gov/). All R code written to perform and evaluate the analyses, including data pre-processing, is made available in Additional file 2. The pre-processed versions of the data sets as used in the analysis are not included in Additional file 2 due to their large sizes. However, they are available from the corresponding author upon request. The R package blockForest (version 0.2.0) in which all five studied variants are implemented is available from CRAN (https://cran.r-project.org/package=blockForest, GitHub version: https://github.com/bips-hb/blockForest).

## Abbreviations

RSF: Random survival forest
TCGA: The Cancer Genome Atlas Project
