# Supplementary material for: Block Forests: random forests for blocks of clinical and omics covariate data
Source: BMC Bioinformatics. 2019 Jun 27;20:358. doi: 10.1186/s12859-019-2942-y (PMC6598279; doi:10.1186/s12859-019-2942-y)
Supplement: Supplementary file 1 — Additional contents referenced in the paper. This file contains all Supporting Information referenced in the paper: A Multi-omics data: C index values obtained for the individual repetitions of the cross-validation. B Multi-omics data: Results obtained for the 17 data sets, for which all five blocks were available. C Multi-omics data: Analysis of the influence of data set characteristics on the performance of BlockForest relative to that of RSF. D Multi-omics data: Optimized block-specific tuning parameter values associated with the different variants. E Clinical covariates plus RNA measurements: C index values obtained for the individual repetitions of the cross-validation. F Clinical covariates plus RNA measurements: Analysis of the influence of data set characteristics on the performance of BlockForest relative to that of RSF. G Clinical covariates plus RNA measurements: Optimized block-specific tuning parameter values associated with the different variants. H Clinical covariates plus mutation measurements: Performances of the ten considered methods. I Variations of the variants that prioritize the clinical covariates by including them mandatorily in the split point selection: Description of the variations. J Variations of the variants that prioritize the clinical covariates by including them mandatorily in the split point selection: Results. K Algorithms used for generating random sets of tuning parameter values in the tuning parameter value optimization. L Overview of the data sets used in the comparison study. (PDF 590 kb) [file 12859_2019_2942_MOESM1_ESM.pdf]

Supplementary Data to the BMC Bioinformatics article:

Block Forests: random forests for blocks of clinical and omics covariate data

Roman Hornung*,[1], Marvin N. Wright [2,3]

[1] Institute for Medical Information Processing, Biometry and Epidemiology, University of Munich, Marchioninistr. 15, 81377 Munich, Germany

[2] Leibniz Institute for Prevention Research and Epidemiology – BIPS, Achterstr. 30, 28359 Bremen, Germany

[3] Section of Biostatistics, Department of Public Health, University of Copenhagen, Øster Farimagsgade 5, 1014 Copenhagen, Denmark

*To whom correspondence should be addressed: hornung@ibe.med.uni-muenchen.de

A Multi-omics data: C index values obtained for the individual repetitions of the cross-validation

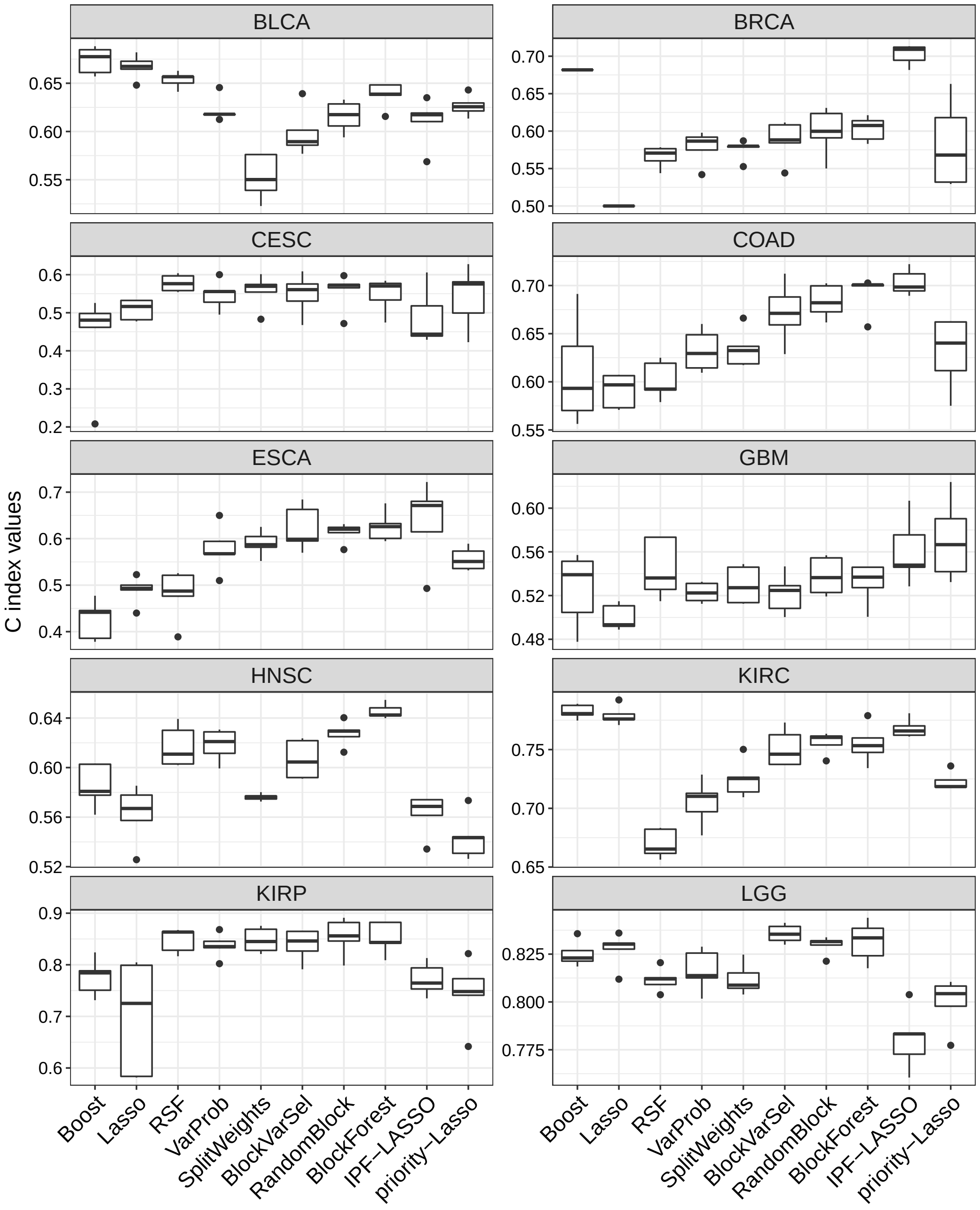

Fig. S1: Multi-omics data: C index values obtained for the individual repetitions of the cross-validation separately for each data set and method – I

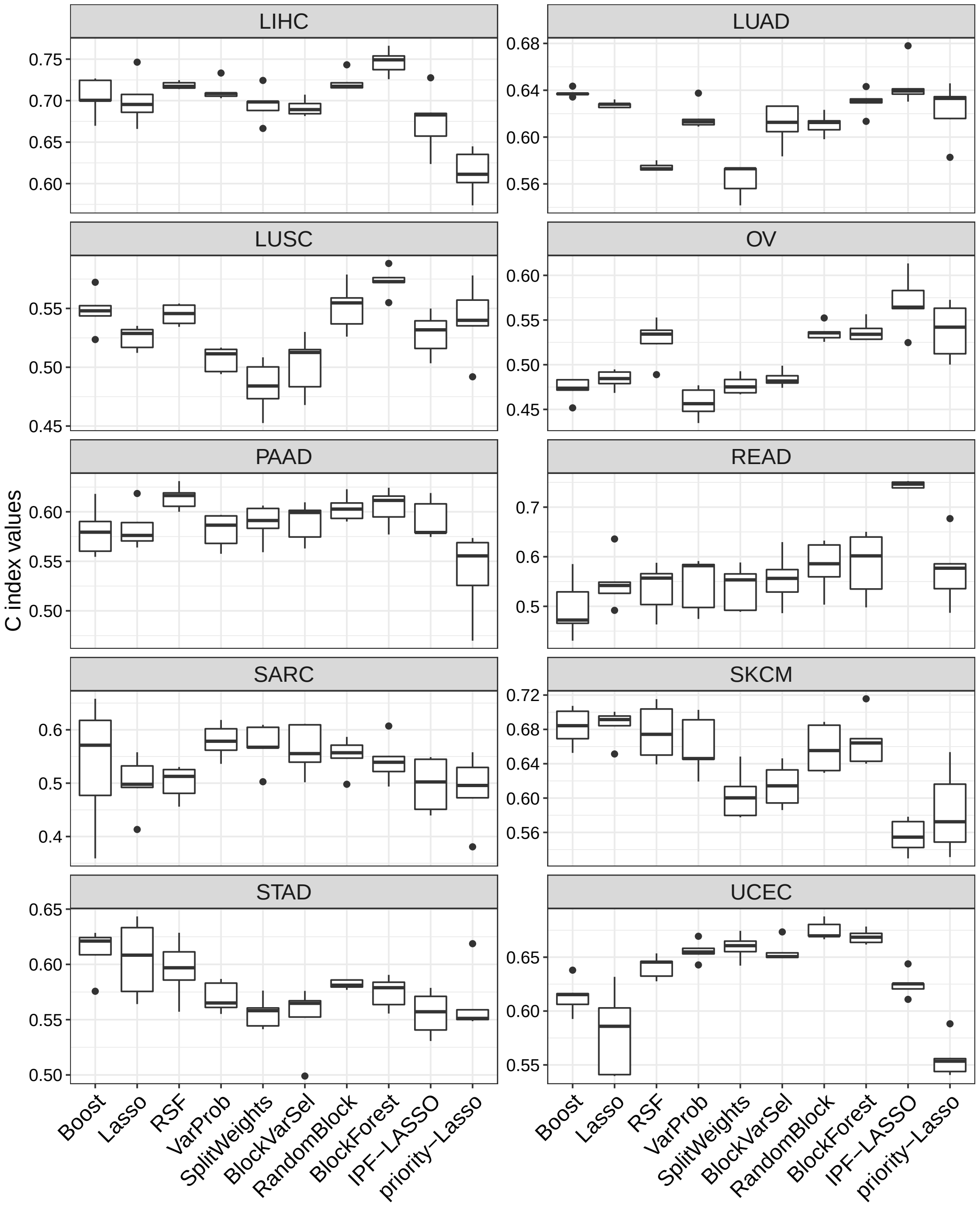

Fig. S2: Multi-omics data: C index values obtained for the individual repetitions of the cross-validation separately for each data set and method – II

B Multi-omics data: Results obtained for the 17 data sets, for which all five blocks were available

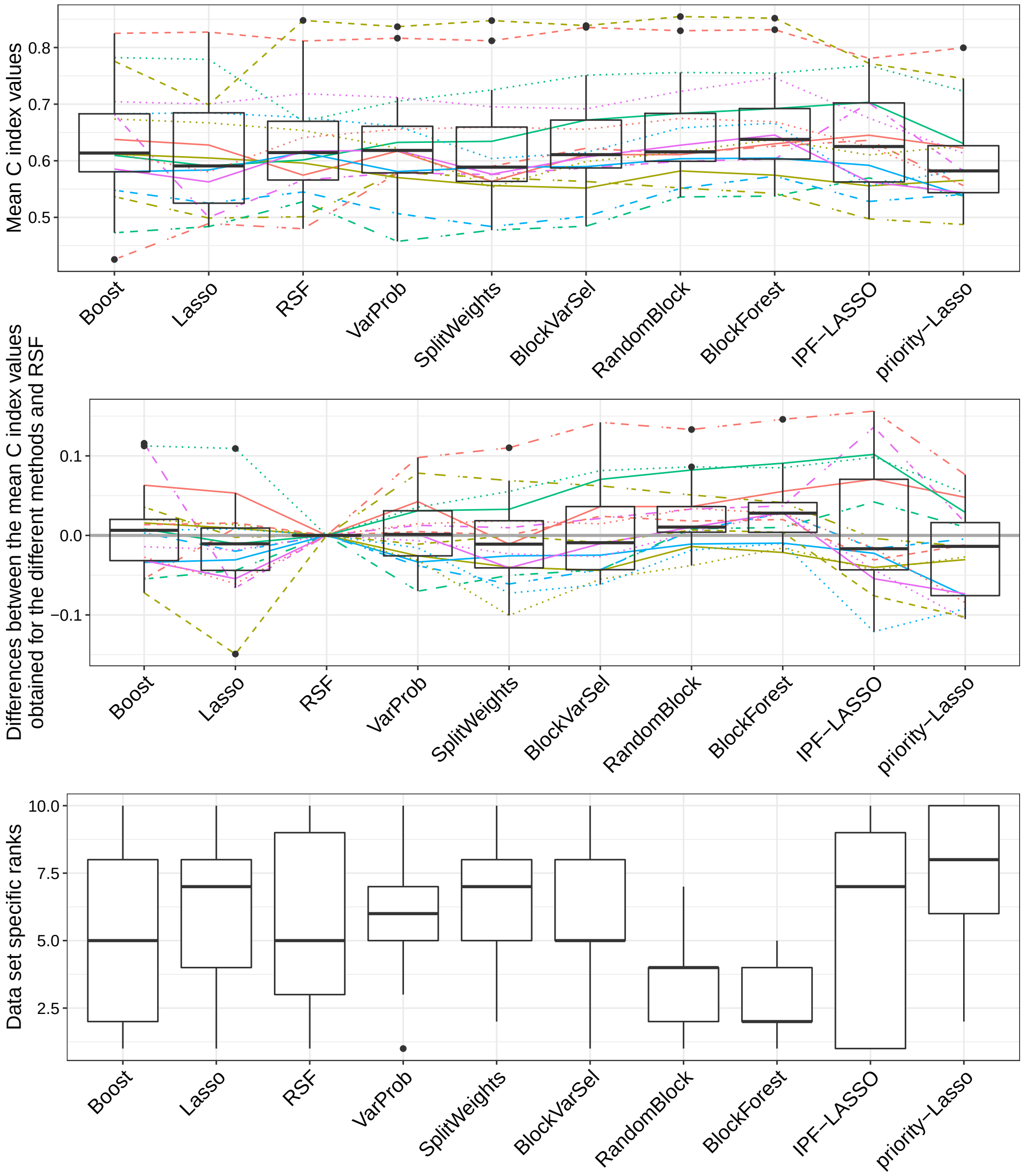

Fig. S3: Multi-omics data: Performances of the ten considered methods on the 17 data sets, for which all five blocks were available. Upper panel: Mean C index values for each data set and each of the ten methods considered. Middle panel: Differences between the mean C index values obtained using the different methods and that obtained using RSF. Lower panel: Data set specific ranks of each method among the other methods in terms of the mean cross-validated C index values. The colored lines in the upper and middle panel connect the results obtained for the same data sets.

C Multi-omics data: Analysis of the influence of data set characteristics on the performance of BlockForest relative to that of RSF

Except for one data set the differences between the mean C index values obtained using RSF and that obtained using BlockForest were not larger than 0.02 for any of the data sets, where BlockForest outperformed RSF for the majority of data sets. The values of the differences between the mean C index values obtained for these methods differed quite strongly across data sets. This suggests that there are certain data set specific factors that determine whether or not we can expect to obtain a considerable improvement through using BlockForest as opposed to RSF. It would be valuable, if these factors and the forms of their influences on the performance differences between BlockForest and RSF would be known. In this way, it would be possible to discern situations in which we can expect BlockForest to perform considerably better than RSF from situations in which there is not much gain in prediction performance by using BlockForest or in which RSF might even be preferable.

Therefore, in this section we present an analysis in which we related the values of several data set specific factors to the values of the differences between the mean C index values obtained for BlockForest and that obtained for RSF. The latter differences are referred to as *diffC* in the following. The investigated data set specific factors are:

- Sample size: n

  BlockForest involves M tuning parameters, which makes this algorithm more complex than standard RSF. We assumed that for larger data sets the optimization of these tuning parameters is more stable than for smaller data sets, which could have the effect that, compared to RSF, for BlockForest there might be a greater gain in prediction performance with increasing sample size.

- Degree of dominance of the most important block: *oneblockimp*

  We hypothesized that the more the predictions are dominated by one of the blocks, the less improvement there will be from using BlockForest (or the other variants) in place of RSF. If almost all information relevant for prediction is contained in only one of the blocks, it is not necessary to exploit interactions between blocks or, more generally, let the other blocks participate in the prediction process. Instead, in this situation it is better to consider almost exclusively covariates from the relevant block. This is, however, already accomplished by the standard RSF algorithm, because the covariate with the best value of the split criterion among the *mtry* randomly sampled covariates will in general stem from the relevant block. By contrast, any potential upweighting of the other blocks performed by the variants will in such situations not be beneficial and can even be harmful. For example, the randomization of the block choice as performed by BlockForest and RandomBlock is counter-productive if there is only one relevant block, because it would be best to simply always use the relevant block in such situations. A notable exception to the tendency of the standard RSF algorithm to select mostly covariates from the (single) relevant block is the case in which this block involves only a few covariates (which happens, e.g., in the case of the clinical block). In this

situation, the covariates from the relevant block will be selected too infrequently, because of the fact that the great majority of the *mtry* sampled covariates will stem from the other blocks. For this reason, it will frequently be the case that a covariate from a non-informative block will divide the samples in the current node better than the best sampled covariate from the informative block simply by chance in this setting.

We measured the degree of dominance of the most important block through the maximum of the b_m values, $m = 1, \ldots, M$, associated with the RandomBlock variant. This metric is denoted as *oneblockimp* in the following.

- Strength of the biological signal: *signal*

  It would be interesting to know, whether the level of biological signal contained in the covariate data has a notable effect on the gain in prediction performance to expect by using BlockForest as opposed to RSF. For example, if it would be known that a particular strong gain in prediction performance can be expected in situations in which the biological signal is strong, it would be particularly recommended to use BlockForest instead of RSF in situations in which a decent level of prediction performance is already attainable using conventional prediction methods that do not take the block structure into account. If, by contrast, a considerable improvement through using BlockForest can be expected for weaker biological signals in particular, BlockForest can be employed effectively in situations in which conventional prediction methods do not deliver good results.

  We measured the degree of biological signal present in a data set by the average of the mean C index value obtained using BlockForest and the mean C index value obtained using RSF. This metric is referred to as *signal* in the following. Note that since the target metric in our analysis is the difference between the mean C index value obtained using BlockForest and the mean C index value obtained using RSF, the plot of the values of this difference *diffC* against the values of *signal* corresponds to a Bland-Altman plot.

In Figure S4, the values of *diffC* are plotted against the values of each of the quantities described above.

There seems to be a general trend that the improvements obtain by using BlockForest become larger for larger data sets. All data sets for which we observe a (small) deterioration by performing BlockForest in place of RSF are small to medium sized data sets. However, there are also small data sets for which BlockForest performed notably better than RSF. For example, in the case of the smallest data set the improvement of BlockForest over RSF was the strongest among all data sets.

The plot of the values of *diffC* against those of *oneblockimp* suggests the following: if no block is dominating the others, a comparably strong improvement might be obtained through the use of BlockForest, but the mere fact that no block is dominating is not a sufficient condition for a comparably strong improvement.

The Bland-Altman plot of the values of *diffC* against those of *signal* resembles a funnel: for smaller values of *signal* the values of *diffC* vary more strongly, that is, for weaker signals, there were more often stronger improvements by performing BlockForest instead of RSF, but also more often merely weak improvements and also (slight) impairments.

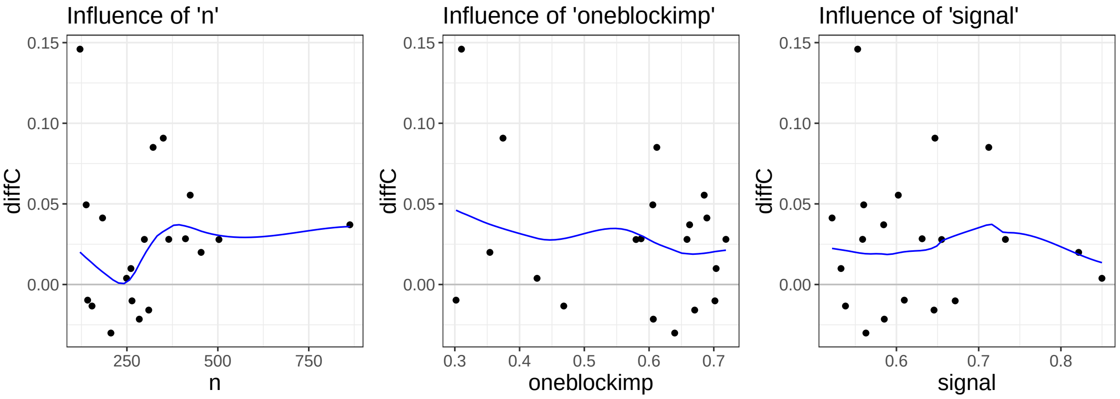

Fig. S4: Multi-omics data: Differences between the mean C index values obtained using Block-Forest and that obtained using RSF plotted against the values of 'n' (left panel), 'oneblockimp' (middle panel), and 'signal' (right panel). The blue lines show LOESS estimates obtained using a re-descending M estimator.

D Multi-omics data: Optimized block-specific tuning parameter values associated with the different variants

Figures S5 to S14 show the optimized values of the tuning parameters associated with the different variants for each data set.

The variable selection probabilities v_m optimized using VarProb are for most data sets considerably larger for the clinical block than for most or all of the omics blocks (Figures S5 and S6). However, for many data sets there are also omics blocks with high optimized values of v_m. This demonstrates that, depending on the considered data set, it can also be effective to sample covariates from certain high-dimensional blocks with the same or even a higher probability as covariates from the low-dimensional clinical block.

The optimized weights w_m associated with SplitWeights (Figures S7 and S8) tend to feature a high variability across cross-validation iterations, which is congruent with the fact that this variant did not perform well in comparison to RSF.

For BlockVarSel the rankings of the blocks with respect to their optimized weights (Figures S9 and S10) are very similar to that obtained with SplitWeights. However, the variabilities of the optimized weights tend to be smaller for BlockVarSel. This reduced variability might be explainable by the fact that with BlockVarSel variables from each block are drawn for each split, which should make the optimization more stable. Note that BlockVarSel quite clearly outperformed SplitWeights, where the superiority of BlockVarSel over SplitWeights might in part be due to the more stable optimization associated with the former procedure.

The values of the block selection probabilities b_m optimized using RandomBlock (Figures S11 and S12) tend to be relatively stable across the cross-validation iterations. As written in Section 'RandomBlock: Random block selection' of the main paper the optimized block selection probabilities can give indications of the relative importances of the different blocks for prediction. We obtained the following mean block selection probabilities across the data sets (sorted from highest to lowest): 0.43 (mutation), 0.29 (RNA), 0.12 (clinical), 0.11 (CNV), 0.07 (miRNA). Thus, the mutation block and the RNA block seem to be by far the most important blocks. Note however that the b_m values depend strongly on the specific set of blocks available in the data sets. As noted in Section 'RandomBlock: Random block selection' of the main paper, for individual data sets, small optimized b_m values must be interpreted with great care, because important blocks can be attributed small optimized b_m values. The latter can occur for blocks that share much predictive information with another block that contains (slightly) more predictive information. In such cases, the latter block will be attributed a high b_m value, whereas the former block that contains (slightly) less predictive information will be attributed a small b_m value even though it contains much predictive information. This is more efficient than attributing high b_m values to both blocks, because if two blocks with strongly overlapping predictive information had high selection probabilities, the information considered across different splits would be more similar. By contrast, if the predictive information contained in two blocks is only mildly overlapping, the b_m values attributed to the two blocks will not strongly correlate and will be similar if the levels of predictive information contained in the two blocks are similar.

We averaged the optimized b_m values per data set for each block and investigated the correlations of these averaged block selection probabilities between the blocks. The strongest negative

correlation ($r \approx -0.90$) we observed was that between the mutation block and the RNA block. In Section 'RandomBlock: Random block selection' of the main paper we described the mechanism that if two informative blocks feature strongly overlapping predictive information, one of these blocks will be attributed a large b_m value and the other one a small b_m value. The fact that for most data sets either the b_m value of the mutation block was very large and that of the RNA block very small or vice versa, suggests that the predictive information contained in these two blocks is both strong and strongly overlapping. The unrealistic alternative explanation for this fact would be that for each of these data sets either the mutation block or the RNA block is important and the respective other one is unimportant. This scenario is unrealistic, because each of these data sets features patients of a different cancer type and both mutation data and RNA data are known to be predictive of cancer [1, 2]. The fact that the RNA block is an informative block throughout cancer types asdf will also become evident in the results obtained for the setting with only the clinical block and the RNA block, where the optimized b_m values obtained for the RNA block were high for the vast majority of data sets (see Section G of Additional file 1). The correlation of the (averaged) b_m values between the clinical block and the mutation block was -0.43, while it was 0.10 between the clinical block and the RNA block. The fact that the latter correlation is small and, more importantly, non-negative suggests that the information overlap between the clinical block and the RNA block is weak, which in turn suggests a high additional predictive value of the RNA block over the clinical block. By contrast, the fact that the correlation between the clinical block and the mutation block is negative suggests a stronger information overlap between these two blocks. Thus, the additional predictive value of the mutation block over the clinical block might in general be smaller than that of the RNA block over the clinical block.

The optimized weight values associated with BlockForest (Figures S13 and S14) are quite similar to those associated with BlockVarSel. However, with BlockForest there are more often data sets for which there are several blocks with very large optimized weights. This can be explained by the fact that when considering each block for each split, as performed with BlockVarSel, the weights of the more informative blocks (like those of the weak blocks) need to be sufficiently distinct. This inhibits non-optimal blocks from being used too often for splitting, but at the same time prevents large weights being attributed to several of the more informative blocks. The block randomization of BlockForest, by contrast, has the effect that the more informative blocks are frequently not considered at the same time. As a consequence, these blocks do not compete strongly in the split selection, which is why it is not that important that the weights attributed to these blocks differ strongly. This allows for greater differences between the set of weights of the more informative blocks on the one hand and that of the weights of the weak blocks on the other, ensuring that the weak blocks are not used too often for splitting.

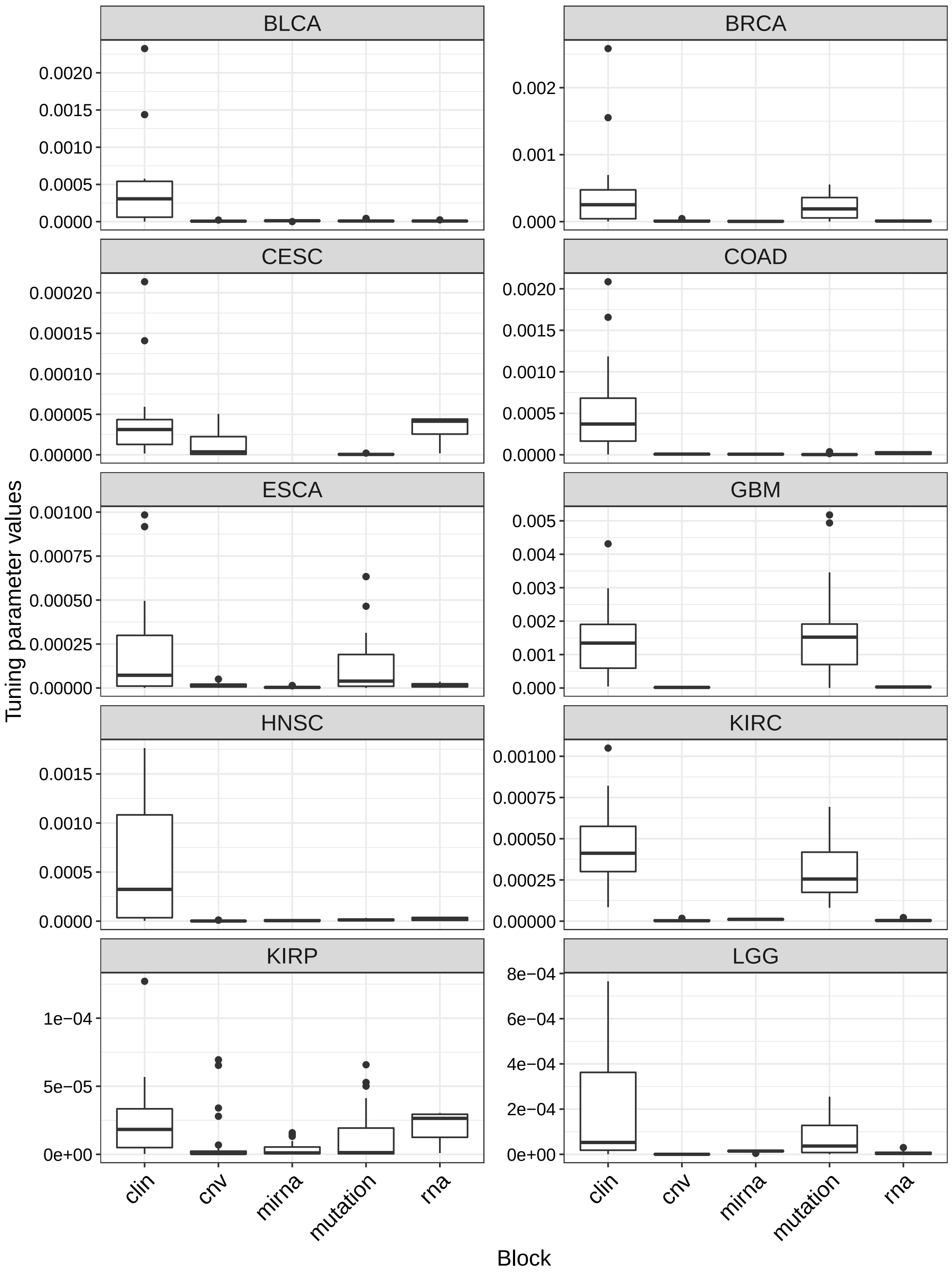

Fig. S5: Multi-omics data: v_m values optimized for variant VarProb – I

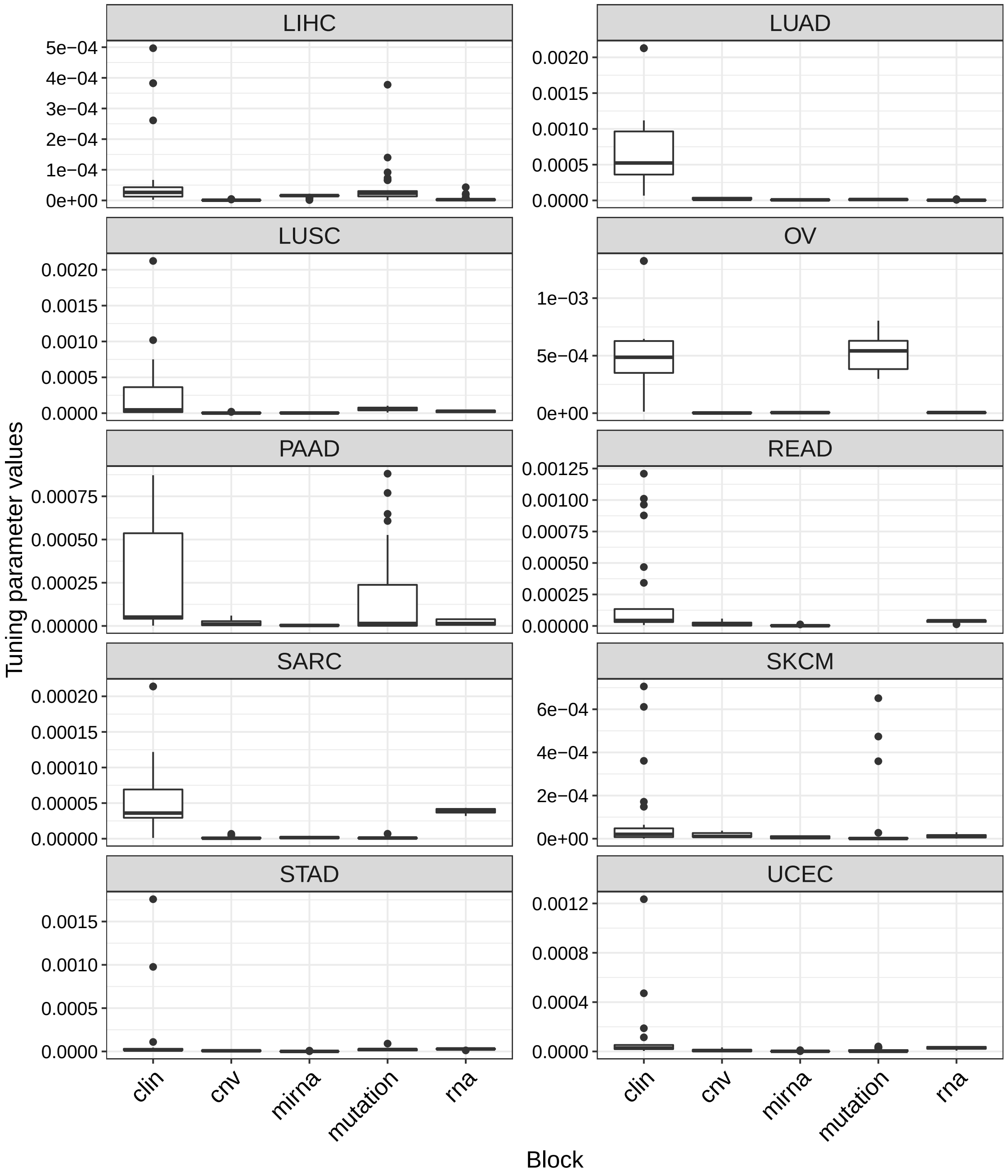

Fig. S6: Multi-omics data: v_m values optimized for variant VarProb – II

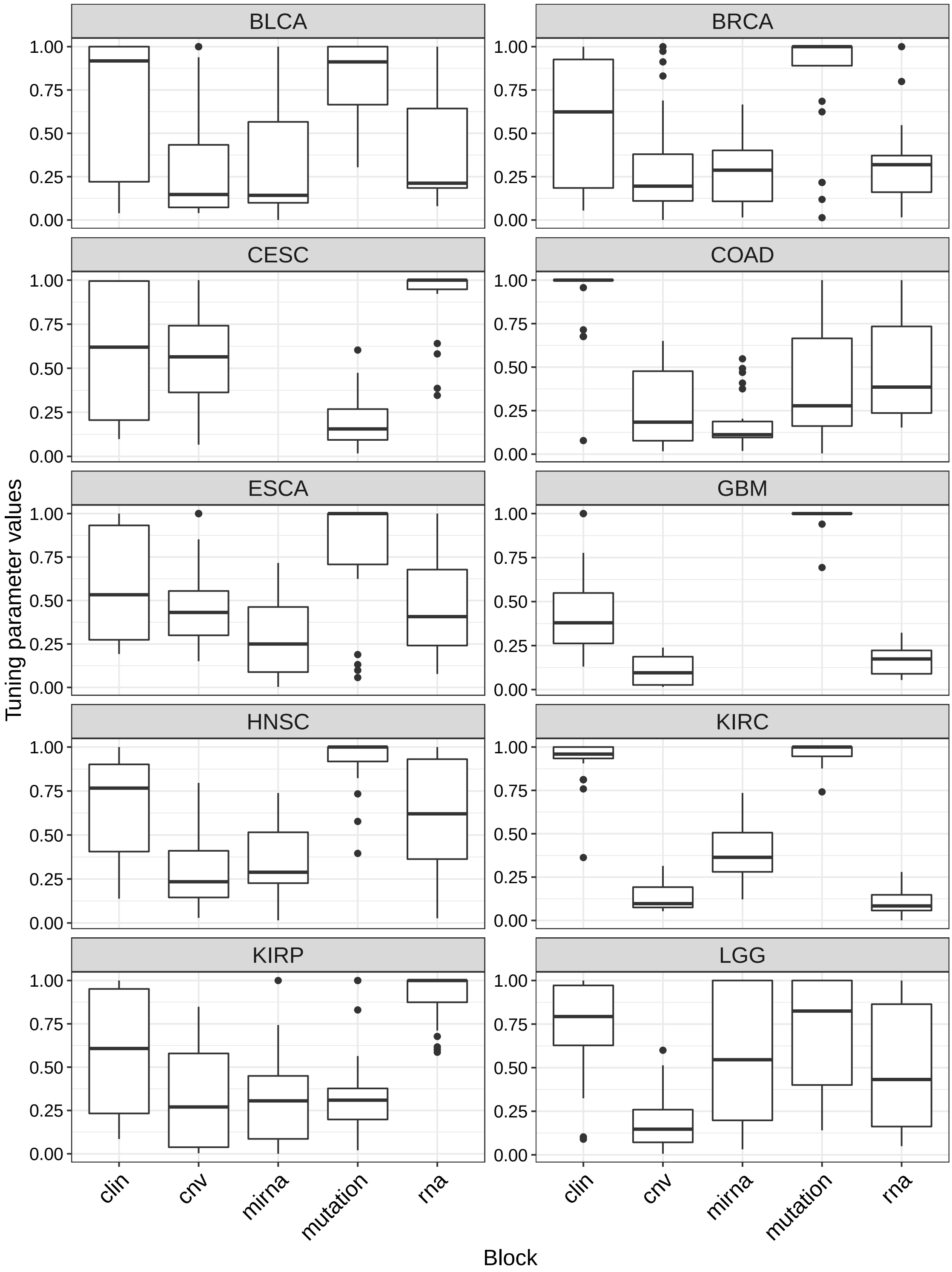

Fig. S7: Multi-omics data: w_m values optimized for variant SplitWeights – I

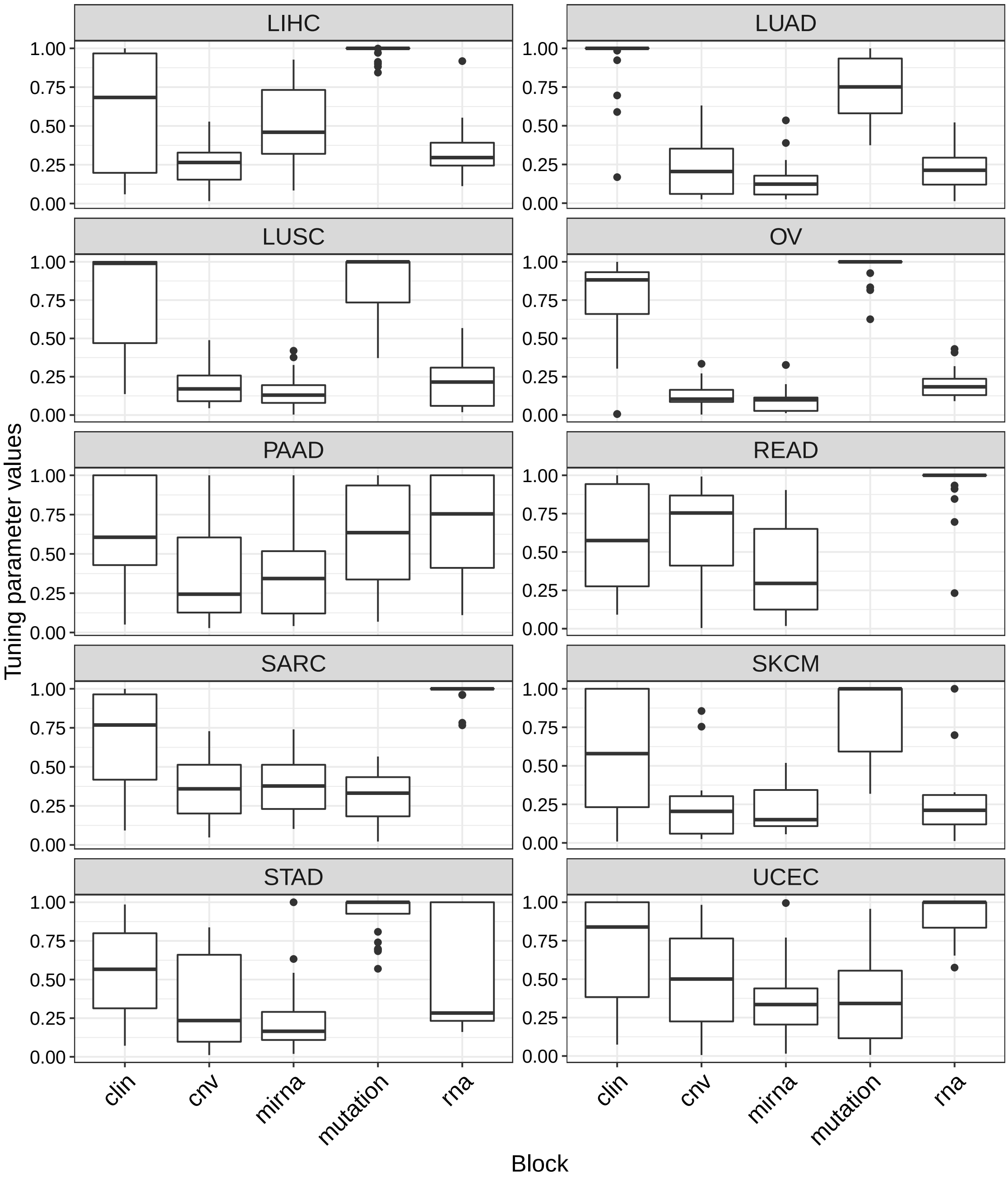

Fig. S8: Multi-omics data: w_m values optimized for variant $\text{SplitWeights} - \text{II}$

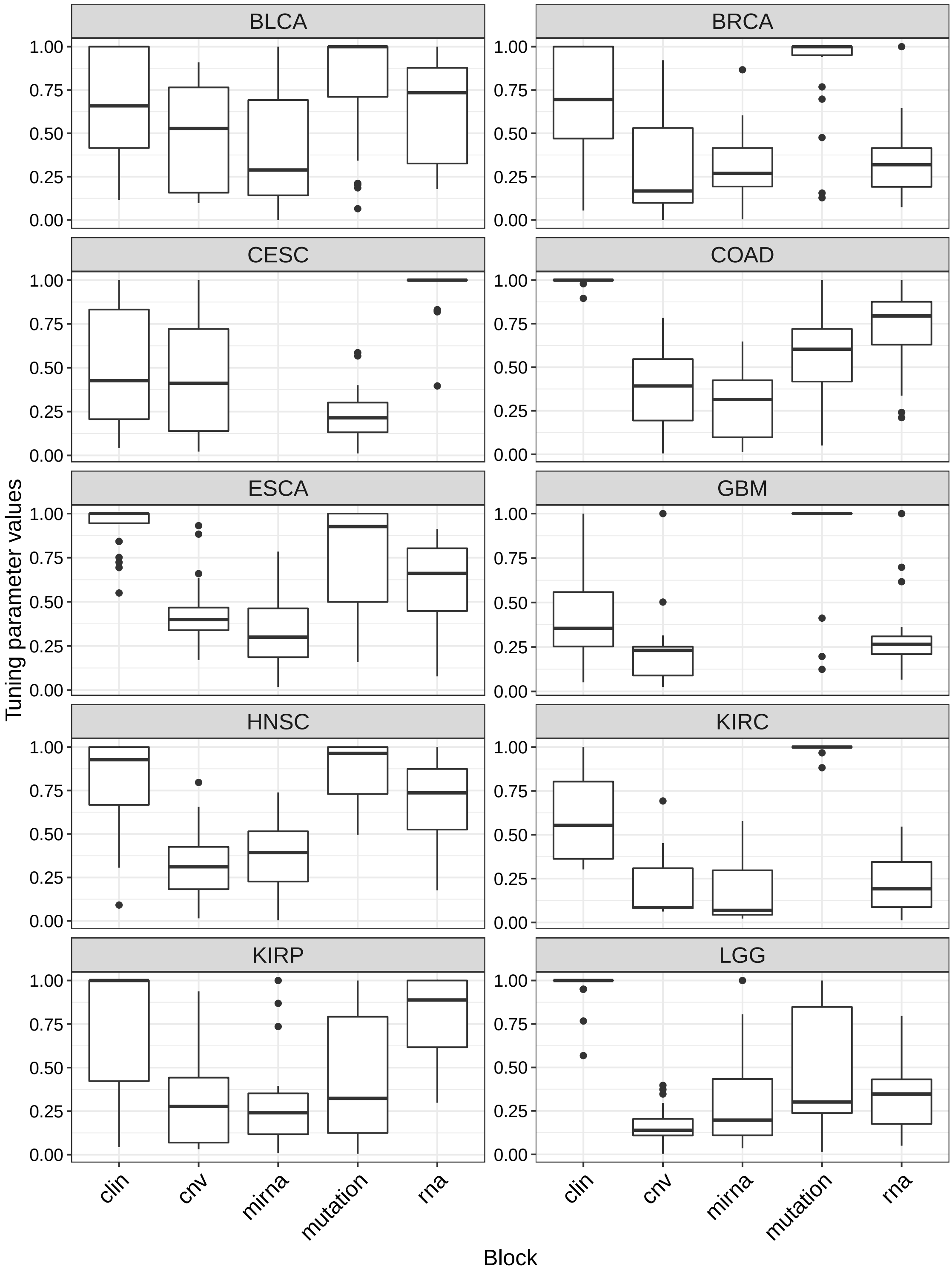

Fig. S9: Multi-omics data: w_m values optimized for variant BlockVarSel – I

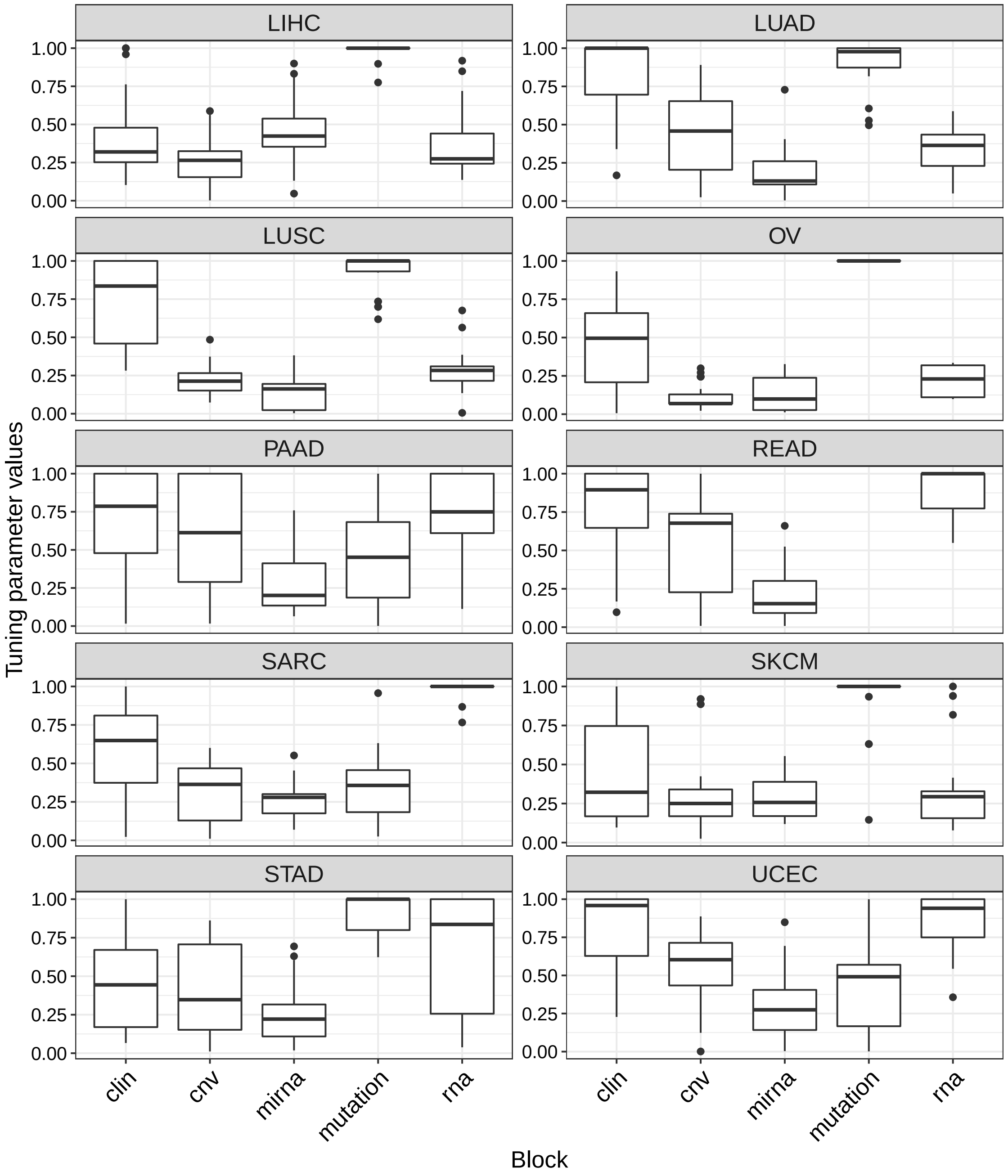

Fig. S10: Multi-omics data: w_m values optimized for variant BlockVarSel – II

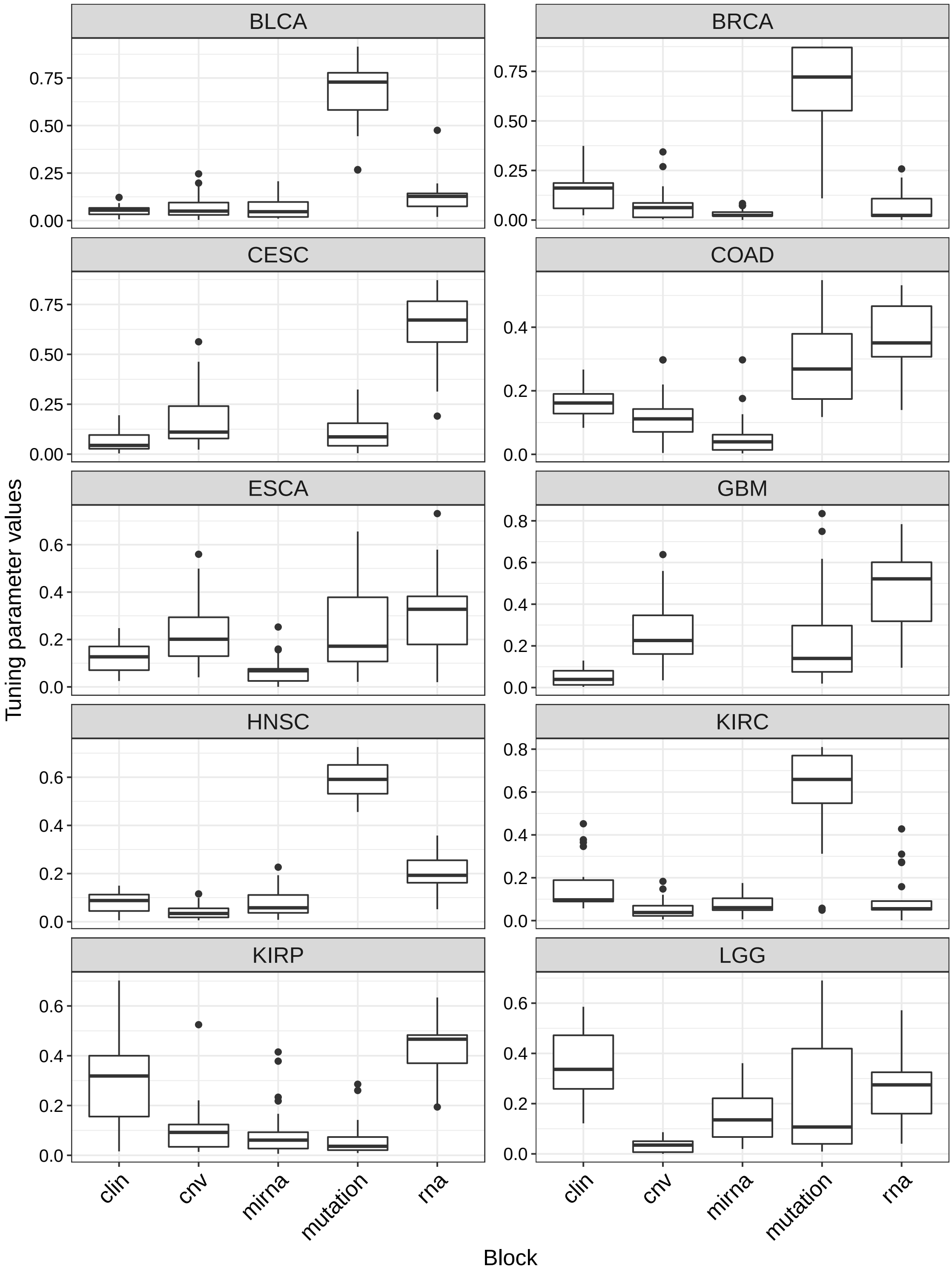

Fig. S11: Multi-omics data: b_m values optimized for variant RandomBlock – I

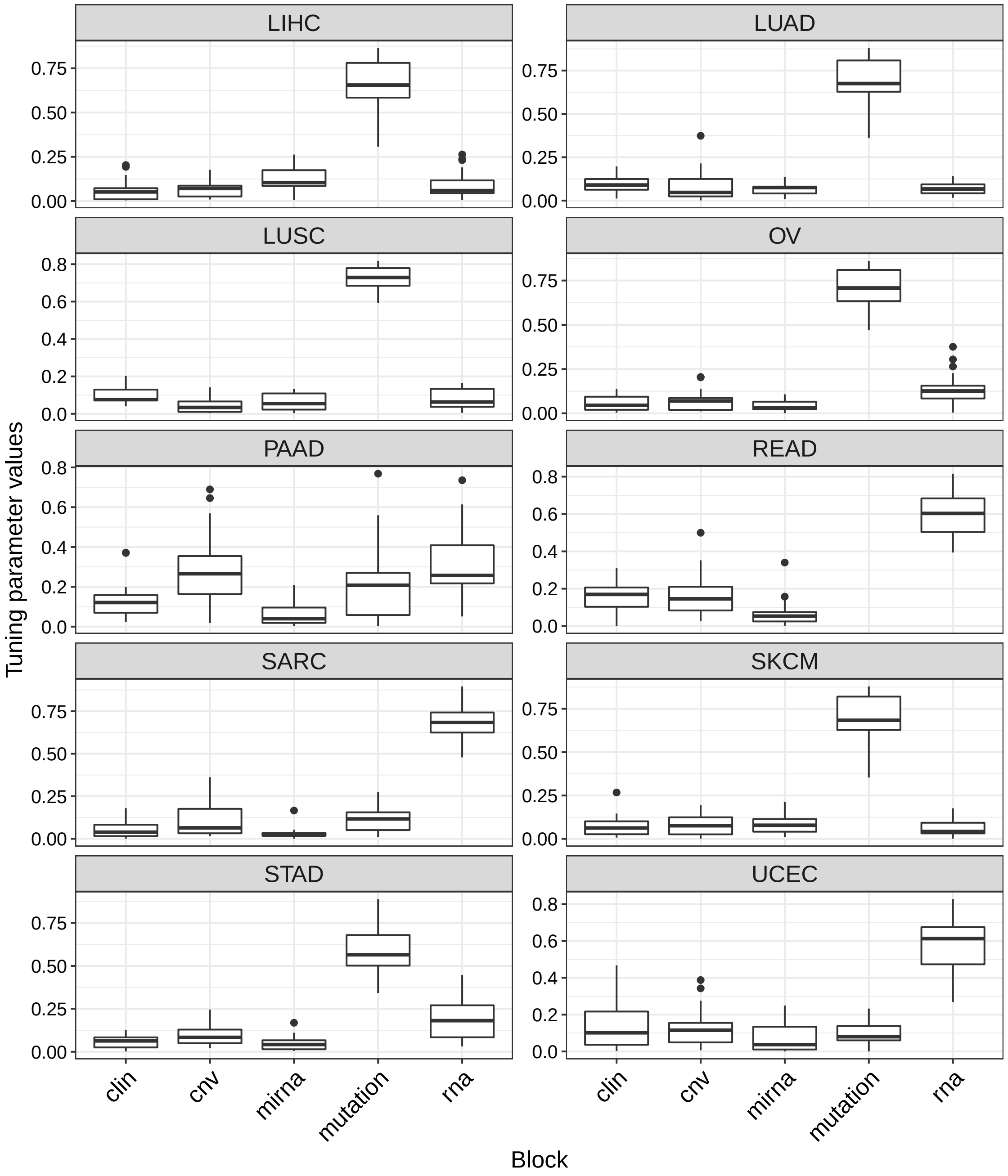

Fig. S12: Multi-omics data: b_m values optimized for variant RandomBlock – II

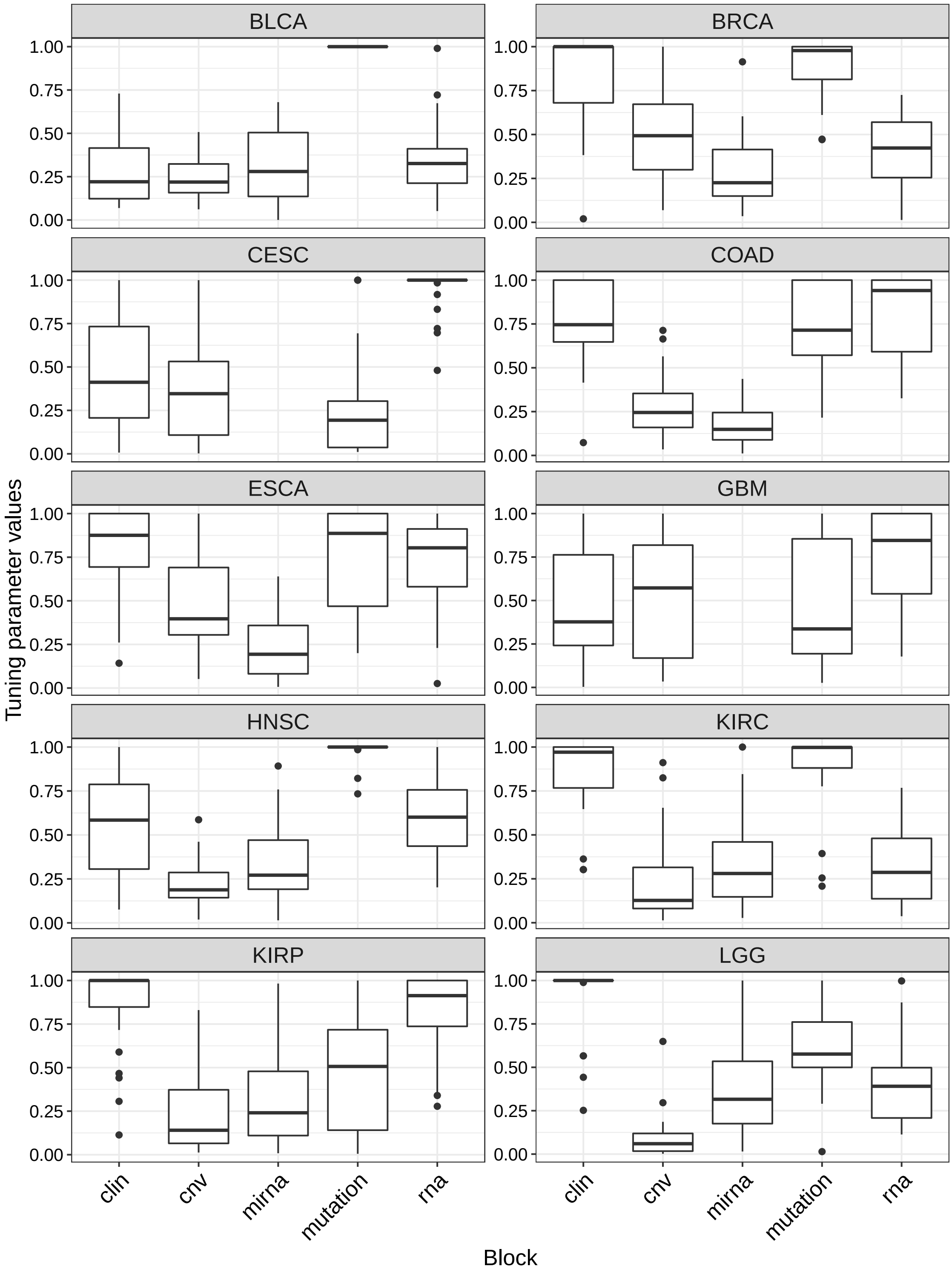

Fig. S13: Multi-omics data: w_m values optimized for variant BlockForest – I

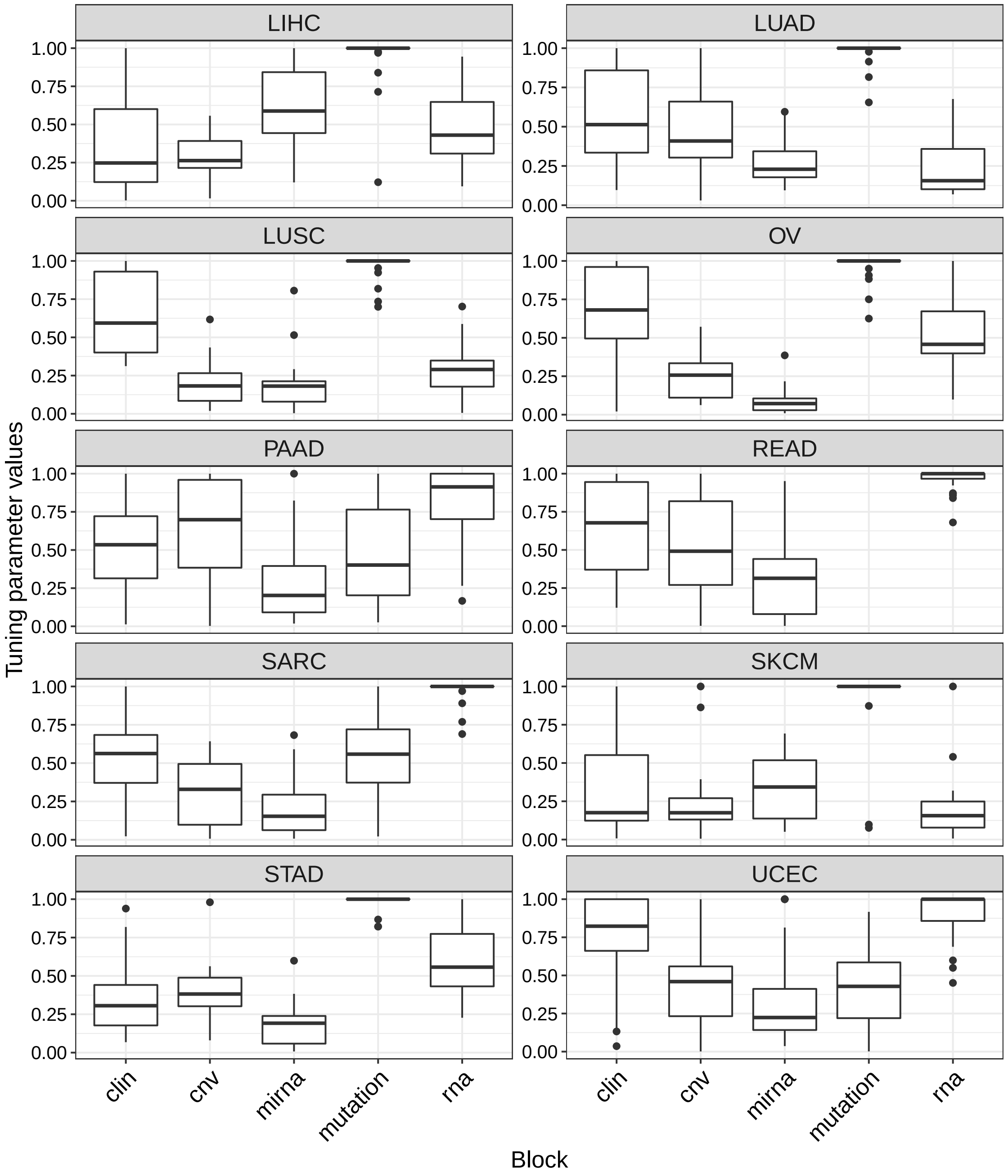

Fig. S14: Multi-omics data: w_m values optimized for variant BlockForest – II

E Clinical covariates plus RNA measurements: C index values obtained for the individual repetitions of the cross-validation

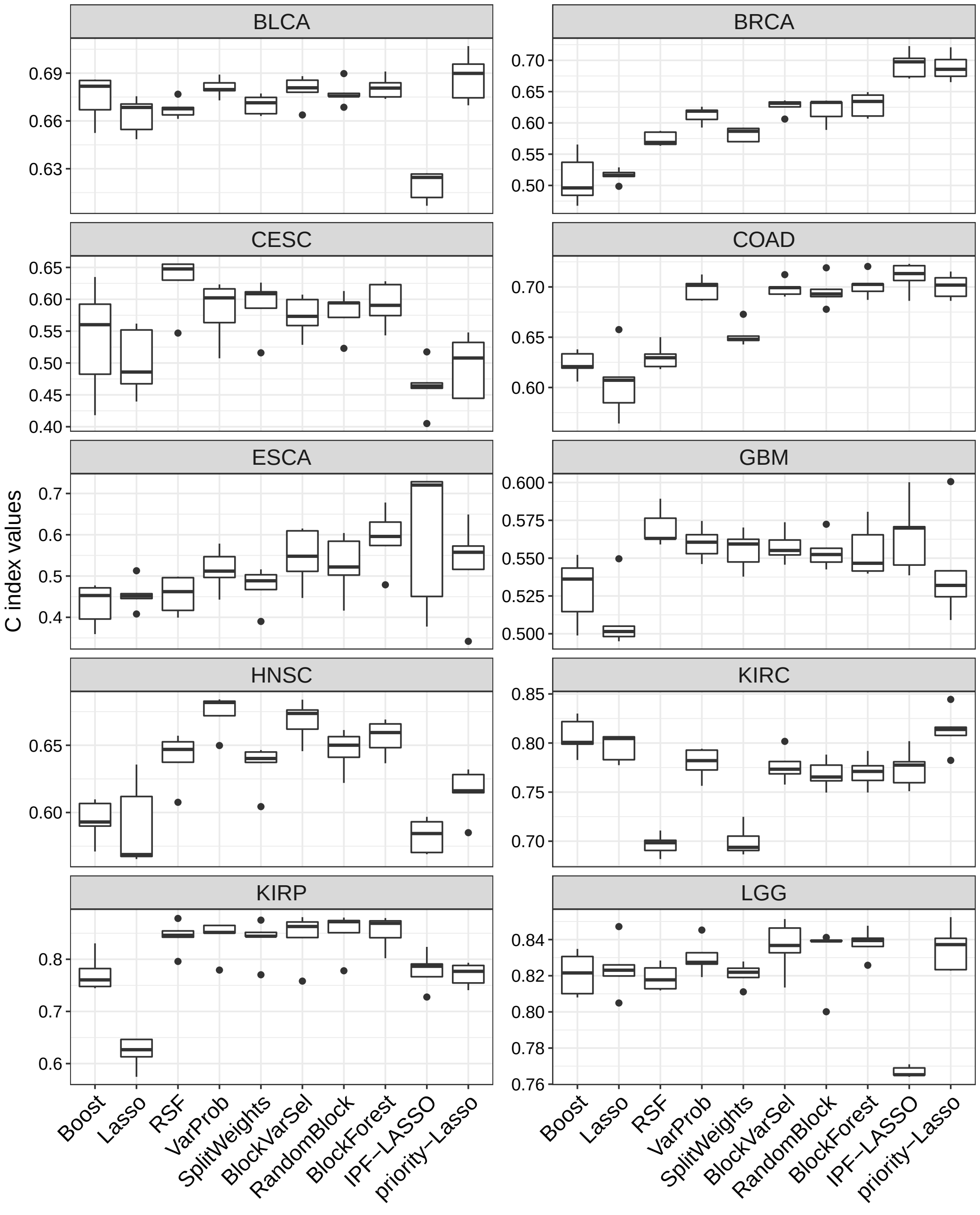

Fig. S15: Clinical covariates plus RNA measurements: C index values obtained for the individual repetitions of the cross-validation separately for each data set and method – I

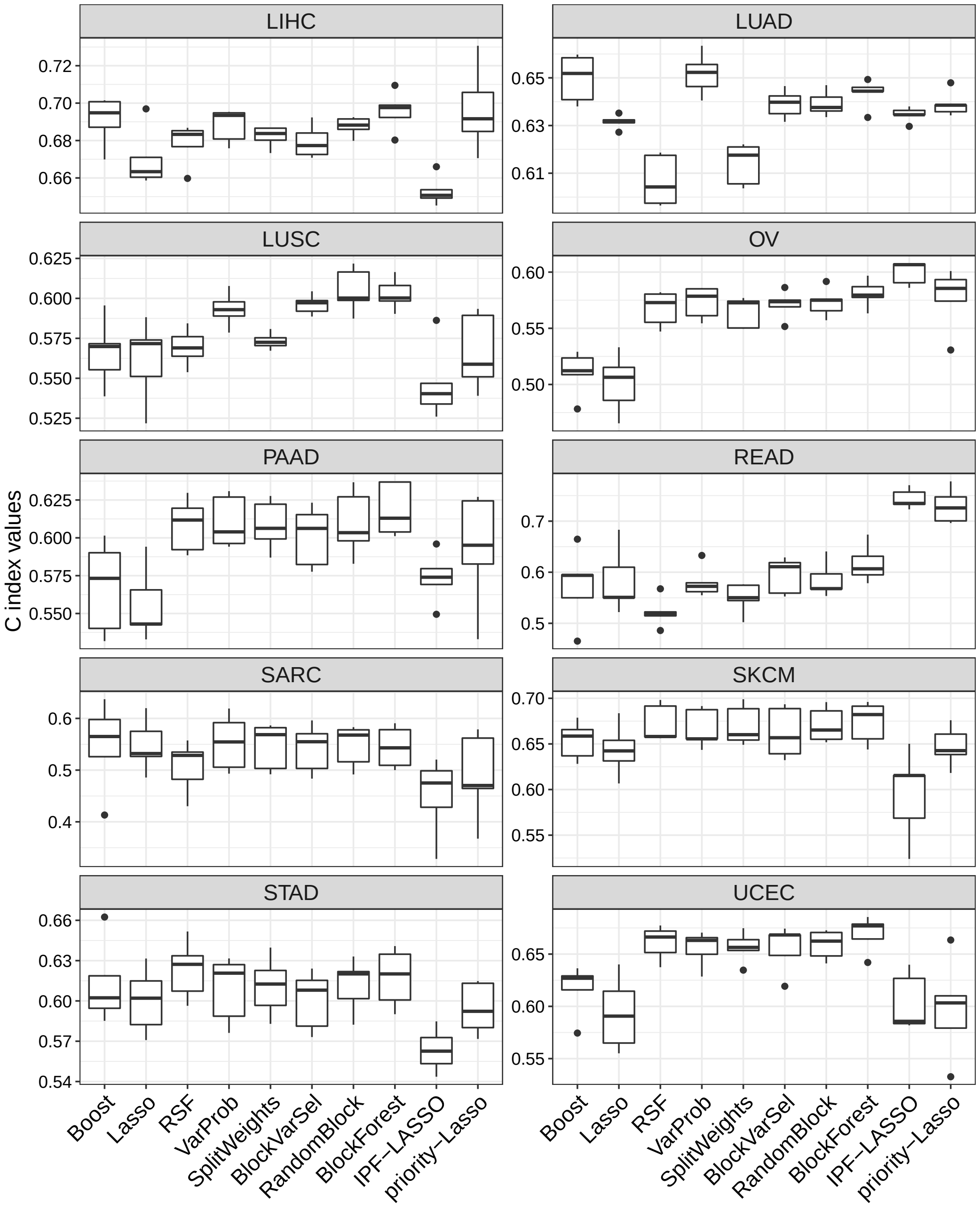

Fig. S16: Clinical covariates plus RNA measurements: C index values obtained for the individual repetitions of the cross-validation separately for each data set and method – II

F Clinical covariates plus RNA measurements: Analysis of the influence of data set characteristics on the performance of BlockForest relative to that of RSF

In Section C of Additional file 1 we presented an analysis of the influence of certain data set characteristics on the performance of BlockForest relative to that of RSF for the multi-omics data. We performed an analogous analysis for the case of having clinical covariates plus RNA measurements. The design of the analysis is the same as that of the analysis presented in Section C with a single exception: Instead of investigating the influence of the maximum *oneblockimp* of the b_m values optimized using RandomBlock, we investigated the influence of the optimized b_m value of the clinical block. The latter will be denoted as b_{clin} in the following. We expected that the larger the value of b_{clin} becomes, the stronger will be the improvement of BlockForest over RSF. We assumed the latter to be the case, because large values of the optimized selection probability b_m of the clinical block indicate that there is much prediction information contained in the clinical covariates, making it particularly effective to exploit the predictive information contained in these covariates.

In Figure S17, the values of *diffC* are plotted against the values of each of the three quantities n, b_{clin}, and *signal*.

In comparison to the multi-omics case, the relation between the sample size n and the values of *diffC* is not clearly positive. While the greatest *diffC* values were obtained for very small data sets, the three data sets for which RSF performed better than BlockForest were also small to medium sized. The fact that we do not observe a clearly positive association between n and *diffC* here might be explained by the fact that when including only the clinical block and the RNA block, only two tuning parameters have to be optimized instead of five (or sometimes four) in the multi-omics case. The optimized tuning parameter values can be expected to be more precise when there are only two blocks compared to when there are five (or four), in particular for small sample sizes. This is because, in general, the smaller the number of parameters in a model, the more precise will be the estimates of these parameters. For this reason, a common rule of thumb in linear regression is that at least 10 to 15 observations are required for each included covariate, because an estimate of the coefficient of each covariate in the model has to be obtained.

As we had expected, there is a quite clear positive association between the values of b_{clin} and the *diffC* values. The b_{clin} values of the two data sets for which BlockForest performed the worst in comparison to RSF were smaller than 0.1. For these two data sets the clinical covariates do obviously carry little to no predictive information that is not contained in the RNA block, which is why it is not expected that BlockForest would perform better than RSF here. Indeed, if the clinical block carries almost no predictive information, we would rather expect BlockForest to perform worse than RSF for the following reason: Given the fact that with BlockForest for each split, each block is sampled with probability 0.5, where this sampling is repeated until at least one block is drawn, for 33% of the splits only covariates from the clinical block are considered. Thus, given the fact that there is almost no predictive information in the clinical block, at least 33% of splits in the forest are not informative, independently of the optimized values of the weights w_1 and w_2.

The relation between *signal* and *diffC* seems to be weakly negative overall, meaning that the improvement in prediction performance by performing BlockForest instead of RSF tends to be stronger for less strongly predictive covariates. Nevertheless, for the three data sets for which RSF performed worse than BlockForest, the signal is rather weak.

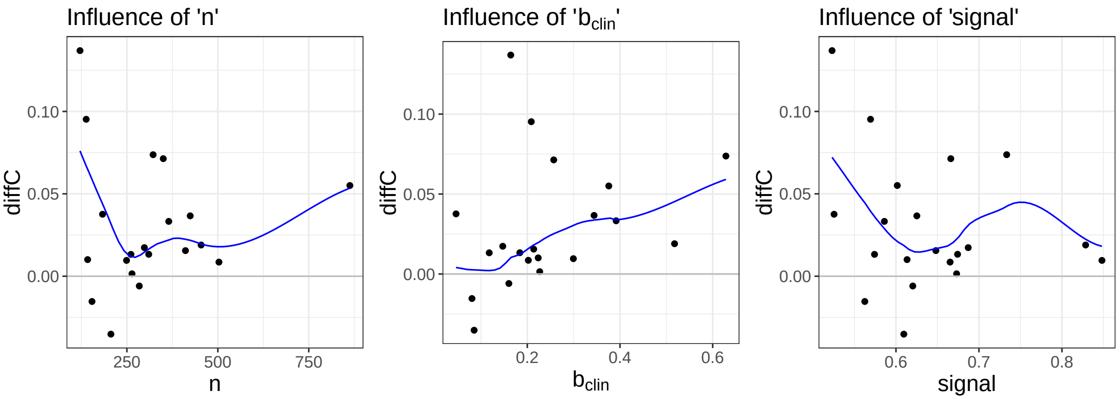

Fig. S17: Clinical covariates plus RNA measurements: Differences between the mean C index values obtained using BlockForest and that obtained using RSF plotted against the values of 'n' (left panel), 'b_{clin}' (middle panel), and 'signal' (right panel). The blue lines show LOESS estimates obtained using a re-descending M estimator.

G Clinical covariates plus RNA measurements: Optimized block-specific tuning parameter values associated with the different variants

The values of the optimized selection probabilities associated with VarProb (Figures S18 and S19) are for all data sets larger for the clinical block than those for the RNA block. This suggests that the clinical block contains some predictive information for each data set. For most data sets, the optimized selection probabilities are very small for the RNA block and comparably large for the clinical block. This was different for the multi-omics data, where there were frequently large optimized selection probabilities for some omics blocks.

For most data sets, the weight values of the clinical block optimized with SplitWeights (Figures S20 and S21) take the value one for the great majority of the cross-validation iterations, while the optimized weights of the RNA block are much smaller and quite variable across the cross-validation iterations.

The weight values optimized with BlockVarSel (Figures S22 and S23) are similar to those optimized with SplitWeights. However, the optimized weights of the RNA block tend to be larger than those optimized with SplitWeights. This can be explained as follows: For BlockVarSel the weights of the clinical block do not have to be as high in comparison to that of the RNA block, because of the fact that with BlockVarSel for each split covariates from both blocks are drawn. Therefore, the average numbers of clinical covariates considered per split are higher for BlockVarSel. As a consequence the value of the weight attributed to the clinical block compared to that attributed to the RNA block must be smaller for BlockVarSel in order to obtain the same frequency of splits performed using one of the clinical covariates as when using SplitWeights.

For most data sets, the values of the block selection probabilities optimized using RandomBlock (Figures S24 and S25) differ only weakly across the cross-validation iterations. For 18 of the 20 data sets the optimized block selection probability of the RNA block is larger than that of the clinical block. The mean optimized block selection probabilities across data sets are as follows: 0.24 (clinical), 0.76 (RNA). In the median, the block selection probability of the RNA block was 3.7 higher than that of the clinical block. This can be interpreted as meaning that the RNA block was in the median 3.7 times as important for prediction as the clinical block for this collection of data sets when considering only the clinical block and the RNA block.

While the optimized weight of the clinical block was higher than that of the RNA block for the majority of data sets in the case of BlockVarSel, it is the other way round in the case of BlockForest (Figures S26 and S27). This can be explained as follows: Due to the block sampling procedure of BlockForest, for 33% of the splits only the clinical block is considered for splitting with this method. Therefore, independently of the optimized weights, with BlockForest at least 33% of the splits will use a clinical covariate for splitting. This is not the case with BlockVarSel, where the clinical covariates compete more strongly with the RNA measurements. The clinical block is most often attributed a higher weight in this setting to compensate for the fact that there are many more RNA measurements than clinical covariates.

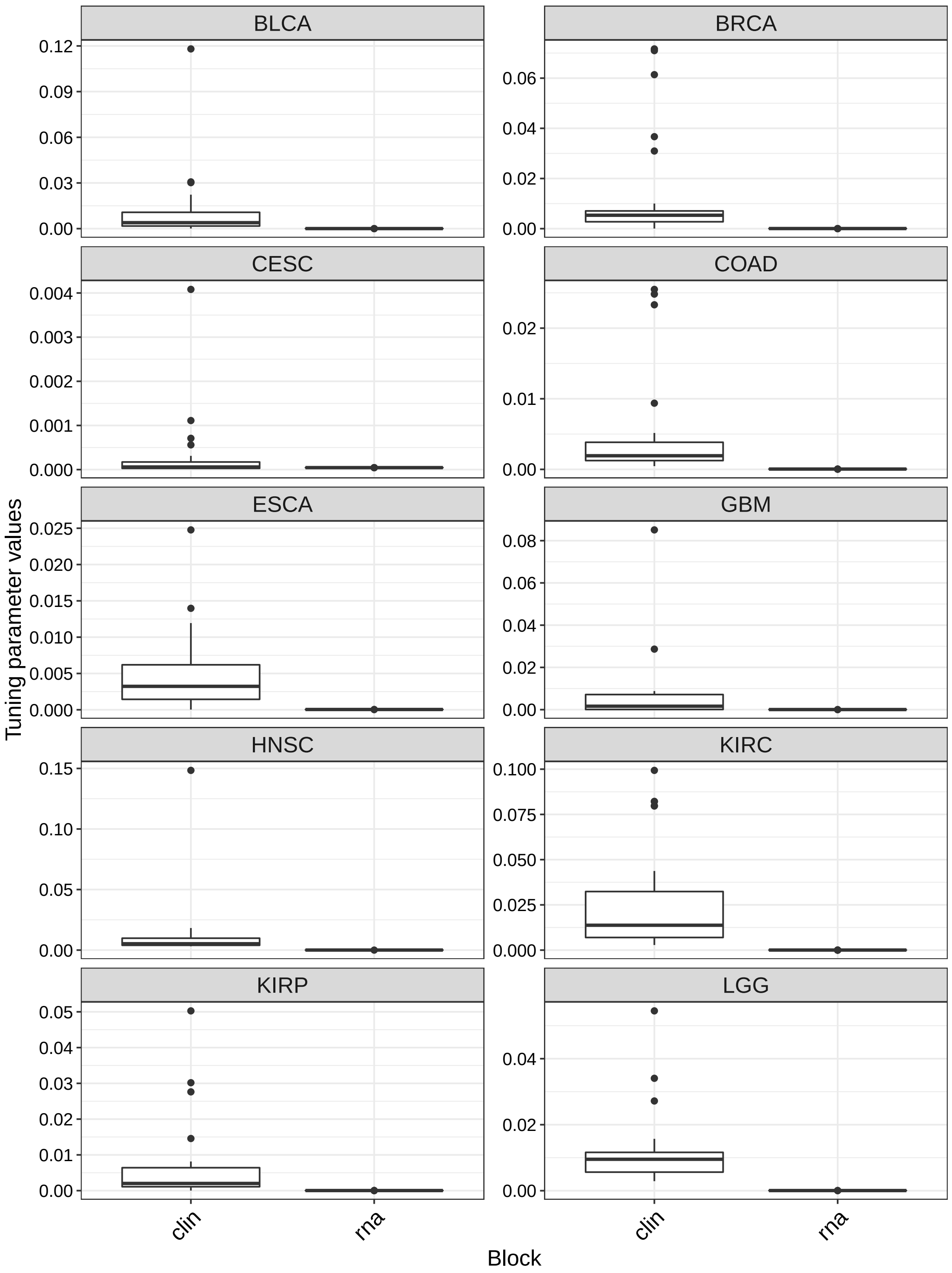

Fig. S18: Clinical covariates plus RNA measurements: v_m values optimized for variant VarProb – I

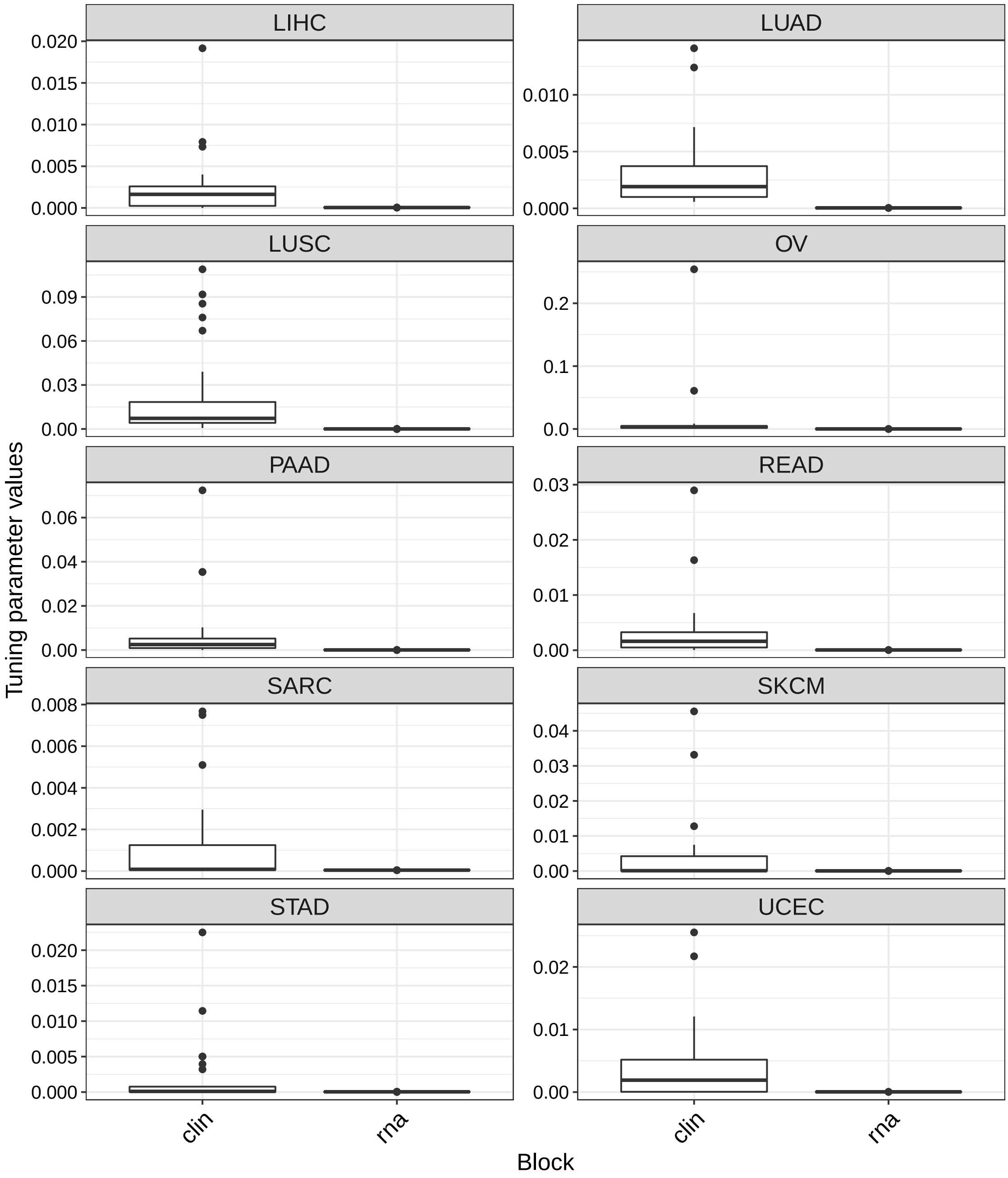

Fig. S19: Clinical covariates plus RNA measurements: v_m values optimized for variant VarProb – II

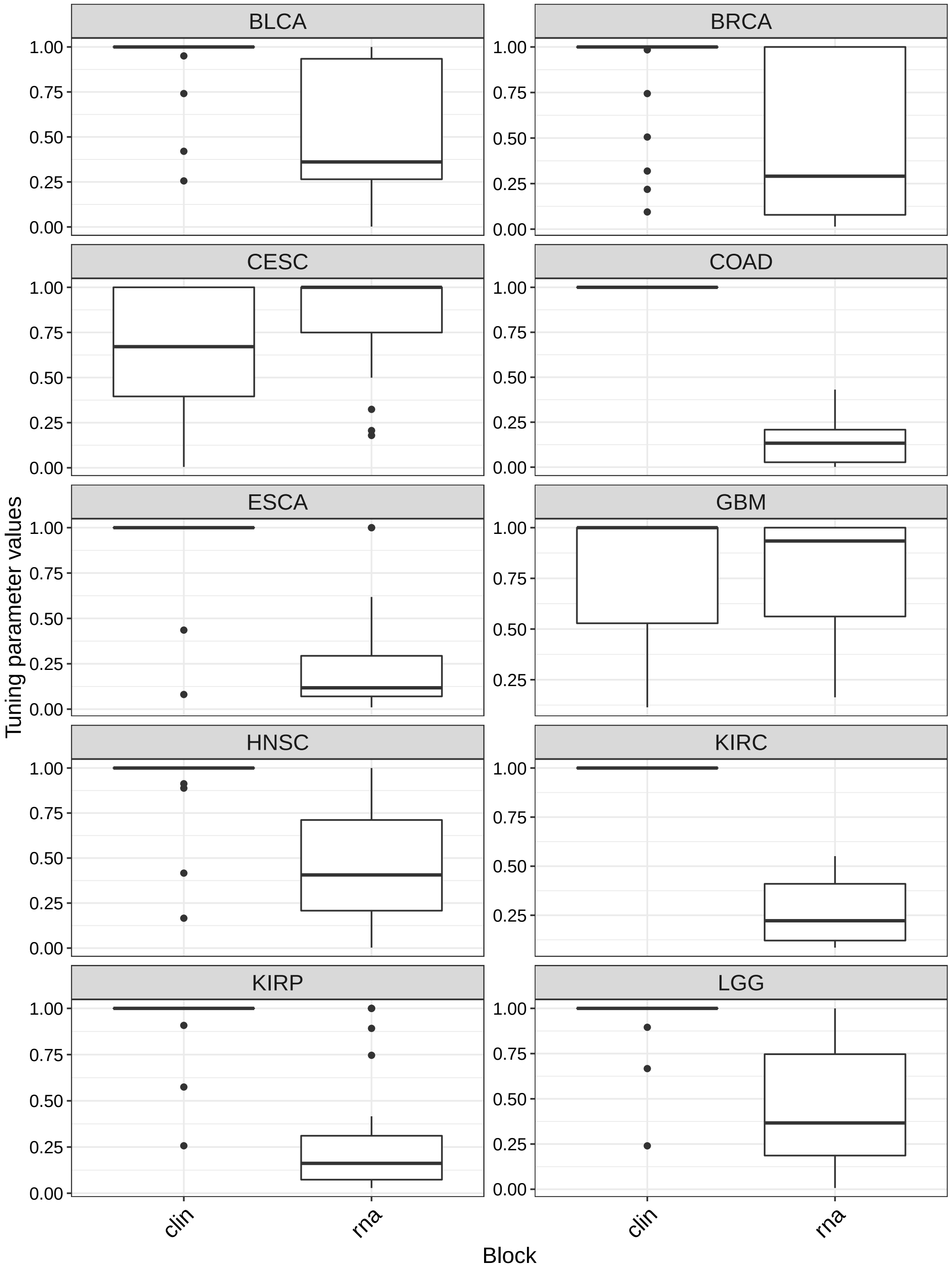

Fig. S20: Clinical covariates plus RNA measurements: w_m values optimized for variant SplitWeights $-$ I

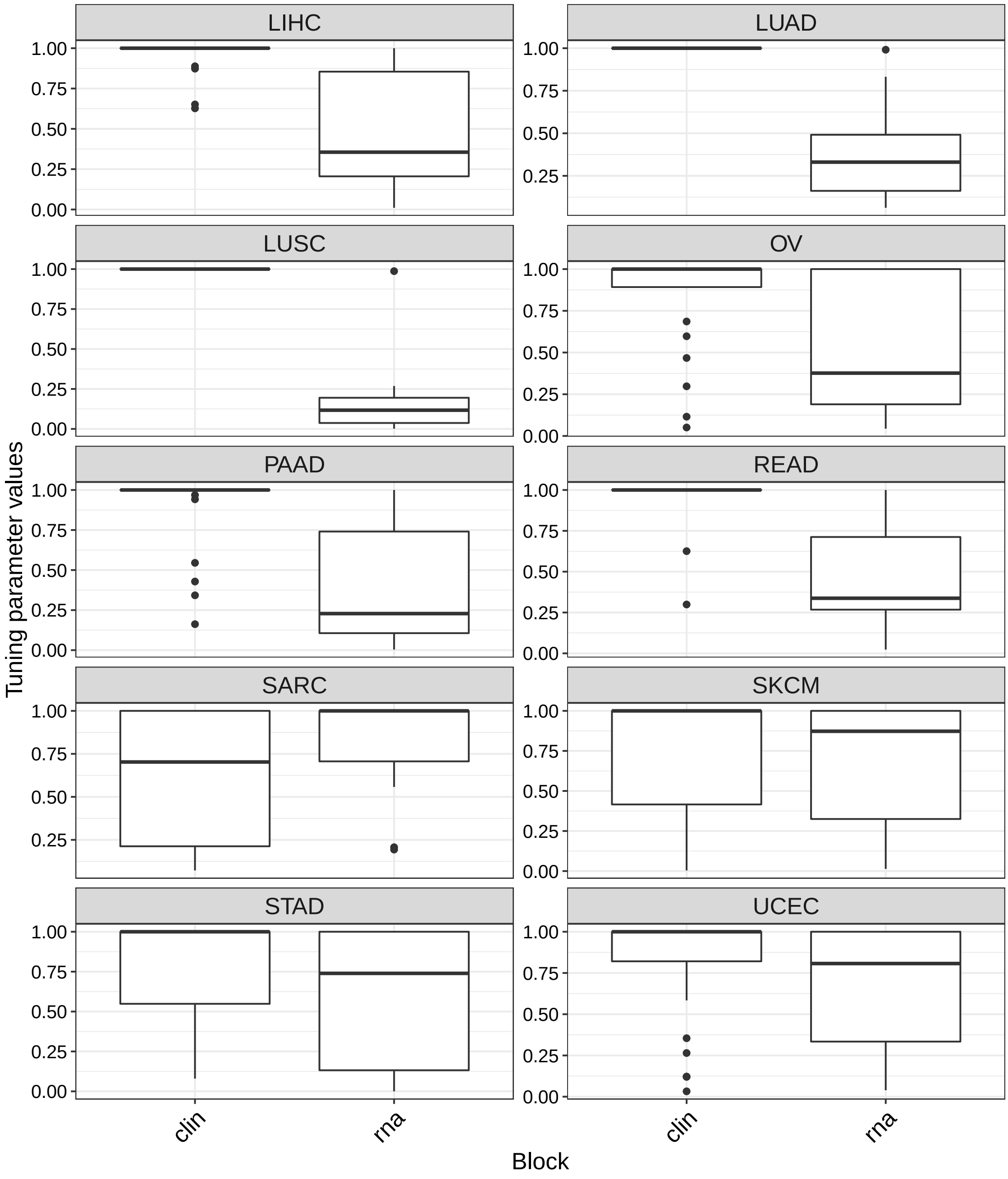

Fig. S21: Clinical covariates plus RNA measurements: w_m values optimized for variant SplitWeights − II

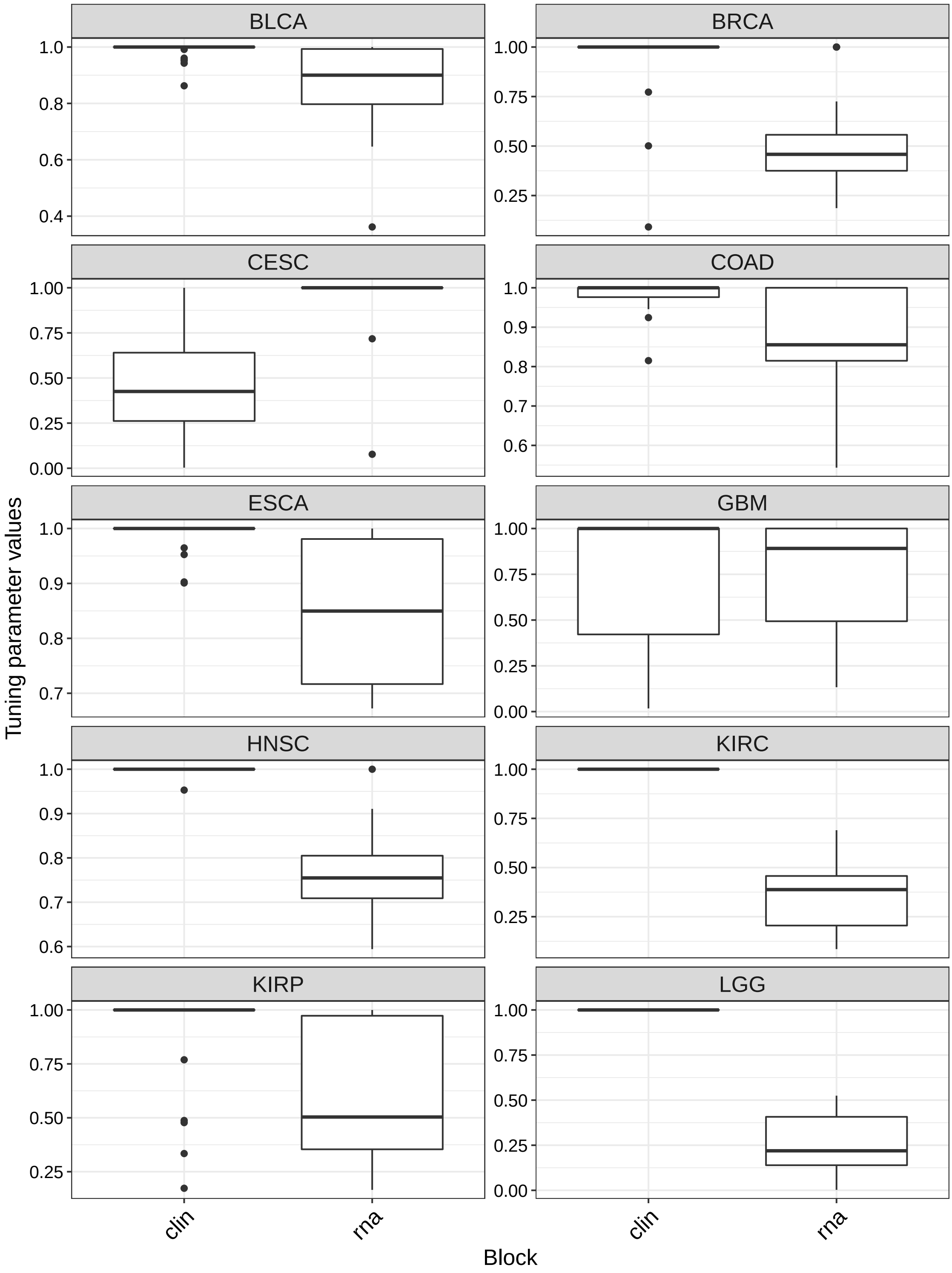

Fig. S22: Clinical covariates plus RNA measurements: w_m values optimized for variant BlockVarSel – I

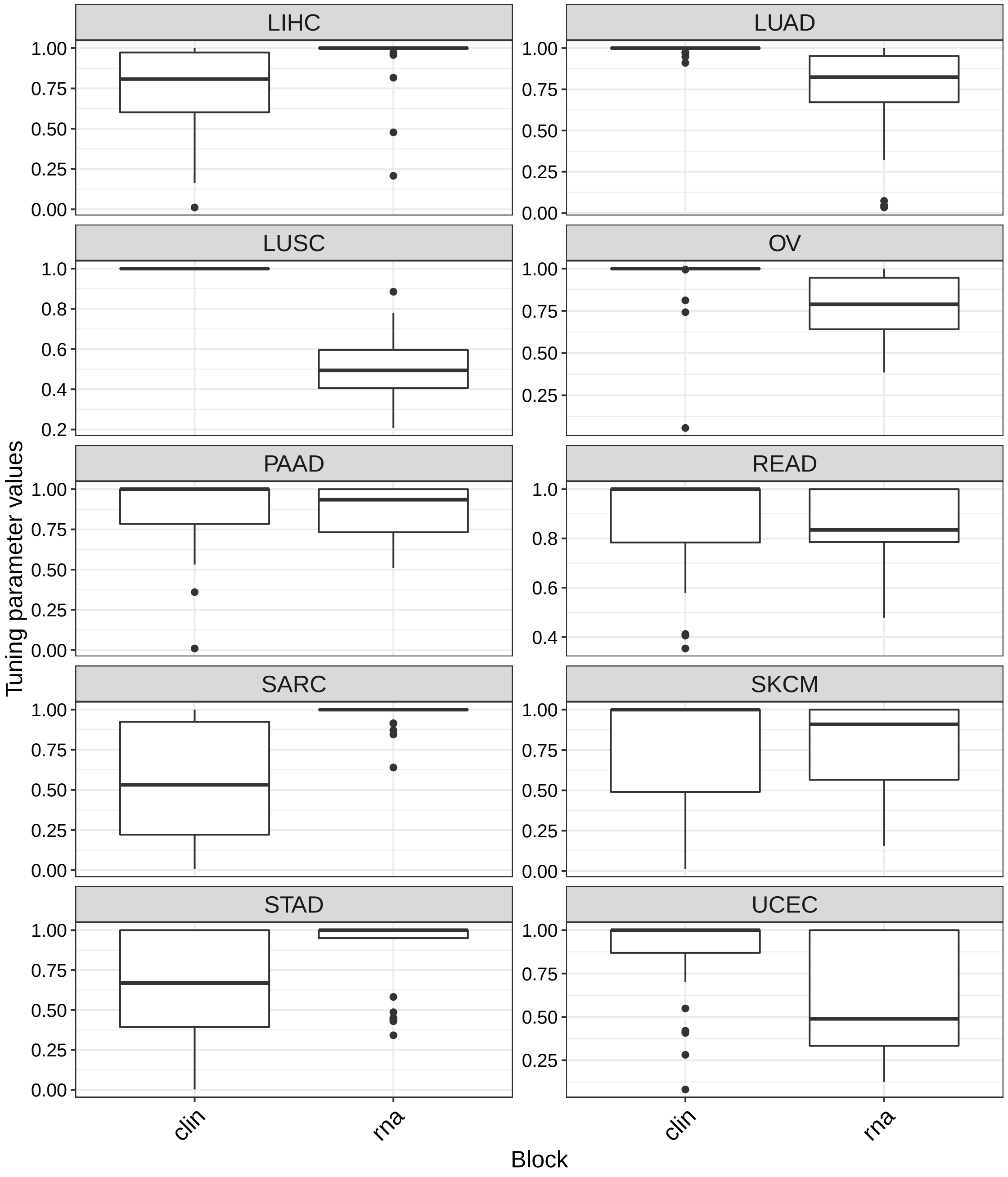

Fig. S23: Clinical covariates plus RNA measurements: w_m values optimized for variant BlockVarSel – II

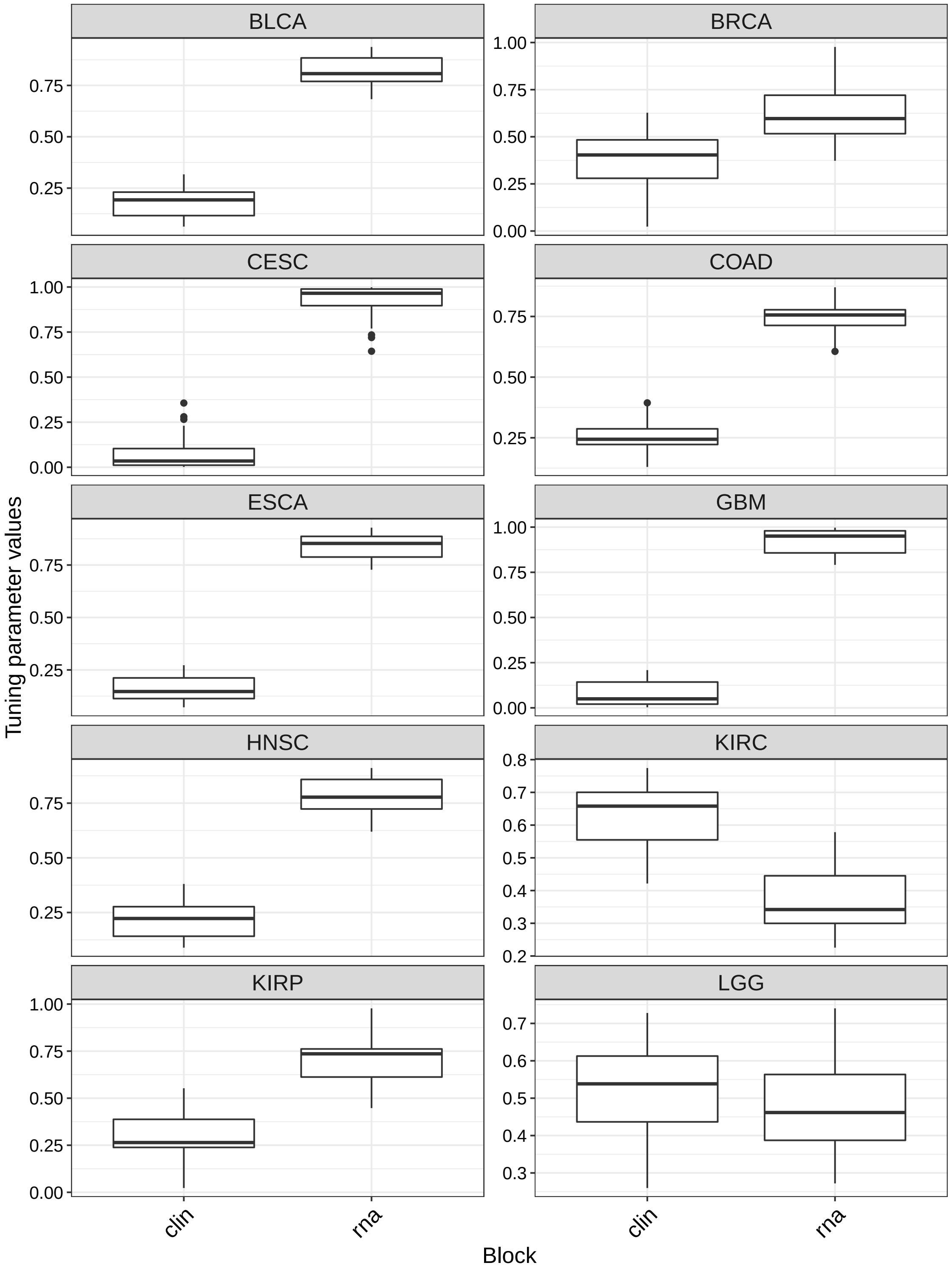

Fig. S24: Clinical covariates plus RNA measurements: b_m values optimized for variant RandomBlock – I

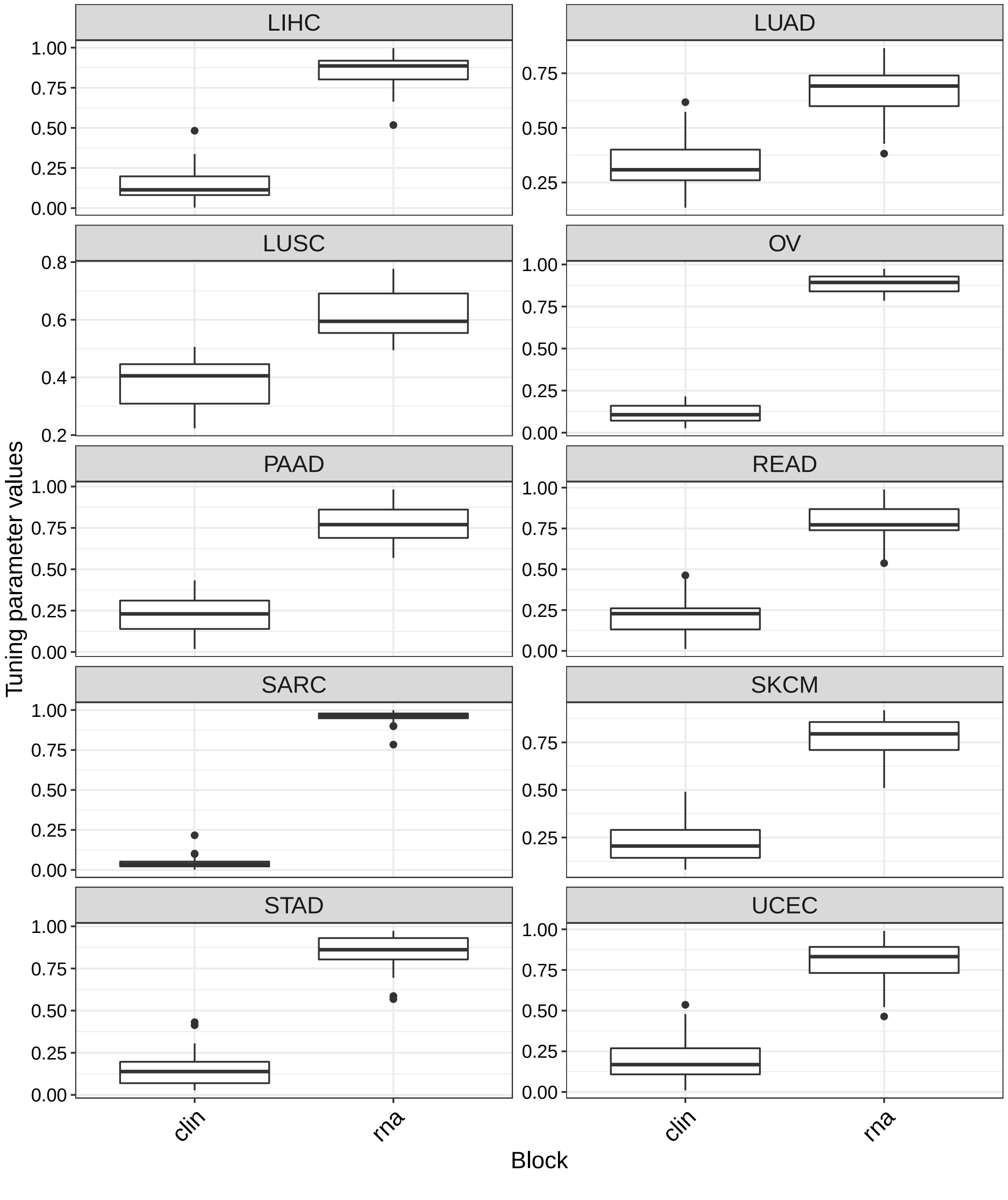

Fig. S25: Clinical covariates plus RNA measurements: b_m values optimized for variant RandomBlock – II

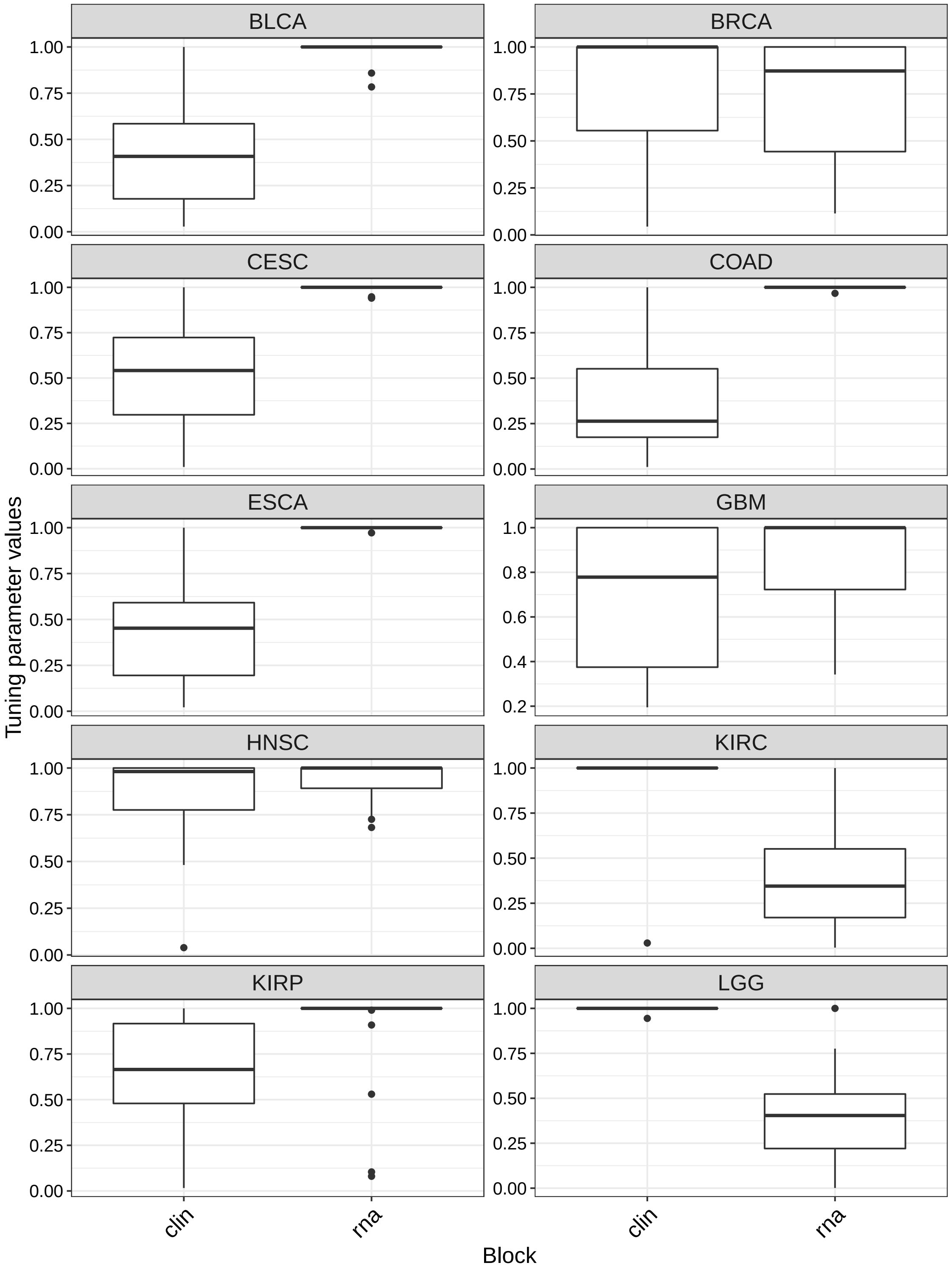

Fig. S26: Clinical covariates plus RNA measurements: w_m values optimized for variant BlockForest – I

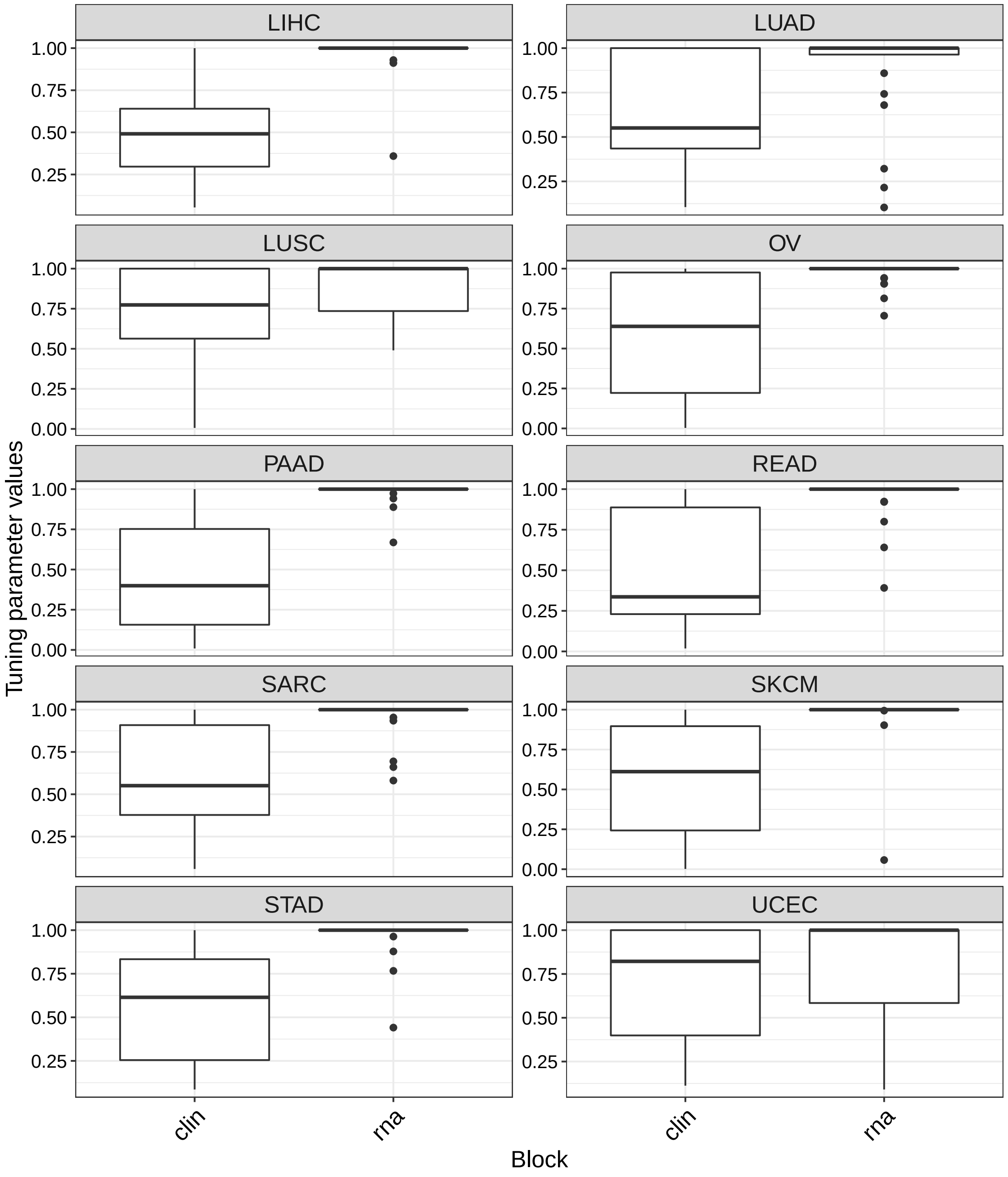

Fig. S27: Clinical covariates plus RNA measurements: w_m values optimized for variant BlockForest – II

H Clinical covariates plus mutation measurements: Performances of the ten considered methods

Figure S28 shows the results in the same form as in Figures 1 and 3 in the main paper. Note that this analysis was performed using 19 instead of 20 data sets, because the data set READ does not feature mutation data. For all ten prediction methods, the mean data set specific C index values are smaller than in the case of using the RNA block. We also performed a paired Student's t-test for each of the ten prediction methods to test for significant differences between the data set specific C index values obtained using the RNA block and those obtained using the mutation block. After adjustment for multiple testing with the Bonferroni-Holm method, for four of the ten methods the differences in performance were significant to the 5% significance level. Further notable differences to the case of using the RNA block are that the priority-Lasso and IPF-LASSO performed much better in relation to the other methods. BlockForest had the lowest mean rank 3.68 with respect to the data set specific C index values (where the latter did, however, not feature the lowest median rank as can be seen from Figure S28), followed by priority-Lasso with mean rank 3.74 and IPF-LASSO and RandomBlock with mean ranks 4.21 and 5.26, respectively. The ranks of BlockForest featured the lowest variance among these four methods. Again we used t-tests to test for superiority of the variants over RSF, adjusting for multiple testing using Bonferroni-Holm. Here, only BlockForest was significantly superior to RSF (adjusted p-value: 0.003). While the mean ranks of BlockForest and priority-Lasso are very similar, Figure S29 reveals that these two methods performed quite differently for many of the data sets.

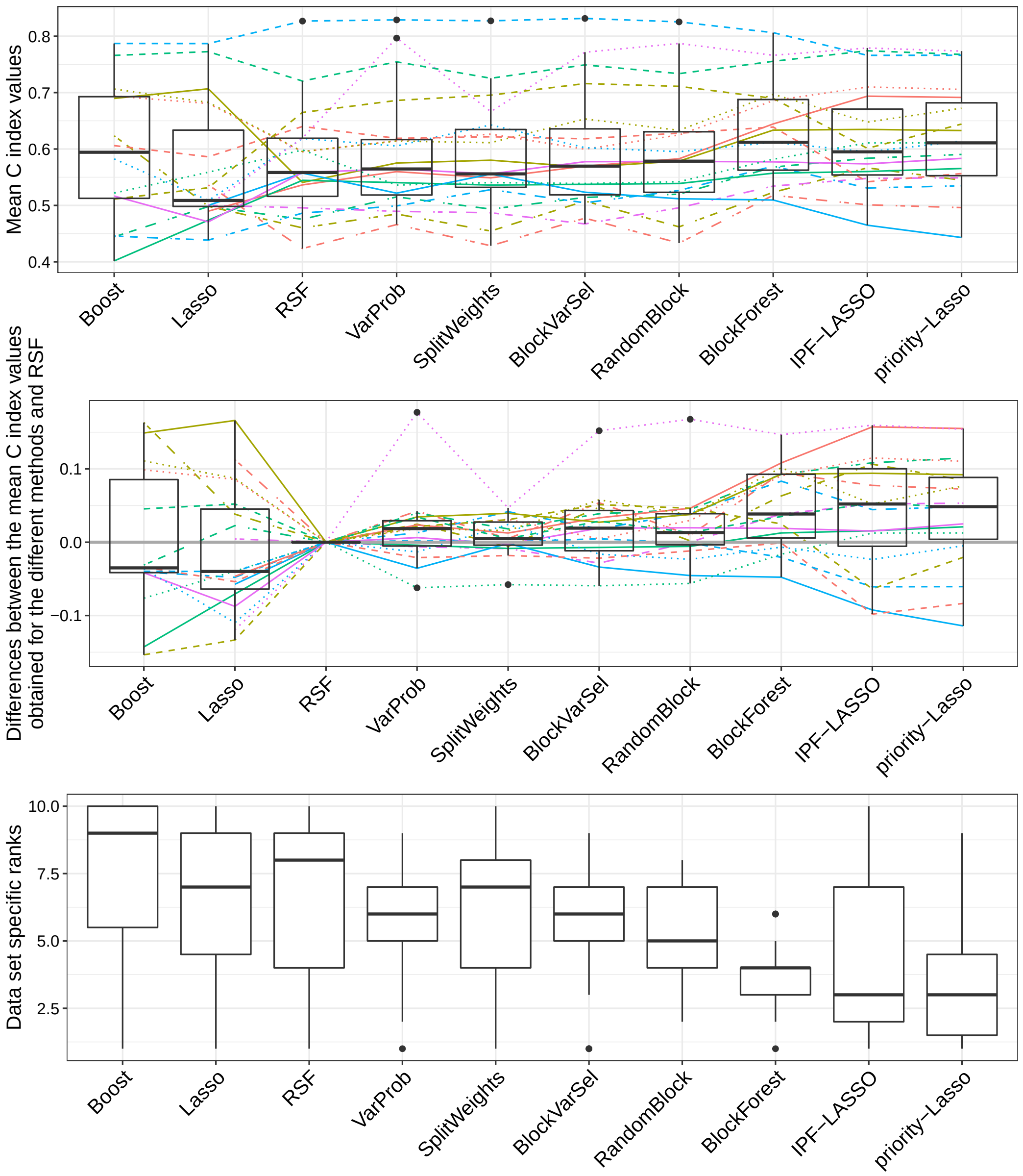

Fig. S28: Clinical covariates plus mutation measurements: Performances of the ten considered methods on the 19 data sets, for which measurements of the mutation block were available. Upper panel: Mean C index values for each data set and each of the ten methods considered. Middle panel: Differences between the mean C index values obtained using the different methods and that obtained using RSF. Lower panel: Data set specific ranks of each method among the other methods in terms of the mean C index values. The colored lines in the upper and middle panel connect the results obtained for the same data sets.

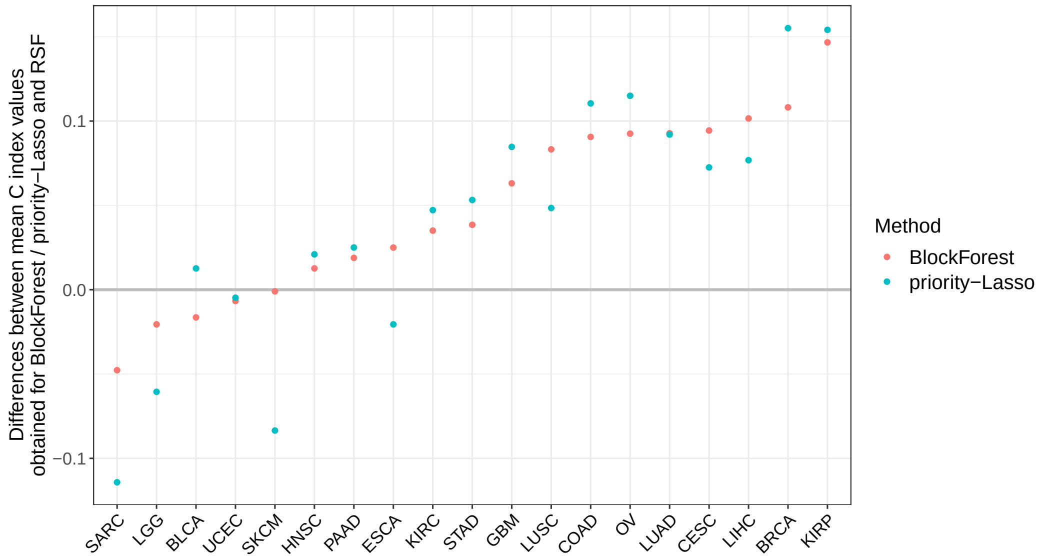

Fig. S29: Clinical covariates plus mutation measurements: Performances of BlockForest and priority-Lasso relative to that of RSF. Differences between the mean C index values obtained using BlockForest / priority-Lasso and that obtained using RSF, ordered by difference between the values obtained for BlockForest and RSF.

I Variations of the variants that prioritize the clinical covariates by including them mandatorily in the split point selection: Description of the variations

The following descriptions build upon the descriptions of the algorithms used for generating random sets of tuning parameter values in the cases of the standard variants, that is, those that do not prioritize the clinical covariates. See Section K of Additional file 1 for the latter descriptions.

In the cases of VarProb, SplitWeights, BlockVarSel, and BlockForest the sampling of the candidate variables and the tuning parameter optimization are performed as in the corresponding standard variants, except that all variables from the clinical block are always added to the sets of candidate variables.

In the case of RandomBlock all clinical covariates are also taken into the sets of candidate variables mandatorily. The candidate tuples of block selection probabilities are drawn as in the standard RandomBlock procedure, that is, drawing tuples of size M, disregarding the fact that only $M-1$ block selection probabilities are needed, since the clinical covariates are drawn mandatorily. With this procedure, the $M-1$ (optimized) block selection probabilities of the blocks different from the clinical block do not add up to one. As a result, it can happen that none of the $M-1$ blocks different from the clinical block are drawn. More precisely, this occurs with probability one minus the sum of the (optimized) block selection probabilities of the blocks different from the clinical block. This fact that it is possible that none of the $M-1$ blocks different from the clinical block are drawn becomes particularly important in the case of using only clinical covariates plus a single type of omics data. In this situation, if the block selection probabilities of the blocks different from the clinical block added up to one, the omics block would always be drawn. This is because when using only two blocks, there is only one block different from the clinical block, which would then be attributed a selection probability of one. By contrast, when drawing $M = 2$ selection probabilities, the omics block can, in principle, be attributed any selection probability from zero to one, where the optimized selection probability will depend on the levels of information contained in the omics block and in the clinical block.

J Variations of the variants that prioritize the clinical covariates by including them mandatorily in the split point selection: Results

In the following, the variations that include the clinical covariates mandatorily in the split point selection are designated using the names of the corresponding variants extended by the suffix "_clin".

Figure S30 shows the results for the multi-omics data in the same form as in the cases of the main analyses. For each variant, including the clinical covariates mandatorily improved the prediction results, these improvements are the strongest in the cases of RandomBlock and SplitWeights. In the multi-omics case, the following mean ranks for the methods were obtained (from best to worst): 2.70 (RandomBlock_clin), 4.55 (BlockForest_clin), 5.35 (BlockForest), 6.35 (RandomBlock), 7.15 (VarProb_clin), 7.35 (BlockVarSel_clin), 8.00 (SplitWeights_clin), 8.55 (IPF-LASSO), 8.70 (RSF), 9.00 (Boost), 9.95 (BlockVarSel), 10.10 (VarProb), 10.50 (Lasso), 10.75 (SplitWeights), 11.00 (priority-Lasso). The mean rank of RandomBlock_clin is substantially smaller than that of the other methods. Excluding the three data sets for which only four of the five blocks were available did not change these results notably (see Figure S31). For seven of the twenty datasets the improvement of the best performing method RandomBlock_clin over RSF was greater than 0.05 in terms of absolute difference (see Figure S32), while for BlockForest this was the case for only four data sets (see Figure **??** in the main paper).

Also in the case of using only the clinical block and the RNA block, including the clinical covariates mandatorily improved the prediction results for each variant (see Figure S33). However, the improvement of RandomBlock_clin over RandomBlock was less strong than in the multi-omics case. We obtained the following mean ranks (from best to worst): 3.45 (BlockForest_clin), 4.80 (BlockForest), 5.10 (RandomBlock_clin), 5.35 (VarProb_clin), 6.65 (BlockVarSel_clin), 7.70 (VarProb), 7.80 (RandomBlock), 7.90 (SplitWeights_clin), 8.50 (BlockVarSel), 8.95 (priority-Lasso), 9.75 (RSF), 10.20 (SplitWeights), 10.30 (IPF-LASSO), 10.85 (Boost), 12.70 (Lasso). While BlockForest_clin thus had the lowest rank, it does not set itself apart as strongly from the second best method (BlockForest) as RandomBlock_clin did in the multi-omics case. While both BlockForest_clin and BlockForest outperformed RSF by more than 0.05 in terms of absolute difference for the same five data sets, the improvements obtained for these data sets are (slightly) stronger in the case of BlockForest_clin (see Figure S34).

For the analysis in which we used the clinical block and the mutation block, the variations that include the clinical covariates mandatorily did not all perform better than the corresponding original variants (see Figure S35). However, we do observe considerable improvements here in the cases of VarProb and RandomBlock. The mean ranks were (from best to worst): 5.21 (BlockForest_clin), 5.47 (BlockForest), 5.74 (priority-Lasso), 5.84 (RandomBlock_clin), 6.11 (VarProb_clin), 6.37 (IPF-LASSO), 8.21 (RandomBlock), 9.00 (BlockVarSel), 9.05 (SplitWeights_clin), 9.16 (VarProb), 9.26 (BlockVarSel_clin), 9.47 (SplitWeights), 9.89 (RSF), 10.00 (Lasso), 11.21 (Boost). Note that the mean ranks differ to a considerably lesser degree from each other than they did when using the RNA block instead of the mutation block. The mean ranks of the best methods hardly differ form each other at all. The reason that the variability of the mean ranks is smaller in the case of using

the mutation block is probably that the mean C index values have higher variances here, leading to less clear rankings of the methods. The higher variances of the mean C index values can probably be explained by the fact that the variables in the mutation block are binary as opposed to metric for the other blocks, that is, they contain less concise information, making the estimation more variable. Note that priority-Lasso is among the best methods here. IPF-LASSO also performs relatively well. Figure S36 reveals that the method that performed best according to the mean ranks, BlockForest_clin was, for 10 of the 19 data sets (the mutation block was missing for one data set), better than RSF by more than 0.05 in terms of absolute difference.

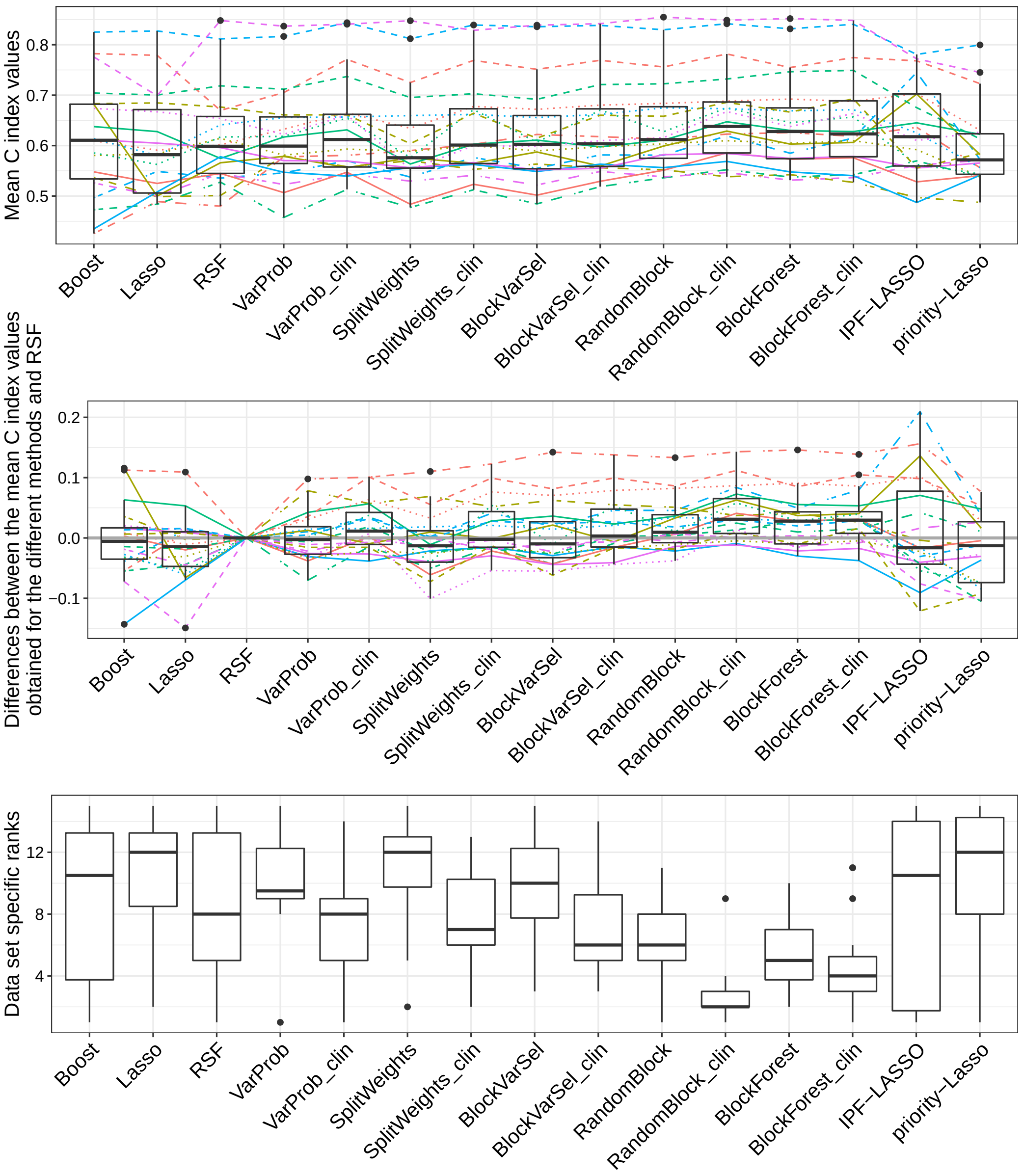

Fig. S30: Multi-omics data: Performances of all 15 considered methods including the variations of the variants that include the clinical covariates mandatorily in the split point selection. Upper panel: Mean C index values for each data set and each of the 15 methods considered. Middle panel: Differences between the mean C index values obtained using the different methods and that obtained using RSF. Lower panel: Data set specific ranks of each method among the other methods in terms of the mean C index values. The colored lines in the upper and middle panel connect the results obtained for the same data sets.

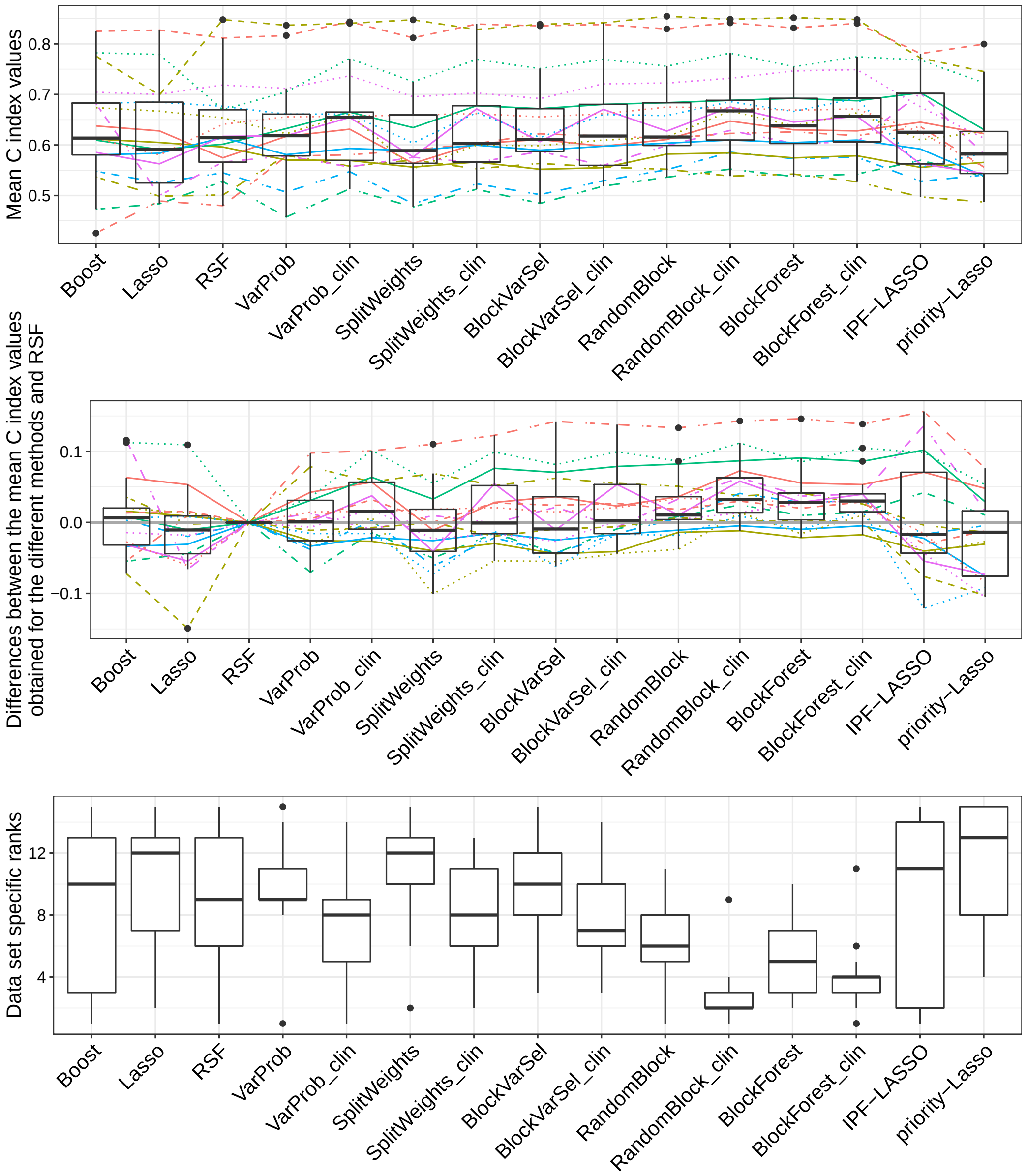

Fig. S31: Multi-omics data: Performances of all 15 considered methods including the variations of the variants that include the clinical covariates mandatorily in the split point selection; excluding the three data sets for which not all five blocks were available. Upper panel: Mean C index values for each data set and each of the 15 methods considered. Middle panel: Differences between the mean C index values obtained using the different methods and that obtained using RSF. Lower panel: Data set specific ranks of each method among the other methods in terms of the mean C index values. The colored lines in the upper and middle panel connect the results obtained for the same data sets.

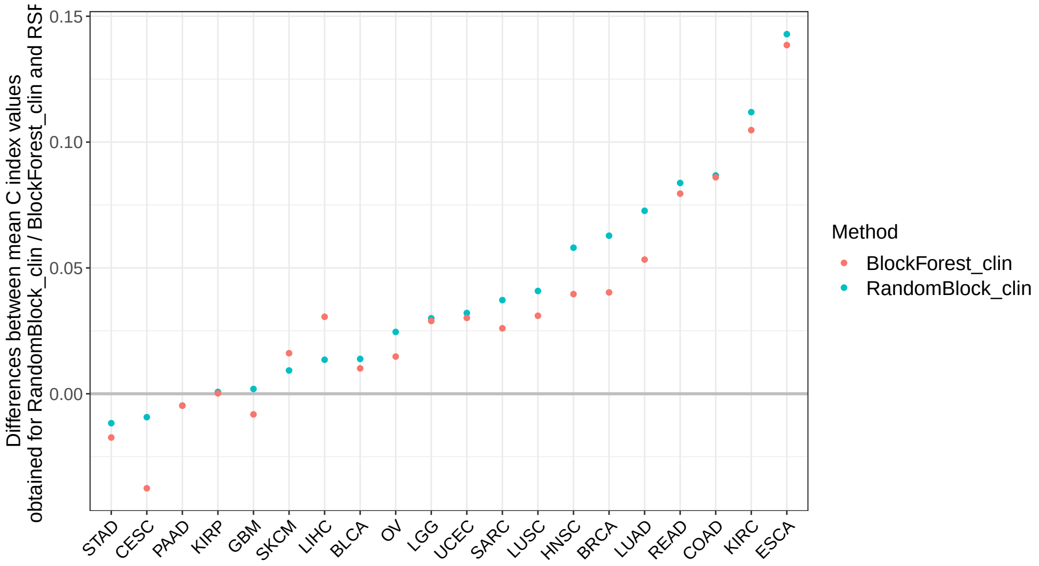

Fig. S32: Multi-omics data: Performances of RandomBlock_clin and BlockForest_clin relative to that of RSF. Differences between the mean C index values obtained using RandomBlock_clin / BlockForest_clin and that obtained using RSF, ordered by difference between the values obtained for RandomBlock_clin and RSF.

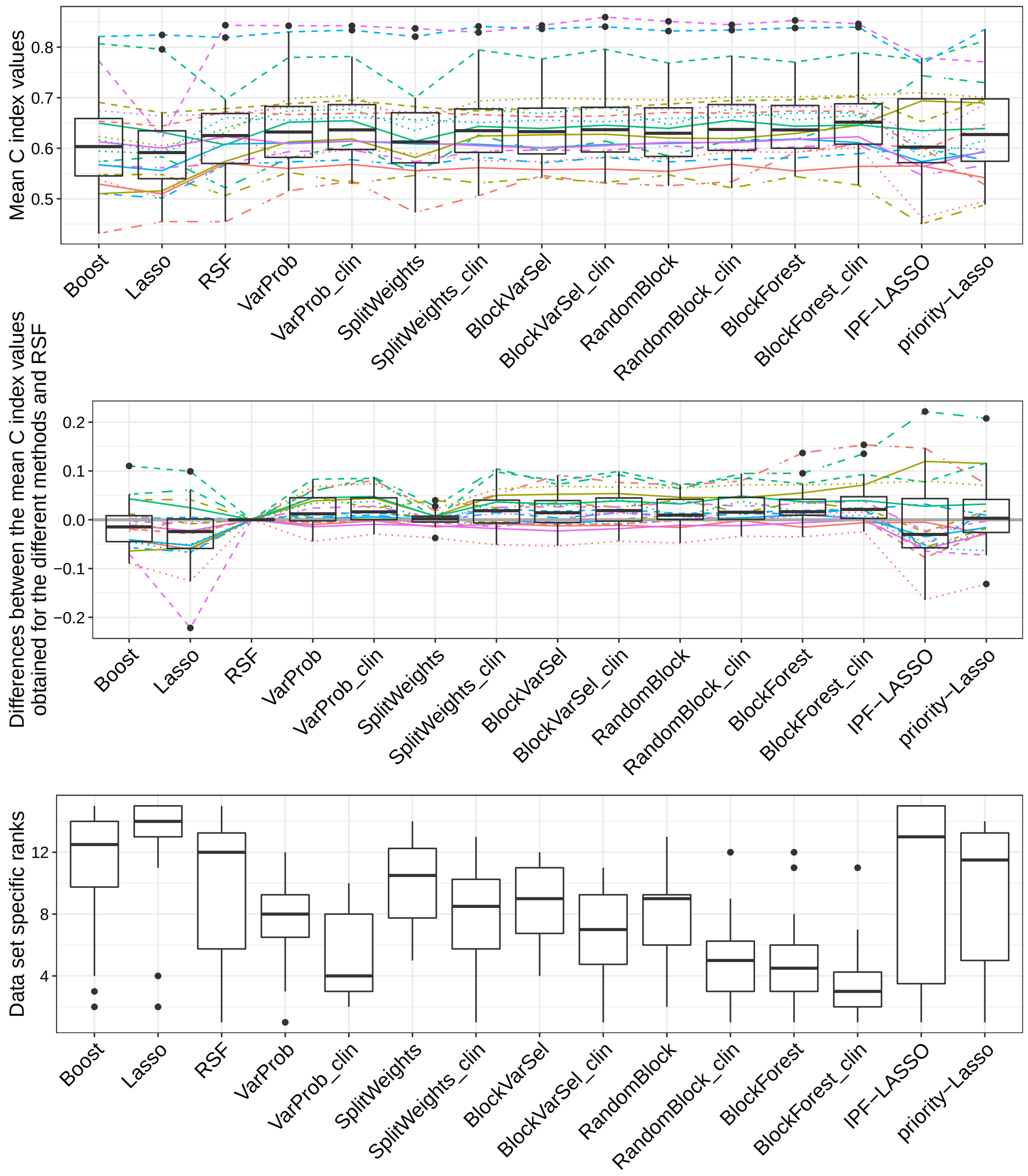

Fig. S33: Clinical covariates plus RNA measurements: Performances of all 15 considered methods including the variations of the variants that include the clinical covariates mandatorily in the split point selection. Upper panel: Mean C index values for each data set and each of the 15 methods considered. Middle panel: Differences between the mean C index values obtained using the different methods and that obtained using RSF. Lower panel: Data set specific ranks of each method among the other methods in terms of the mean C index values. The colored lines in the upper and middle panel connect the results obtained for the same data sets.

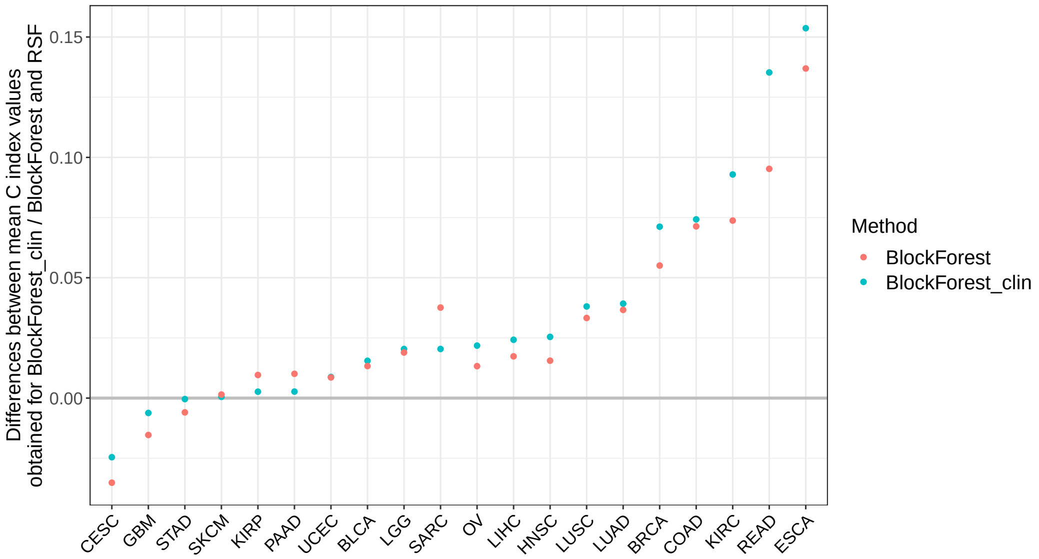

Fig. S34: Clinical covariates plus RNA measurements: Performances of BlockForest_clin and BlockForest relative to that of RSF. Differences between the mean C index values obtained using BlockForest_clin / BlockForest and that obtained using RSF, ordered by difference between the values obtained for BlockForest_clin and RSF.

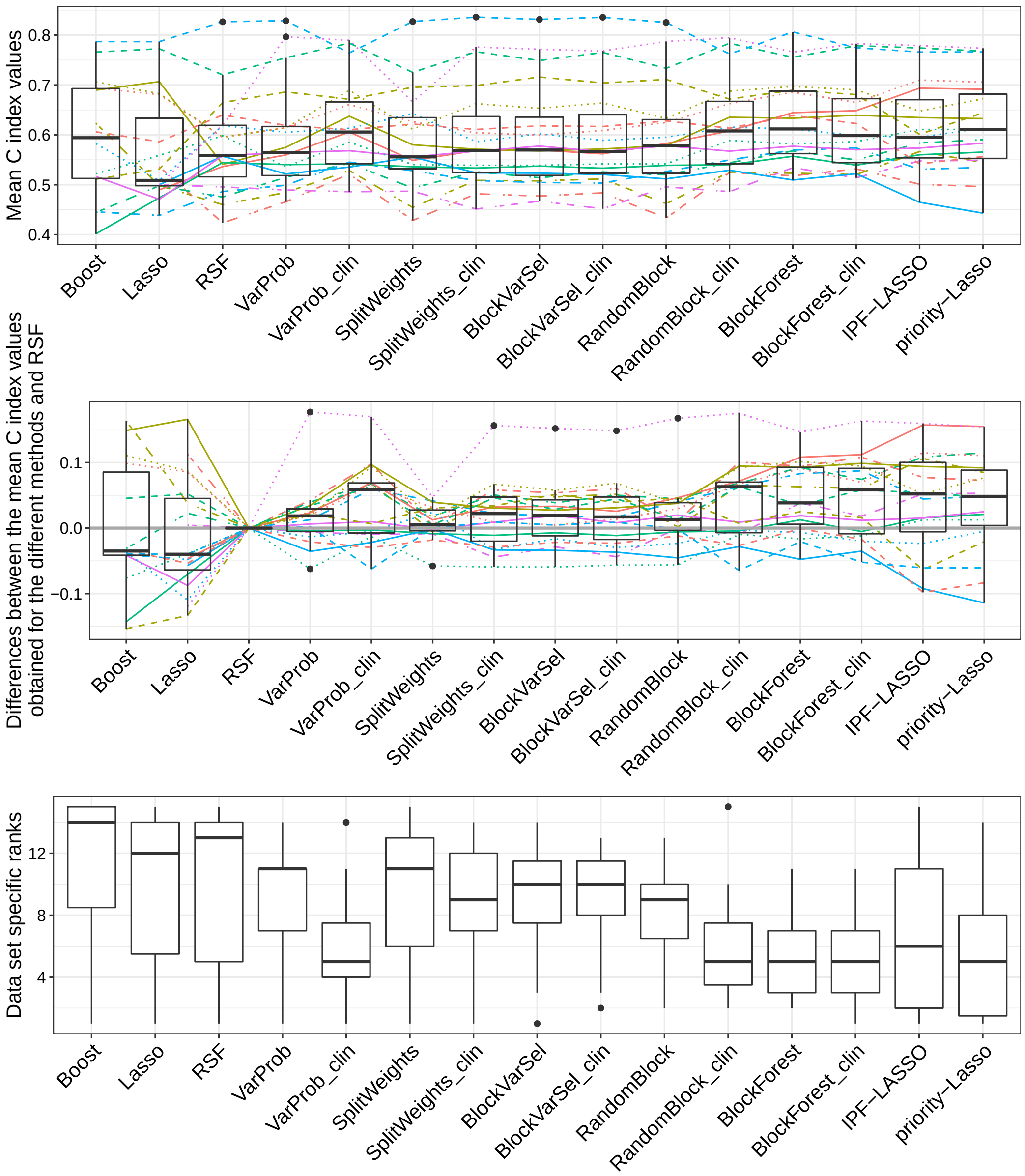

Fig. S35: Clinical covariates plus mutation measurements: Performances of all 15 considered methods including the variations of the variants that include the clinical covariates mandatorily in the split point selection. Upper panel: Mean C index values for each data set and each of the 15 methods considered. Middle panel: Differences between the mean C index values obtained using the different methods and that obtained using RSF. Lower panel: Data set specific ranks of each method among the other methods in terms of the mean C index values. The colored lines in the upper and middle panel connect the results obtained for the same data sets.

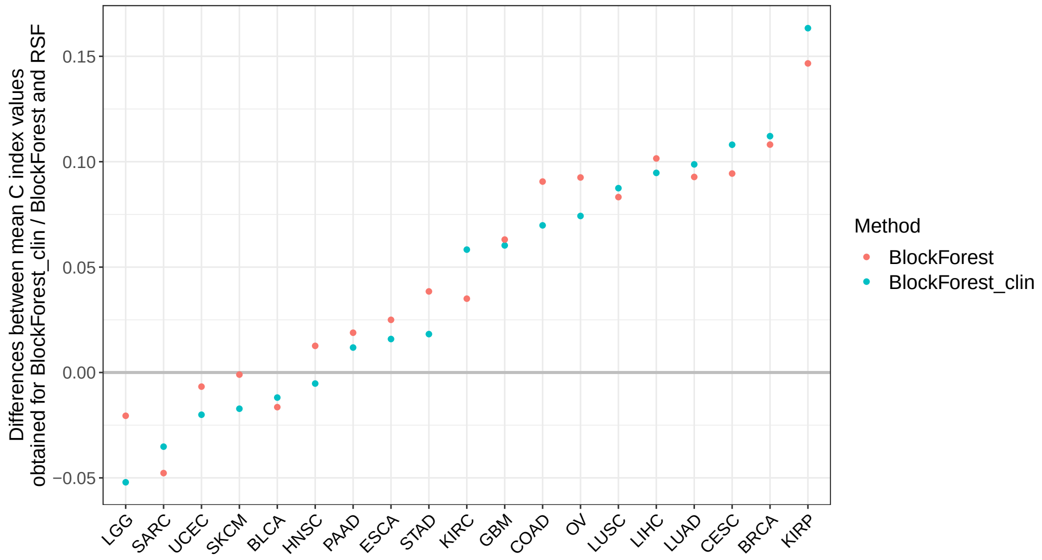

Fig. S36: Clinical covariates plus mutation measurements: Performances of BlockForest_clin and BlockForest relative to that of RSF. Differences between the mean C index values obtained using BlockForest_clin / BlockForest and that obtained using RSF, ordered by difference between the values obtained for BlockForest_clin and RSF.

K Algorithms used for generating random sets of tuning parameter values in the tuning parameter value optimization

A random set of values $\mathcal{S}_{it,1}, \ldots, \mathcal{S}_{it,M}$ for the tuning parameters $w_1, \ldots, w_M$ used by SplitWeights, BlockVarSel, and BlockForest can be obtained as follows:

1. Draw $M-1$ values $a_1, \ldots, a_{M-1}$ from the uniform distribution U(0,1) and set $a_M := 1$.
2. Permute the values $a_1, \ldots, a_M$ randomly.

The procedure VarProb features the tuning parameters $v_1, \ldots, v_M$, for which we generate a random set $\mathcal{S}_{it,1}, \ldots, \mathcal{S}_{it,M}$ of values in the following way:

1. For $m = 1, \ldots, M$:
   (a) Draw u from the uniform distribution U(0,1).
   (b) If $u < 0.5$, draw a value $\mathcal{S}_{unst,it,m}$ from the uniform distribution U(0, $\sqrt{p_m}/p_m$) and if $u \geq 0.5$ draw a value $\mathcal{S}_{unst,it,m}$ from the uniform distribution U($\sqrt{p_m}/p_m$, 1).
2. Standardize the $\mathcal{S}_{unst,it,m}$ values by dividing each value by $\sum_{m=1}^{M} p_m \mathcal{S}_{unst,it,m}$ to ensure that $\sum_{m=1}^{M} p_m \mathcal{S}_{it,m} = 1$.

The reason why the $\mathcal{S}_{unst,it,m}$ values are centered about $\sqrt{p_m}/p_m$ (step 1(b)) is that if $v_m = (\sqrt{p_m}/p_m)/\sum_{m^*=1}^{M} p_{m^*}\sqrt{p_{m^*}}/p_{m^*}$, on average there would be $\sqrt{p_m}$ values drawn from block m given that $\sum_{m=1}^{M} \sqrt{p_m}$ variables are sampled in total. Sampling $\sqrt{p_m}$ covariates per block is performed by the procedures BlockVarSel, RandomBlock, and BlockForest.

Lastly, the procedure RandomBlock uses the tuning parameter values $b_1, \ldots, b_M$. We generate a random set $\mathcal{S}_{it,1}, \ldots, \mathcal{S}_{it,M}$ of these values as follows:

1. Draw $M-1$ values $a_1, \ldots, a_{M-1}$ from the uniform distribution U(0,1).
2. Sort $a_1, \ldots, a_{M-1}$ and denote the sorted sequence by $a_1^*, \ldots, a_{M-1}^*$.
3. Calculate the values $\mathcal{S}_{it,1}, \ldots, \mathcal{S}_{it,M}$ through $a_1^*, a_2^* - a_1^*, \ldots, a_{M-1}^* - a_{M-2}^*, 1 - a_{M-1}^*$.

L Overview of the data sets used in the comparison study

Table S1: Detailed overview of the data sets used in the comparison study. The following information is given: data set label, the numbers of covariates per block, where '#' indicates the cardinality, the sample size and the percentage of observations for which the survival time was uncensored.

data set label	# clinical	# CNV	# miRNA	# mutation	# RNA	Sample size	% of uncensored observations
BLCA	4	57964	825	18650	23081	310	32 %
BRCA	8	57964	835	18847	22694	863	9 %
CESC	4	57447	–	18998	22398	206	15 %
COAD	5	57964	802	19786	22210	350	22 %
ESCA	4	57964	763	15162	25494	121	21 %
GBM	3	57964	–	17106	23288	154	73 %
HNSC	5	57964	793	17840	21520	411	35 %
KIRC	6	57964	725	12017	22972	322	22 %
KIRP	4	57964	593	11610	32525	249	10 %
LGG	3	57964	645	13389	22297	454	21 %
LIHC	4	57964	776	15924	20994	298	28 %
LUAD	6	57964	799	18966	23681	424	30 %
LUSC	7	57964	895	18832	23524	365	39 %
OV	2	57447	975	16837	24508	261	54 %
PAAD	4	57964	612	12882	22348	142	49 %
PRAD	4	57925	585	12416	21769	425	2 %
READ	5	57964	769	–	21896	138	16 %
SARC	2	57964	778	12478	22842	183	16 %
SKCM	5	57964	1002	19488	22248	264	25 %
STAD	6	57967	787	19141	26027	284	27 %
UCEC	3	57447	866	21226	23978	503	13 %

References

[1] Kandoth C, McLellan MD, Vandin F, Ye K, Niu B, Lu C, et al. Mutational landscape and significance across 12 major cancer types. Nature. 2013;502:333–339.

[2] Zhang W, Yu Y, Hertwig F, Thierry-Mieg J, Zhang W, Thierry-Mieg D, et al. Comparison of RNA-seq and microarray-based models for clinical endpoint prediction. Genome Biol. 2015;16:133.
