# Supplementary figures and images for: Block Forests: random forests for blocks of clinical and omics covariate data
Source: BMC Bioinformatics. 2019 Jun 27;20:358. doi: 10.1186/s12859-019-2942-y (PMC6598279; doi:10.1186/s12859-019-2942-y)

Influence of 'n'

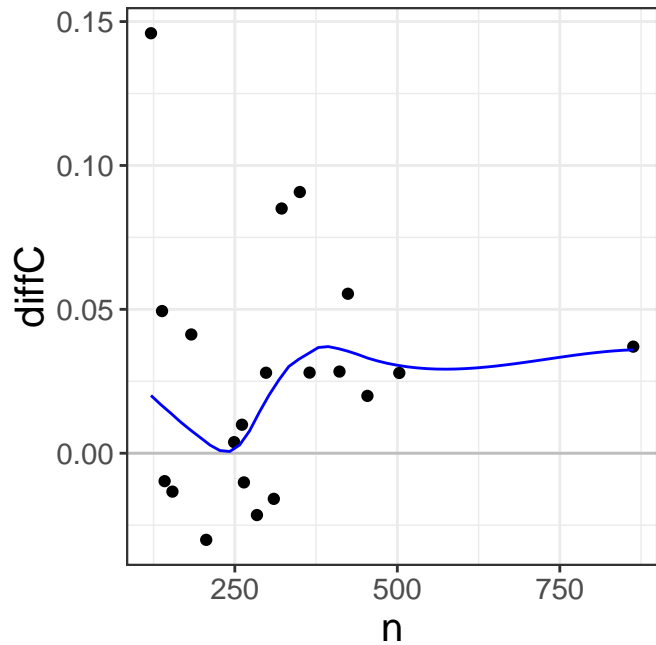

Influence of 'oneblockimp'

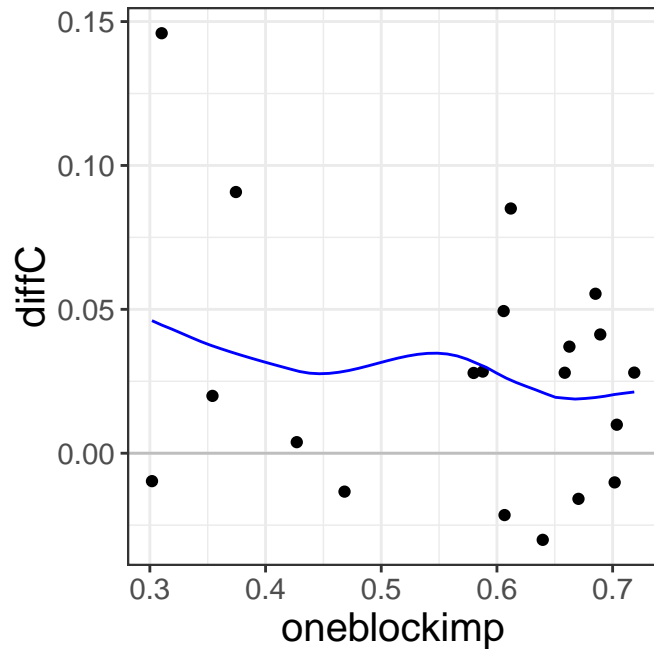

Influence of 'signal'

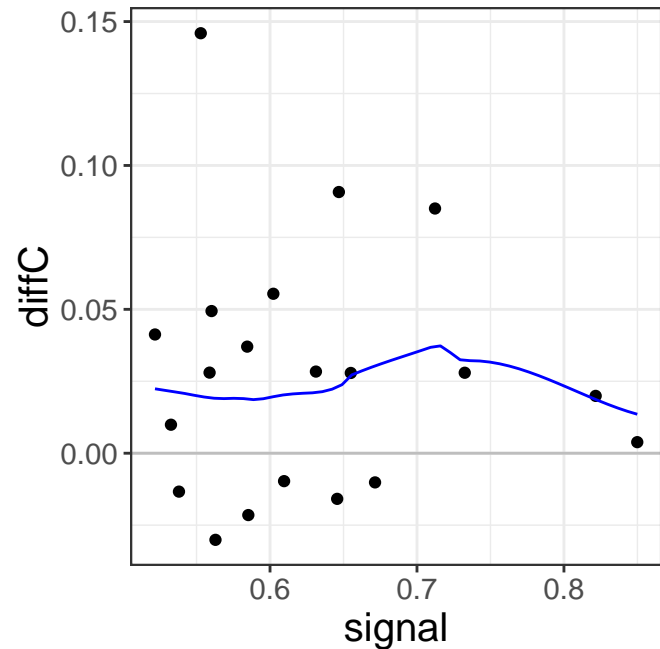

Supplement: Supplementary file 2 — Electronic Appendix. This folder contains all R Code written to perform the analyses presented in this paper and in Additional file 1 as well as Rda files enabling fast evaluation of the results. (ZIP 26,855 kb) [file 12859_2019_2942_MOESM2_ESM.zip › Additional_file_2_HornungWright/Figures/FactorsDifferenceInPerformance.pdf]

Influence of 'n'

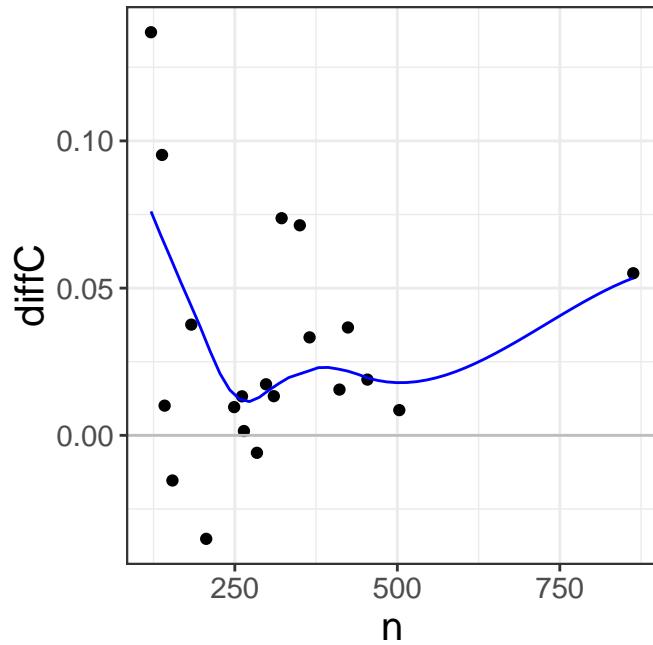Influence of ' $b_{\text{clin}}$ '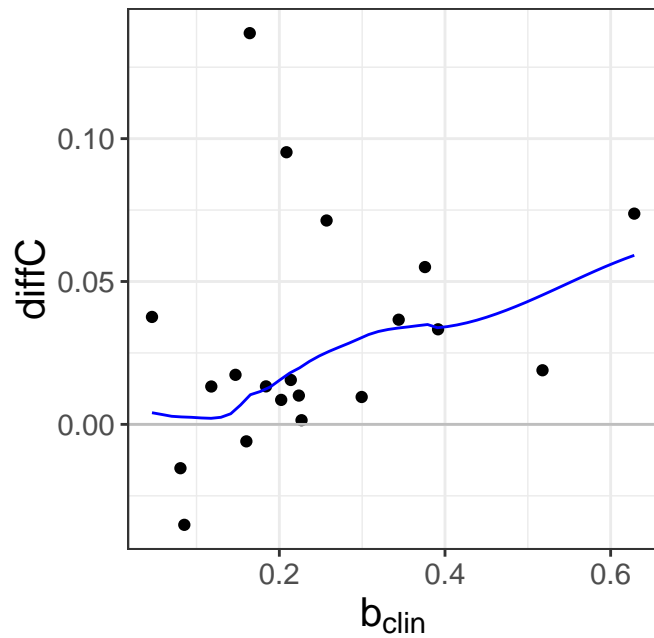

Influence of 'signal'

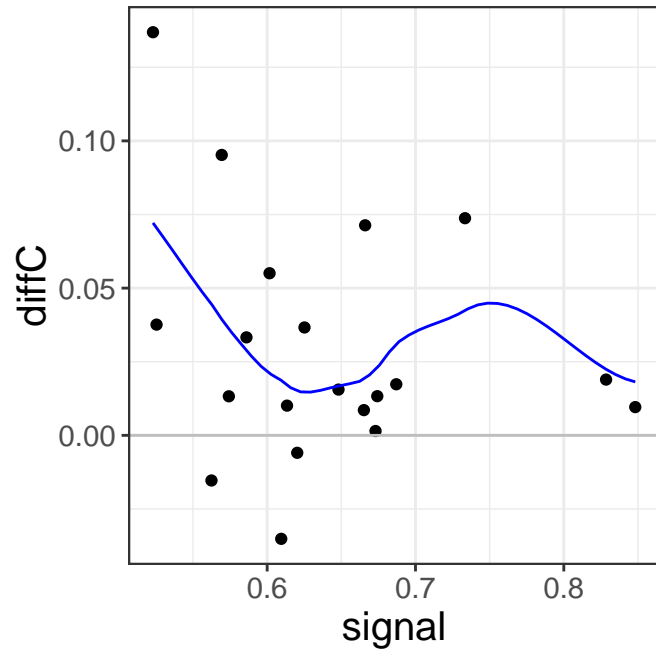

Supplement: Supplementary file 2 — Electronic Appendix. This folder contains all R Code written to perform the analyses presented in this paper and in Additional file 1 as well as Rda files enabling fast evaluation of the results. (ZIP 26,855 kb) [file 12859_2019_2942_MOESM2_ESM.zip › Additional_file_2_HornungWright/Figures/FactorsDifferenceInPerformanceTwoBlocks.pdf]

**a**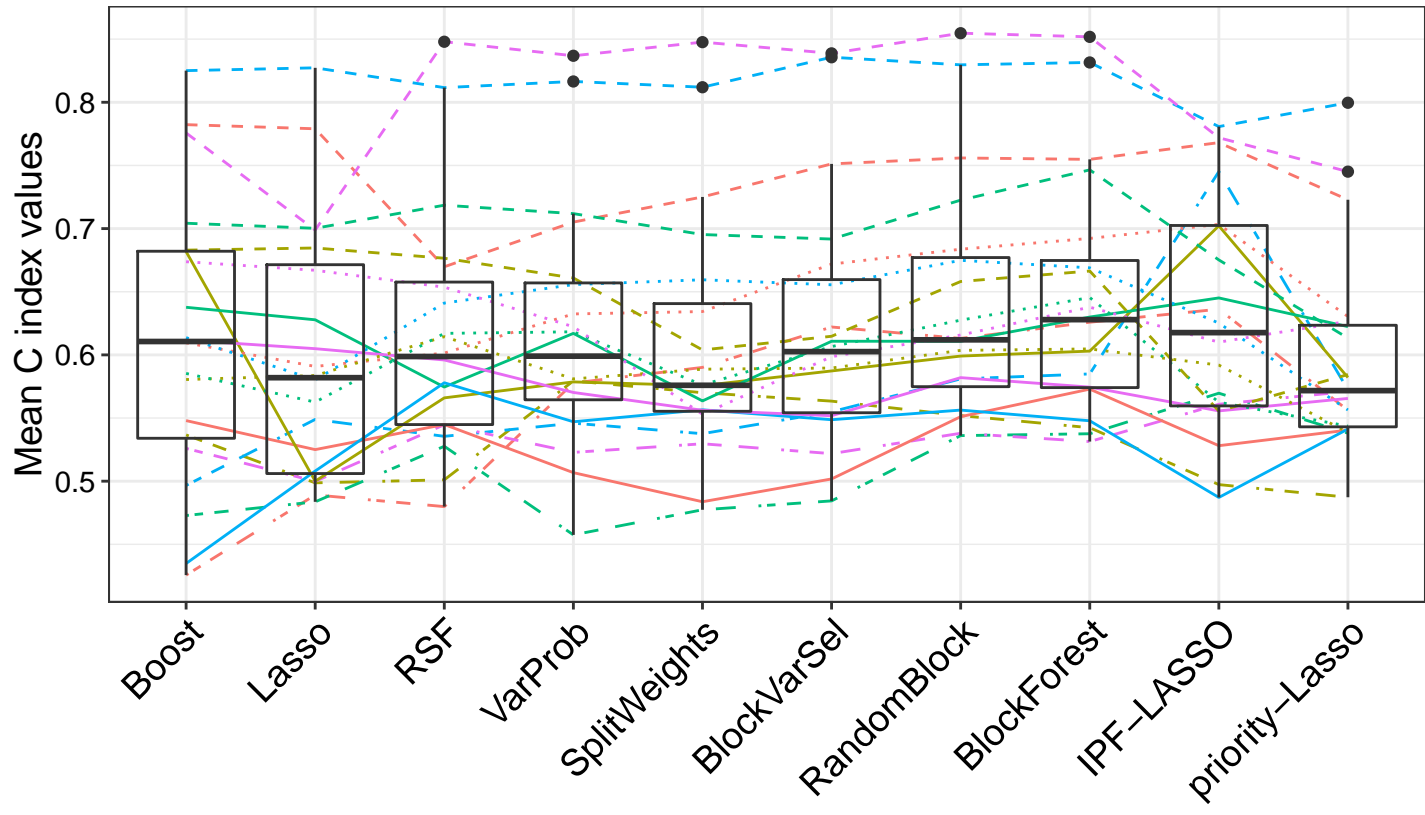**b**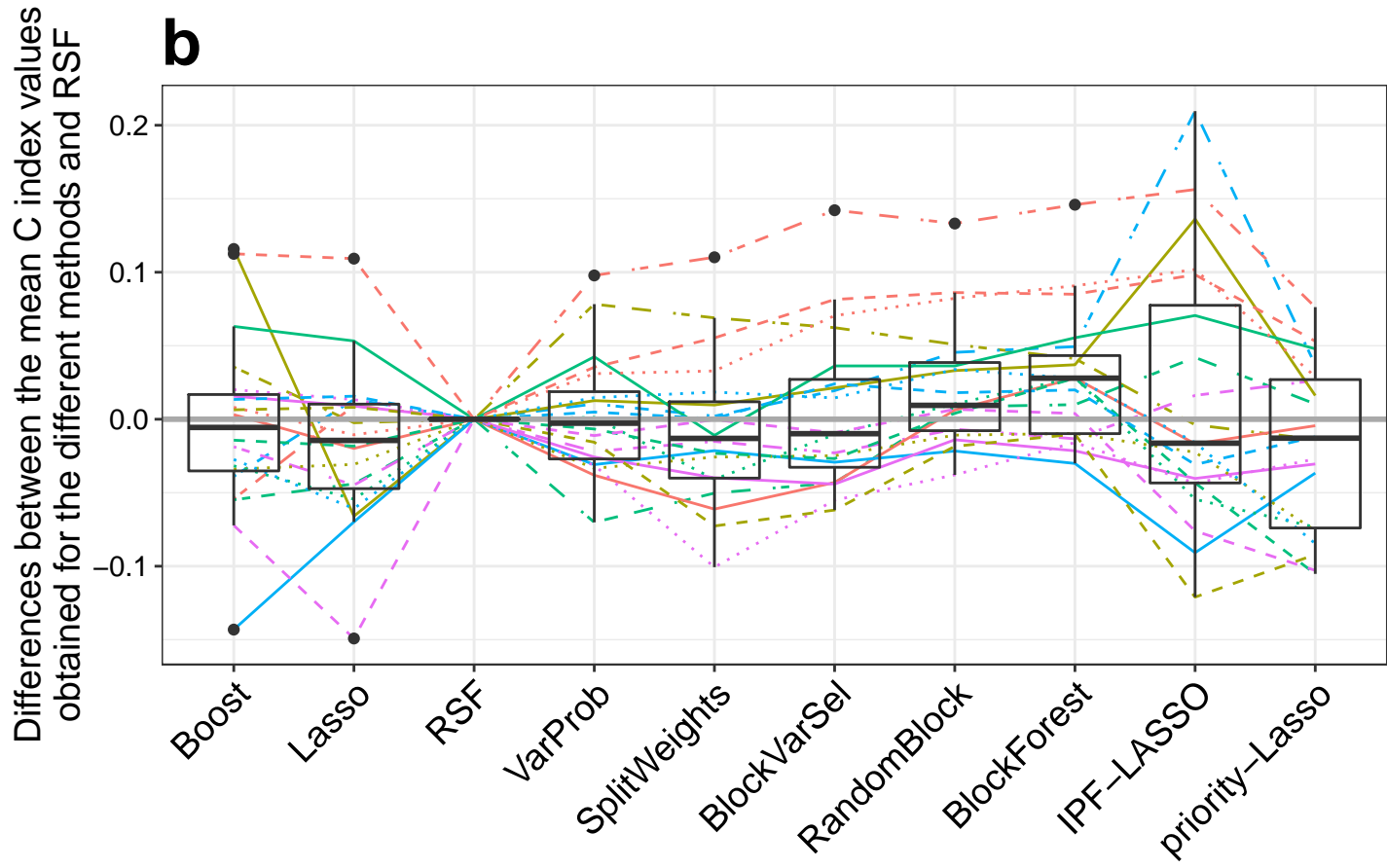**c**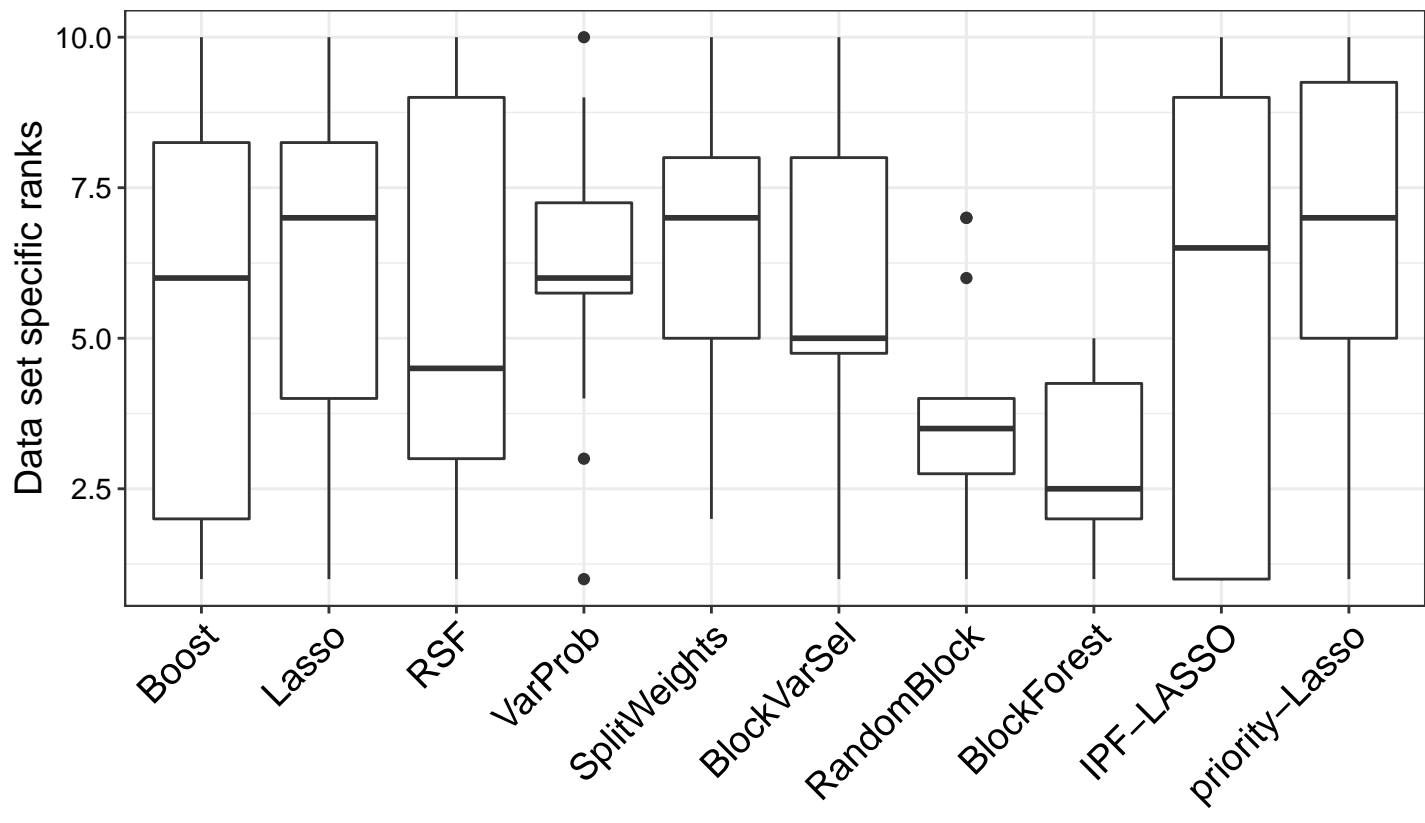

Supplement: Supplementary file 2 — Electronic Appendix. This folder contains all R Code written to perform the analyses presented in this paper and in Additional file 1 as well as Rda files enabling fast evaluation of the results. (ZIP 26,855 kb) [file 12859_2019_2942_MOESM2_ESM.zip › Additional_file_2_HornungWright/Figures/Figure1.pdf]

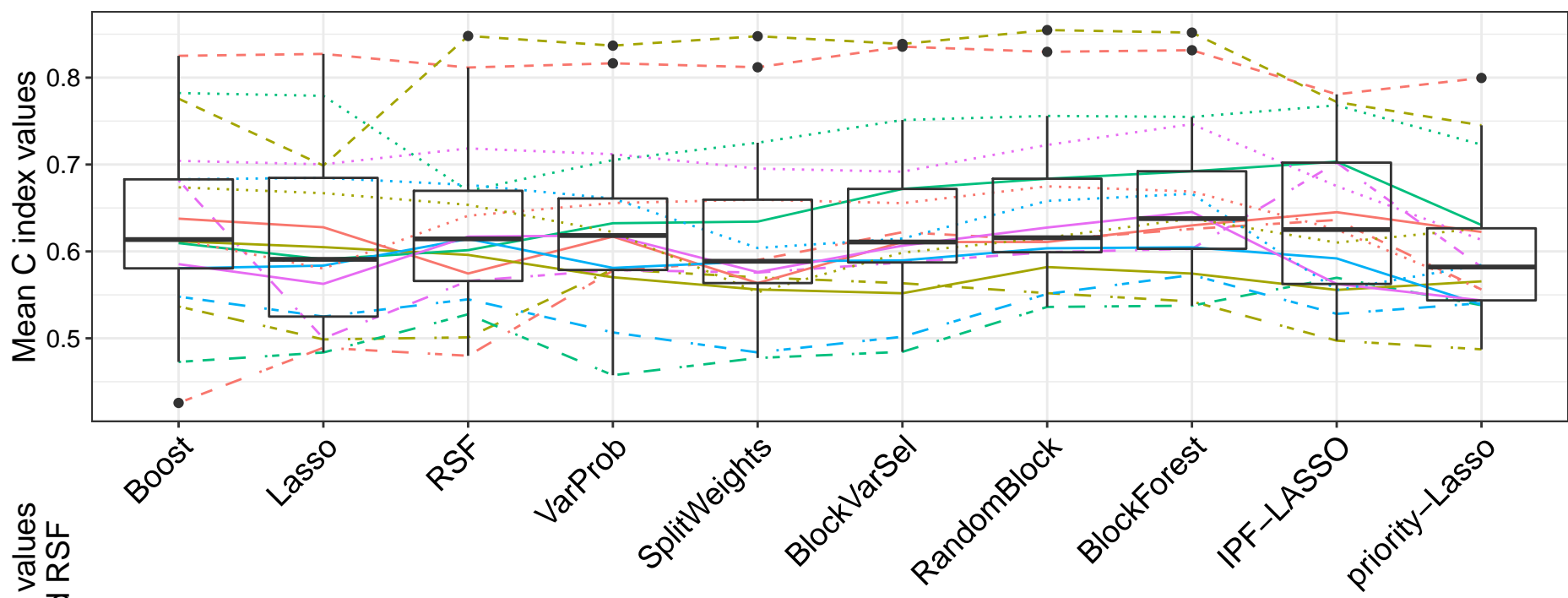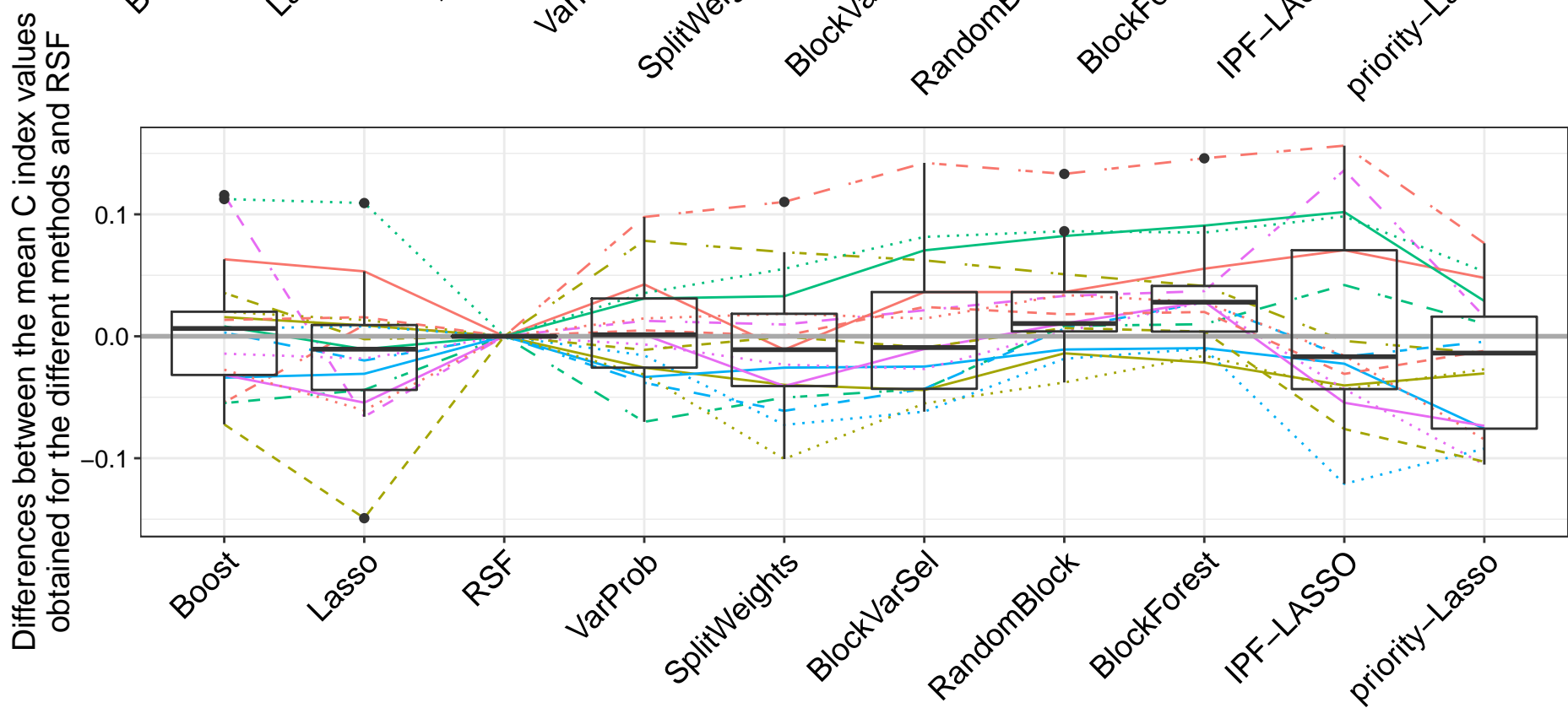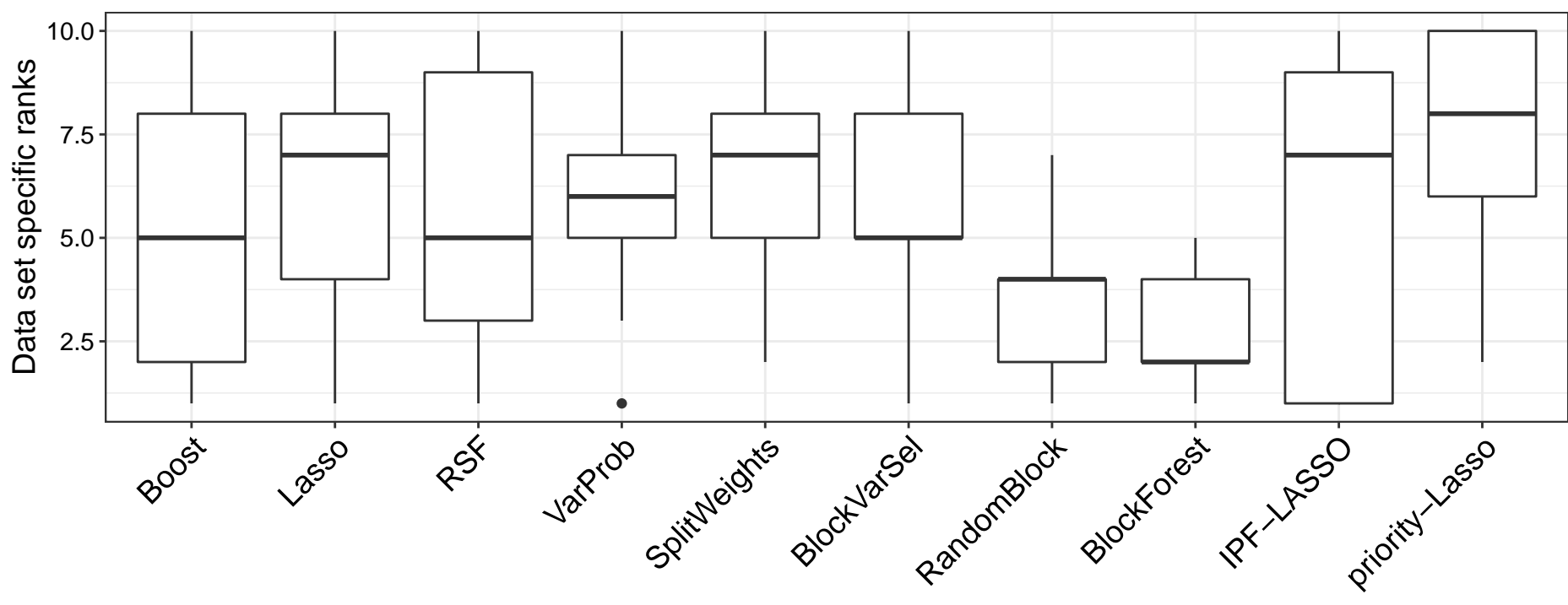

Supplement: Supplementary file 2 — Electronic Appendix. This folder contains all R Code written to perform the analyses presented in this paper and in Additional file 1 as well as Rda files enabling fast evaluation of the results. (ZIP 26,855 kb) [file 12859_2019_2942_MOESM2_ESM.zip › Additional_file_2_HornungWright/Figures/Figure1_without_CESC_GBM_READ.pdf]

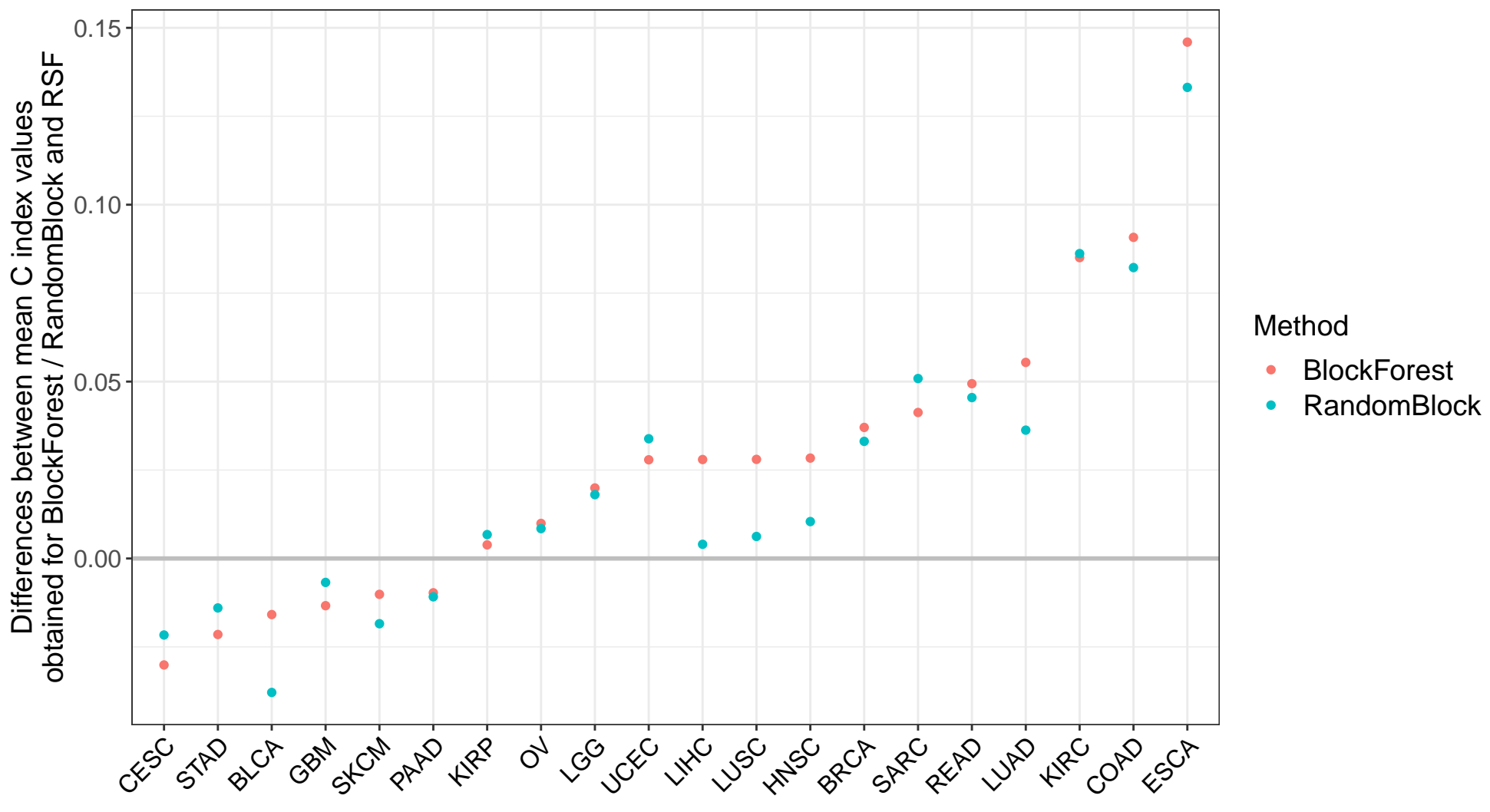

Supplement: Supplementary file 2 — Electronic Appendix. This folder contains all R Code written to perform the analyses presented in this paper and in Additional file 1 as well as Rda files enabling fast evaluation of the results. (ZIP 26,855 kb) [file 12859_2019_2942_MOESM2_ESM.zip › Additional_file_2_HornungWright/Figures/Figure2.pdf]

**a**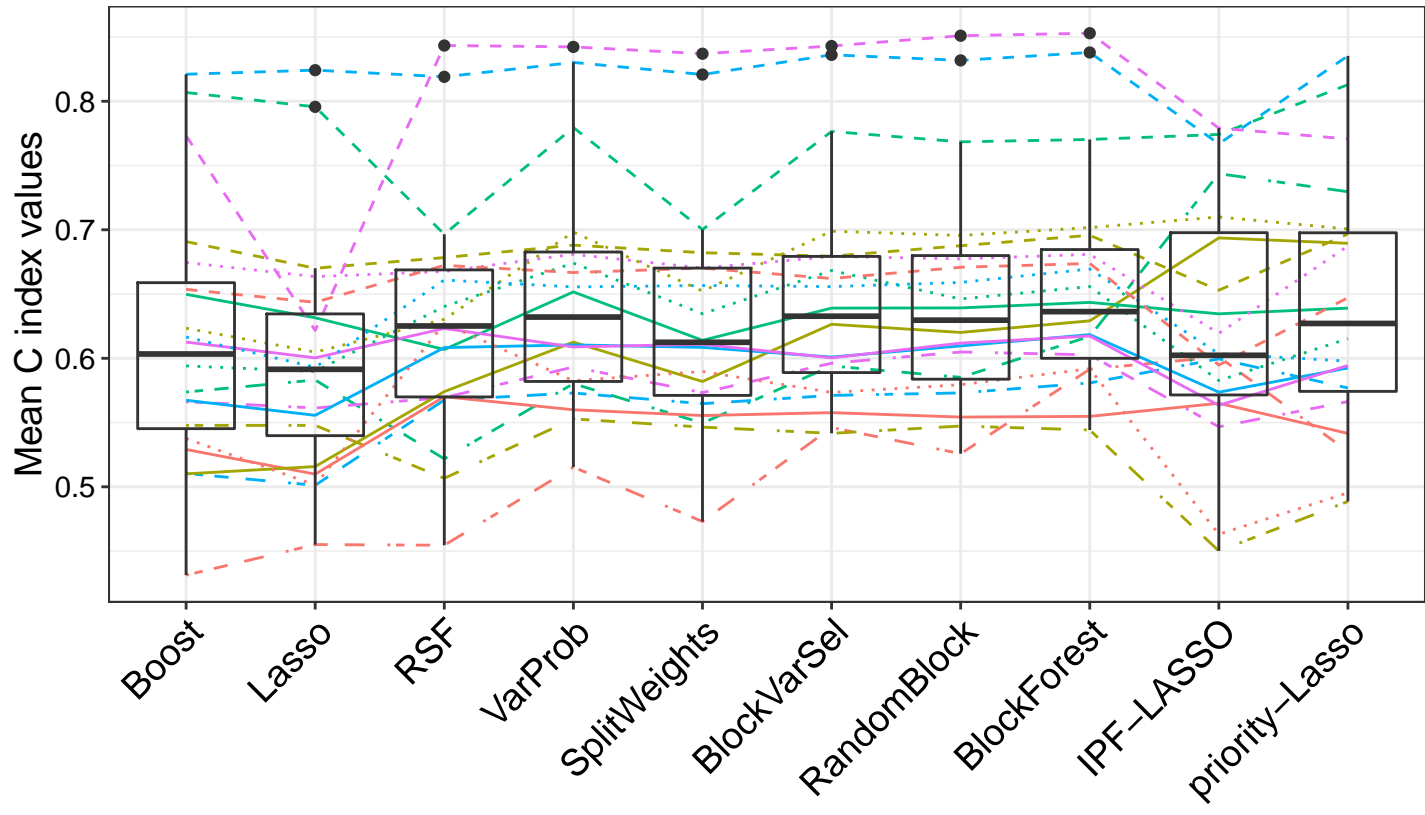**b**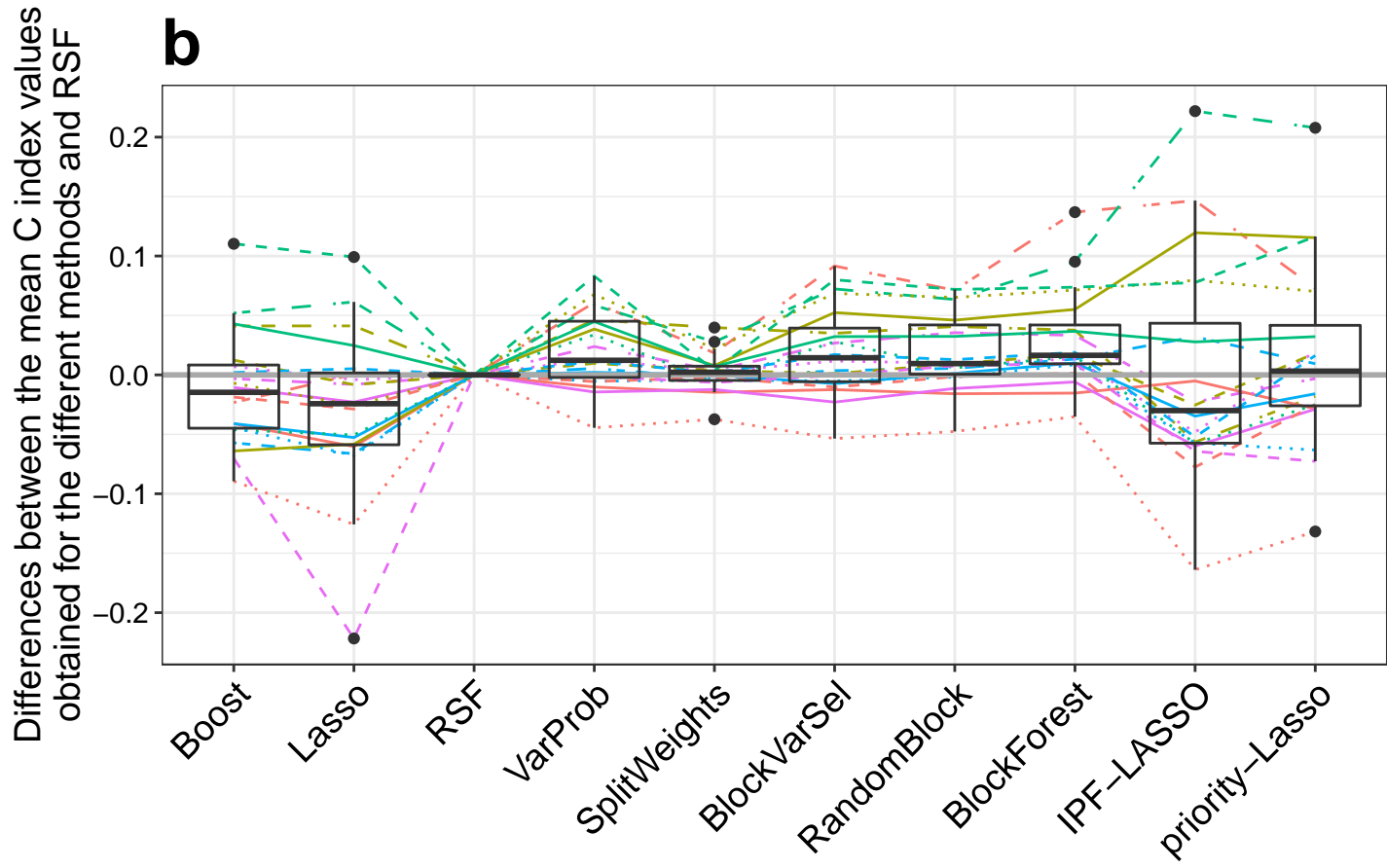**c**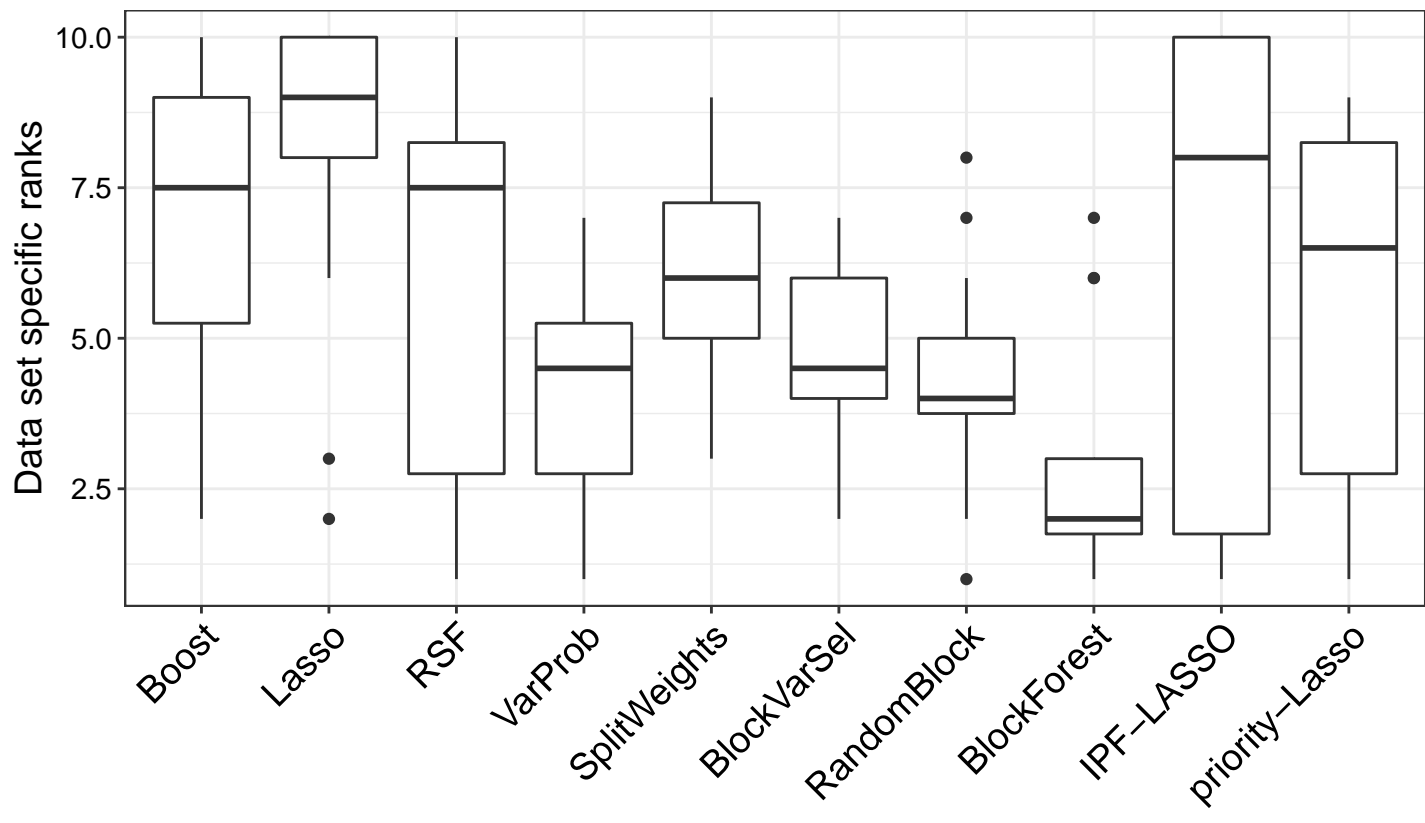

Supplement: Supplementary file 2 — Electronic Appendix. This folder contains all R Code written to perform the analyses presented in this paper and in Additional file 1 as well as Rda files enabling fast evaluation of the results. (ZIP 26,855 kb) [file 12859_2019_2942_MOESM2_ESM.zip › Additional_file_2_HornungWright/Figures/Figure3.pdf]

Differences between mean C index values  
obtained for BlockForest / VarProb and RSF

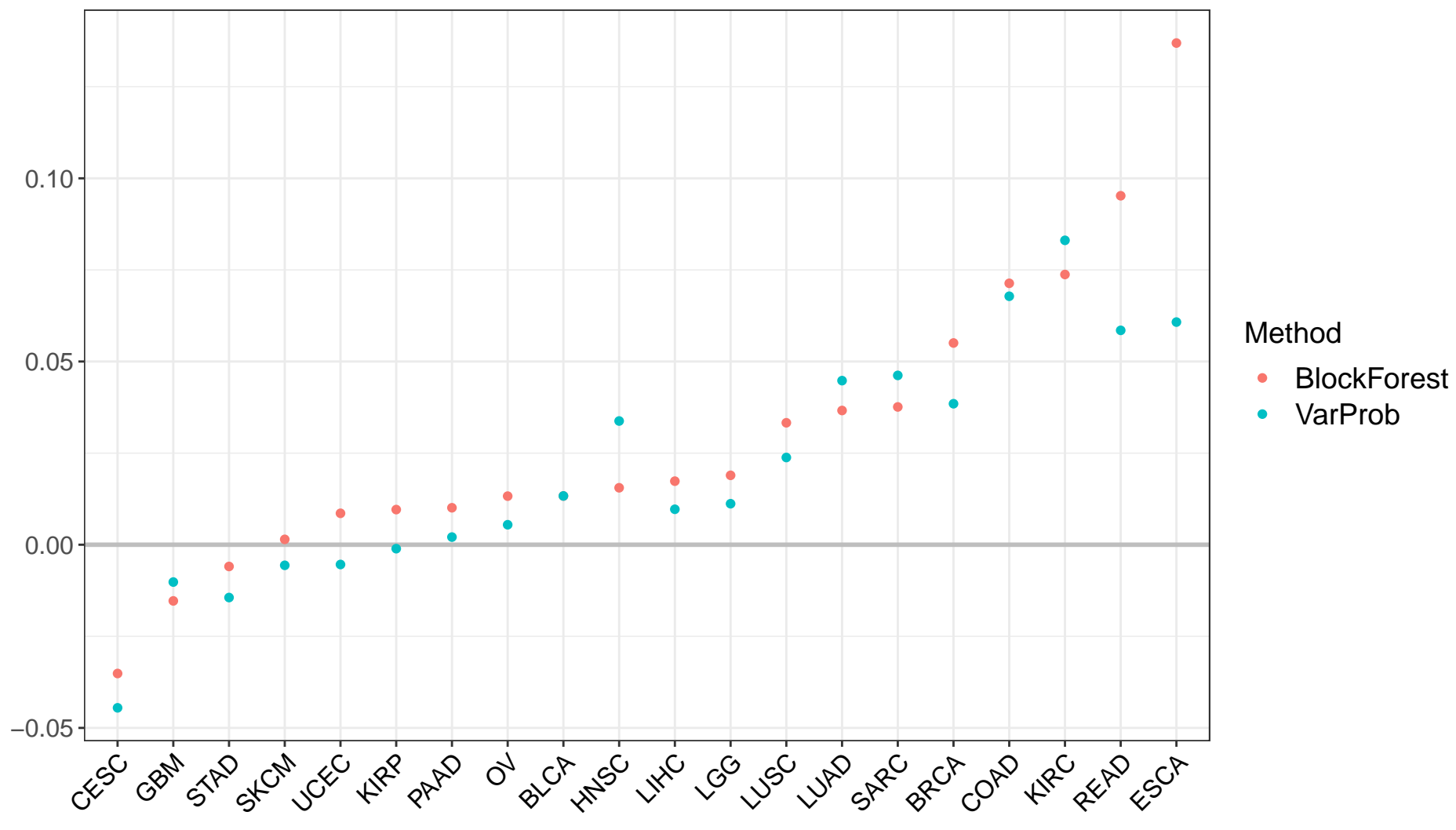

Supplement: Supplementary file 2 — Electronic Appendix. This folder contains all R Code written to perform the analyses presented in this paper and in Additional file 1 as well as Rda files enabling fast evaluation of the results. (ZIP 26,855 kb) [file 12859_2019_2942_MOESM2_ESM.zip › Additional_file_2_HornungWright/Figures/Figure4.pdf]

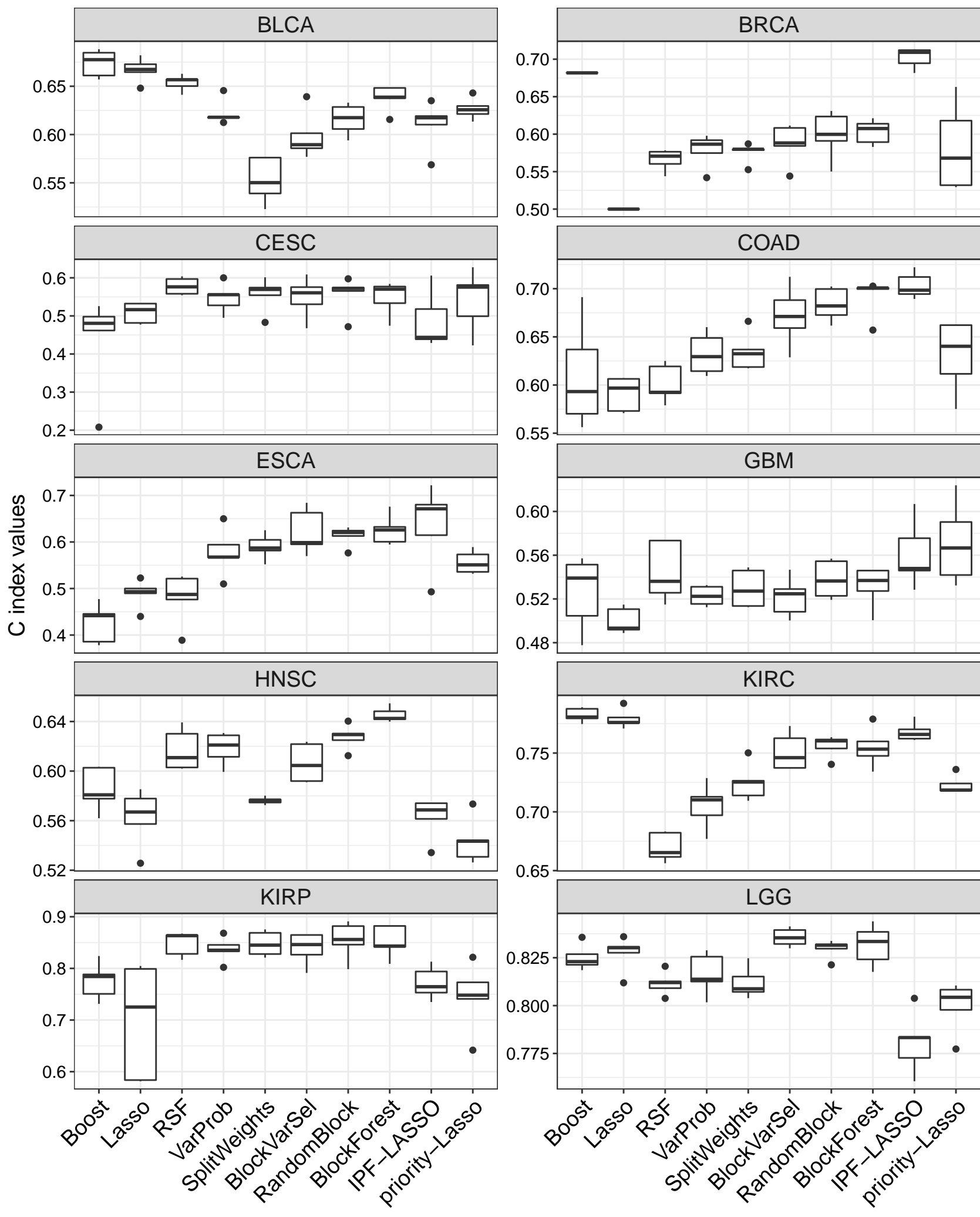

Supplement: Supplementary file 2 — Electronic Appendix. This folder contains all R Code written to perform the analyses presented in this paper and in Additional file 1 as well as Rda files enabling fast evaluation of the results. (ZIP 26,855 kb) [file 12859_2019_2942_MOESM2_ESM.zip › Additional_file_2_HornungWright/Figures/Results_AnalysisCluster_1.pdf]

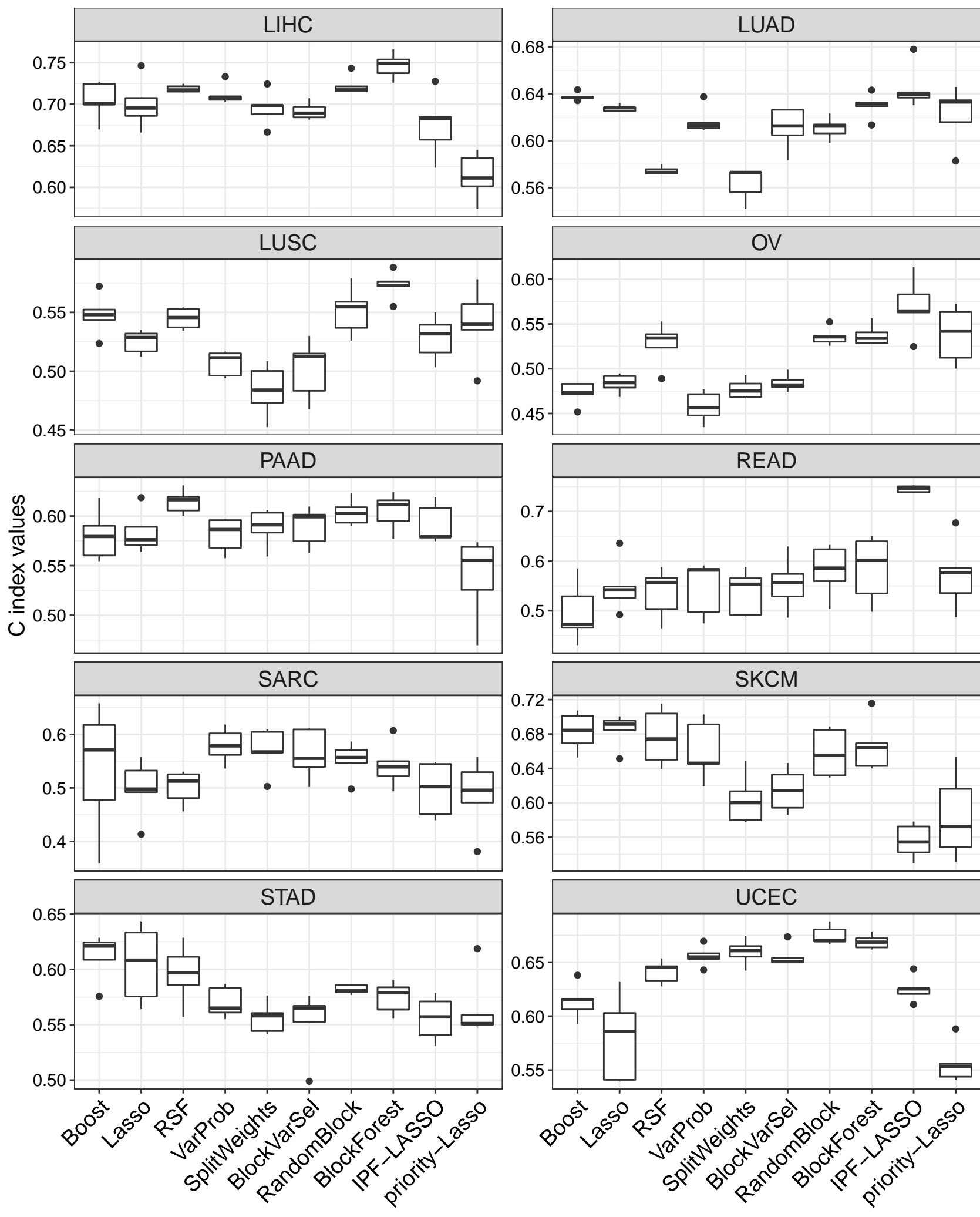

Supplement: Supplementary file 2 — Electronic Appendix. This folder contains all R Code written to perform the analyses presented in this paper and in Additional file 1 as well as Rda files enabling fast evaluation of the results. (ZIP 26,855 kb) [file 12859_2019_2942_MOESM2_ESM.zip › Additional_file_2_HornungWright/Figures/Results_AnalysisCluster_2.pdf]

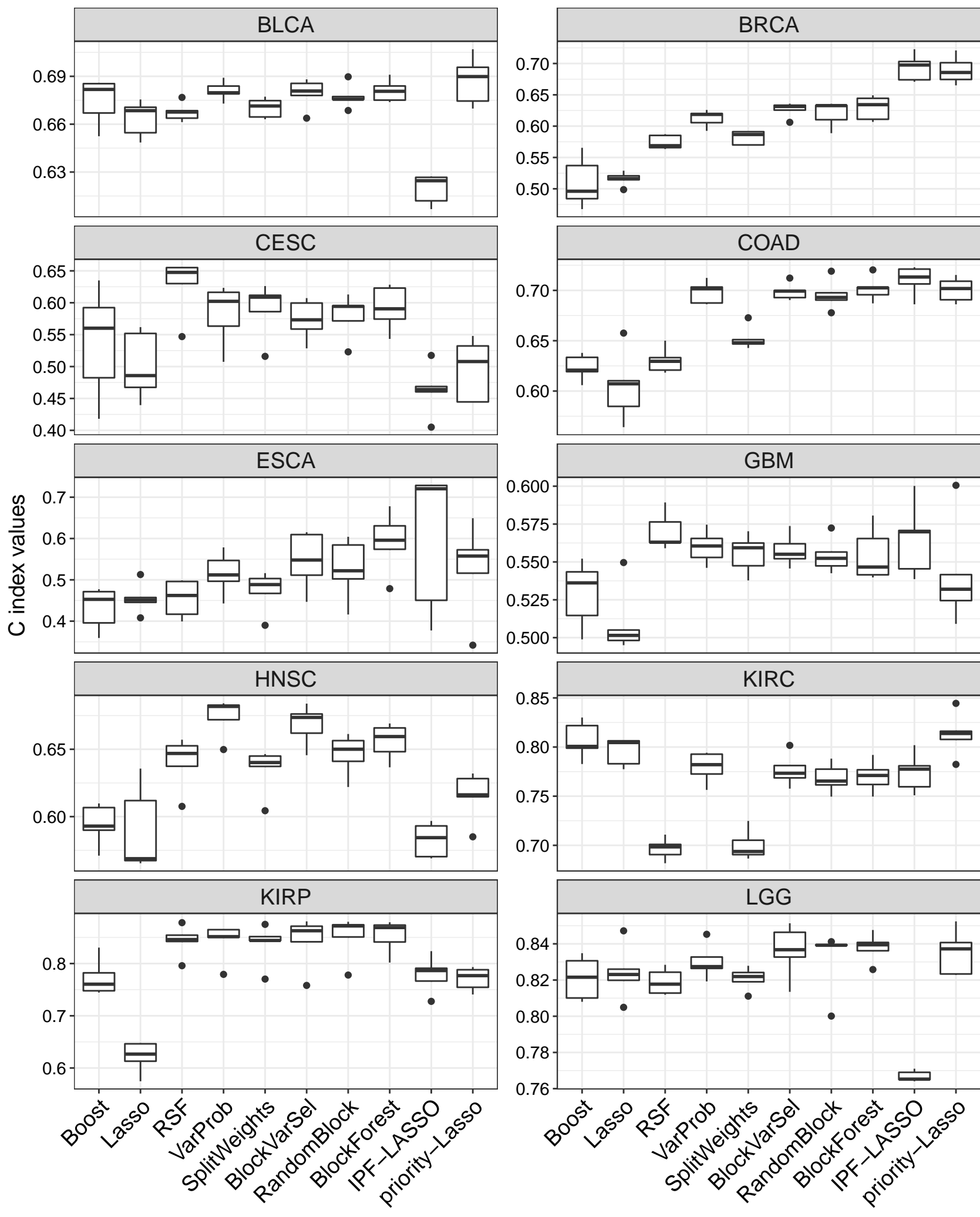

Supplement: Supplementary file 2 — Electronic Appendix. This folder contains all R Code written to perform the analyses presented in this paper and in Additional file 1 as well as Rda files enabling fast evaluation of the results. (ZIP 26,855 kb) [file 12859_2019_2942_MOESM2_ESM.zip › Additional_file_2_HornungWright/Figures/Results_AnalysisClusterTwoBlocks_1.pdf]

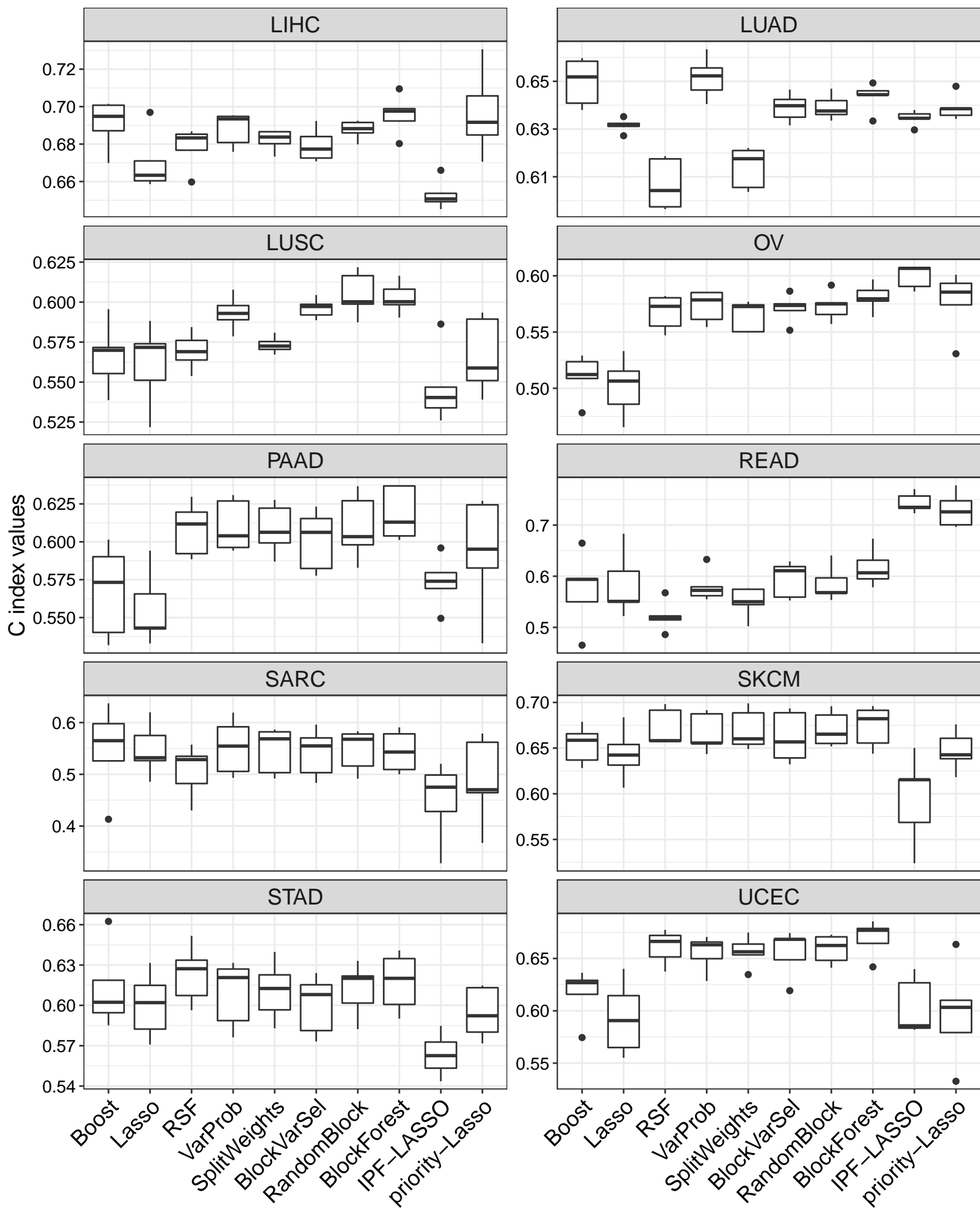

Supplement: Supplementary file 2 — Electronic Appendix. This folder contains all R Code written to perform the analyses presented in this paper and in Additional file 1 as well as Rda files enabling fast evaluation of the results. (ZIP 26,855 kb) [file 12859_2019_2942_MOESM2_ESM.zip › Additional_file_2_HornungWright/Figures/Results_AnalysisClusterTwoBlocks_2.pdf]

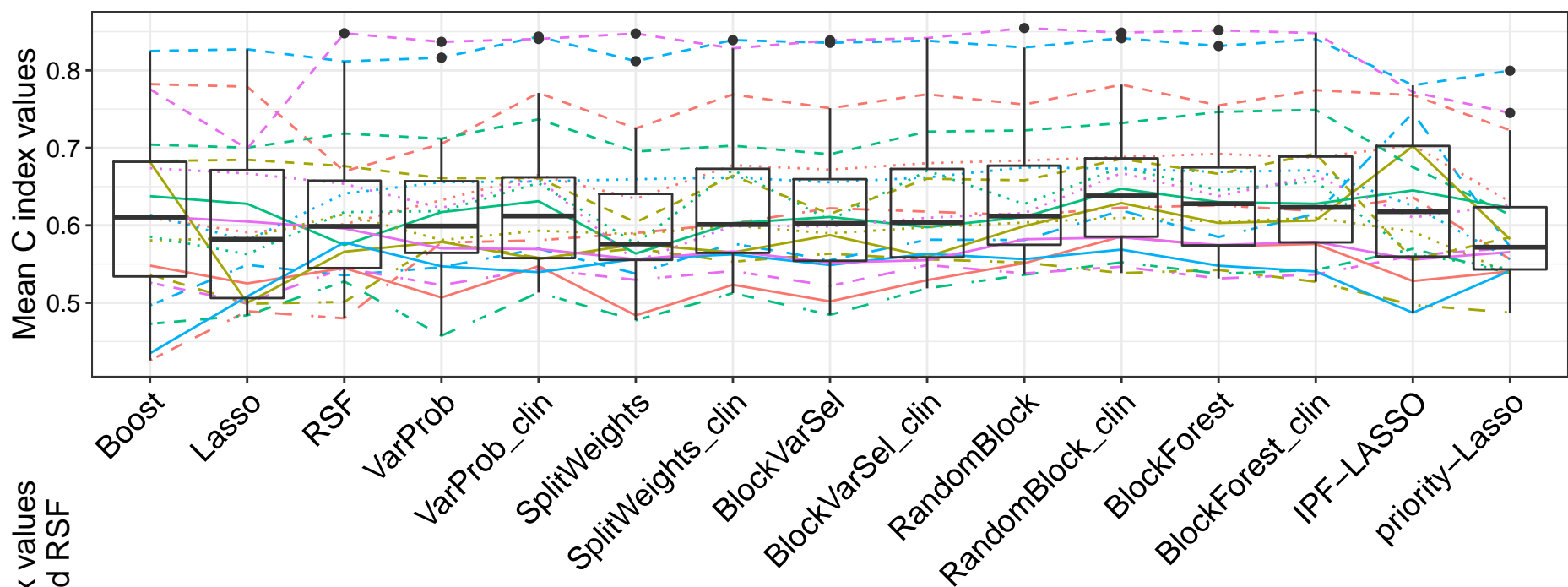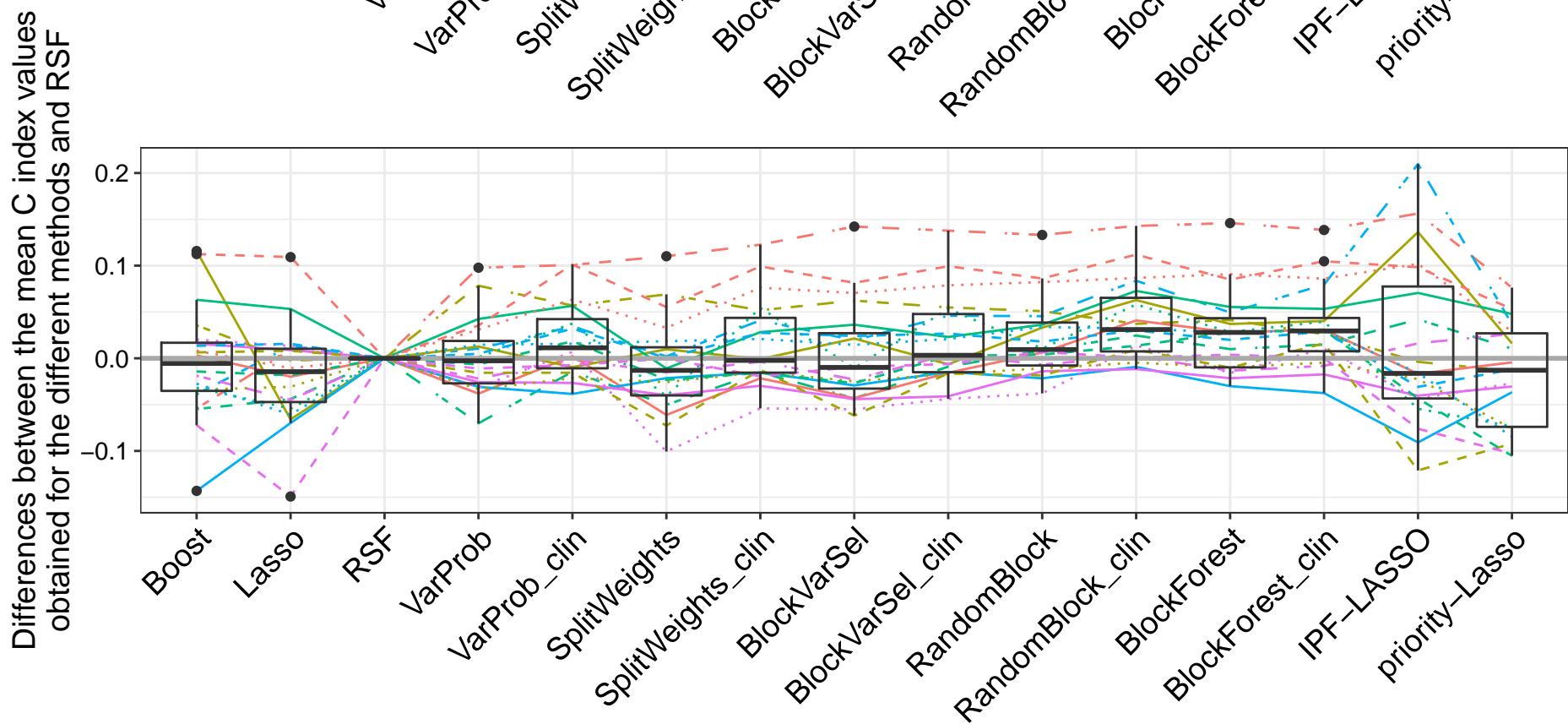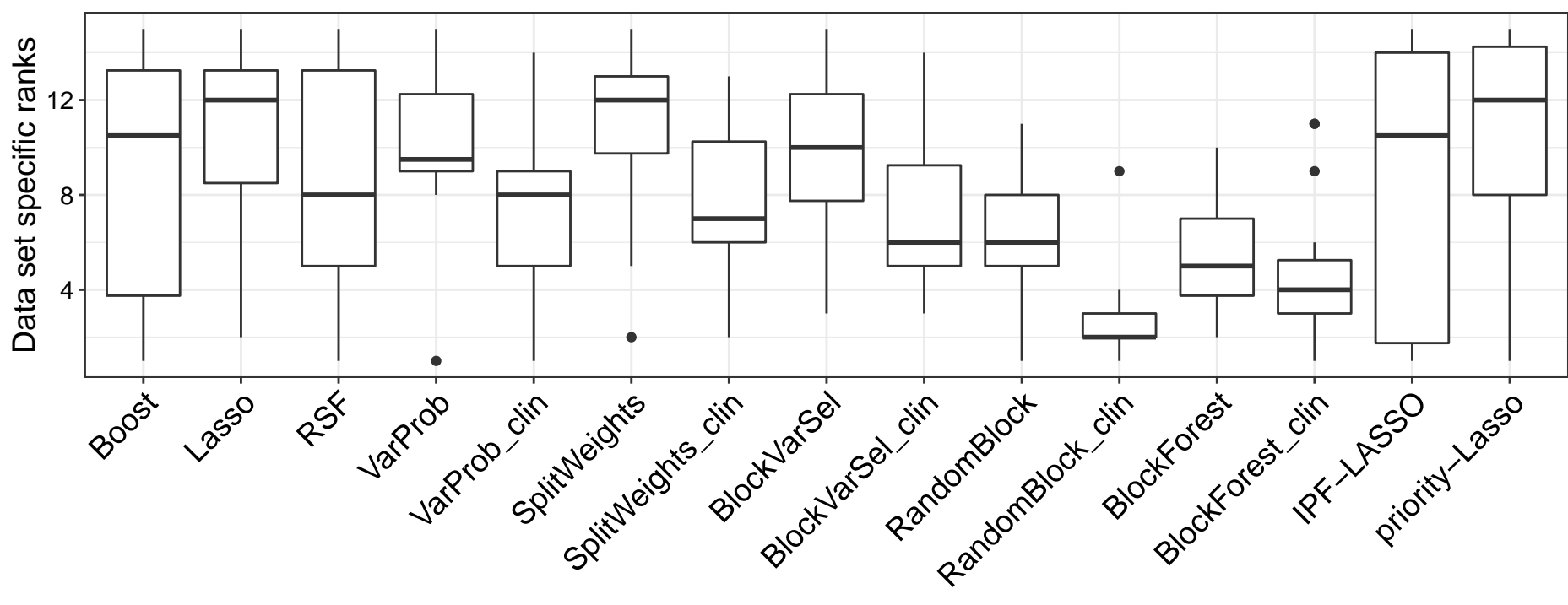

Supplement: Supplementary file 2 — Electronic Appendix. This folder contains all R Code written to perform the analyses presented in this paper and in Additional file 1 as well as Rda files enabling fast evaluation of the results. (ZIP 26,855 kb) [file 12859_2019_2942_MOESM2_ESM.zip › Additional_file_2_HornungWright/Figures/Results_MultiOmicsWithalwaysclinical.pdf]

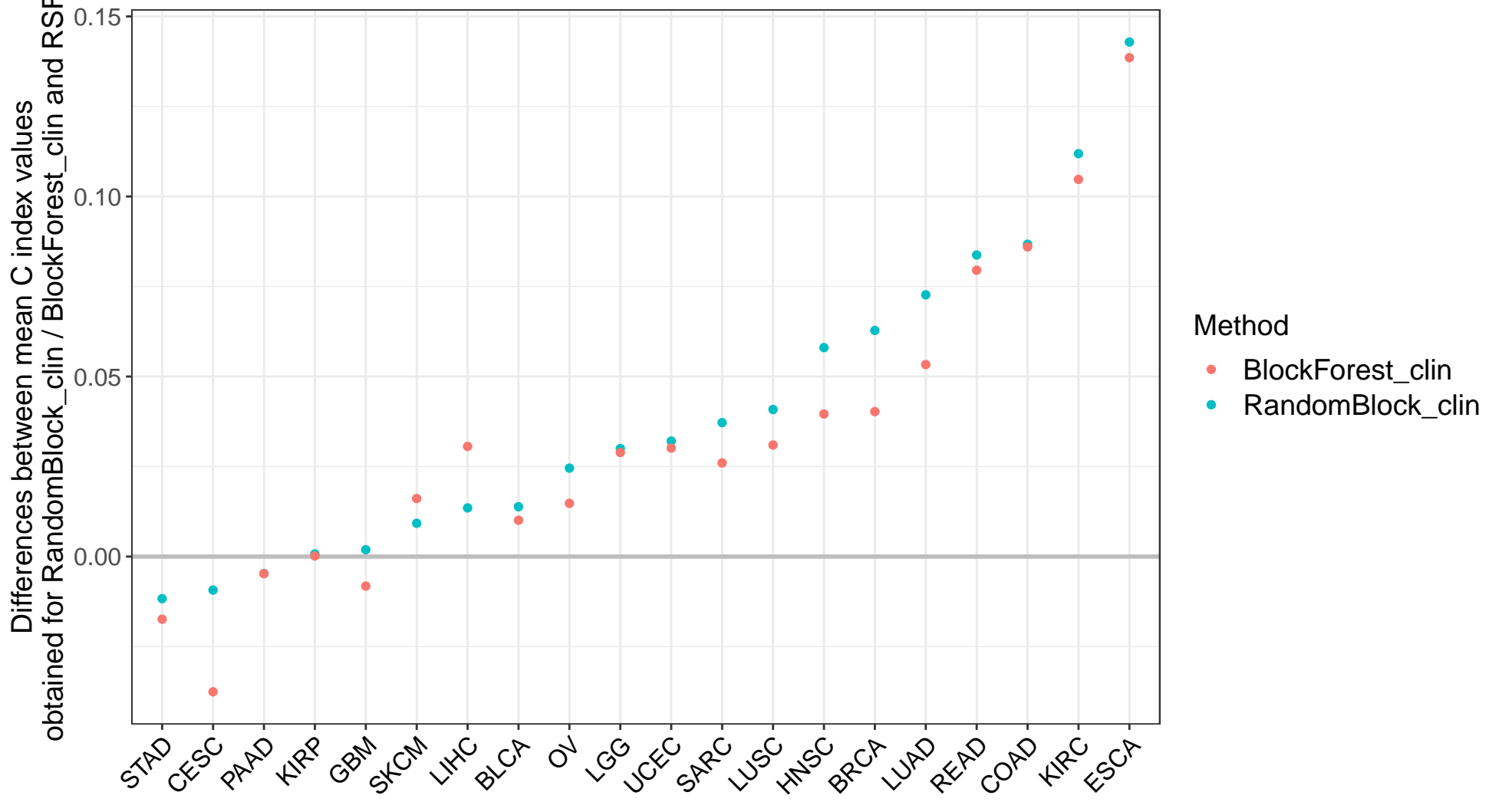

Supplement: Supplementary file 2 — Electronic Appendix. This folder contains all R Code written to perform the analyses presented in this paper and in Additional file 1 as well as Rda files enabling fast evaluation of the results. (ZIP 26,855 kb) [file 12859_2019_2942_MOESM2_ESM.zip › Additional_file_2_HornungWright/Figures/Results_MultiOmicsWithalwaysclinical_PerfDiff.pdf]

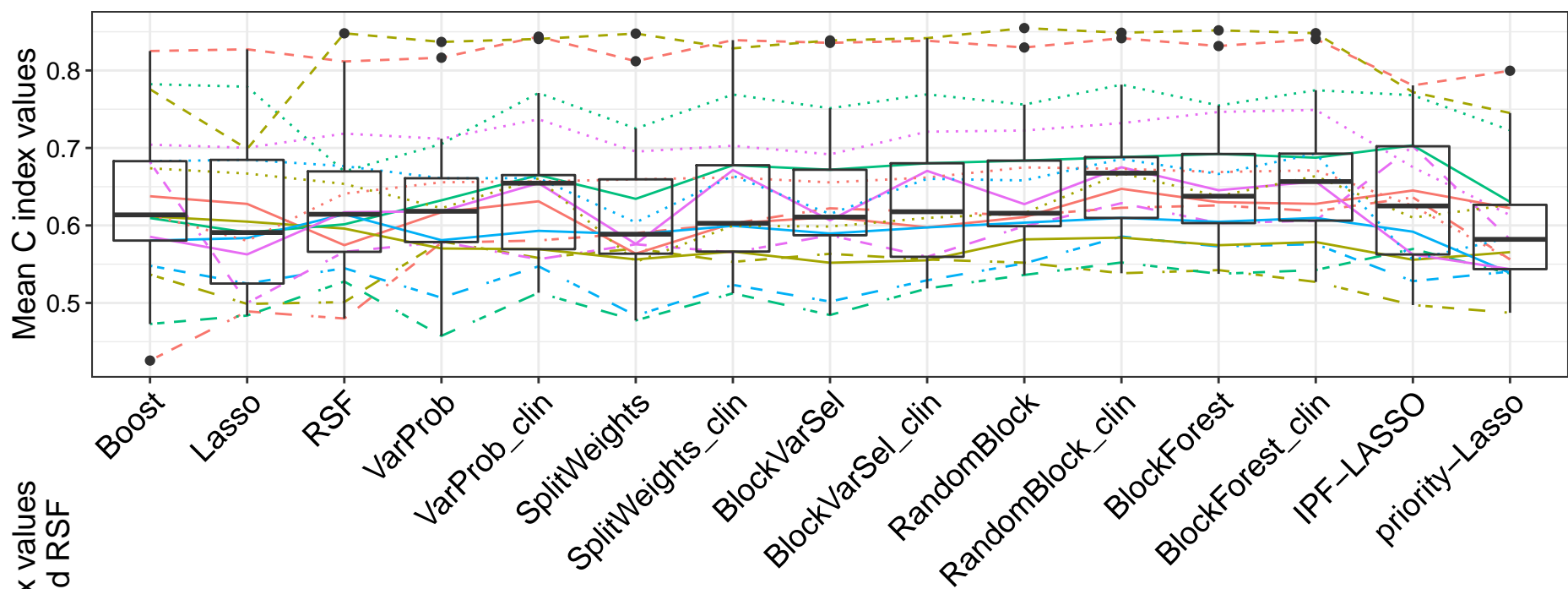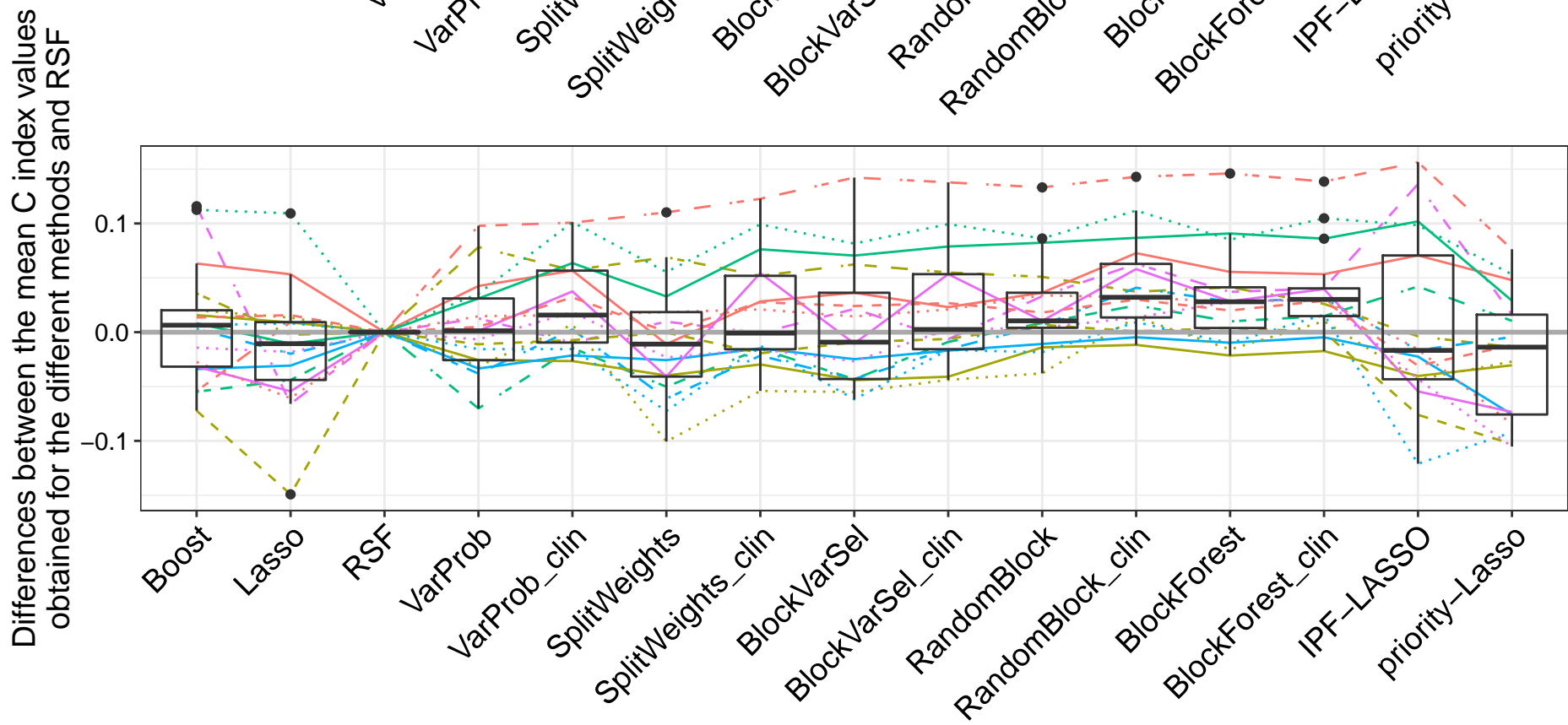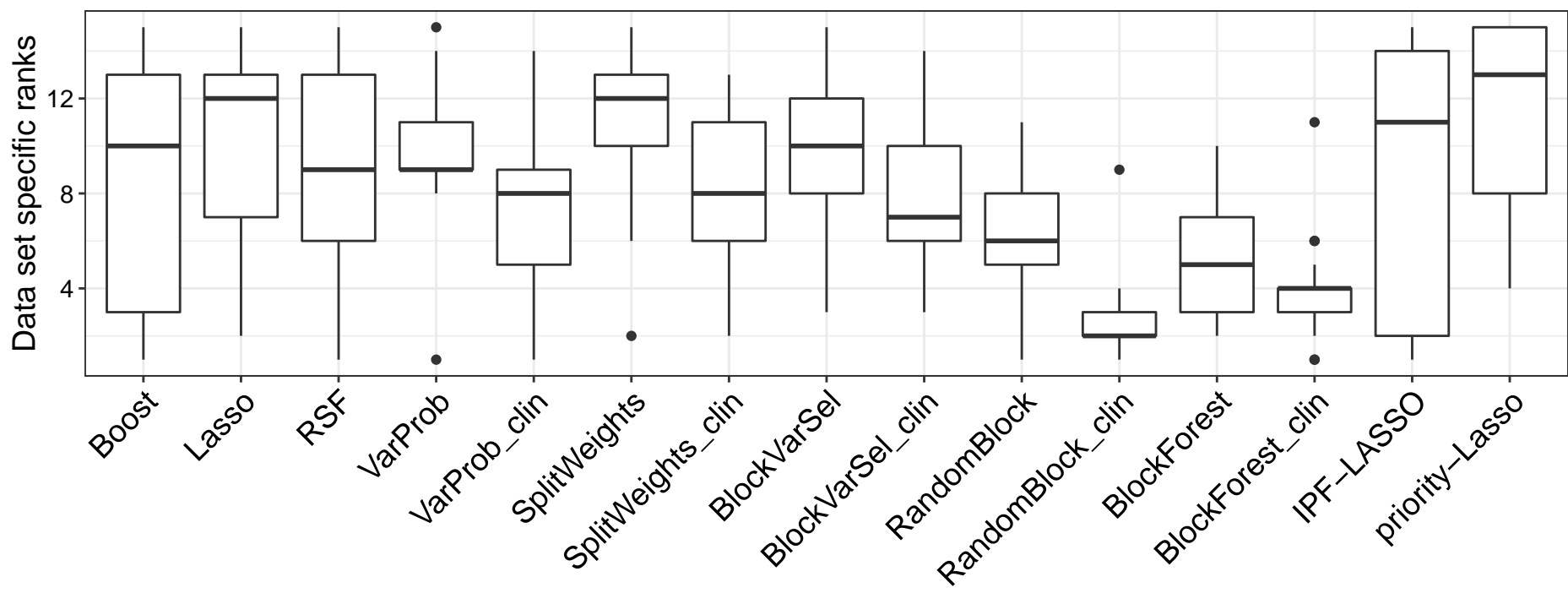

Supplement: Supplementary file 2 — Electronic Appendix. This folder contains all R Code written to perform the analyses presented in this paper and in Additional file 1 as well as Rda files enabling fast evaluation of the results. (ZIP 26,855 kb) [file 12859_2019_2942_MOESM2_ESM.zip › Additional_file_2_HornungWright/Figures/Results_MultiOmicsWithalwaysclinical_without_CESC_GBM_READ.pdf]

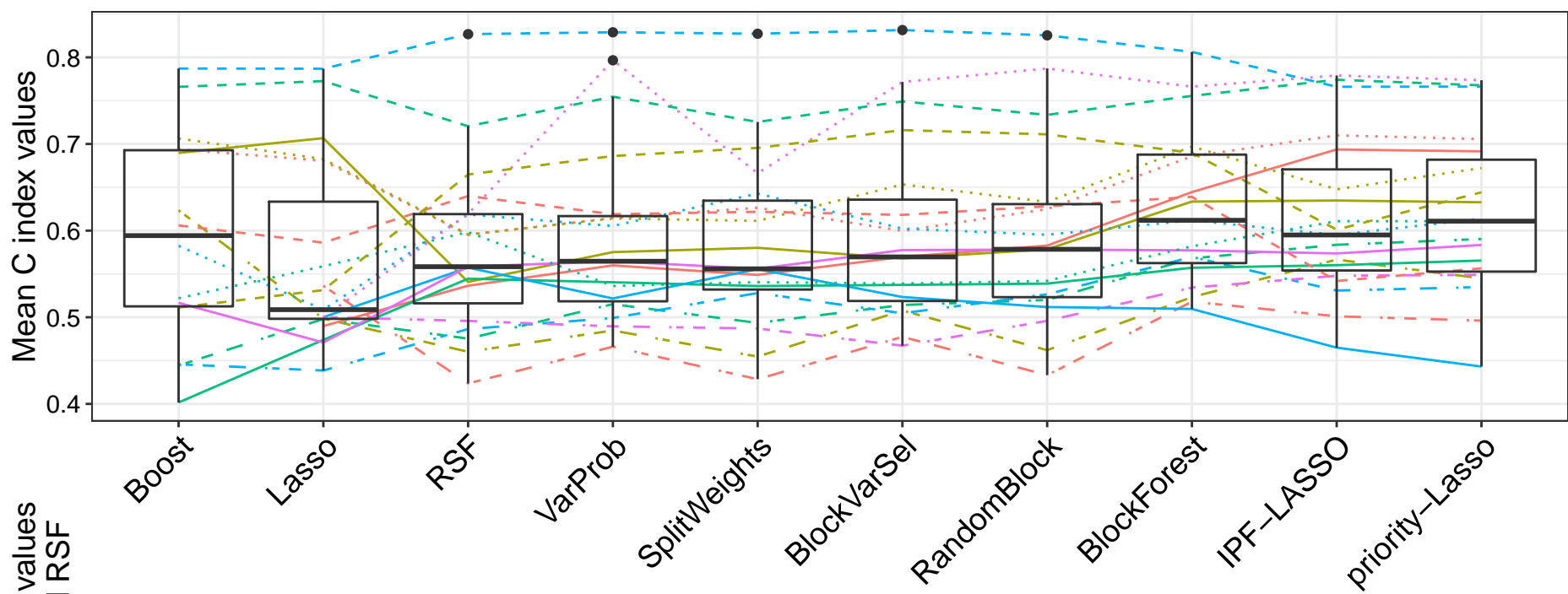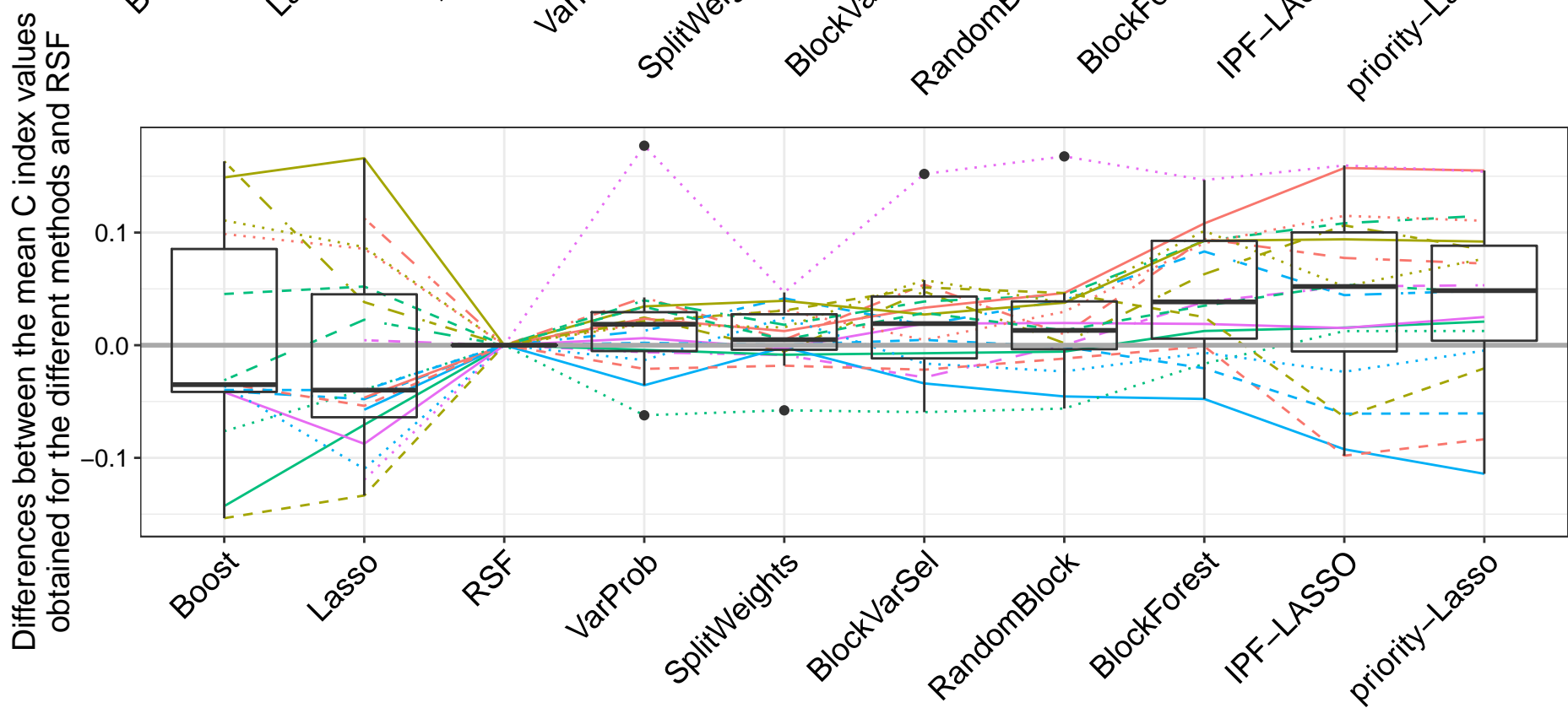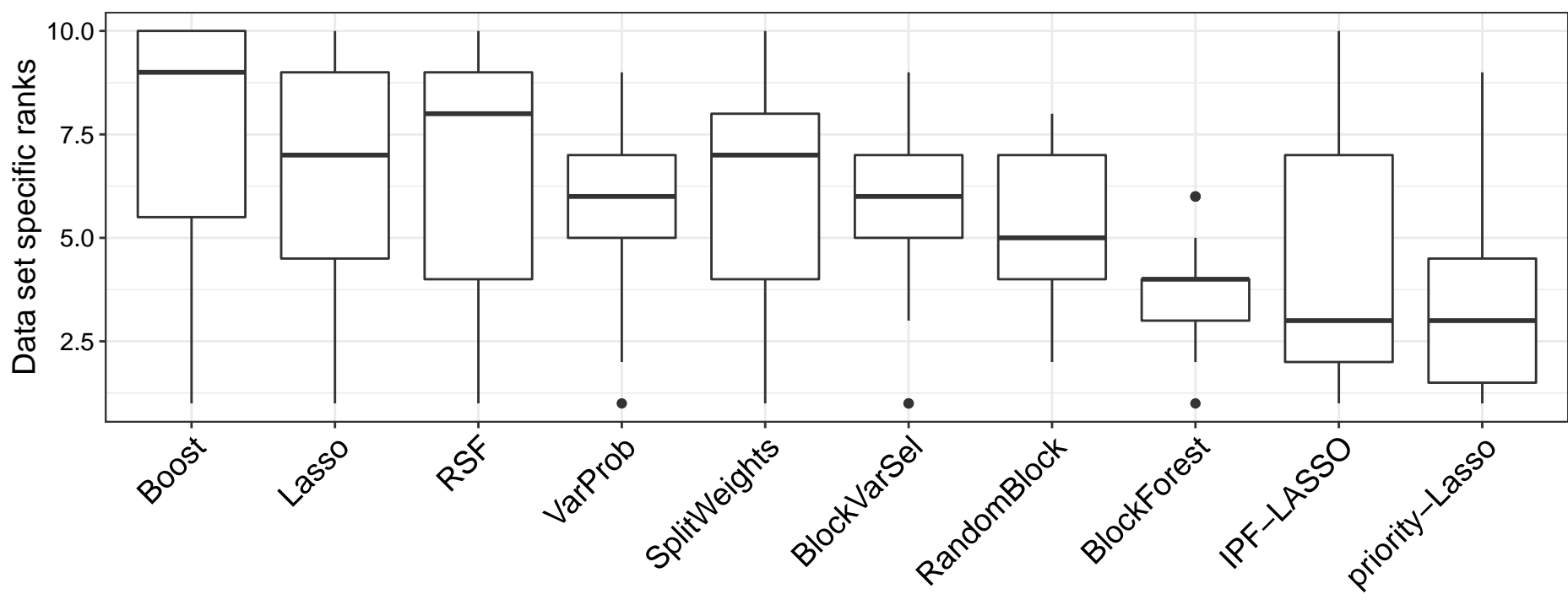

Supplement: Supplementary file 2 — Electronic Appendix. This folder contains all R Code written to perform the analyses presented in this paper and in Additional file 1 as well as Rda files enabling fast evaluation of the results. (ZIP 26,855 kb) [file 12859_2019_2942_MOESM2_ESM.zip › Additional_file_2_HornungWright/Figures/Results_TwoBlocksMutation.pdf]

Differences between mean C index values  
obtained for BlockForest / priority-Lasso and RSF

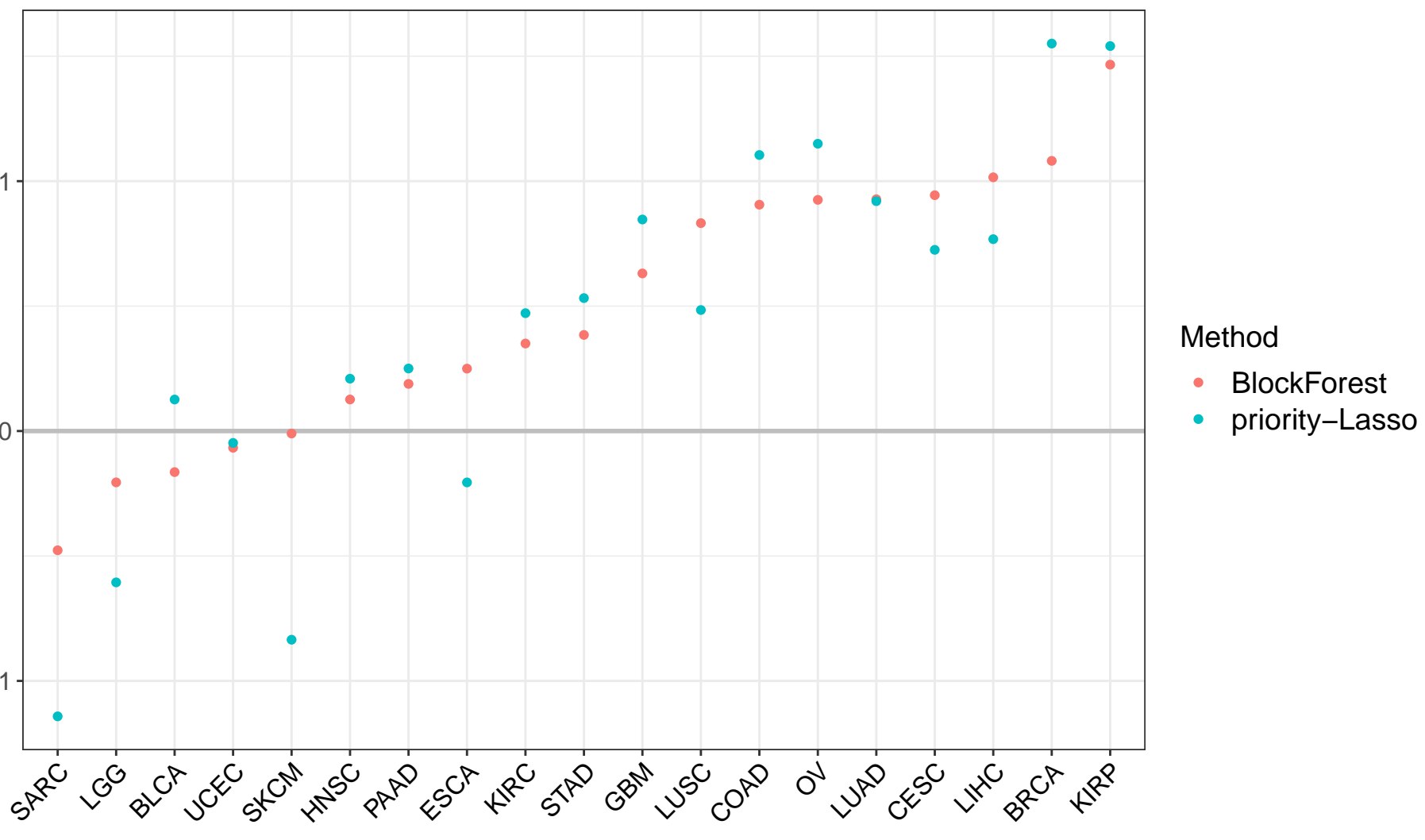

Supplement: Supplementary file 2 — Electronic Appendix. This folder contains all R Code written to perform the analyses presented in this paper and in Additional file 1 as well as Rda files enabling fast evaluation of the results. (ZIP 26,855 kb) [file 12859_2019_2942_MOESM2_ESM.zip › Additional_file_2_HornungWright/Figures/Results_TwoBlocksMutation_PerfDiff.pdf]

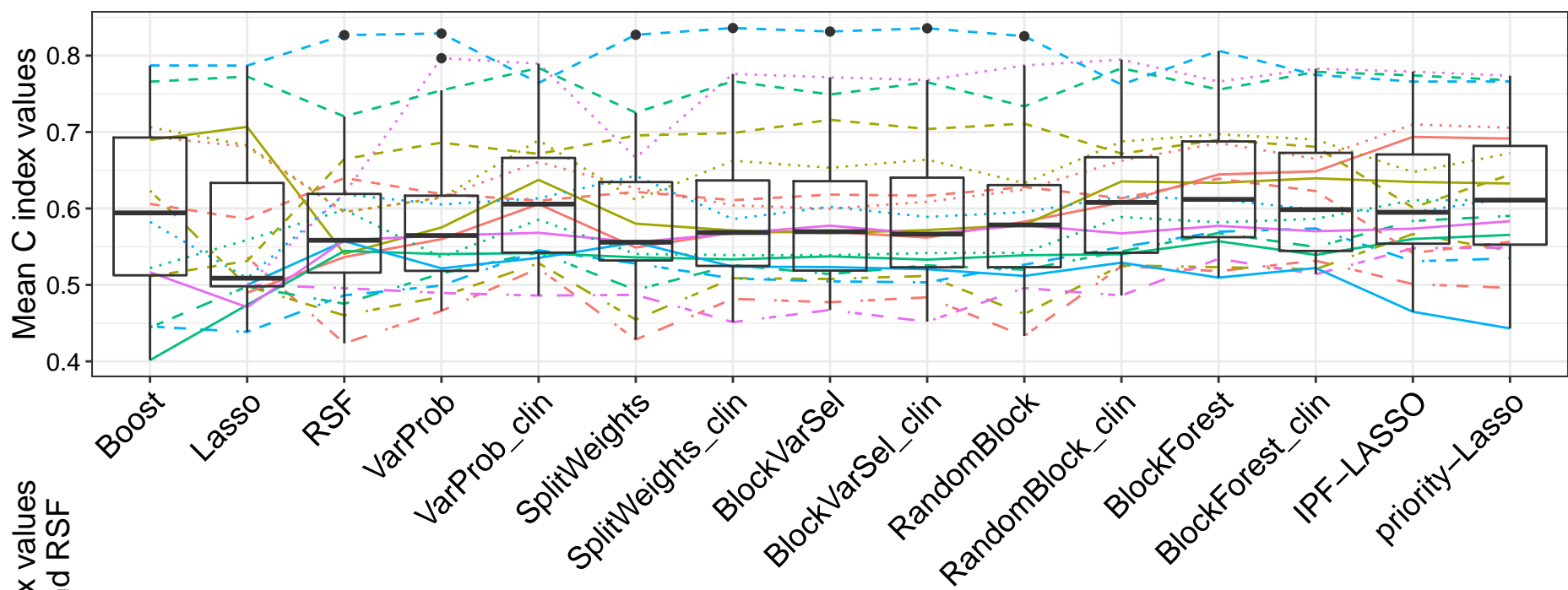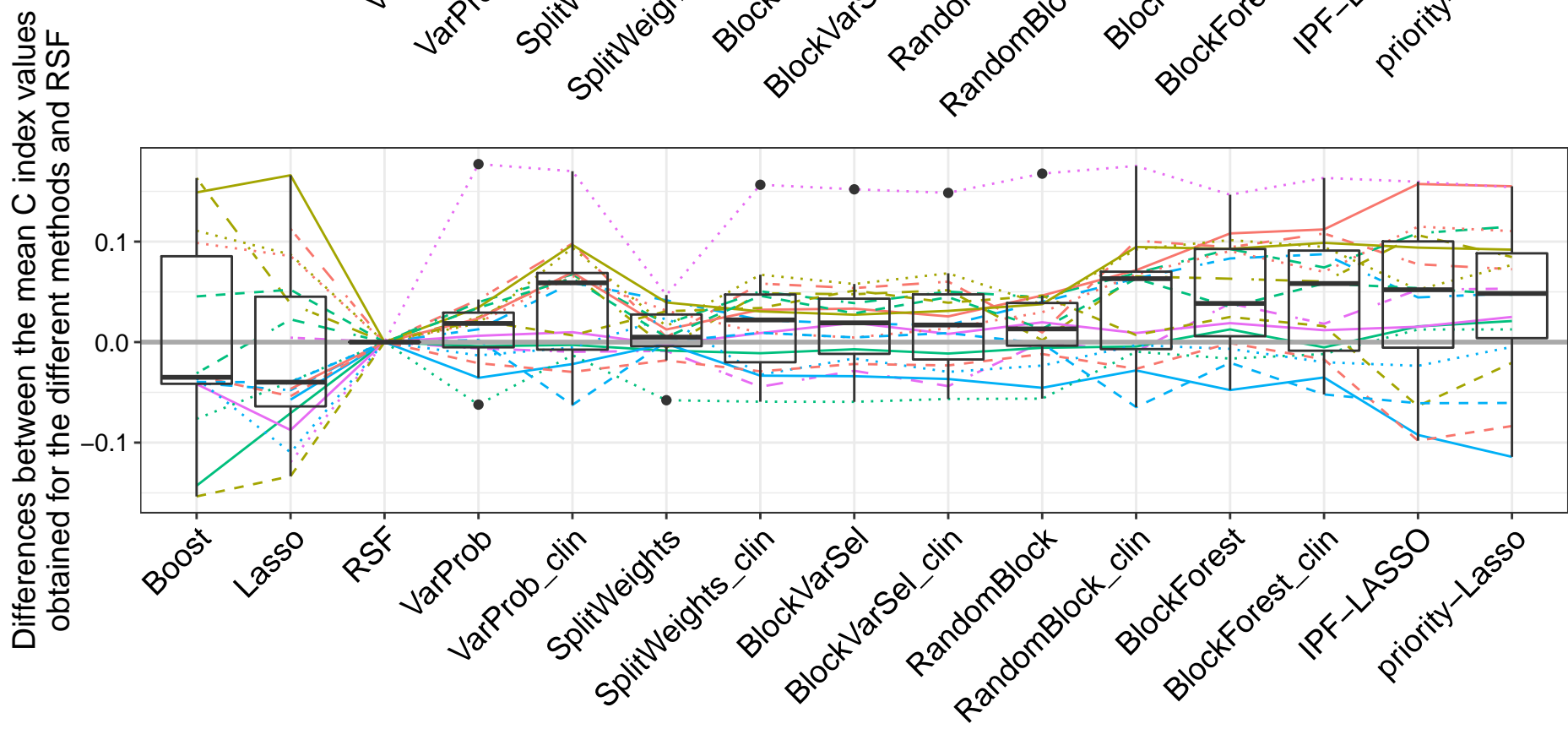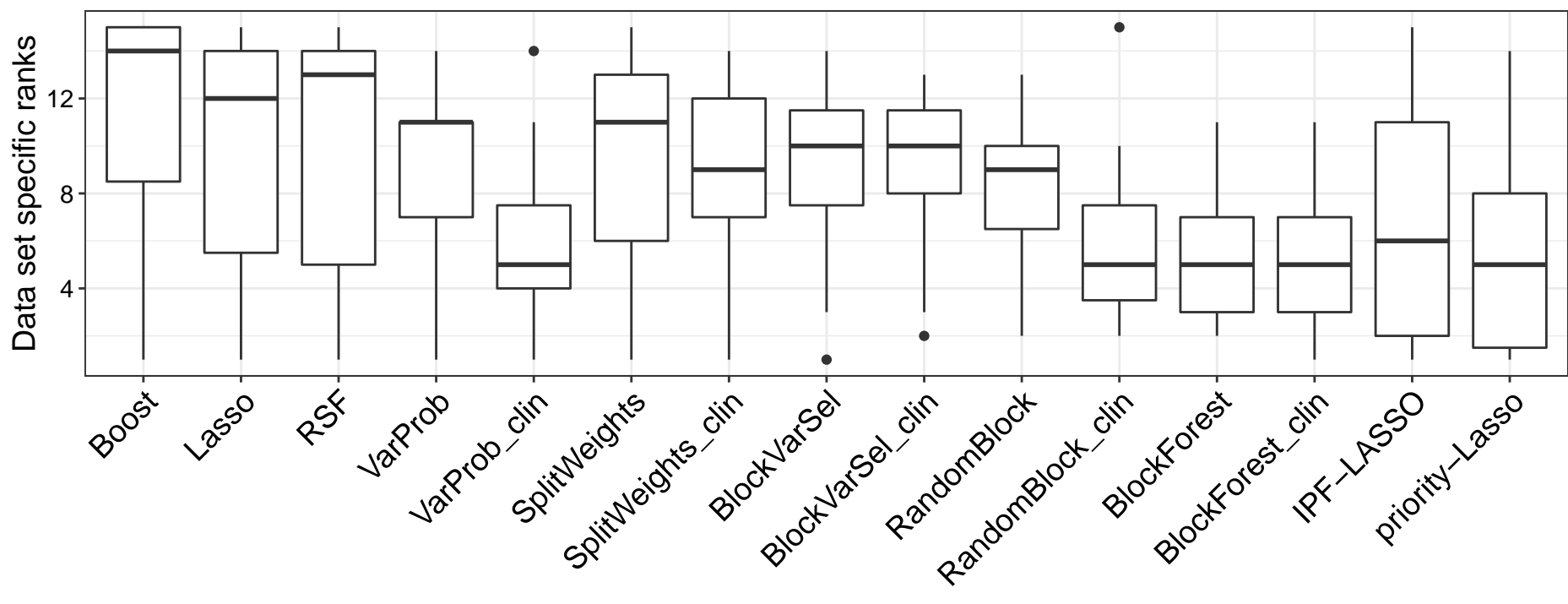

Supplement: Supplementary file 2 — Electronic Appendix. This folder contains all R Code written to perform the analyses presented in this paper and in Additional file 1 as well as Rda files enabling fast evaluation of the results. (ZIP 26,855 kb) [file 12859_2019_2942_MOESM2_ESM.zip › Additional_file_2_HornungWright/Figures/Results_TwoBlocksMutationWithalwaysclinical.pdf]

Differences between mean C index values  
obtained for BlockForest\_clin / BlockForest and RSF

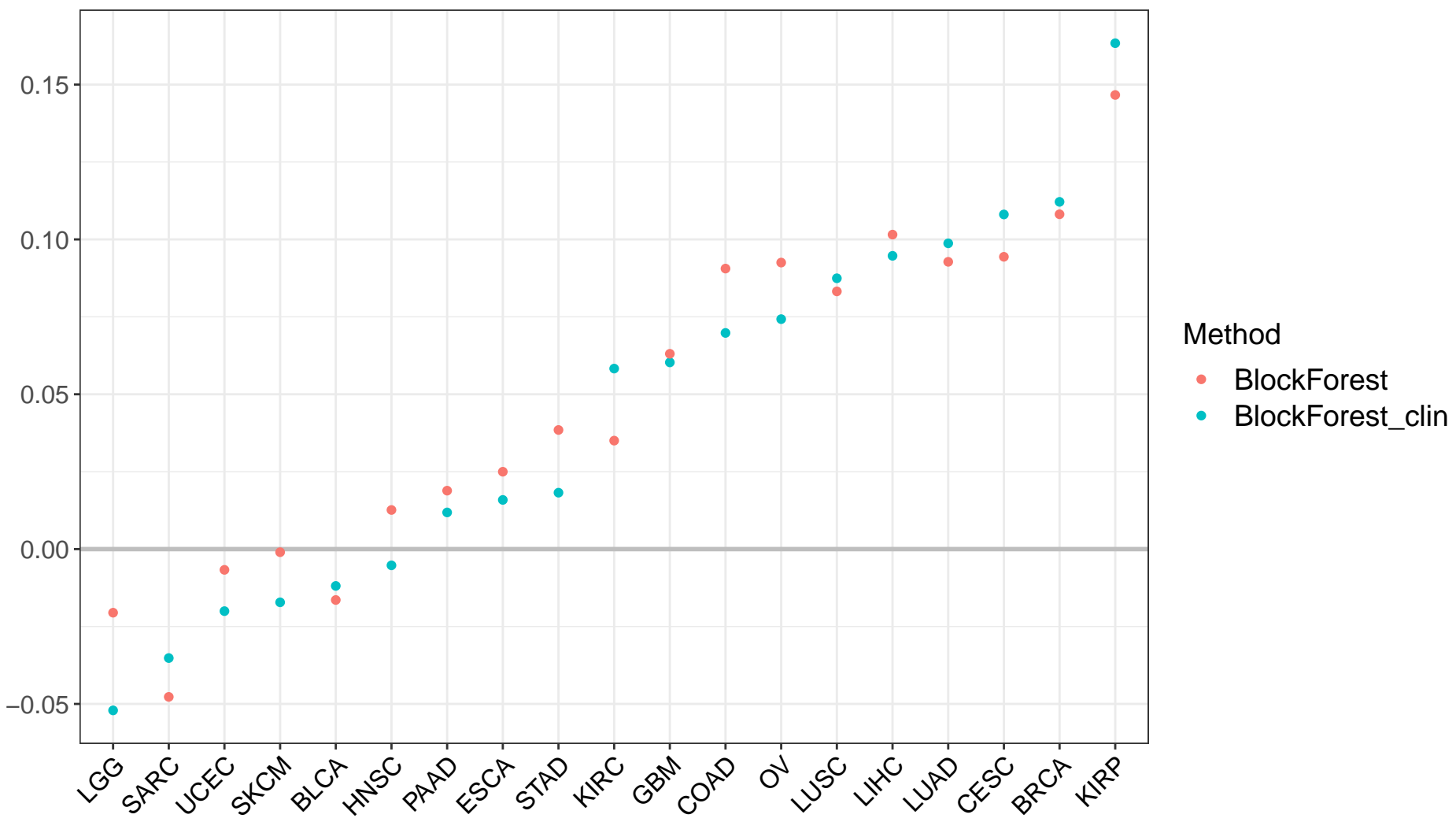

Supplement: Supplementary file 2 — Electronic Appendix. This folder contains all R Code written to perform the analyses presented in this paper and in Additional file 1 as well as Rda files enabling fast evaluation of the results. (ZIP 26,855 kb) [file 12859_2019_2942_MOESM2_ESM.zip › Additional_file_2_HornungWright/Figures/Results_TwoBlocksMutationWithalwaysclinical_PerfDiff.pdf]

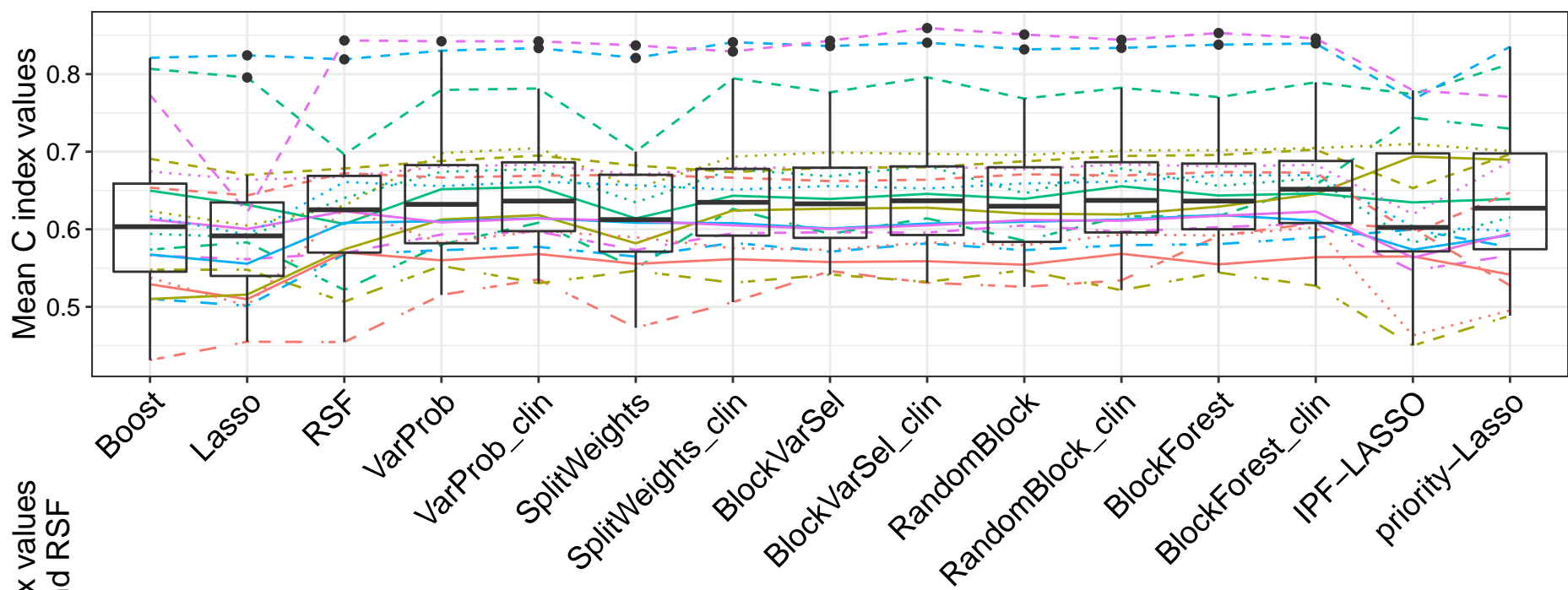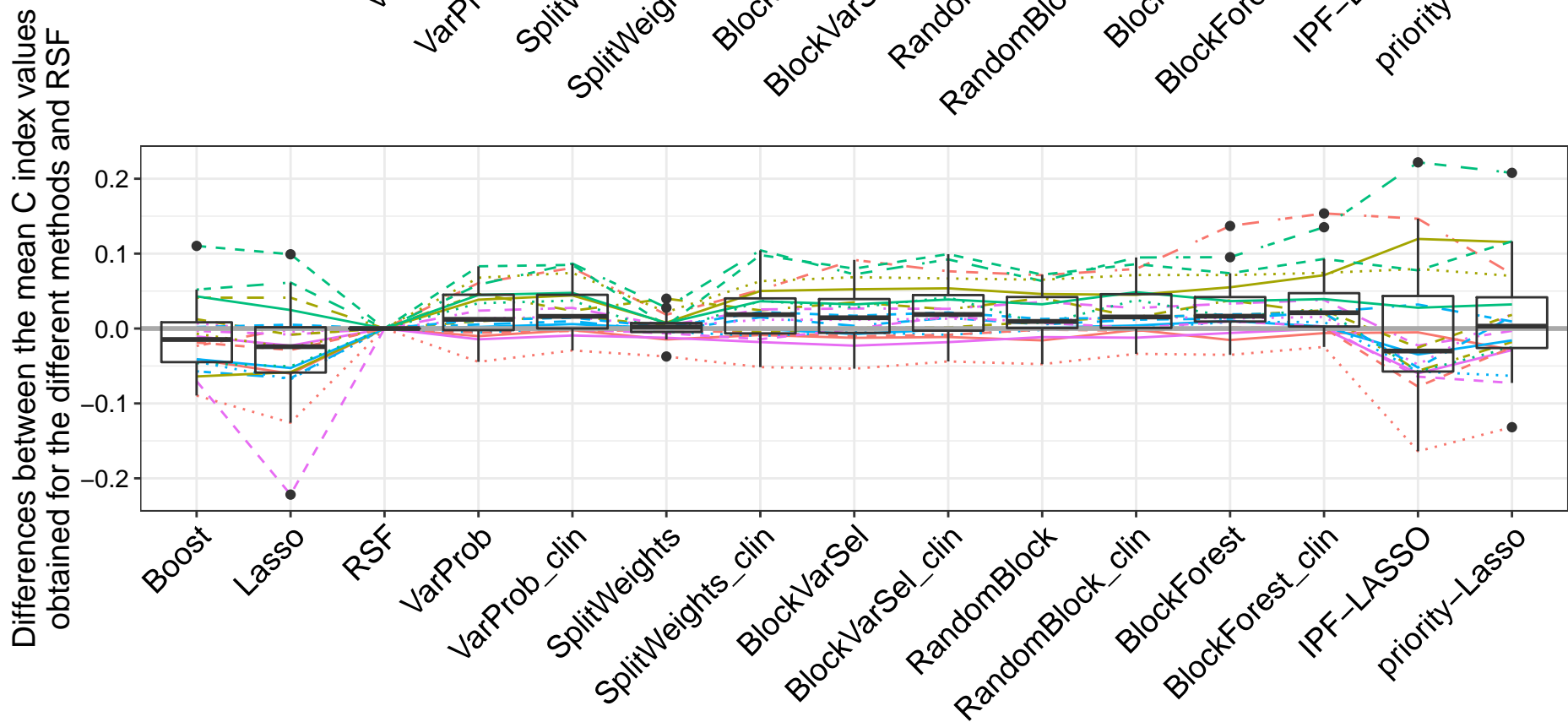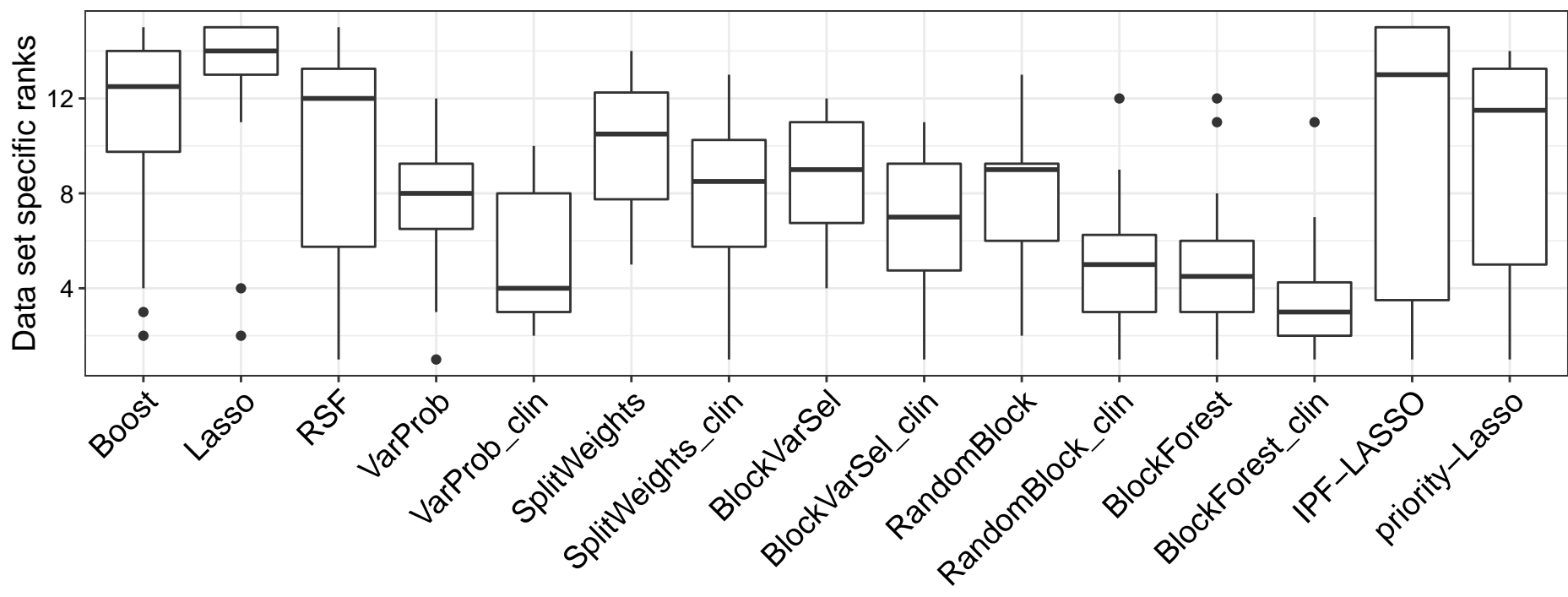

Supplement: Supplementary file 2 — Electronic Appendix. This folder contains all R Code written to perform the analyses presented in this paper and in Additional file 1 as well as Rda files enabling fast evaluation of the results. (ZIP 26,855 kb) [file 12859_2019_2942_MOESM2_ESM.zip › Additional_file_2_HornungWright/Figures/Results_TwoBlocksWithalwaysclinical.pdf]

Differences between mean C index values  
obtained for BlockForest\_clin / BlockForest and RSF

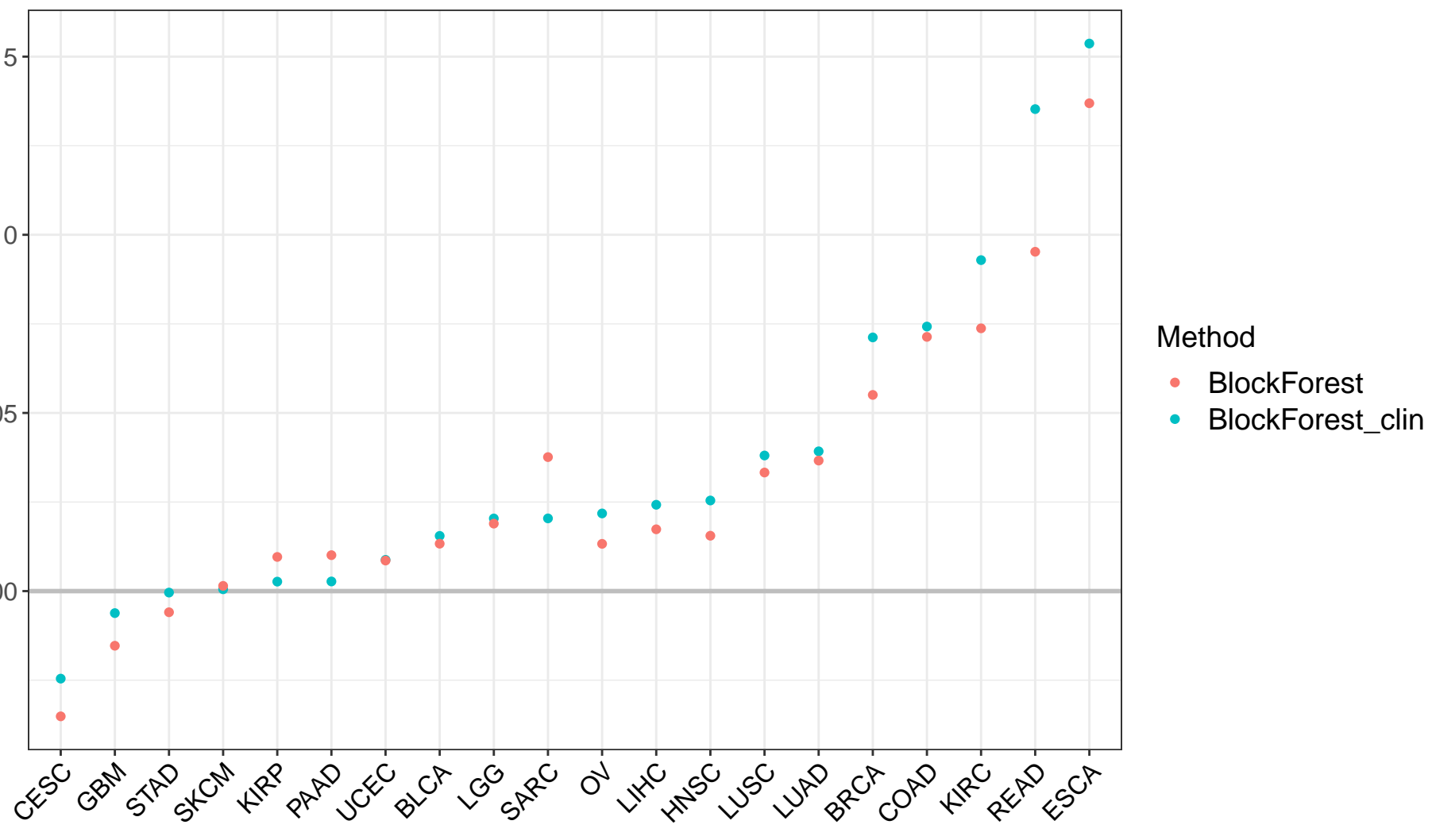

Supplement: Supplementary file 2 — Electronic Appendix. This folder contains all R Code written to perform the analyses presented in this paper and in Additional file 1 as well as Rda files enabling fast evaluation of the results. (ZIP 26,855 kb) [file 12859_2019_2942_MOESM2_ESM.zip › Additional_file_2_HornungWright/Figures/Results_TwoBlocksWithalwaysclinical_PerfDiff.pdf]

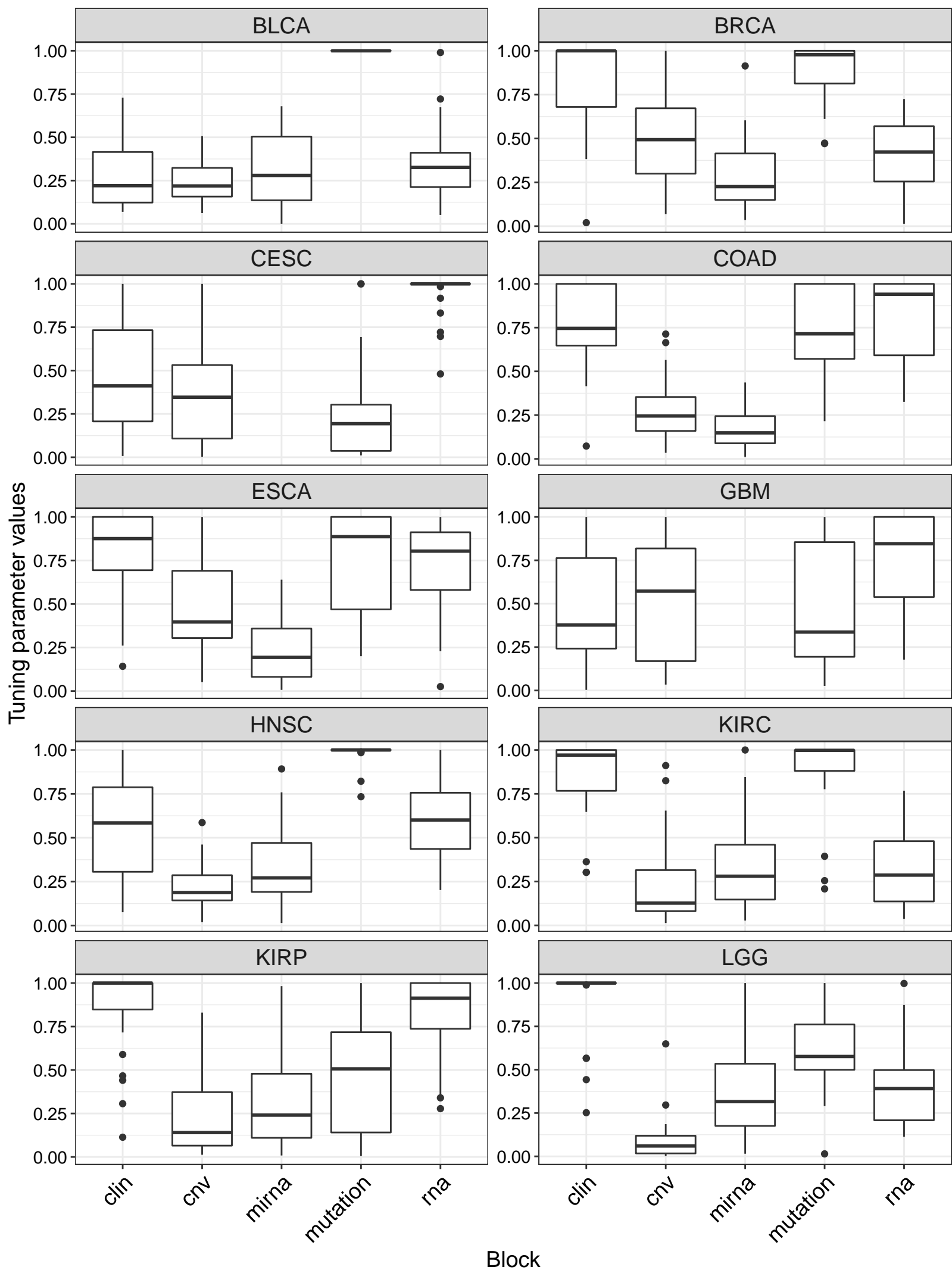

Supplement: Supplementary file 2 — Electronic Appendix. This folder contains all R Code written to perform the analyses presented in this paper and in Additional file 1 as well as Rda files enabling fast evaluation of the results. (ZIP 26,855 kb) [file 12859_2019_2942_MOESM2_ESM.zip › Additional_file_2_HornungWright/Figures/TunParam_BlockForest_1.pdf]

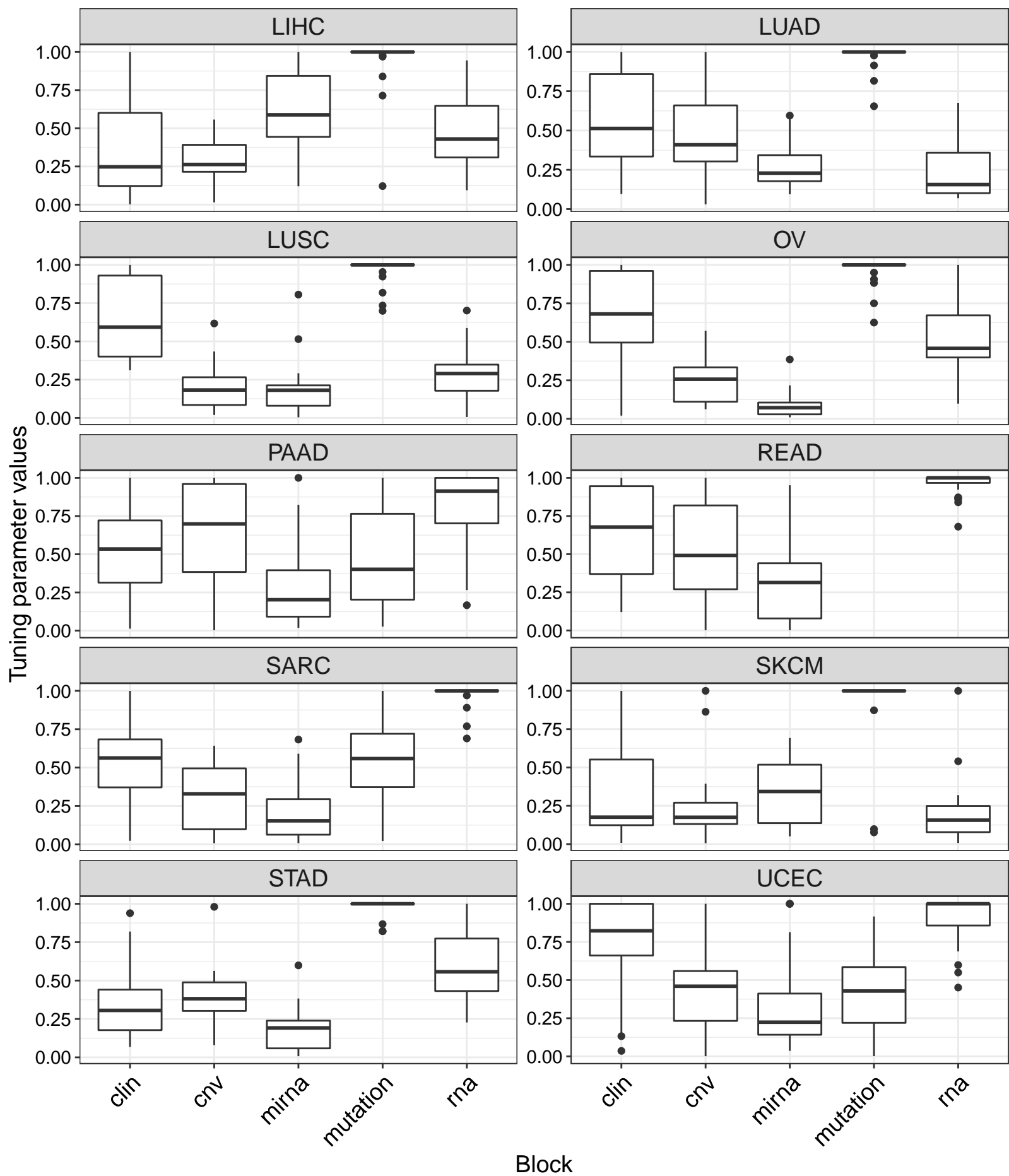

Supplement: Supplementary file 2 — Electronic Appendix. This folder contains all R Code written to perform the analyses presented in this paper and in Additional file 1 as well as Rda files enabling fast evaluation of the results. (ZIP 26,855 kb) [file 12859_2019_2942_MOESM2_ESM.zip › Additional_file_2_HornungWright/Figures/TunParam_BlockForest_2.pdf]

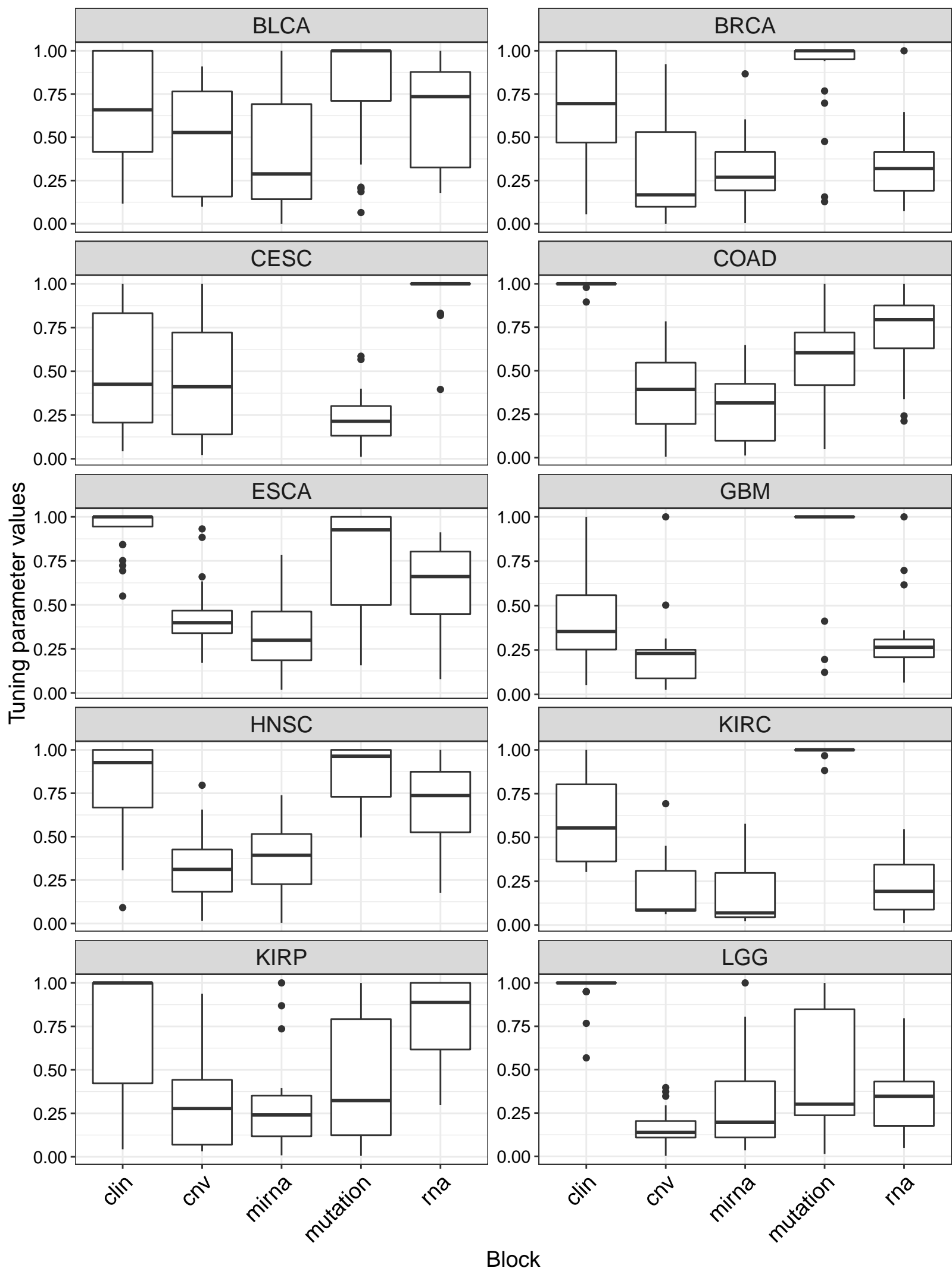

Supplement: Supplementary file 2 — Electronic Appendix. This folder contains all R Code written to perform the analyses presented in this paper and in Additional file 1 as well as Rda files enabling fast evaluation of the results. (ZIP 26,855 kb) [file 12859_2019_2942_MOESM2_ESM.zip › Additional_file_2_HornungWright/Figures/TunParam_BlockVarSel_1.pdf]

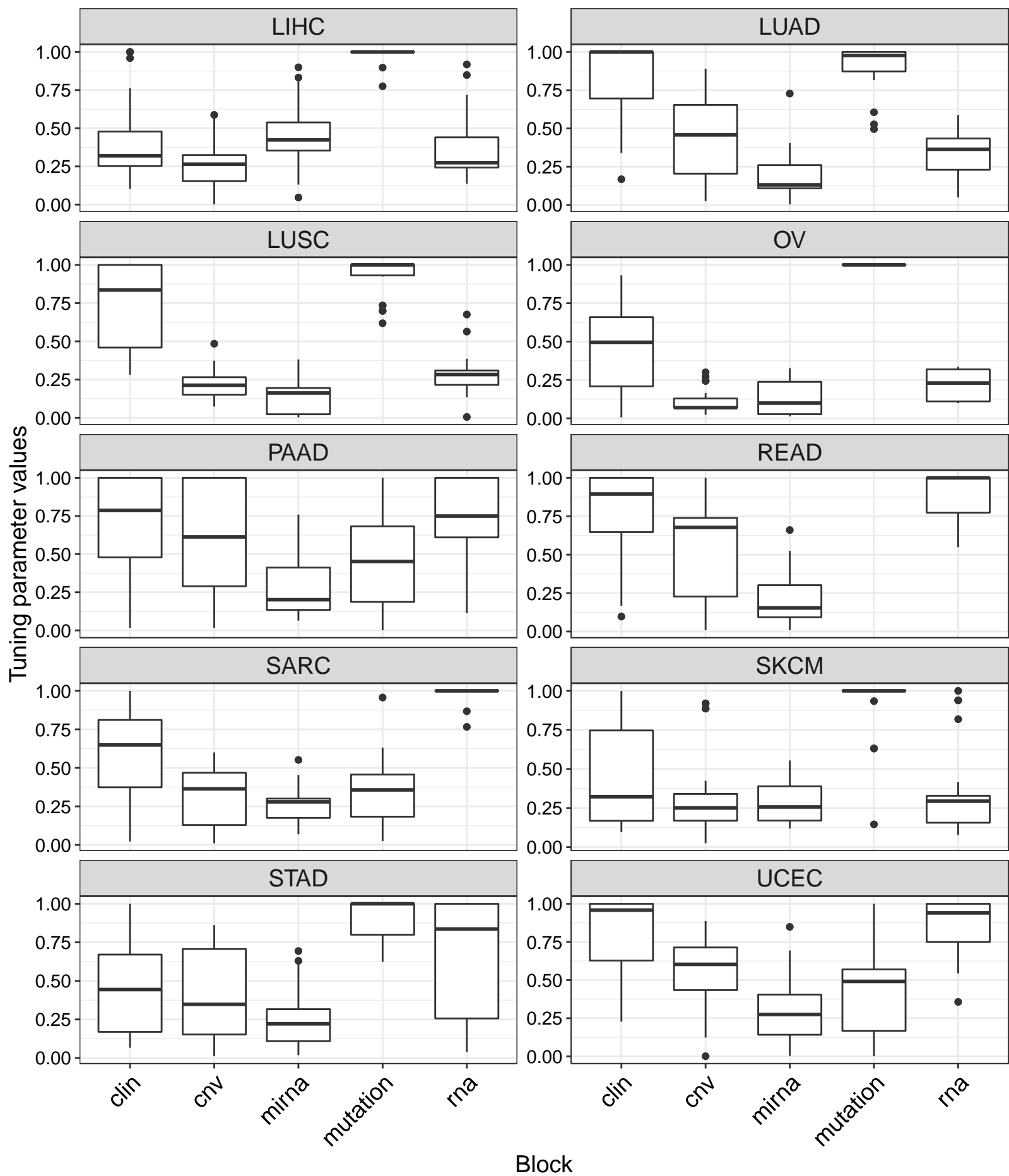

Supplement: Supplementary file 2 — Electronic Appendix. This folder contains all R Code written to perform the analyses presented in this paper and in Additional file 1 as well as Rda files enabling fast evaluation of the results. (ZIP 26,855 kb) [file 12859_2019_2942_MOESM2_ESM.zip › Additional_file_2_HornungWright/Figures/TunParam_BlockVarSel_2.pdf]

Tuning parameter values

BLCA

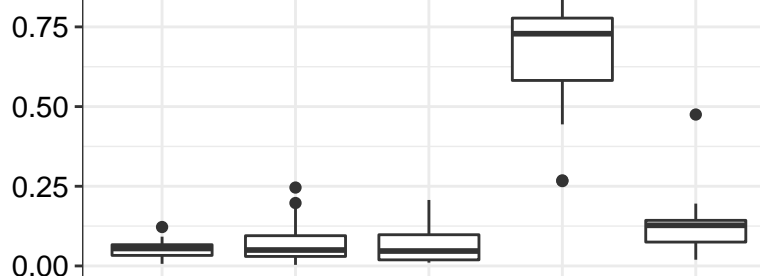

BRCA

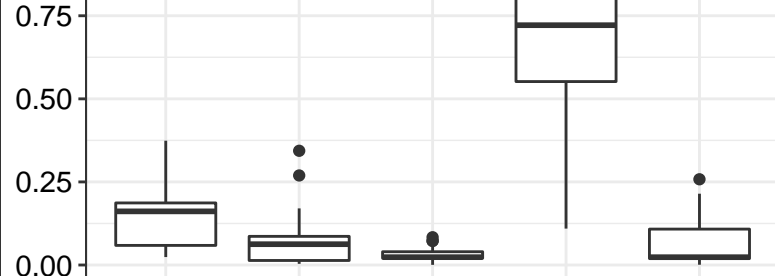

CESC

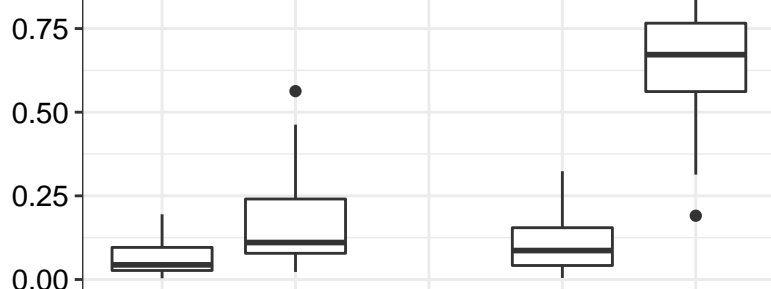

COAD

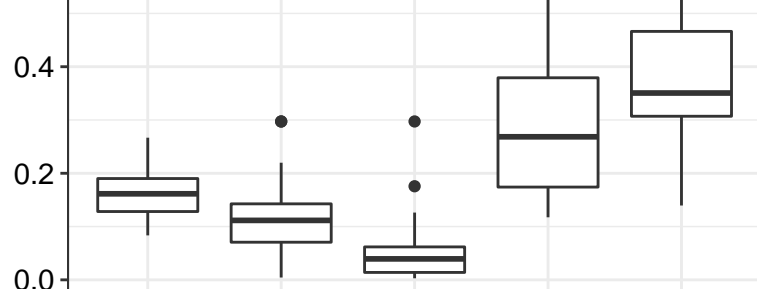

ESCA

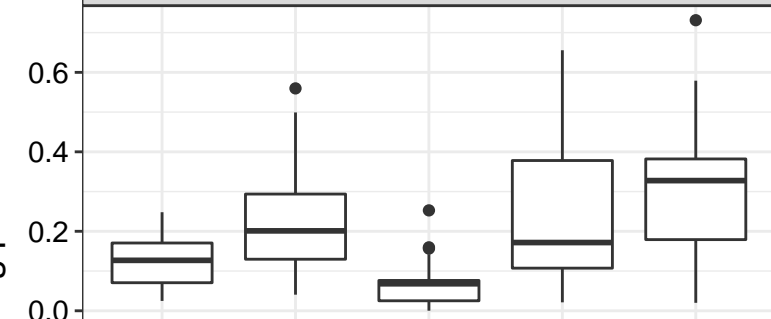

GBM

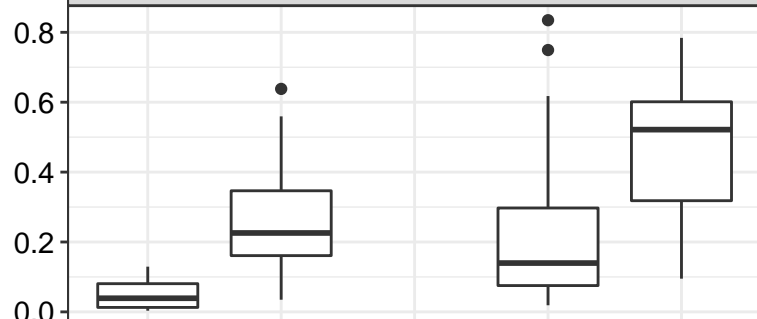

HNSC

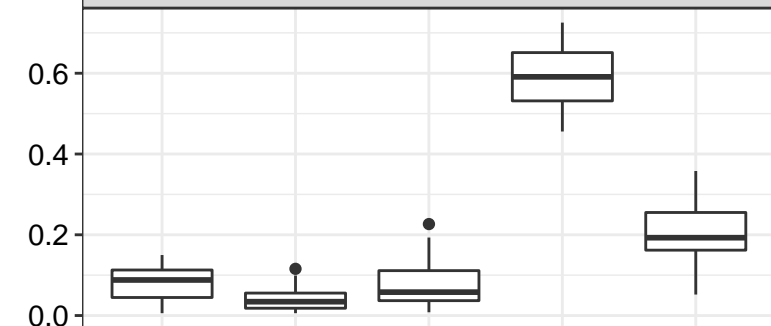

KIRC

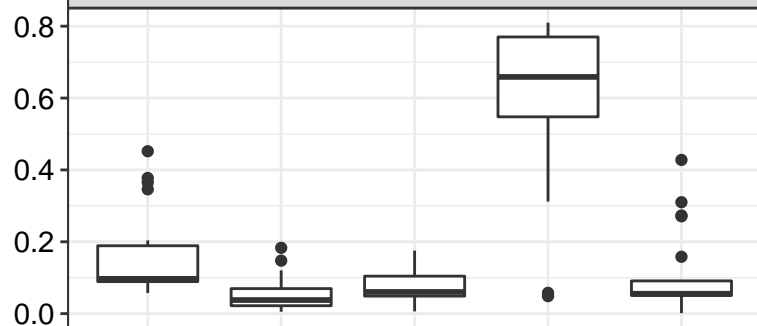

KIRP

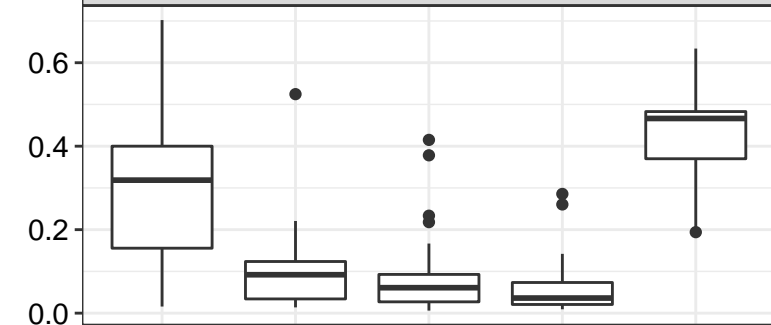

LGG

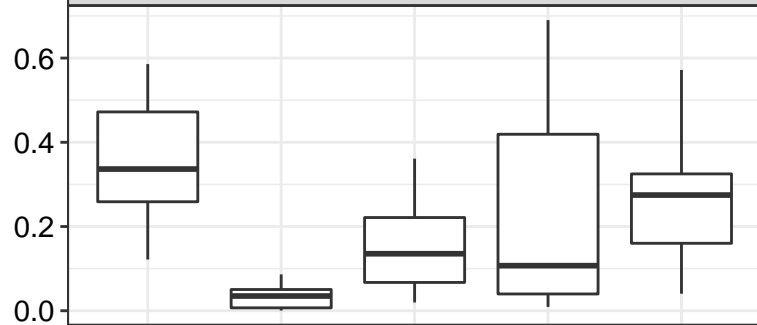

Block

Supplement: Supplementary file 2 — Electronic Appendix. This folder contains all R Code written to perform the analyses presented in this paper and in Additional file 1 as well as Rda files enabling fast evaluation of the results. (ZIP 26,855 kb) [file 12859_2019_2942_MOESM2_ESM.zip › Additional_file_2_HornungWright/Figures/TunParam_RandomBlock_1.pdf]

Tuning parameter values

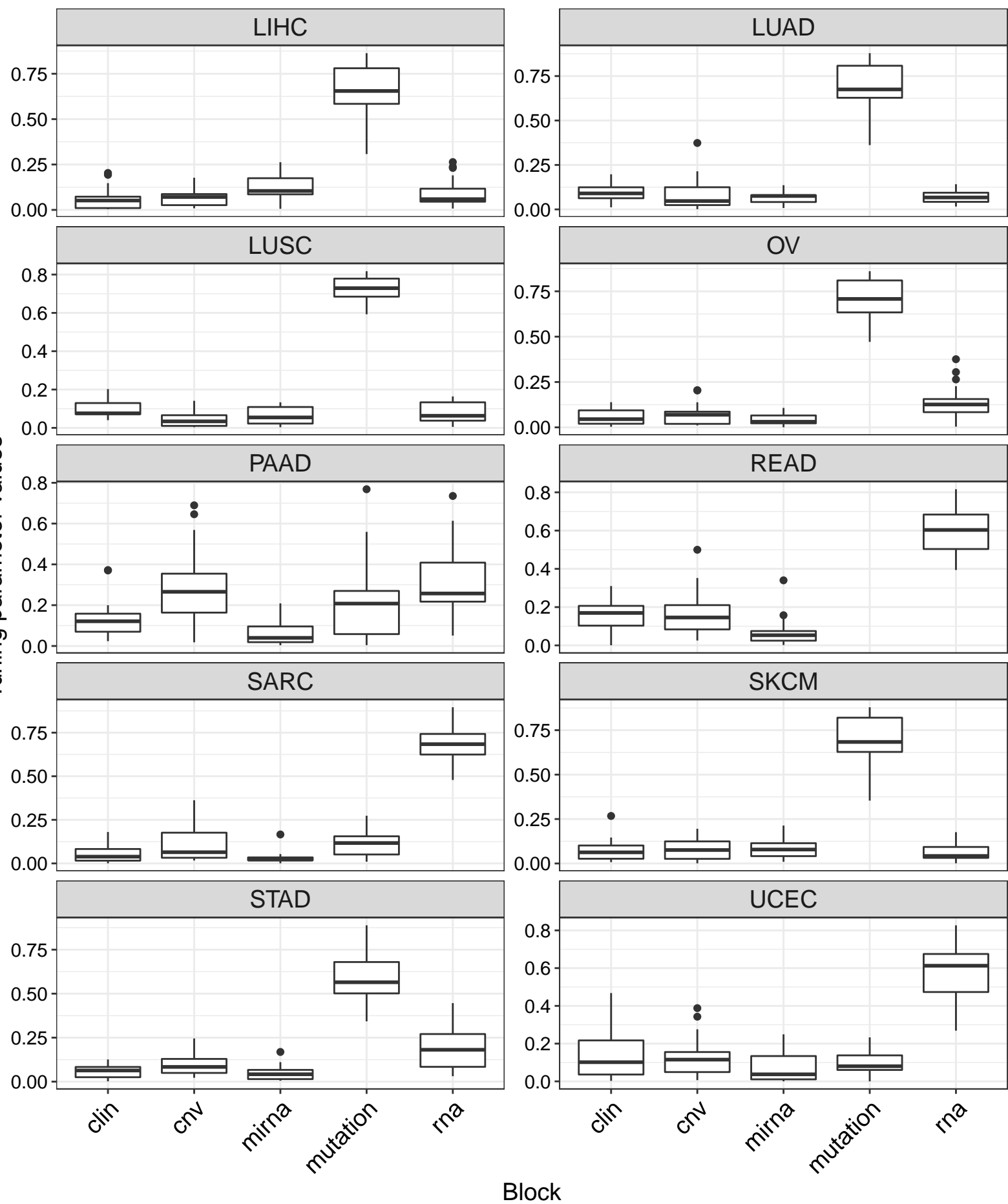

Supplement: Supplementary file 2 — Electronic Appendix. This folder contains all R Code written to perform the analyses presented in this paper and in Additional file 1 as well as Rda files enabling fast evaluation of the results. (ZIP 26,855 kb) [file 12859_2019_2942_MOESM2_ESM.zip › Additional_file_2_HornungWright/Figures/TunParam_RandomBlock_2.pdf]

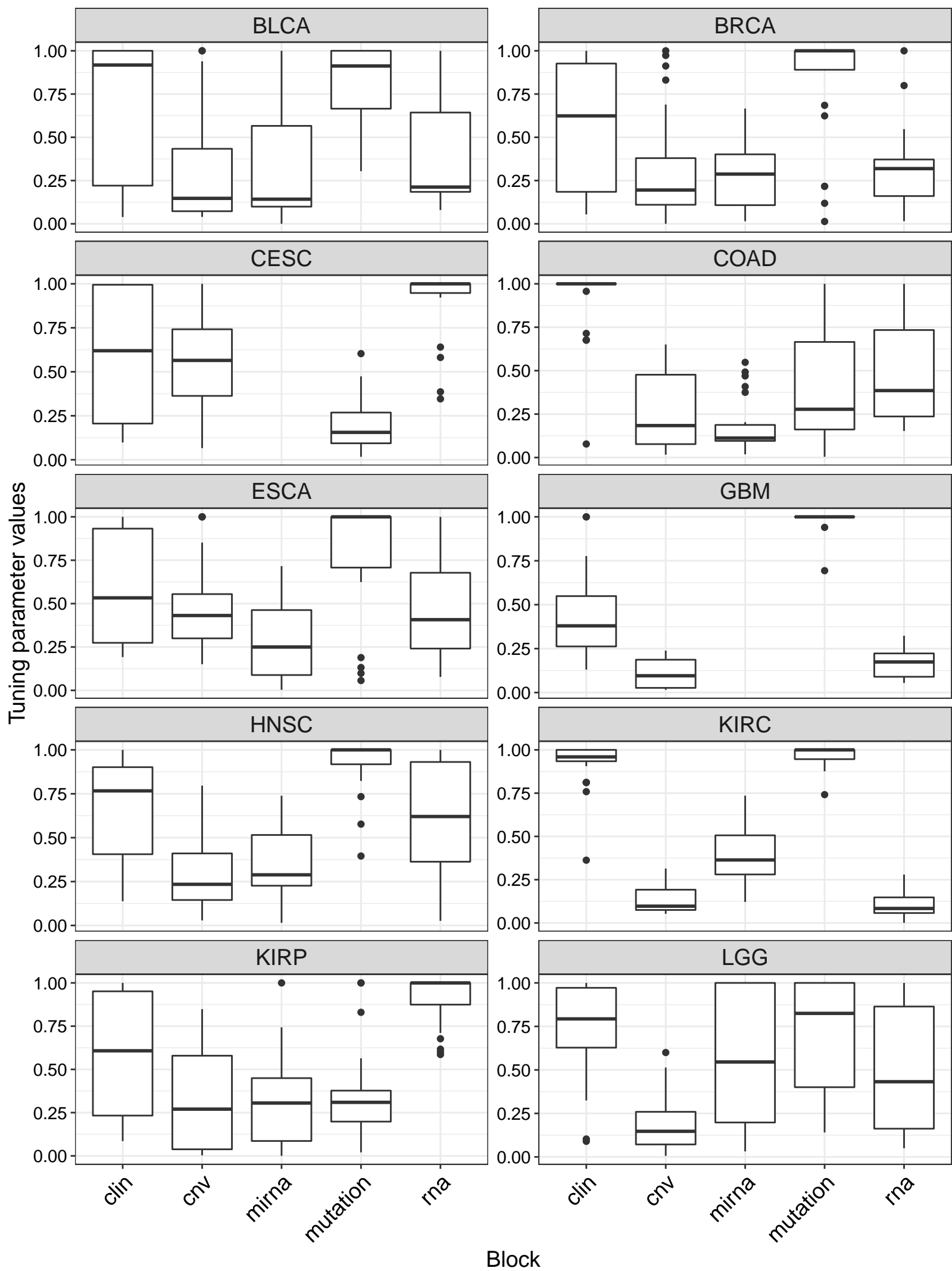

Supplement: Supplementary file 2 — Electronic Appendix. This folder contains all R Code written to perform the analyses presented in this paper and in Additional file 1 as well as Rda files enabling fast evaluation of the results. (ZIP 26,855 kb) [file 12859_2019_2942_MOESM2_ESM.zip › Additional_file_2_HornungWright/Figures/TunParam_SplitWeights_1.pdf]

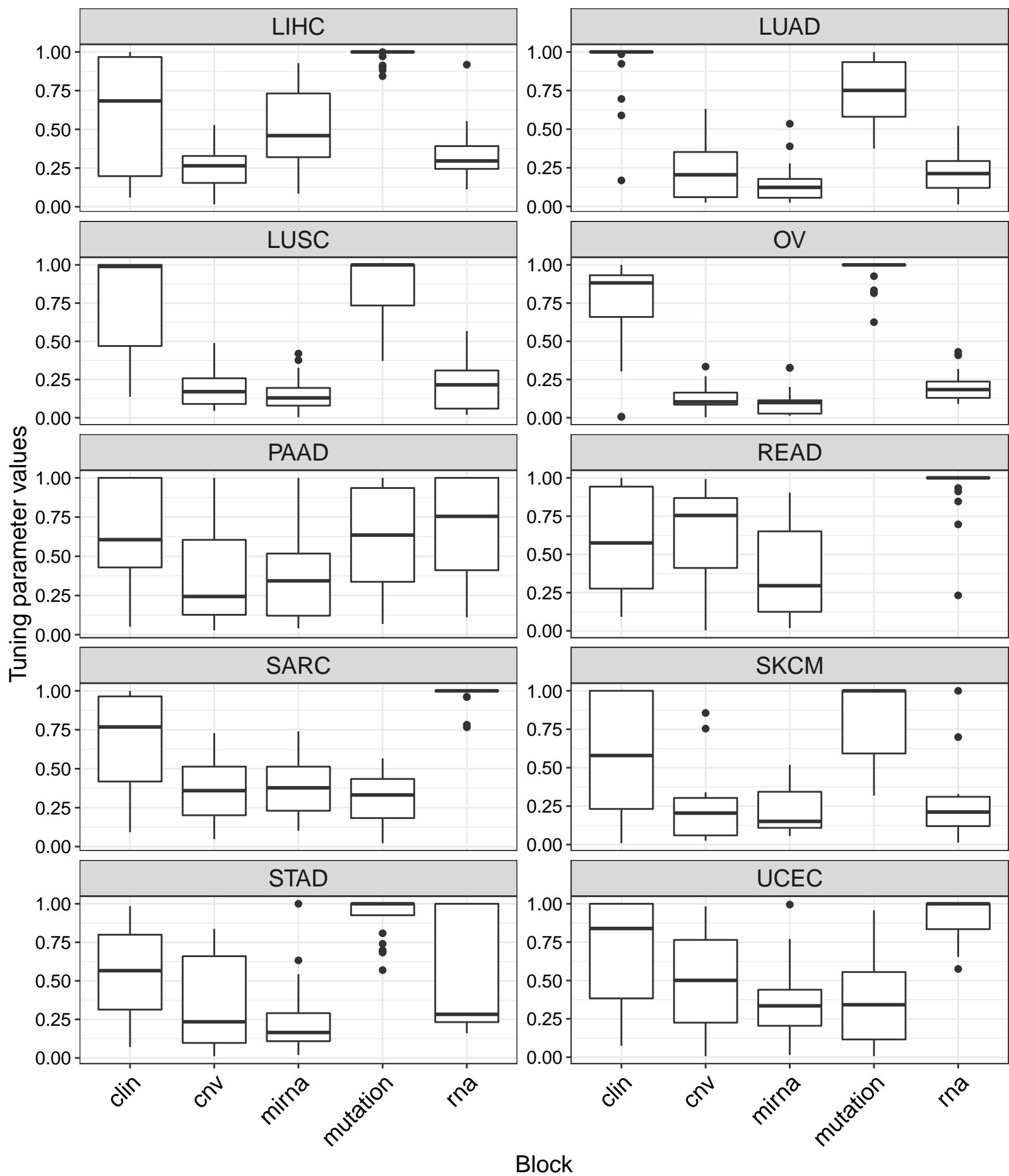

Supplement: Supplementary file 2 — Electronic Appendix. This folder contains all R Code written to perform the analyses presented in this paper and in Additional file 1 as well as Rda files enabling fast evaluation of the results. (ZIP 26,855 kb) [file 12859_2019_2942_MOESM2_ESM.zip › Additional_file_2_HornungWright/Figures/TunParam_SplitWeights_2.pdf]

Tuning parameter values

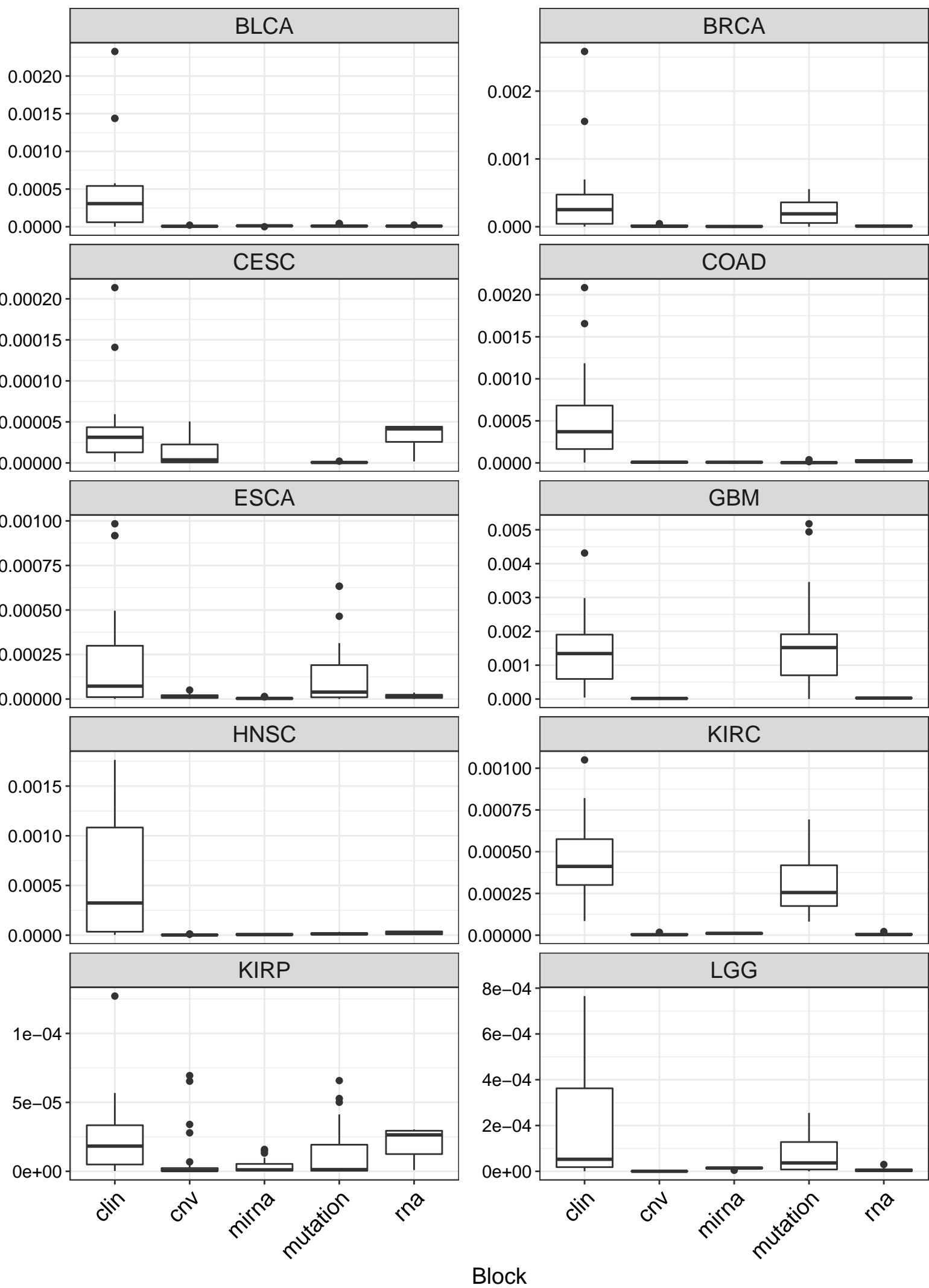

Supplement: Supplementary file 2 — Electronic Appendix. This folder contains all R Code written to perform the analyses presented in this paper and in Additional file 1 as well as Rda files enabling fast evaluation of the results. (ZIP 26,855 kb) [file 12859_2019_2942_MOESM2_ESM.zip › Additional_file_2_HornungWright/Figures/TunParam_VarProb_1.pdf]

Tuning parameter values

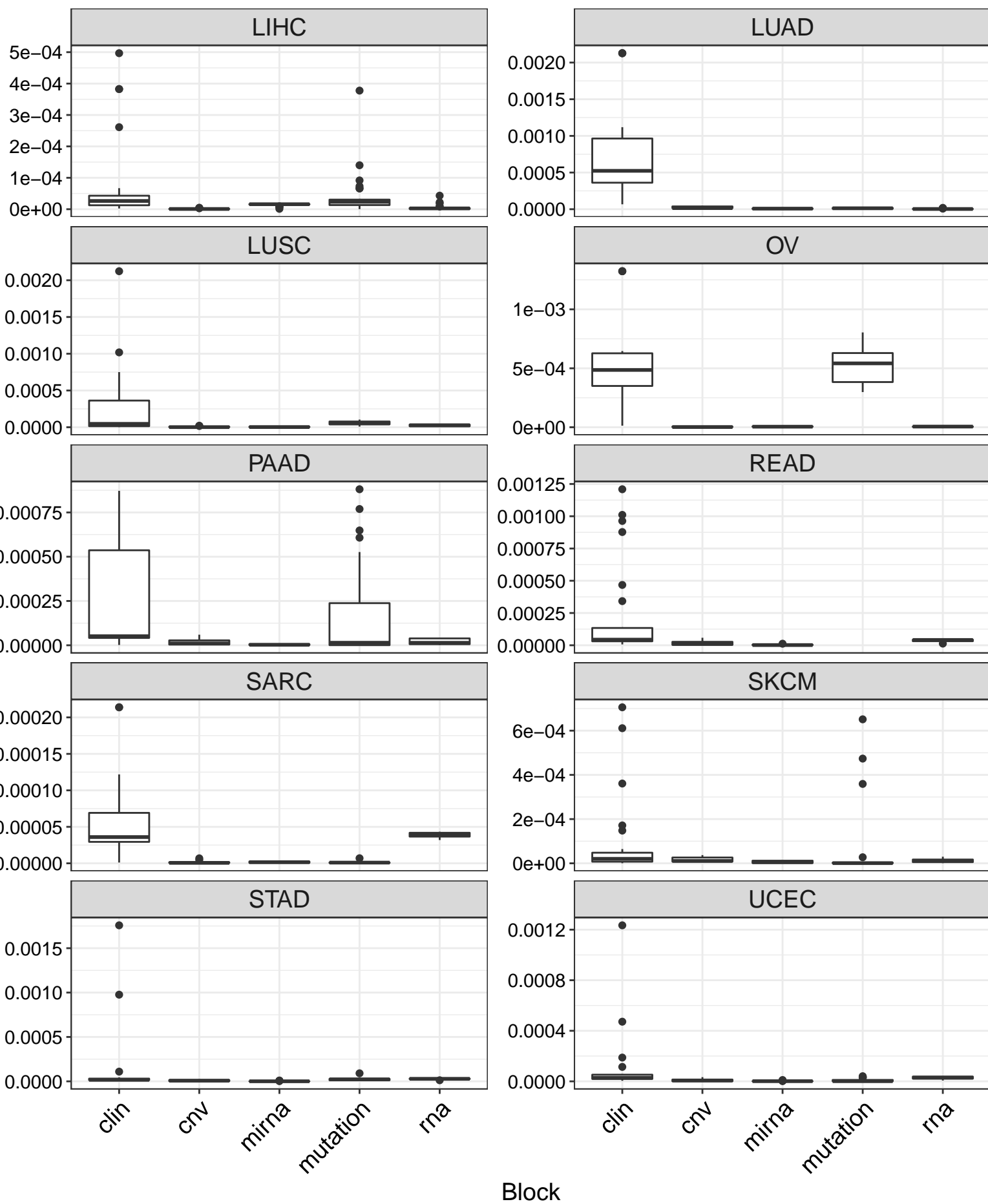

Supplement: Supplementary file 2 — Electronic Appendix. This folder contains all R Code written to perform the analyses presented in this paper and in Additional file 1 as well as Rda files enabling fast evaluation of the results. (ZIP 26,855 kb) [file 12859_2019_2942_MOESM2_ESM.zip › Additional_file_2_HornungWright/Figures/TunParam_VarProb_2.pdf]

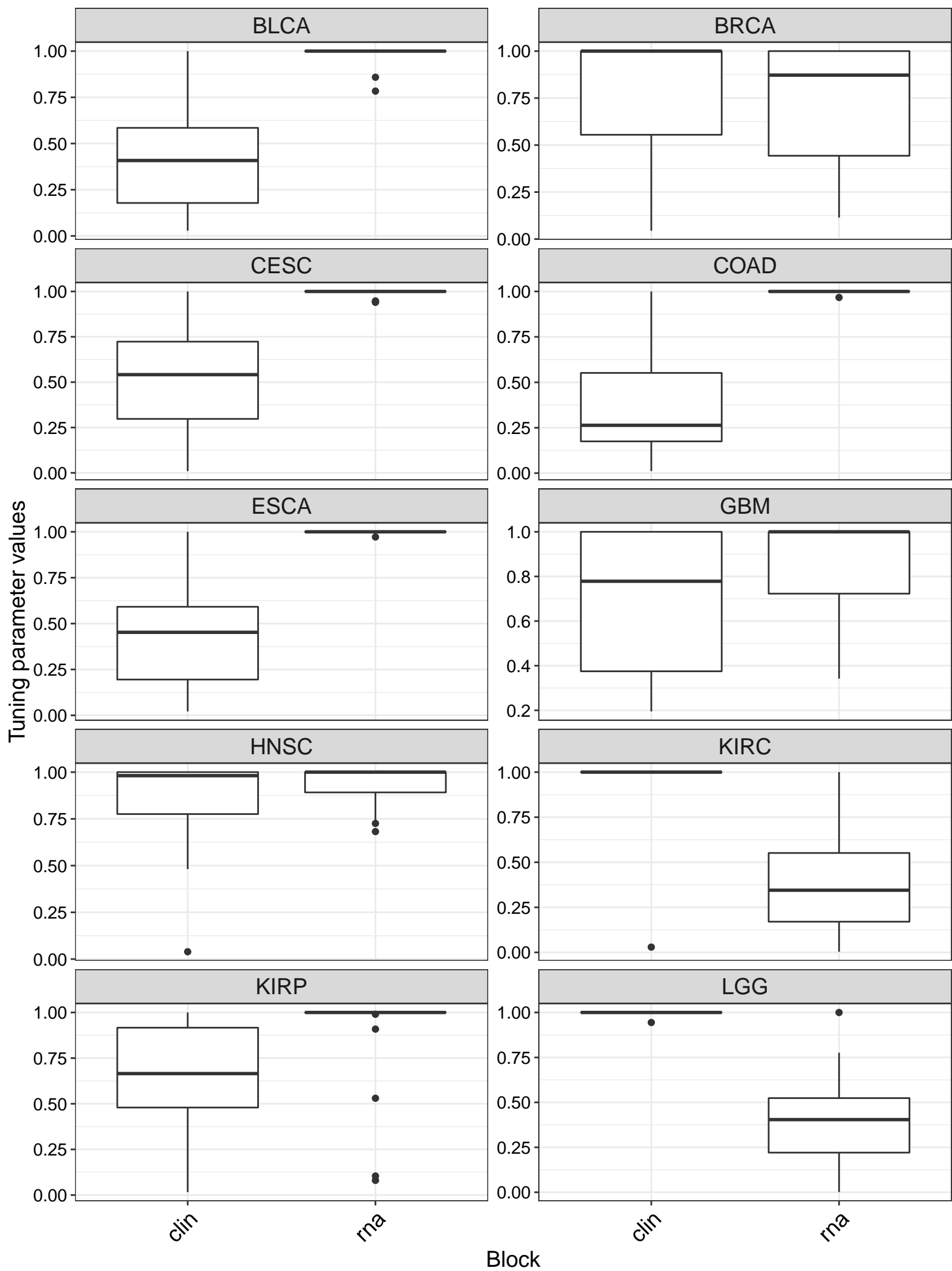

Supplement: Supplementary file 2 — Electronic Appendix. This folder contains all R Code written to perform the analyses presented in this paper and in Additional file 1 as well as Rda files enabling fast evaluation of the results. (ZIP 26,855 kb) [file 12859_2019_2942_MOESM2_ESM.zip › Additional_file_2_HornungWright/Figures/TunParamTwoBlocks_BlockForest_1.pdf]

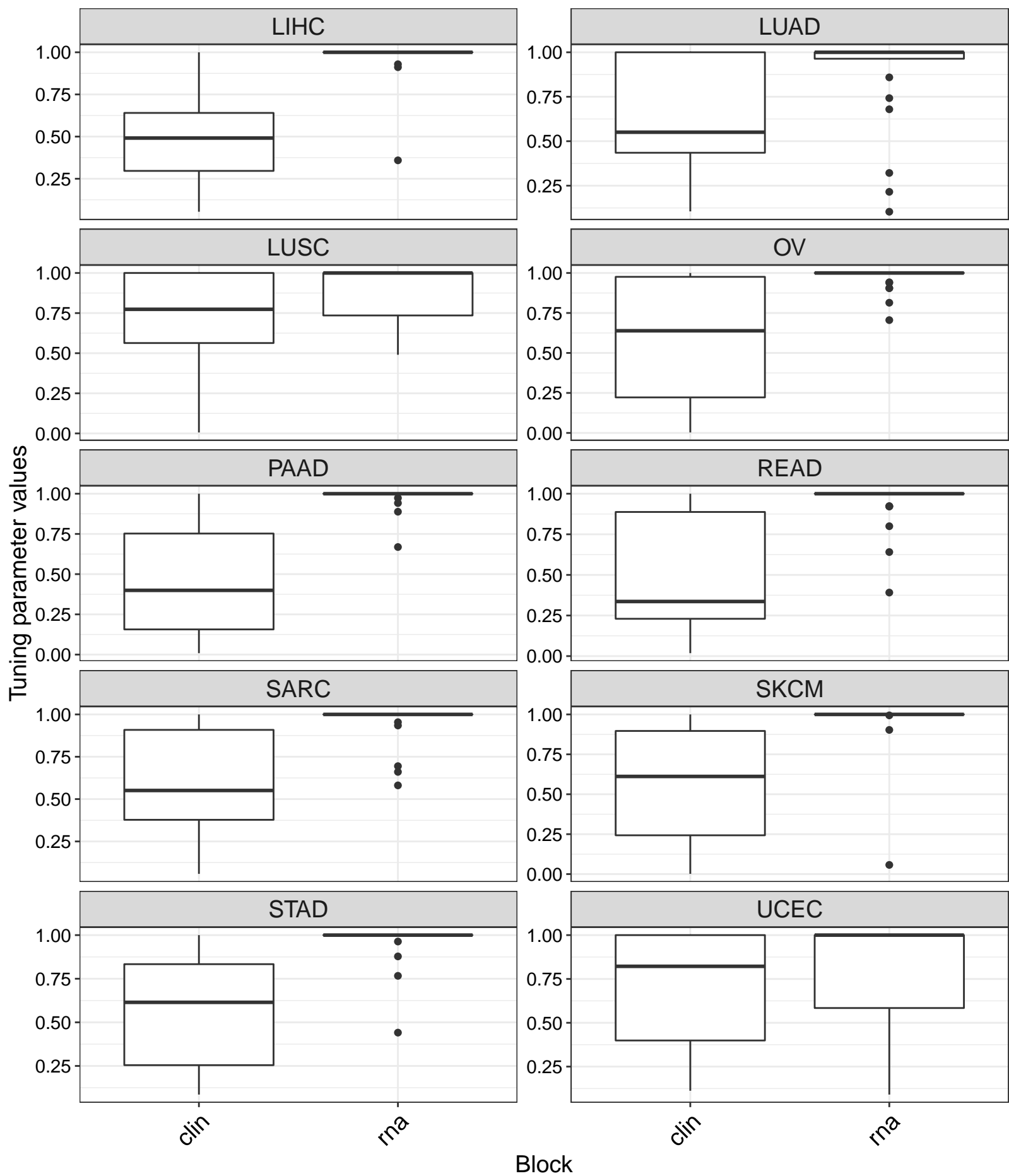

Supplement: Supplementary file 2 — Electronic Appendix. This folder contains all R Code written to perform the analyses presented in this paper and in Additional file 1 as well as Rda files enabling fast evaluation of the results. (ZIP 26,855 kb) [file 12859_2019_2942_MOESM2_ESM.zip › Additional_file_2_HornungWright/Figures/TunParamTwoBlocks_BlockForest_2.pdf]

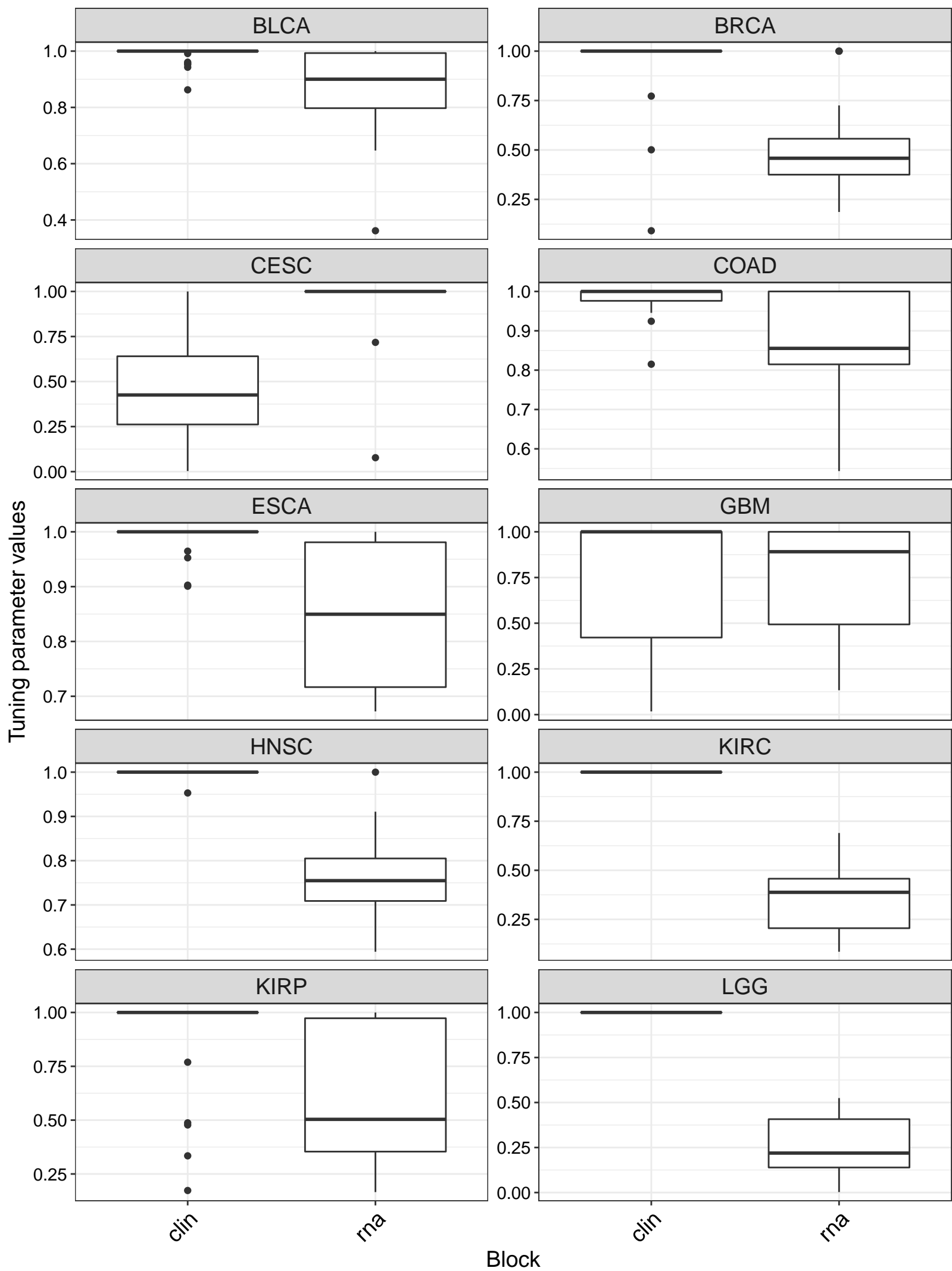

Supplement: Supplementary file 2 — Electronic Appendix. This folder contains all R Code written to perform the analyses presented in this paper and in Additional file 1 as well as Rda files enabling fast evaluation of the results. (ZIP 26,855 kb) [file 12859_2019_2942_MOESM2_ESM.zip › Additional_file_2_HornungWright/Figures/TunParamTwoBlocks_BlockVarSel_1.pdf]

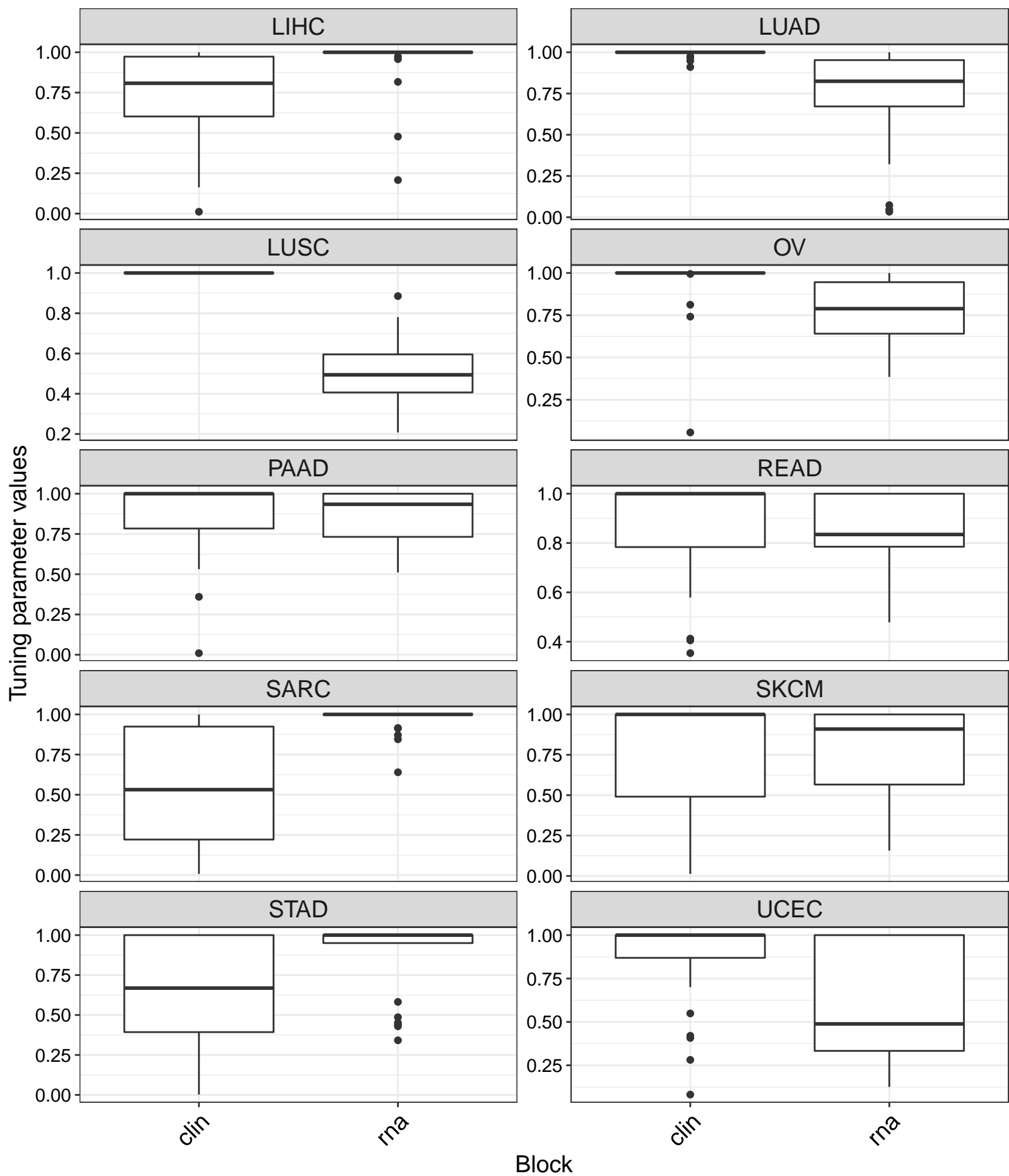

Supplement: Supplementary file 2 — Electronic Appendix. This folder contains all R Code written to perform the analyses presented in this paper and in Additional file 1 as well as Rda files enabling fast evaluation of the results. (ZIP 26,855 kb) [file 12859_2019_2942_MOESM2_ESM.zip › Additional_file_2_HornungWright/Figures/TunParamTwoBlocks_BlockVarSel_2.pdf]

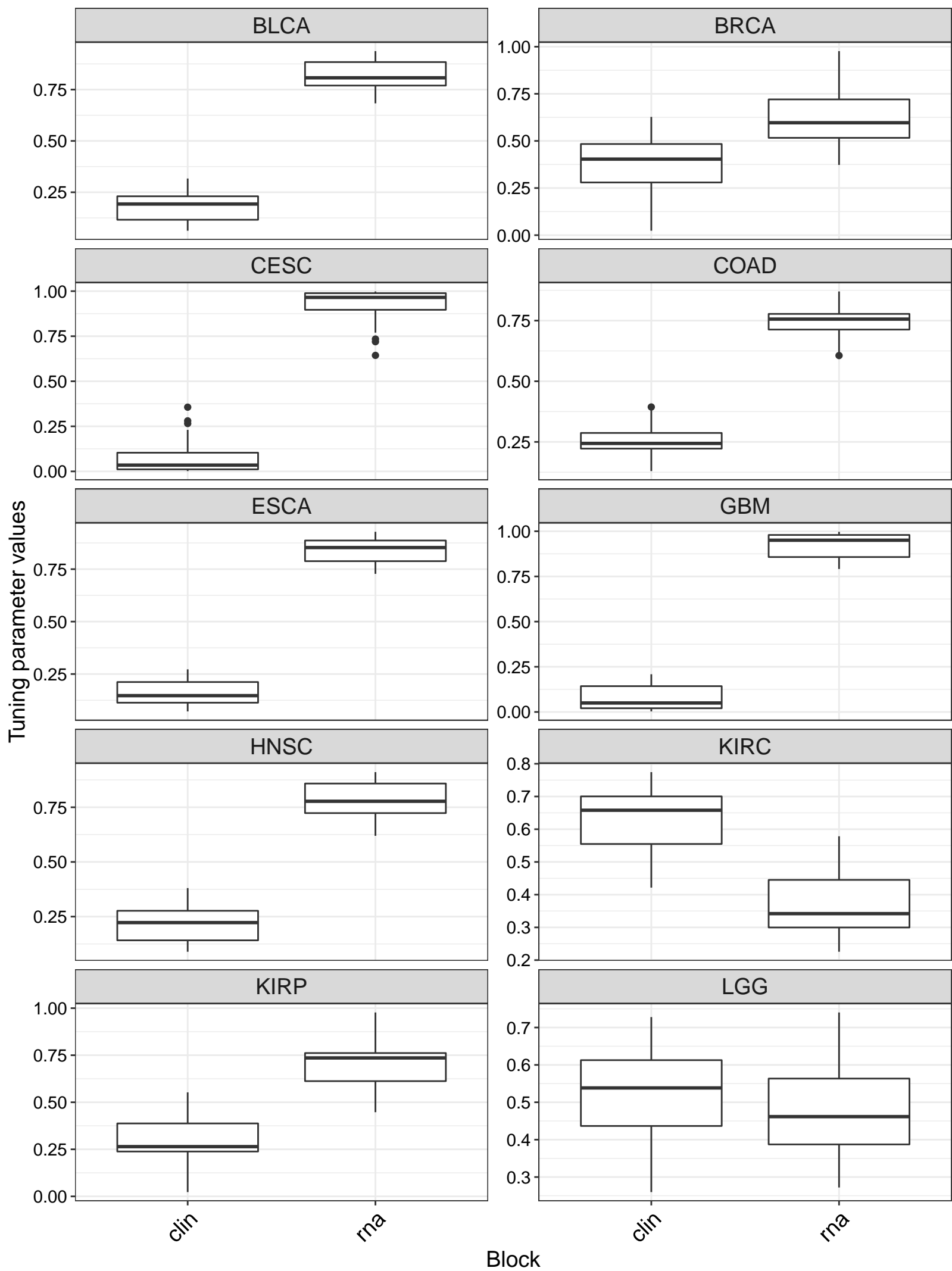

Supplement: Supplementary file 2 — Electronic Appendix. This folder contains all R Code written to perform the analyses presented in this paper and in Additional file 1 as well as Rda files enabling fast evaluation of the results. (ZIP 26,855 kb) [file 12859_2019_2942_MOESM2_ESM.zip › Additional_file_2_HornungWright/Figures/TunParamTwoBlocks_RandomBlock_1.pdf]

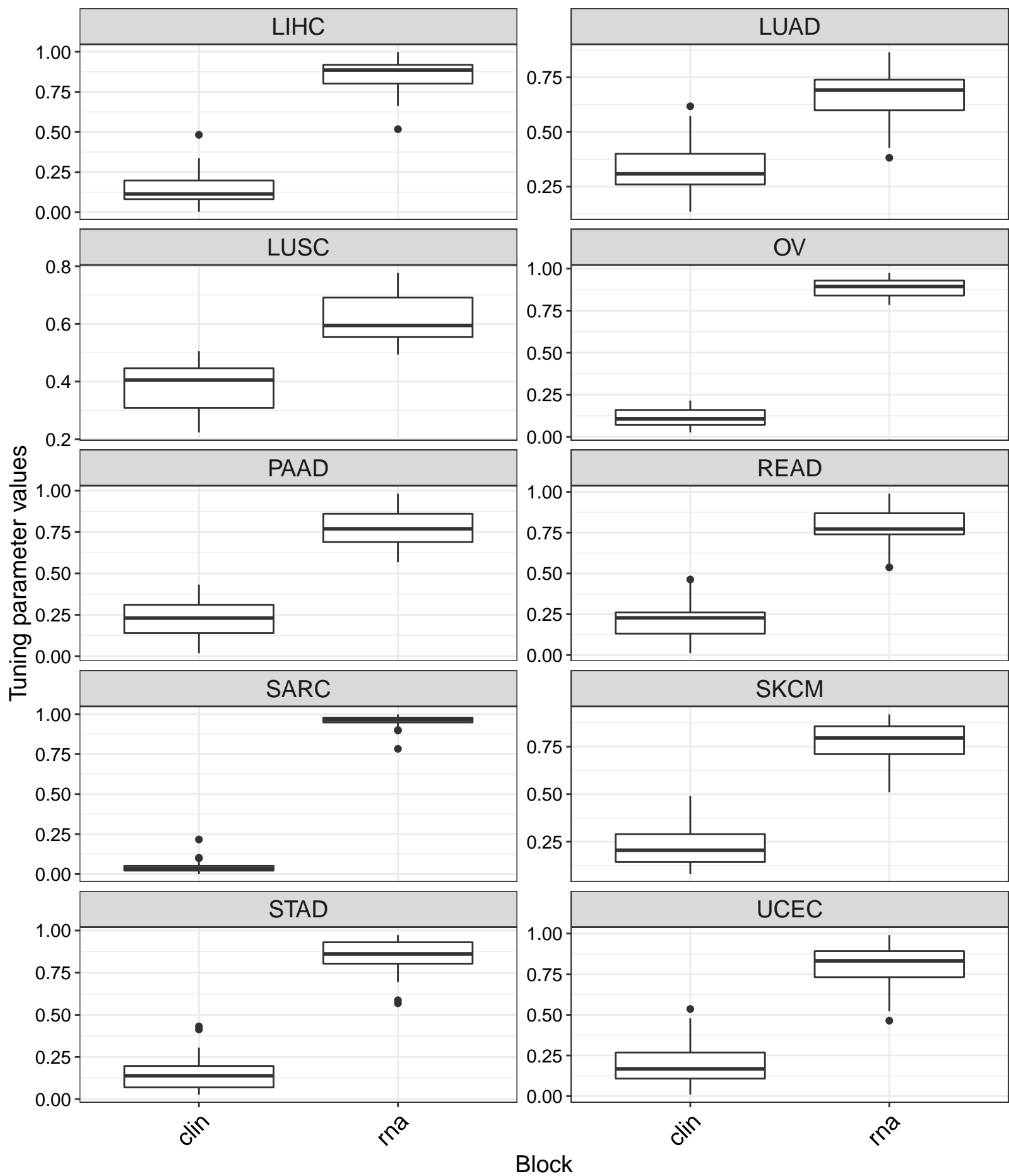

Supplement: Supplementary file 2 — Electronic Appendix. This folder contains all R Code written to perform the analyses presented in this paper and in Additional file 1 as well as Rda files enabling fast evaluation of the results. (ZIP 26,855 kb) [file 12859_2019_2942_MOESM2_ESM.zip › Additional_file_2_HornungWright/Figures/TunParamTwoBlocks_RandomBlock_2.pdf]

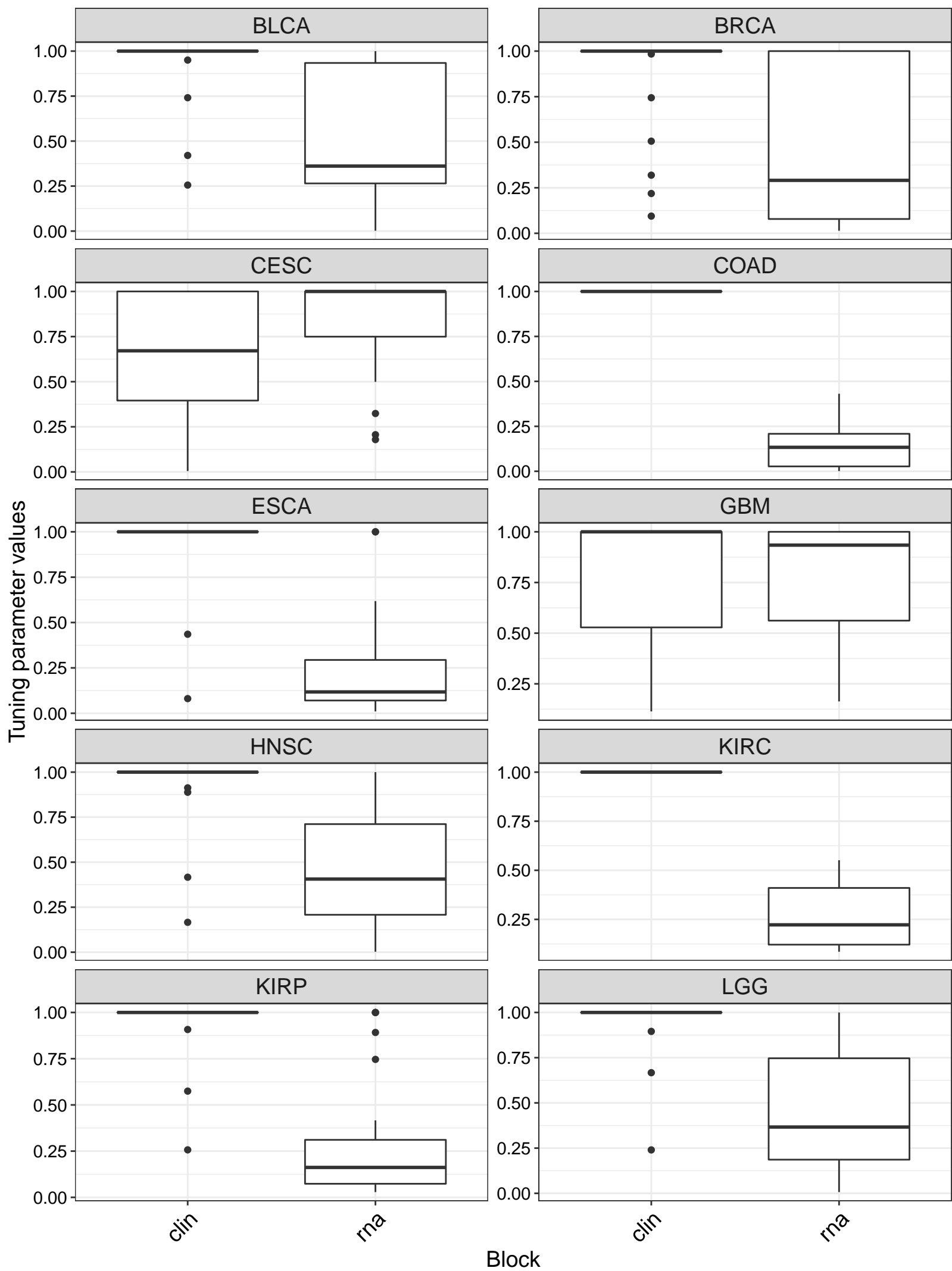

Supplement: Supplementary file 2 — Electronic Appendix. This folder contains all R Code written to perform the analyses presented in this paper and in Additional file 1 as well as Rda files enabling fast evaluation of the results. (ZIP 26,855 kb) [file 12859_2019_2942_MOESM2_ESM.zip › Additional_file_2_HornungWright/Figures/TunParamTwoBlocks_SplitWeights_1.pdf]

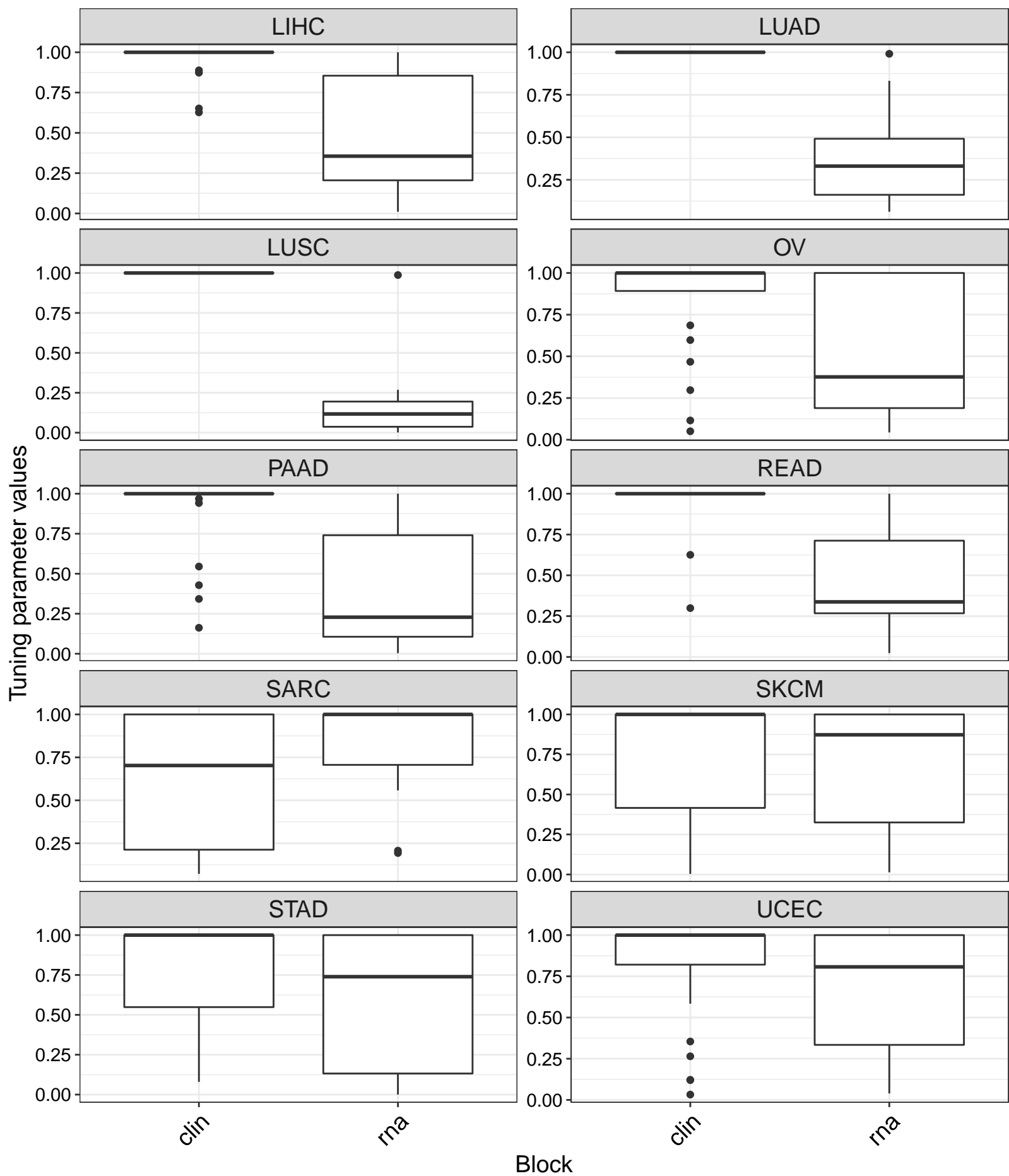

Supplement: Supplementary file 2 — Electronic Appendix. This folder contains all R Code written to perform the analyses presented in this paper and in Additional file 1 as well as Rda files enabling fast evaluation of the results. (ZIP 26,855 kb) [file 12859_2019_2942_MOESM2_ESM.zip › Additional_file_2_HornungWright/Figures/TunParamTwoBlocks_SplitWeights_2.pdf]

Tuning parameter values

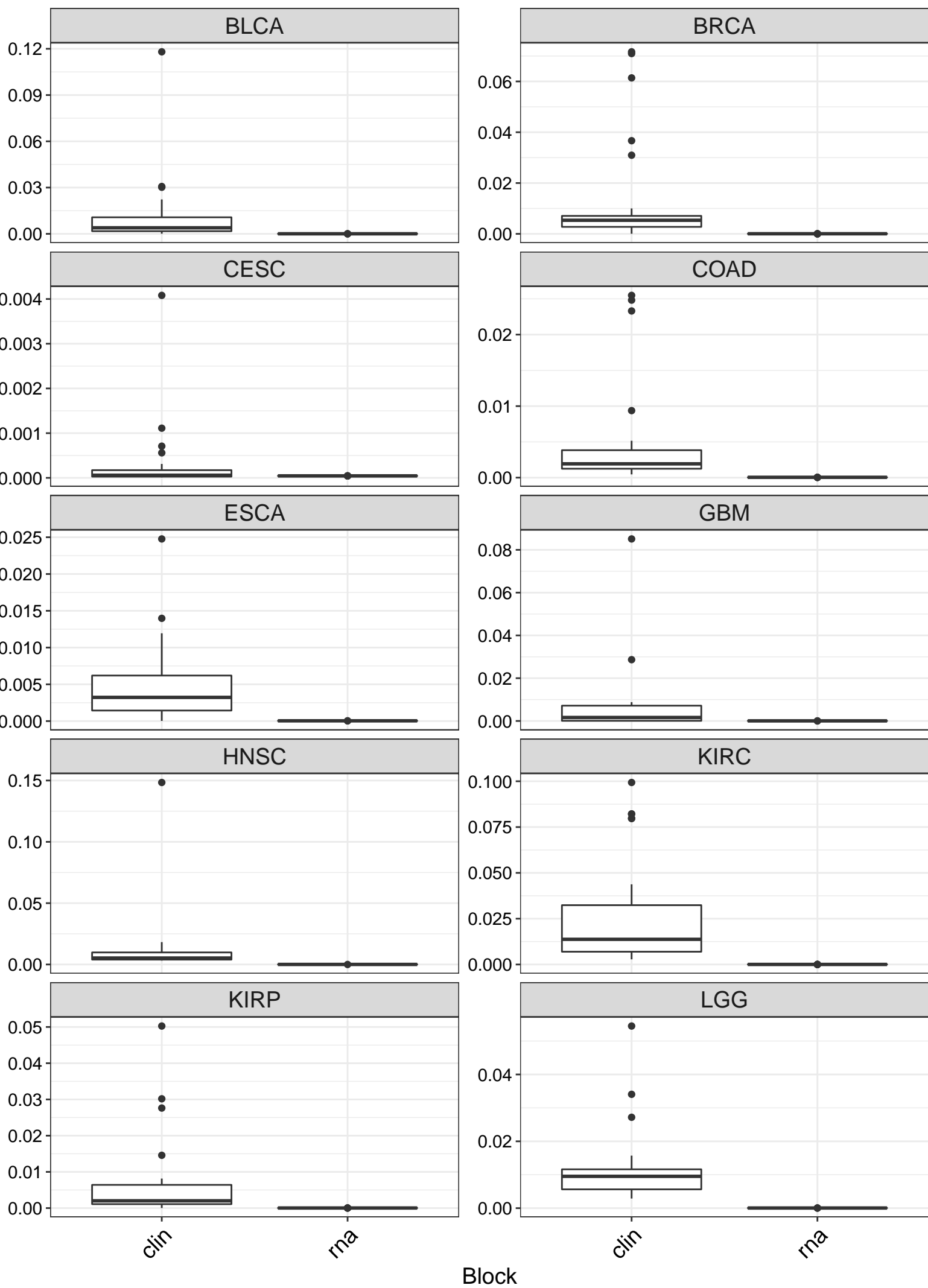

Supplement: Supplementary file 2 — Electronic Appendix. This folder contains all R Code written to perform the analyses presented in this paper and in Additional file 1 as well as Rda files enabling fast evaluation of the results. (ZIP 26,855 kb) [file 12859_2019_2942_MOESM2_ESM.zip › Additional_file_2_HornungWright/Figures/TunParamTwoBlocks_VarProb_1.pdf]

Tuning parameter values

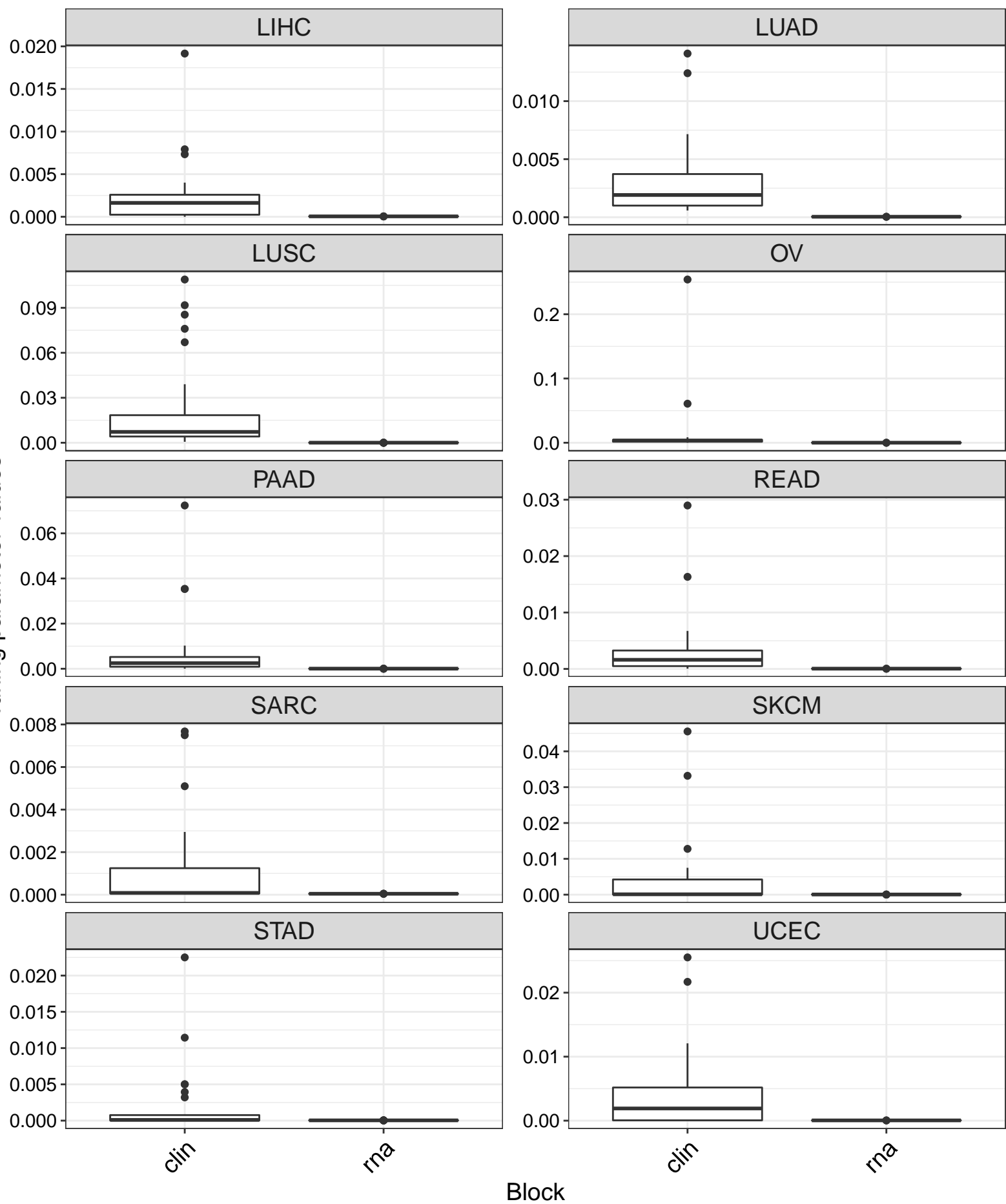

Supplement: Supplementary file 2 — Electronic Appendix. This folder contains all R Code written to perform the analyses presented in this paper and in Additional file 1 as well as Rda files enabling fast evaluation of the results. (ZIP 26,855 kb) [file 12859_2019_2942_MOESM2_ESM.zip › Additional_file_2_HornungWright/Figures/TunParamTwoBlocks_VarProb_2.pdf]
